# Mapping of homoeologous chromosome exchanges influencing quantitative trait variation in *Brassica napus*

**DOI:** 10.1111/pbi.12732

**Published:** 2017-04-27

**Authors:** Anna Stein, Olivier Coriton, Mathieu Rousseau‐Gueutin, Birgit Samans, Sarah V. Schiessl, Christian Obermeier, Isobel A.P. Parkin, Anne‐Marie Chèvre, Rod J. Snowdon

**Affiliations:** ^1^ Department of Plant Breeding IFZ Research Centre for Biosystems, Land Use and Nutrition Justus Liebig University Giessen Germany; ^2^ IGEPP INRA Agrocampus Ouest Université de Rennes 1 Le Rheu France; ^3^ Agriculture and Agri‐Food Canada Saskatoon Canada

**Keywords:** Genome rearrangements, homoeologous exchange, genetic mapping, quantitative trait loci, single nucleotide polymorphism

## Abstract

Genomic rearrangements arising during polyploidization are an important source of genetic and phenotypic variation in the recent allopolyploid crop *Brassica napus*. Exchanges among homoeologous chromosomes, due to interhomoeologue pairing, and deletions without compensating homoeologous duplications are observed in both natural *B. napus* and synthetic *B. napus*. Rearrangements of large or small chromosome segments induce gene copy number variation (CNV) and can potentially cause phenotypic changes. Unfortunately, complex genome restructuring is difficult to deal with in linkage mapping studies. Here, we demonstrate how high‐density genetic mapping with codominant, physically anchored SNP markers can detect segmental homoeologous exchanges (HE) as well as deletions and accurately link these to QTL. We validated rearrangements detected in genetic mapping data by whole‐genome resequencing of parental lines along with cytogenetic analysis using fluorescence *in situ* hybridization with bacterial artificial chromosome probes (BAC‐FISH) coupled with PCR using primers specific to the rearranged region. Using a well‐known QTL region influencing seed quality traits as an example, we confirmed that HE underlies the trait variation in a DH population involving a synthetic *B. napus* trait donor, and succeeded in narrowing the QTL to a small defined interval that enables delineation of key candidate genes.

## Introduction

*Brassica napus* (rapeseed, oilseed rape, canola) is a very recent allopolyploid species that since its origin has become one of the world's most important crops. The species was formed by hybridization and genome doubling from the diploid donor genomes of *Brassica oleracea* and *Brassica rapa*, respectively. Because this cross can be readily reproduced with the help of embryo rescue techniques, *B. napus* has become a popular model for studying the genetic and genomic consequences of *de novo* allopolyploidization and how these have shaped natural and agricultural selection in a modern crop (Mason and Chèvre, 2016).

Segmental exchanges between homoeologous chromosomes are frequent throughout the *B. napus* genome. Numerous small‐scale homoeologous exchanges (HE) are observed throughout the genomes of natural *B. napus* accessions, whereas large‐scale HE are common in synthetic accessions (Chalhoub *et al*., 2014; Rousseau‐Gueutin *et al*., 2017). In fact, resynthesized lines are specifically prone to homoeologous rearrangements, including deletions, duplications and translocations (Gaeta *et al*., 2007; Szadkowski *et al*., 2010; Xiong *et al*., 2011). Other than the assumption that resynthesized rapeseed displays some kind of accelerated oilseed rape evolution, it is unknown whether different mechanisms take place in natural and resynthesized oilseed rape.

Implementation of synthetic *B. napus* in breeding is an interesting, yet challenging strategy to overcome the extreme narrow genetic diversity in modern rapeseed breeding pools. Causes of the genetic bottlenecks are the small number of founder allopolyploidization events during the origin of the *B. napus* species (Allender and King, 2010), strong adaptive selection in strict eco‐geographic gene pools and intensive agronomic selection during recent breeding for essential seed quality traits (Snowdon *et al*., 2015). The diploid progenitor species harbour important variation particularly for disease resistance (e.g. Mei *et al*., 2015; Rygulla *et al*., 2007a,b; Werner *et al*., 2008) and improvement of the heterotic potential (Snowdon *et al*., 2015). Unfortunately, the rich genetic diversity available through *de novo* resynthesis of *B. napus* carries the price of a heavy genetic load, with poor fertility and agronomic performance. Although marker‐assisted backcrossing can accelerate incorporation of such exotic materials, the complex genome restructuring common in synthetic *B. napus* makes such germplasm particularly difficult to deal with in linkage mapping studies.

Furthermore, because most marker systems commonly used for genetic mapping do not assay presence–absence variation (PAV) or copy number variation (CNV) automatically, the extent to which this kind of variation underlies QTL for important agronomic traits is still completely unknown in *B. napus*. Most genetic linkage maps constructed using high‐density SNP array data do not consider CNV or PAV (e.g. Delourme *et al*., 2013; Fopa *et al*., 2014; Liu *et al*., 2013; Raman *et al*., 2014) although both are inherent phenomena in chromosome regions shaped by HEs or deletions. The consequence may be that genome regions affected by genomic rearrangements including deletions, which sometimes span entire chromosomes in synthetic *B. napus* (Chalhoub *et al*., 2014), may not be incorporated into genetic maps and result in large gaps in linkage groups.

Accurate mapping of genomic rearrangement events is essential for mapping of associated QTL and evaluation of their impact in allopolyploid crops. Mason *et al*. (2017) proposed guidelines for scoring of SNP calling results that suggest presence–absence variation (PAV), corresponding with the expected segregation ratio, in a segregating mapping population. Today, high‐density SNP data array, like that generated using the Brassica 60K Illumina Infinium genotyping array (Clarke *et al*., 2016), enable rapid, high‐resolution mapping of large *B. napus* mapping populations at low cost. Due to the large numbers of polymorphic markers that can be assayed using this array, it is easy for users to generate highly dense genetic linkage maps, even when markers that show unexpected segregation or excessive quantities of failed SNP calls are excluded.

Similarly, hemi‐SNPs (Trick *et al*., 2009) are frequently encountered between homoeologous loci in *B. napus*. A hemi‐SNP results from either (i) a simultaneous hybridization of the marker to two homoeologous loci or (ii) a simultaneous hybridization of the marker to duplicated fragments containing a SNP mutation, where only one locus in each instance is polymorphic. These variants generally exceed common threshold limits for tolerated heterozygosity in segregating mapping populations, meaning that hemi‐SNP data tend to be automatically discarded from genetic mapping data sets. On the other hand, inclusion of expected segregation ratios between heterozygous and homozygous individuals enables genotypes to be resolved at a single‐locus level and used for map calculation in a mapping population.

Physical anchoring of SNP loci provides positional information that can improve confidence in calling of deletion and duplication events based on missing SNP calls or hemi‐SNPs, respectively. Although missing calls due to technical failures are reasonably common in array‐based genotyping systems, and repeated failure of the same SNP may point to technical problems with the assay, it is unlikely that two or more physically adjacent SNPs will by chance show the same segregation patterns of technical failures vs. successful amplifications of one or the other SNP allele. Similarly, the calling of a hemi‐SNP does not necessarily indicate presence of a duplication, but may also occur as the consequence of unspecific SNP probe hybridization. Nevertheless, using positionally anchored markers in genetic mapping indicates putatively rearranged genomic regions. Those regions may be validated by genomic resequencing.

Mapping of short‐read genomic resequencing data to the recently published *B. napus* reference genome sequence (Chalhoub *et al*., 2014) enables comprehensive analysis regarding nucleotide polymorphisms and gene content, but also and importantly identification of presence–absence variation (PAV) in *B. napus* samples. However, neither reciprocal nor nonreciprocal rearrangements between the homoeologous subgenomes can be detected from resequencing data *per se*. Nevertheless, segmental deletions that show corresponding duplications of the homoeologous chromosome segment imply that this incidence is a homoeologous exchange (HE).

Fluorescence *in situ* hybridization using bacterial artificial chromosome probes (BAC‐FISH) in *B. napus* enables chromosome painting using BAC probes containing subgenome‐specific repeat sequences (Leflon *et al*., 2006), or even molecular karyotyping based on chromosome‐specific probes (Xiong and Pires, 2011). Multicolour combination of chromosome‐specific BACs and subgenome‐specific BACs enables unambiguous chromosome identification *in situ*. Thus, comparing the combination of FISH signals from specific BAC clones within putative HE regions can distinguish individuals, carrying single copies of the exchanged region, from individuals with duplicated copies of one homoeologous locus and/or deleted copies of the other homoeologue. BAC‐FISH therefore represents an interesting method for independent validation of HE events imputed from genomic sequencing reads or molecular marker segregation data. As homoeologous reciprocal translocations cannot be validated by genome resequencing, however in principle they can be detected by genetic mapping and BAC‐FISH.

## Results

### Natural and synthetic *B. napus* parental genotypes exhibit widespread genomic rearrangements

The aligned resequencing data yielded a generally uniform coverage over the lengths of each chromosome in the respective mapping parents. This facilitated calling of putatively deleted and duplicated segments, which showed consistent patterns of coverage either lower or higher than the chromosome‐wide average and a minimum length of 50 kb. As described by Chalhoub *et al*. (2014), we found widespread evidence for deletions, duplications and HEs among homoeologous A‐subgenome and C‐subgenome chromosomes. Also, as expected, the natural *B. napus* accession Express 617 showed the lowest degree of segmentation (685 deleted or duplicated segments), whereas the two synthetic *B. napus* parents exhibited a considerably higher segmentation degree (821 and 1,630 deletions or duplications, for 1012‐98 and R53, respectively). The semisynthetic parent V8, derived from backcrossing of a synthetic *B. napus* to natural *B. napus*, showed the expected intermediate degree of segmentation (795 segments), between that of the synthetic and natural accessions. In summary, Express 617 showed genomic rearrangement events affecting 8.0% of the genome, 1012‐98 16.2% of the genome, V8 12.3% of the genome and R53 41.5% of the genome. Although these figures may be biased by normalization based on chromosome mean coverage values and alignment to a European winter–oilseed rape reference sequence (Darmor‐*bzh*), the overall scale of rearrangements is clearly greater in synthetic *B. napus*.

As an overview, Figure S1 displays locations of segmental deletions or duplications larger than 500 kb in the four mapping parents. Full details of all detected segmental deletions and duplications, also including those events between 50 kb and 500 kb in size, are provided in Table S1. Coverage plots for the 19 chromosomes of the four parental genotypes are given in Figures S2, S3, S4 and S5.

In Express 617, only five deletions larger than 500 kb were detected, three on chromosome C01, one on chromosome C02 and one on C08. In contrast, duplications and deletions larger than 500 kb were numerous and widespread across both subgenomes in the synthetic and semisynthetic accessions: 22 events in V8, 40 events in 1012‐98 and 107 events in R53. Interestingly, deletions in five genomic regions on chromosomes A03, C02, C05, C07 and C09 were consistently detected in all three parental accessions with synthetic *B. napus* background.

Chromosome C02 in the genotype R53 was not represented by sequence reads and is therefore assumed to be missing (completely or almost completely). On the other hand, chromosome number of R53 was confirmed cytogenetically to be 38 (data not shown). Due to the elevated coverage of sequence reads from chromosome A02 in R53, we assume that chromosome C02 has been replaced by a duplication of A02. Deletions and duplications affecting whole or nearly whole chromosomes have also been observed by Rousseau‐Gueutin *et al*. (2017) in other synthetic *B. napus*.

### Genetic mapping

The total amount of SNP markers used to calculate the genetic bin maps was reduced based on allele frequency and cosegregation from 35 170 preselected SNPs, with putative unique positions in the reference sequence, to sets of 2204, 3135 and 2029 markers for the populations ExV8‐DH, ExR53‐DH and Ex1012‐98‐DH, respectively. The resulting genetic linkage maps comprised 1733, 2186 and 1631 markers, respectively, covering 2512 cM, 3780 cM and 2358 cM over 20, 21 and 22 linkage groups. A total of 273 consensus markers were found in all three populations, with 704 consensus markers between ExV8‐DH and Ex1012‐98‐DH, 729 between ExV8‐DH and ExR53‐DH and 677 between ExV8‐DH and ExR53‐DH, respectively.

High collinearity was achieved among the three linkage maps, whereby the linkage groups in the ExR53‐DH map were generally larger than those in the other two populations, reflecting the considerably greater number of markers. Lengths of individual linkage groups vary among the three populations, indicating differential degrees of diversity and recombination across the chromosomes of the synthetic parents 1012‐98, V8 and R53. All linkage maps are displayed in Figure S6; the map texts can be found in Table S2.

Notably, some linkage groups were found to be considerably longer in a single population than in the other two. Because all populations had mapping parent Express 617 in common, this could indicate a propensity for higher recombination frequency on these specific chromosomes conferred by the particular synthetic/semisynthetic mapping parent.

We also observed frequent incidents where multiple markers assigned to the same chromosome in the Darmor‐*bzh* reference were genetically mapped onto two linkage groups that could not be combined into a single linkage group. The data suggest that this phenomenon, which is common in *B. napus* genetic maps, corresponds with the presence of either large or numerous genomic rearrangements in one of the synthetic or semisynthetic mapping parents and a consequent disruption of pairing and linkage disequilibrium in the DH lines. ‘noncontiguous‘ linkage groups are distinguishable from translocated genomic segments, because the latter map to their new position. In this study, Ex1012‐98DH exhibits two noncontinuous linkage groups representing chromosome A01. Additionally, a duplicated and translocated A01‐fragment of 1012‐98 maps to its new position in linkage group C01.

### Genomic rearrangements can be localized by genetic mapping

Calling of ‘het’ and ‘PA’ marker types as outlined above proved extremely helpful in detecting genomic rearrangements. Segments of the genetic map carrying three or more adjacent ‘het’ markers or ‘PA’ markers were validated by comparing read densities in the corresponding regions of the genomic sequence data. For single mapped ‘PA’ or ‘het’ markers, validation by resequencing data is very difficult. These markers were regarded with care, as their annotation may be false due to sequence similarity. Still, some examples in this study prove them to be useful rearrangement markers.

**Figure 1:**
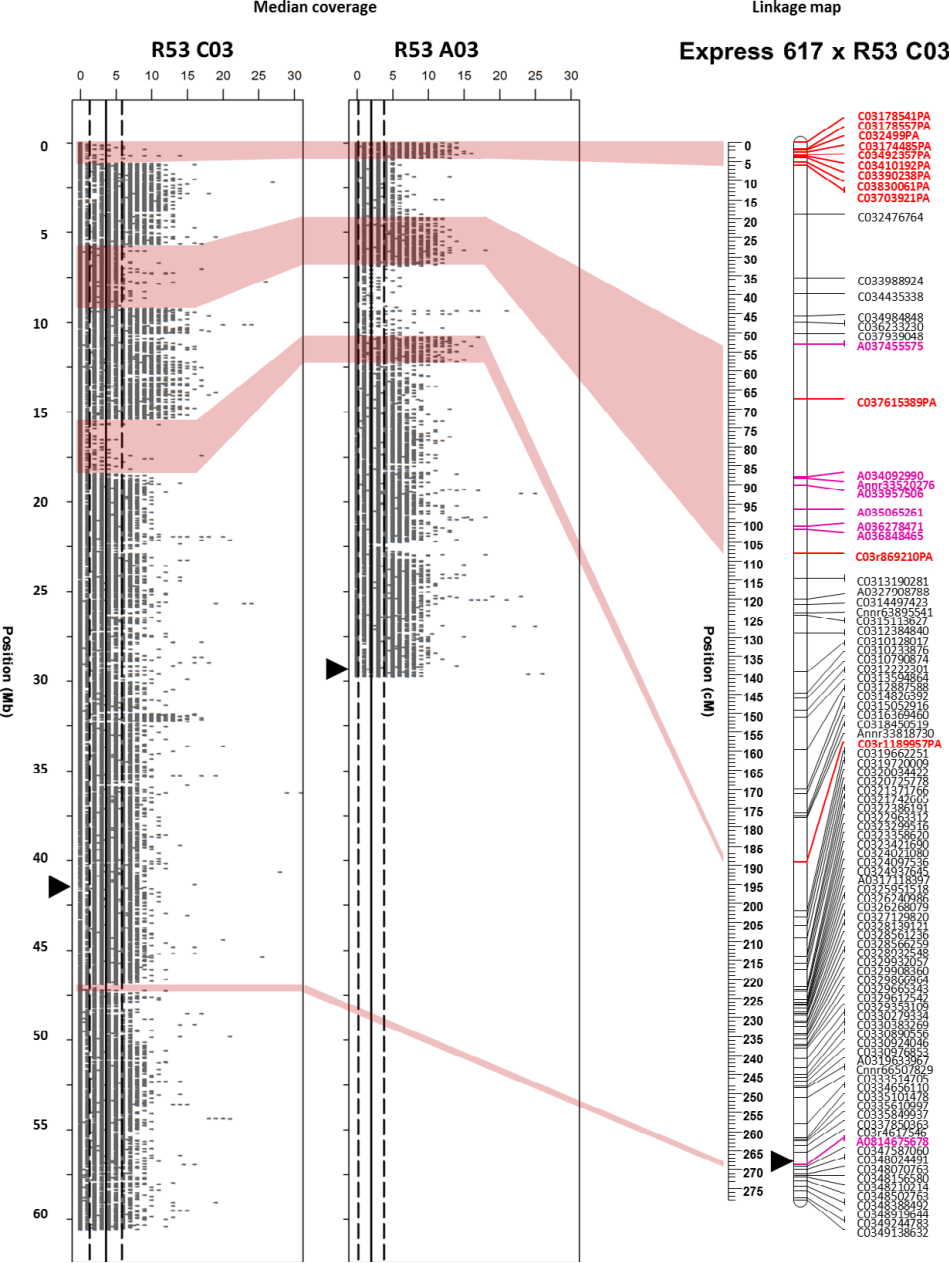
A03 to C03 translocation in the synthetic *B. napus* genotype R53 identified by resequencing and validated by genetic mapping. The plots show resequencing read coverage across the lengths of the respective chromosomes, calculated for segments of 1 kb. The genetic linkage maps on the right of the read maps show genetic mapping including SNPs with normally segregating, bi‐allelic calls with locus names in black text. SNPs called as deletions (presence–absence markers, with suffix ‘–PA’) are indicated by bold red marker names, whereas SNPs with heterozygous–homozygous segregation due to polymorphism in one of two duplicated copies (with suffix ‘–het’) are indicated by bold blue marker names. Polymorphic markers in bold magenta text indicate duplicated markers mapping to their homoeologous position. Opaque red blocks link putative deletions detected in coverage blocks with the corresponding regions in the genetic maps. Centromere regions are indicated by black triangles according to (Mason *et al*., 2013).

**Figure 2:**
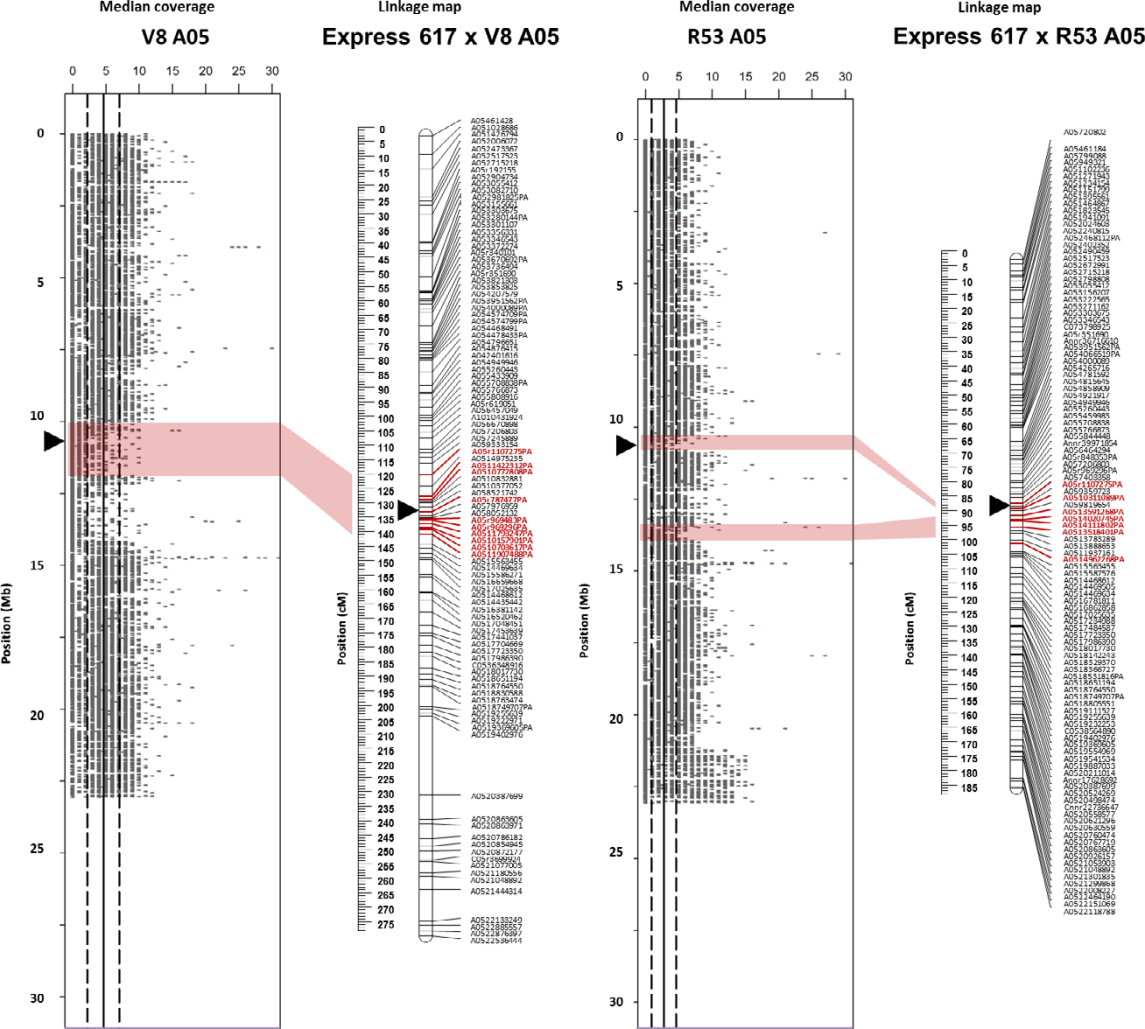
A05 Deletion in the synthetic *B. napus* genotypes V8 und R53 identified by resequencing and validated by genetic mapping. The plots show resequencing read coverage across the lengths of the respective chromosomes, calculated for segments of 1 kb. The genetic linkage maps on the right of the read plots show genetic mapping including SNPs with normally segregating, bi‐allelic calls with locus names in black text. SNPs called as deletions (presence–absence markers, with suffix ‘–PA’) are indicated by bold red marker names, whereas SNPs with heterozygous–homozygous segregation due to polymorphism in one of two duplicated copies (with suffix ‘–het’) are indicated by bold blue marker names. Polymorphic markers in bold magenta text indicate duplicated markers mapping to their homoeologous position. Opaque red blocks link putative deletions detected in coverage blocks with the corresponding regions in the genetic maps. Centromere regions are indicated by black triangles according to (Mason *et al*., 2013).

Two examples for independent validation of prominent HE and deletion events by plotting genomic resequencing coverage and genetic mapping in DH mapping populations are shown in Figures 1 and 2. In Figure 1, four homoeologous nonreciprocal translocations (HNRTs) were identified in linkage group C03 in population ExR53‐DH using genomic resequencing data; each could be correlated to some extent with SNP mapping data. The first of them was mapped by a block of C03‐PA‐markers spanning the region 0–6.1 cM on top of linkage group C03. This corresponds to a deletion in R53 chromosome C03 of 1.16 Mb. A putatively translocated homoeologous A03 duplicate fragment was not mapped. The second was a A03‐duplication and translocation to C03 mapped by a block of A03‐markers spanning the region 53–108.1 cM. This corresponds to the positions 3.9–6.8 Mb on chromosome A03 and 5.5–9.0 Mb on chromosome C03. The third was a C03‐deletion at the position 15.4–18.8 Mb, which was mapped by a single C03‐PA‐marker at 189.2 cM. The deletion corresponds to a duplication of an A03‐fragment. Although the genomic segments here are large in size, the corresponding SNP markers did not meet the expected 1:1 segregation ratio and could not be mapped. The forth HE event, a C03‐deletion at the position 47.9–48.8 Mb and a homoeologous A08‐duplication of 0.8 Mb, was mapped by a single A08‐marker at 268.7 cM.

In Figure 2, several smaller and larger segmental deletions detected by resequencing coverage analysis, spanning the same region of chromosome A05 in both V8 and R53, were genetically mapped by a block of PA‐markers in both the ExV8‐DH and ExR53‐DH genetic maps. The corresponding regions span 20.5 cM (1.750 Mb) in ExV8‐DH and 13.9 cM (4.651 Mb) in ExR53‐DH. Some PA‐markers are common between the two populations, whereas others are unique to one of the populations.

### QTL mapping

In this study, 15 QTL for 15 traits were identified in the Ex1012‐98‐DH population, 61 QTL for 37 traits in the ExV8‐DH population and 25 QTL for 14 traits in the ExR53‐DH population. All QTL share LOD scores higher than 5, allowing an alpha‐error not larger than 0.05. Table 1 gives a list of detected QTL in chromosome regions with putative genomic rearrangements for all measured traits in the three mapping populations. The list includes their genetic and inferred physical positions (unique BLAST hits) in the *B. napus* Darmor*‐bzh* reference genome, R² values indicating the proportion of phenotypic variation explained by the QTL, and the LOD scores at QTL peaks. The colocalization of QTL with regions involved in genomic rearrangements is consistent with the hypothesis that genomic rearrangements generate significant phenotypic variation with considerable selective and evolutionary potential.

**Table 1:** Major QTL for seed quality and agronomic traits located in genomic regions that are rearranged with respect to the *B. napus* Darmor‐*bzh* reference sequence. Calculation was performed by composite interval mapping, considering only QTL with a LOD score of >5; QTL in bold text could also be identified in genetic mapping

Population	Trait	Linkage group	Genetic position (Marker interval) [cM]	Inferred physical position [kb]	LOD score	*R*²	Structural rearrangement
ExV8‐DH	Seedling volume increase	A05	161–165	A05 17 065–17 605	8.0	0.14	Deletion
	**Seed sulphur**	**C03**	**222–227.5**	**C03 51 659–52 628**	**5.2**	**0.09**	**Deletion**
	Seed sulphur	C09	22.5–27.5	C09 2814–3592	29.7	0.42	Deletion
	Seed glucosinolate	C09	22.5–27.5	C09 2814–3592	35.0	0.48	Deletion
Ex1012‐98‐DH	**Seed ADL (acid detergent lignin)**	**A09**	**152**	**C08 33 479**	**28.9**	**0.56**	**HE**
	**Seed NDF (neutral detergent fibre)**	**A09**	**152**	**C08 33 479**	**31.6**	**0.59**	**HE**
	**Seed colour**	**A09**	**152**	**C08 33 479**	**7.7**	**0.20**	**HE**
	**Seed C18:3**	**A09**	**176–177**	**A09 31 557**	**8.4**	**0.21**	**HE**
	Days to flowering	A09	193–194.5	A09r 3826 (~A09 30 000)	7.4	0.20	HE
	**Flowering duration**	**A09**	**207.5–208**	**C08 36 510**	**6.3**	**0.18**	**HE**
	**Seeds per silique**	**A09**	**208**	**C08 36 510**	**5.1**	**0.15**	**HE**
ExR53‐DH	Seed ADL (acid detergent lignin)	A05	15–17.5	A05 1464	5.4	0.10	Deletion
	Seed ADF (acid detergent fibre)	C01	193.5–194	C01 34 145–38 105	8.6	0.16	Deletion
	Seed NDF (neutral detergent fibre)	C01	193.5–194	C01 34 145–38 105	10.4	0.19	Deletion

### A homoeologous rearrangement on A09 causes variation in seed fibre content

In a previous study involving the same plant material, we concluded that a major QTL for seed colour and seed coat fibre content, colocalizing on chromosome A09 in the mapping populations Ex1012‐98‐DH and ExV8‐DH, might have derived from an HE causing deletion or conversion of important candidate genes on chromosome A09 (Stein *et al*., 2013). Because of the comparatively low marker density in that study, it was not possible to precisely map the HE borders and correctly localize the physical position of the QTL. In this study, we confirmed the presence of this HE by sequence coverage and validated the genetic position using a considerably larger set of molecular markers than before.

**Figure 3:**
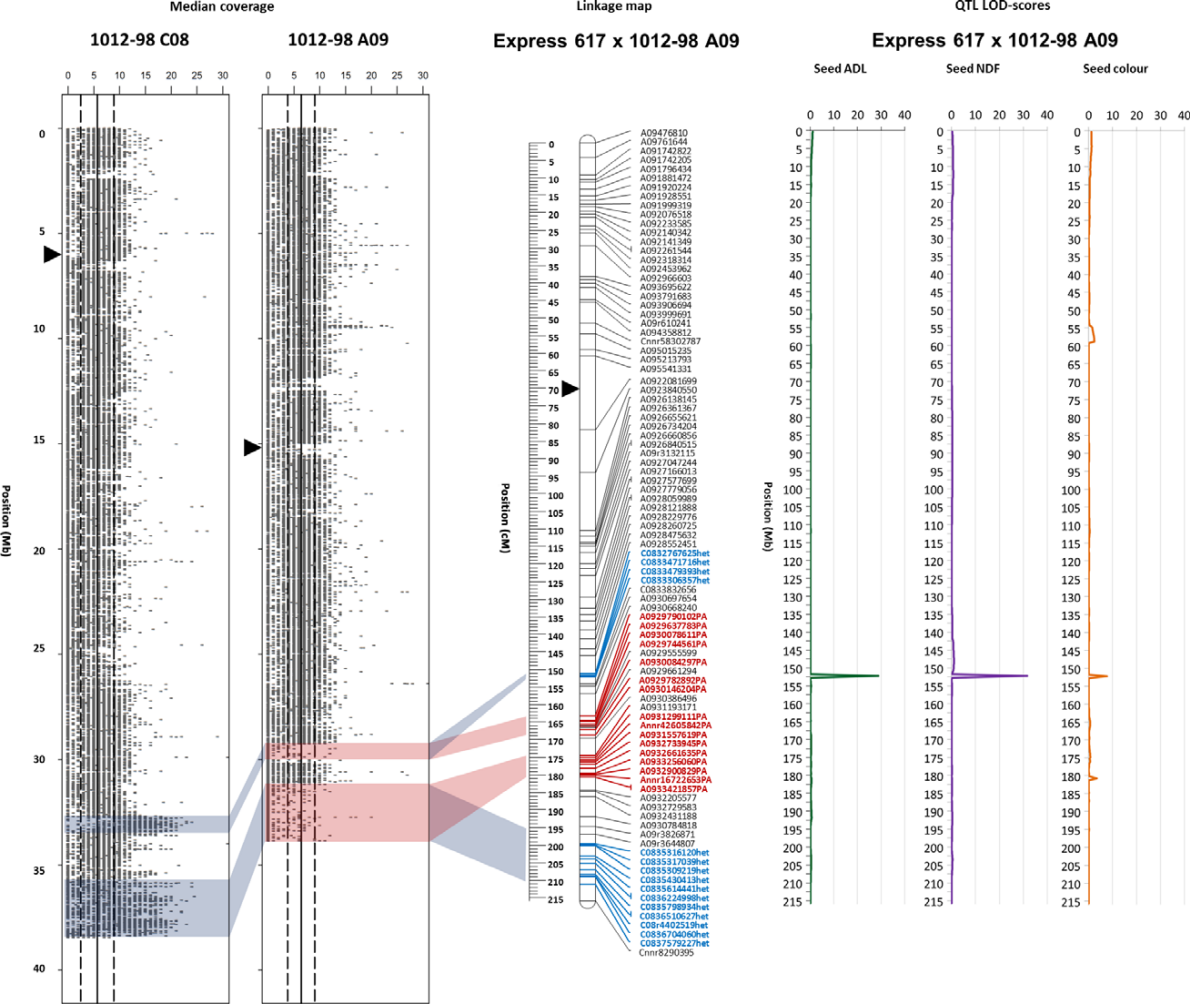
Localization of a major QTL on A09 influencing numerous seed quality traits in the mapping population Ex1012‐98‐DH, within a prominent HE between the distal ends of homoeologous chromosomes A09 and C08. The two plots on the left hand side show median read coverage across the lengths of the respective homoeologous chromosomes, calculated for segments of 1 kb. The two genetic linkage maps on the right show genetic mapping including SNPs with normally segregating, bi‐allelic calls with locus names in black text. SNPs called as deletions (presence–absence markers, with suffix ‘–PA’) are indicated by bold red marker names, whereas SNPs with heterozygous–homozygous segregation due to polymorphism in one of two duplicate copies (with suffix ‘–het’) are indicated by bold blue marker names. Opaque red blocks link putative deletions, detected based on sequence coverage blocks, with the corresponding regions in the genetic maps, while opaque blue blocks indicate putative duplications, respectively. Centromere regions are indicated by black triangles position according to (Mason *et al*., 2013).

The coverage plots of the homoeologous chromosomes of the synthetic parent 1012‐98 (Figure 3) clearly show coincidence of a segmental deletion in A09, corresponding to a duplication of the homoeologous segment in C08. SNP markers derived from chromosome C08 map as ‘het’ markers on linkage group A09, marking the position of a duplicated block. A09 markers representing a deletion (‘PA’) flank this block. Using the new, high‐density genetic map of Ex1012‐98‐DH chromosome A09, including the HE‐tracing markers, we narrowed the QTL to a very small interval with an extremely high LOD peak for the seed fibre QTL (Figure 3). The 173‐kb interval harbours one gene from the monolignol biosynthesis pathway, *BnaCAD2/3* (*BnaA09g42930*/*BnaC08g35540*). The whole translocation region includes also *BnaCCR1* (*BnaA09g56490*/*BnaC08g38580*) and *BnaAHA10* (*BnaA09g41670*/*BnaC08g34260*), confirming the previously hypothesized involvement of these genes in the seed fibre and seed colour QTL (Stein *et al*., 2013). Cinnamoyl‐CoA‐reductase (CCR1) confers reduction in *p*‐coumaroyl‐CoA to *p*‐coumaraldehyde, which is subsequently reduced to the monolignol *p*‐coumarylalcohol by cinnamyl alcohol dehydrogenase (CAD2/3). While CCR1 acts substrate‐specific, CAD2/3 has a potentially wider substrate spectrum, which allows a certain plasticity in monolignol metabolism (Bonawitz and Chapple, 2010). Impaired activity of either CCR1 or CAD2/3 does not necessarily reduce the lignin content in the plant organ, but can affect the lignin composition. Double knockout of both genes, however, has been shown to decrease lignin content (Chabannes *et al*., 2001). Autoinhibited H+‐ATPase isoform 10 (AHA10) is a seed‐expressed transcription factor involved in proanthocyanidin formation in the seed coat endothelium, which has been shown to influence seed coat pigment accumulation (Baxter *et al*., 2005). The physical annotation of these genes within a deleted segment of chromosome A09 in the genotype 1012‐98 likely explains the colocalization of QTL for both seed acid detergent lignin (ADL) and seed colour (Figure 3).

**Figure 4:**
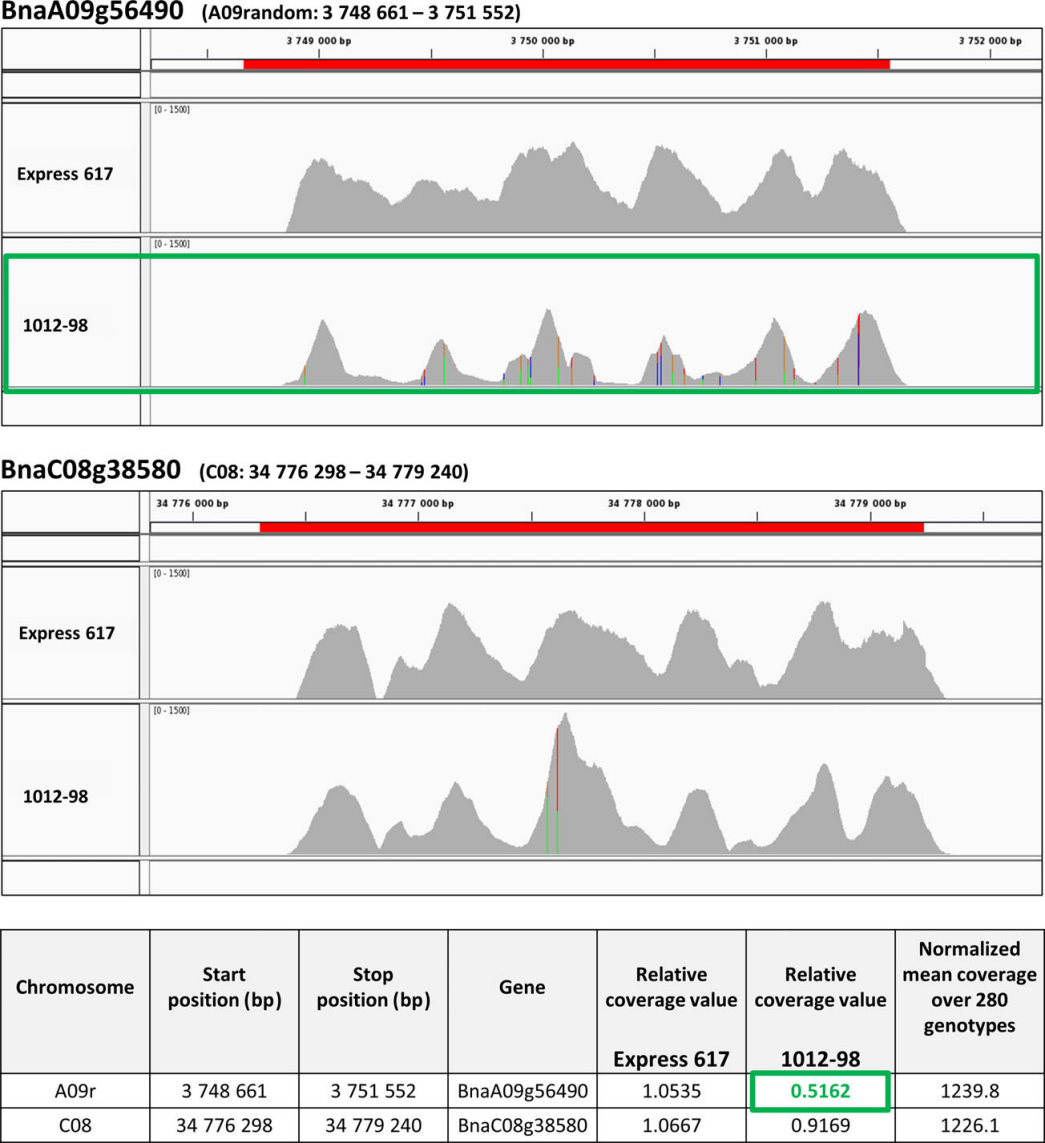
Mapping results from targeted sequence capture of the two *BnaCCR1* copies *BnaA09g56490* and *BnaC08g38580* in the *B. napus* genotypes Express 617 and 1012‐98. Sequence read coverages were mapped against the *B. napus* reference sequence Darmor‐bzh v. 4.1 and are displayed along the gene length. SNPs (indicated by coloured bars) in the homozygous doubled‐haploid genotype 1012‐98 suggest two variants of the *BnaC08g38580* gene copy. This may have also led to mis‐alignment of some reads to the homoeologous gene *BnaA09g56490*. Both the coverage landscape for *BnaA09g56490* in 1012‐98 and the according relative coverage values given in the table indicate a deletion of this gene copy (highlighted in green boxes). Relative coverage values were calculated as the ratio of the normalized mean coverage of the genotype over normalized mean coverage over 280 genotypes for each specific gene copy. Sequence‐capture data were obtained from (Schiessl *et al*., 2017).

A targeted sequence‐capture experiment carried out for *BnaCCR1* in the mapping parents Express 617 and 1012‐98 underpins the hypothesis that the large deletion described above has led to loss of an A‐genome copy of *BnaCCR1* in 1012‐98. Details of the experimental setup and data analysis are described by Schiessl *et al*. (2017). Figure 4 presents sequence‐capture results of the two *BnaCCR1* copies *BnaA09g56490* and *BnaC08g38580* at very high average sequencing depth (approximately 1200×), allowing highly accurate estimations of copy number. Coverage for *BnaA09g56490* is considerably lower and less consistent over the length of the gene copy in 1012‐98. In comparison with the normalized gene coverage across the whole experimental panel of 280 genotypes described by Schiessl *et al*. (2017), the gene coverage is reduced to 51%.

### PCR and BAC‐FISH validation of the QTL‐associated HE between chromosomes C08 and A09

We validated the deletion of a 900‐kb region (29 274–30 174 Mb) in 1012‐98 using three primer pairs specific to this region. As expected, no amplification was observed in 1012‐98, whereas the expected PCR product, indicating presence of the chromosome segment, was observed in Express 617, R53, V8 and Darmor*‐bzh*.

**Figure 5:**
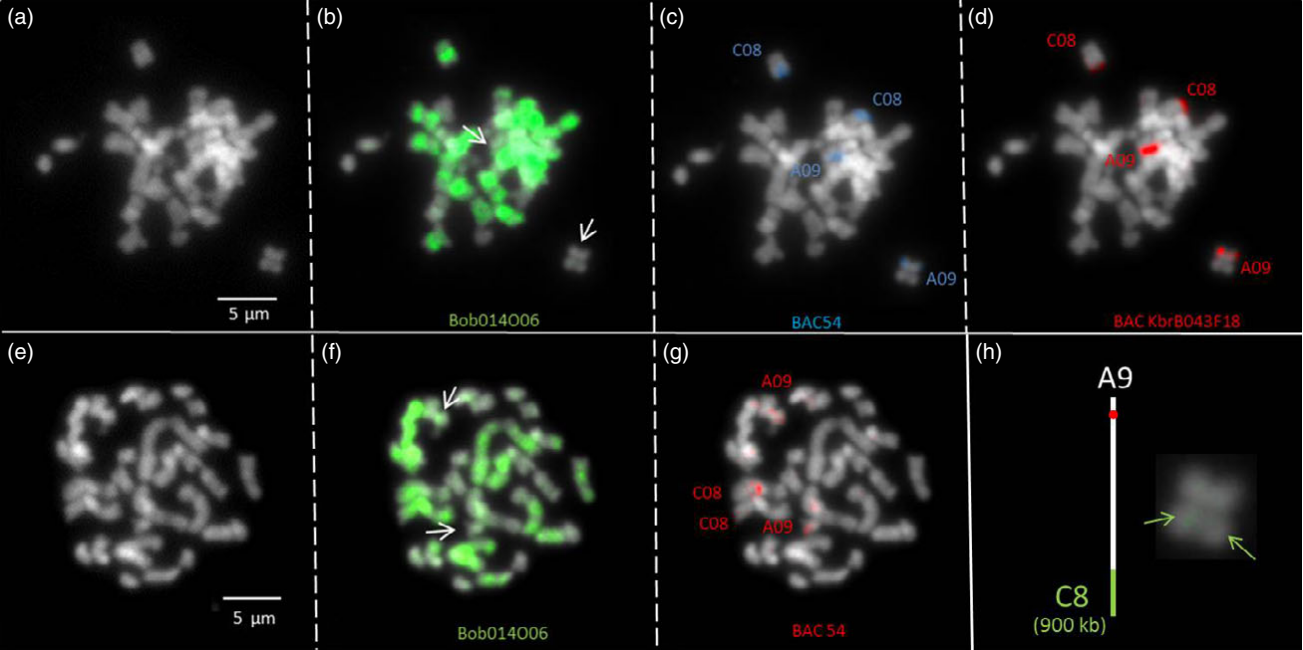
Fluorescence *in situ* hybridization using bacterial artificial chromosome probes to identify a putative HNRT between chromosomes A09 and C08 in the synthetic *B. napus* genotype 1012‐98. (a, e) DAPI background staining of somatic metaphase chromosomes of the synthetic *B. napus* genotype 1012‐98, carrying a putative HNRT between chromosomes A09 and C08. (b, f) Green FISH signals from BAC BoB014O06, which identifies all *Brassica* C‐genome chromosomes. (c) Blue FISH signals and red signals (g) from BAC54, specific for chromosomes A09 and C08. (d) Red FISH signals from KBrB043F18, also specific for chromosomes A09 and C08. Arrows indicate chromosomes with putative rearrangements. (h) Schematic representation of translocation of enlarged A09 chromatids. Green arrows indicate green FISH signal, representing the presence of a C‐subgenome fragment on chromosome A09.

The BAC KBrB043F18 and BoB014O06 were hybridized to identify the A09 and C08 chromosomes and all C‐genome chromosomes, respectively, to detect the homoeologous rearrangement (green) (Figure 5b and c). To further validate the A09 chromosome fragment losses, a BAC‐FISH experiment was performed using BAC clone 54 probe (isolated from Express 617) present in the rearranged region of 1012‐98. Due to the high‐sequence similarity between A and C genomes, two or four signals are expected in the case of a deletion or an HNRT, respectively. In 1012‐98, four signals were observed with BAC clone 54 (Figure 5c), indicating the presence of an HNRT. As the limit of GISH‐like resolution is 5 Mb to clearly observe rearranged chromosomes by harbouring a dual colour signal, it is not easy to observe smaller rearrangements by hybridization of the BoB014O06 BAC. However, in Figure 5b (and enlarged in Figure 5h), the C08‐to‐A09 translocation in 1012‐98 can be observed by a faint green signal in the otherwise unstained A09 chromosome.

## Discussion

Genomic rearrangements are widespread in the recent allopolyploid species *B. napus*, making it an interesting model for studying the cause and effect of *de novo* polyploidization. The presence of two additive genomes with a high degree of homoeology in the same nucleus leads to meiotic chromosome pairing between homoeologous chromosomes during the first generations after allopolyploidization (Szadkowski *et al*., 2010), causing considerable HE occurrence and gene conversion (Chalhoub *et al*., 2014). Implementation of novel variation caused by genomic rearrangement events, such as HE events or pure deletions in synthetic *B. napus,* is of considerable interest for rapeseed and canola breeders. However, skewed marker segregation patterns, which occur as consequence of genomic rearrangements, prevent standard mapping procedures from accurately localizing QTL, tightly linked markers and causal genes in these regions.

Here, we present a method which enabled us to accurately map agronomic QTL to a number of genomic rearrangement events. The 60K Illumina SNP array marker data were used to generate high‐density genetic maps in half‐sib DH populations from three synthetic *B. napus* accessions, carrying interesting disease resistance (Obermeier *et al*., 2013), agronomic and yield‐related traits (Basunanda *et al*., 2007, 2010; Radoev *et al*., 2008) and seed quality characters (Badani *et al*., 2006; Stein *et al*., 2013). Although the putative colocalization of HE events with QTL in *B. napus* has been suggested in previous studies (Chalhoub *et al*., 2014; Liu *et al*., 2012; Stein *et al*., 2013) to our knowledge, this is the first study providing independent validation of QTL‐HE colocalization. Alongside genetic mapping and evidence from genomic coverage sequencing, we provide cytological indications from BAC‐FISH that a chromosome fragment spanning an HE‐associated QTL is indeed involved in the suspected HE. This supports our use of SNP marker data from loci spanning deletions or duplications, which normally would be discarded in mapping procedures. The results provide an interesting reminder about the importance of structural chromosome variation in genome mapping and QTL analysis and suggest implementation of routine screening for presence–absence variation as a resource for breeding diversity.

### Using the 60K Infinium SNP array allows genetic mapping of deletions and translocations

Homoeologous translocations were first detected in mapping populations using codominant RFLP markers (Parkin *et al*., 1995; Sharpe *et al*., 1995), which also gave initial insights into the frequency and extent of HE events in natural and synthetic *B. napus* (Song *et al*., 1995). In contrast to RFLP assays, rapid and cost‐effective analysis with high‐density SNP arrays provides considerably higher resolution of genetic recombination in mapping populations. For analyses of homoeologous rearrangements, the ability to anchor SNP markers to a reference genome sequence and to compare patterns for multiple SNPs per homoeologous chromosome segment allows a more accurate and detailed assessment of genomewide HE events. Genetic mapping of homoeologous reciprocal translocations is more challenging unless duplicated markers can be assigned to a unique locus. SNPs with multiple physical BLAST positions were discarded. This may mean that markers from highly similar homoeologous regions are neglected in the mapping process.

### Applicability of HE mapping approaches

Our method to derive HE information from high‐density SNP array data in segregating mapping populations provides an opportunity to include structural genome variation in genetic maps and QTL studies. Revisiting historical QTL data sets using SNP array data analysed with this technique will give a more comprehensive picture of HE associations to QTL. We are currently developing thresholds and techniques to apply HE calling from SNPs in nonrelated populations for genomewide association studies. In multiparent mapping populations, for example those described by Snowdon *et al*. (2015) for nested‐association mapping in *B. napus*, we expect that capturing of HE variants will greatly increase the power to detect QTL in genome regions that were previously very difficult to accurately access by genetic mapping techniques.

Access to multiple genome assemblies per species, using new assembly methods that greatly improve genome coverage and accuracy, will further improve our ability to trace the impact of chromosome‐level structural variation on quantitative trait expression.

## Experimental procedures

### Plant material

Three half‐sib doubled‐haploid (DH) winter–oilseed rape populations were used in this study, developed from crosses of the black‐seeded inbred winter–oilseed rape line ‘Express 617’ to the synthetic *B. napus* lines 1012‐98 and R53 and to the semisynthetic line V8. The origins of the parental lines, along with the DH mapping populations Express 617 × 1012‐98 (Ex1012‐98‐DH, *n* = 164), Express 617 × R53 (ExR53‐DH, *n* = 248) and Express 617 × V8 (ExV8‐DH, *n* = 248), have been described previously in detail (Badani *et al*., 2006; Basunanda *et al*., 2007, 2010; Radoev *et al*., 2008).

### Detection of genomic rearrangements from genomic resequencing data

Genomic resequencing data were collected from the parental genotypes Express 617, 1012‐98, V8 and R53 using the Illumina MiSeq and HiSeq 2000 systems. The MiSeq system delivered 250‐bp paired‐end reads, while 100‐bp paired‐end reads were collected using the HiSeq system. All reads were aligned to the *B. napus* reference sequence version 4.1 (Chalhoub *et al*., 2014) using the short oligonucleotide alignment program (SOAP2 v2.21) (Li *et al*., 2008), and read depths were calculated using the command *genomecov* in the bedtools package v.2.20.1 for every nucleotide position genomewide. Median coverage over 1000‐bp blocks was calculated using an R script, and adjacent blocks with the same coverage value were aggregated to consecutive segments using a circular binary segmentation algorithm implemented in R package PSCBS (Bengtsson *et al*., 2010; Olshen *et al*., 2011). Adjacent segments over 50 kb in length and with the same mean coverage value were merged unless separated by gaps larger than 50 kb.

Mean read coverage and standard deviation were calculated for each individual and chromosome. Segments with a coverage value exceeding the chromosome mean by 1 standard deviation were defined as segmental duplications, while segments with a coverage value of 1 standard deviation lower than the chromosome mean were defined as deletions. Accordingly, segments not deviating by more than 1 standard deviation from the chromosome‐wide average were assumed to have ‘normal’ coverage. The script used is provided in Data S1.

### DNA extraction and high‐density genetic mapping including HE markers

Total genomic DNA was extracted from 200 mg leaf material of young leaves of mapping parents and DH lines, using the Qiagen BioSprint 96 DNA Plant Kit (Qiagen GmbH, Hilden, Germany). All samples were subjected to high‐density, genomewide SNP genotyping using the Brassica Illumina 60K SNP genotyping array (Clarke *et al*., 2016). Genotyping was outsourced to TraitGenetics GmbH (Gatersleben, Germany).

Genomic positions in the *B. napus* reference genome sequence were assigned to all tested SNP markers showing a BLAST hit with the SNP flanking sequences, as described previously (Qian *et al*., 2014; Mason and Snowdon, 2016). In brief, SNP markers were physically localized on the *Brassica napus* Darmor*‐bzh* reference genome sequence assembly (version 4.1) (Chalhoub *et al*., 2014) using the following criteria: minimum overlap of 50‐bp length, minimum identity of 95%, no sequence gaps. SNPs with only one BLAST hit were regarded as informative in terms of physical position, and only these markers were included in the genetic mapping. Linkage mapping was conducted using the software Joinmap 4 (van Ooijen, 2006), after assignment of cosegregating markers into bins with Perl to reduce the locus number. Markers were subsequently scored according to three different genotype patterns (Mason *et al*., 2017): (i) polymorphic simple SNP calls; (ii) polymorphic hemi‐SNP calls; and (iii) polymorphic presence–absence SNP calls. The latter two allow recording of markers potentially affected by genomic rearrangement events. All of these scoring types were required to fit the expected 1:1 segregation for DH populations.

All SNP markers that were included in the mapping file were named with their predetermined physical position and a suffix indicating markers showing dominant presence–absence segregation (‘PA’) on the one hand, or codominantly segregating heterozygous hemi‐SNPs (‘het’) on the other hand. The map was calculated using the maximum‐likelihood algorithm and groups were formed with a cut‐off at recombination frequency 0.2. Maps were subsequently joined using Mapchart 2.3 to link bins containing SNP markers shared across the three maps.

### QTL mapping

Quantitative trait loci were calculated using Qgene4.3.10 (Joehanes and Nelson, 2008) based on the calculated genetic maps. Seed trait phenotyping for all populations was performed by near‐infrared reflectance spectrometry (NIRS) analysis on seed samples produced in field trials at four different locations in Germany, over multiple years from 2003 until 2015, following the seed analysis procedures (Wittkop *et al*., 2012). The seed colour and quality data from the first 2 years of experiments correspond with those reported by Badani *et al*. (2006). A fully randomized complete block design was used, with plots sizes of 10–13 m^2^ depending on the standard practice at each location. A high number of locations and years were preferred rather than multiple replications of genotypes per location, as is standard practice when testing large rapeseed populations, because the large plot size reduces field homogeneity when too many test plots are included per location. Selfed seeds from 3 to 5 representative plants per line were hand‐harvested at maturity and subjected to NIRS as above, with two technical replicates per sample. Germination traits in the ExV8‐DH population were phenotyped at the French national seed testing laboratory (GEVES, Angers, France), with 100 seeds per genotype and repetition with seed lots from two different production environments (Hatzig *et al*., 2015). Morphological traits in the ExV8‐DH population were collected from two locations in Germany in the years 2006 and 2007.

### HE validation using specific primers and BAC‐FISH

To validate the deletion of a 900‐kb fragment on A09 (29 274–30 174 Mb) in 1012‐98, we extracted this 900‐kb region from *B. napus* ‘Darmor‐*bzh*’ genome (Chalhoub *et al*., 2014) and blasted it against the whole ‘Darmor‐*bzh*’ genome sequence, enabling us to identify fragments of at least 500 bp that were specific to this A09 chromosome region (i.e. absent from the homoeologous region). We then designed primer pairs from these specific A09 regions using Primer 3.0 (Rozen and Skaletsky, 2000). Only the primers (3 pairs) that gave a single band in different *B. napus* varieties tested and no amplification in *B. oleracea* were retained (primer details are given in Table S3). The PCR products obtained using *B. napus* ‘Darmor’ DNA were also directly sequenced, enabling further validation of the specificity of these primer pairs to A09. Subsequently, these primer pairs were tested using DNA from synthetic line 1012‐98. Each PCR amplification was performed in a total volume of 50 μL containing 10 μL of 5× buffer (Promega), 4 μL of 25 mm MgCl2, 0.5 μL of 25 mm dNTP mix, 2.5 μL of each primer (10 mm), 0.2 μL of GoTaq^®^ G2 Hot Start polymerase (5 U/μL) and 50 ng of DNA. For PCRs, genomic DNA was denatured at 94 °C for 2 min, followed by 30 cycles of 94 °C for 30 s, 58 °C for 30 s and 72 °C for 1 min.

The proximal 0.5–1.5 cm of young seedling roots were excised, treated in the dark with 0.04% 8‐hydroxyquinoline for 2 h at 4 °C and then transferred to room temperature for 2 h to accumulate cells at metaphase. Root tips were then fixed in 3:1 ethanol–glacial acetic acid for 48 h at 4 °C and stored in 70% ethanol at −20 °C until required. After washing in 0.01 m citric acid–sodium citrate (pH 4.5) for 15 min, the tips were digested in 5% Onozuka R‐10 cellulase (Sigma‐Aldrich, St. Louis, MO) containing 1% Y23 pectolyase (Sigma) at 37 °C for 30 min and then washed carefully with distilled water for 30 min. Single root tips were transferred to cleaned microscope slides and macerated with a drop of 3:1 fixation solution using a preparation needle. After air‐drying, slides with good metaphase chromosome spreads were stored at −20 °C until further use.

The *B. napus* BAC clone 54 from the parental genotype Express 617 was used to probe a chromosome spread of synthetic *B. napus* parental genotype 1012‐98, in which a putative HE was suspected between homoeologous chromosomes C08 and A09. The BAC clone was previously identified by PCR screens with markers from a QTL region on chromosome A09 (see below). Sanger sequencing confirmed alignment of the BAC clone to *B. napus* Darmor*‐bzh* chromosome A09.

BAC clone 54 was labelled by random priming with biotin‐14‐dUTP (Invitrogen, Life Technologies Waltham, MA). The BAC clones KBrB043F18 (from *B. rapa* chromosome A09, homoeologous to *B. oleracea* chromosome C08 (Xiong and Pires, 2011) and *B. oleracea* BoB014O06 (Howell *et al*. 2002) were labelled by random priming with Alexa 594‐5‐dUTP and Alexa 488‐5‐dUTP, respectively. BoB014O06 was used as genomic *in situ* hybridization (GISH)‐like probe to specifically stain all C‐genome chromosomes in *B. napus* (Suay *et al*., 2014; Szadkowski *et al*., 2010).

Chromosome preparations were incubated in RNAse A (100 ng/μL) and pepsin (100 μg/mL) in 0.01 m HCl and fixed with paraformaldehyde (4%). Chromosomes were denatured in a solution of 70% formamide in 2× saline‐sodium citrate buffer (SSC) at 70 °C for 2 min, dehydrated in an ethanol series (70%, 90% and 100%) and air‐dried. The hybridization mixture consisted of 50% deionized formamide, 10% dextran sulphate, 2xSSC and 1% SDS. Labelled probes (200 ng per slide) were denatured at 92 °C for 6 min and transferred to ice. The denatured probe was placed on the slide, and *in situ* hybridization was carried out overnight in a moist chamber at 37 °C. After hybridization, slides were washed for 5 min in 50% formamide in 2xSSC at 42 °C, followed by several washes in 4xSSC‐Tween to remove nonconjugated probe. Biotinylated probe was immuno‐detected by Texas Red Avidin DCS (Vector Laboratories, Burlingame, CA), and the signal was amplified with biotinylated anti‐avidin D (Vector Laboratories). The chromosomes were mounted and counterstained in Vectashield (Vector Laboratories) containing 2.5 μg/mL 4′,6‐diamidino‐2‐phenylindole (DAPI). Fluorescence images were captured using a CoolSnap HQ camera (Photometrics, Tucson, AZ) on an Axioplan 2 microscope (Zeiss, Oberkochen, Germany) and analysed using MetaVueTM (Universal Imaging Corporation, Downingtown, PA).

## Supporting information


**Figure S1** Genome‐wide coverage segmentation derived from resequencing data from four mapping parents across the 19 chromosomes of the *B. napus* A and C subgenomes.

**Figure S2** Resequencing read coverage plots of genotype Express 617.

**Figure S3** Resequencing read coverage plots of genotype 1012‐98.

**Figure S4** Resequencing read coverage plots of genotype R53.

**Figure S5** Resequencing read coverage plots of genotype V8.

**Figure S6** Genetic linkage maps of three half‐sib DH populations.

**Table S1** Coverage segments of equal copy number.

**Table S2** Map texts for genetic maps of three half‐sib DH populations.

**Table S3** List of primer pairs designed to validate a 900 kb deletion on the A09 chromosome in the synthetic *B. napus* line 1012‐98.

**Data S1** Code for calculation of genomic coverage and detection of duplication and deletion events.
