# Supplementary material for: Mapping of homoeologous chromosome exchanges influencing quantitative trait variation in Brassica napus
Source: Plant Biotechnol J. 2017 Apr 27;15(11):1478–89. doi: 10.1111/pbi.12732 (PMC5633767; doi:10.1111/pbi.12732)
Supplement: Supplementary file 1 — Figure S1 Genome‐wide coverage segmentation derived from resequencing data from four mapping parents across the 19 chromosomes of the B. napus A and C subgenomes. [file PBI-15-1478-s005.pptx]

## Slide 1
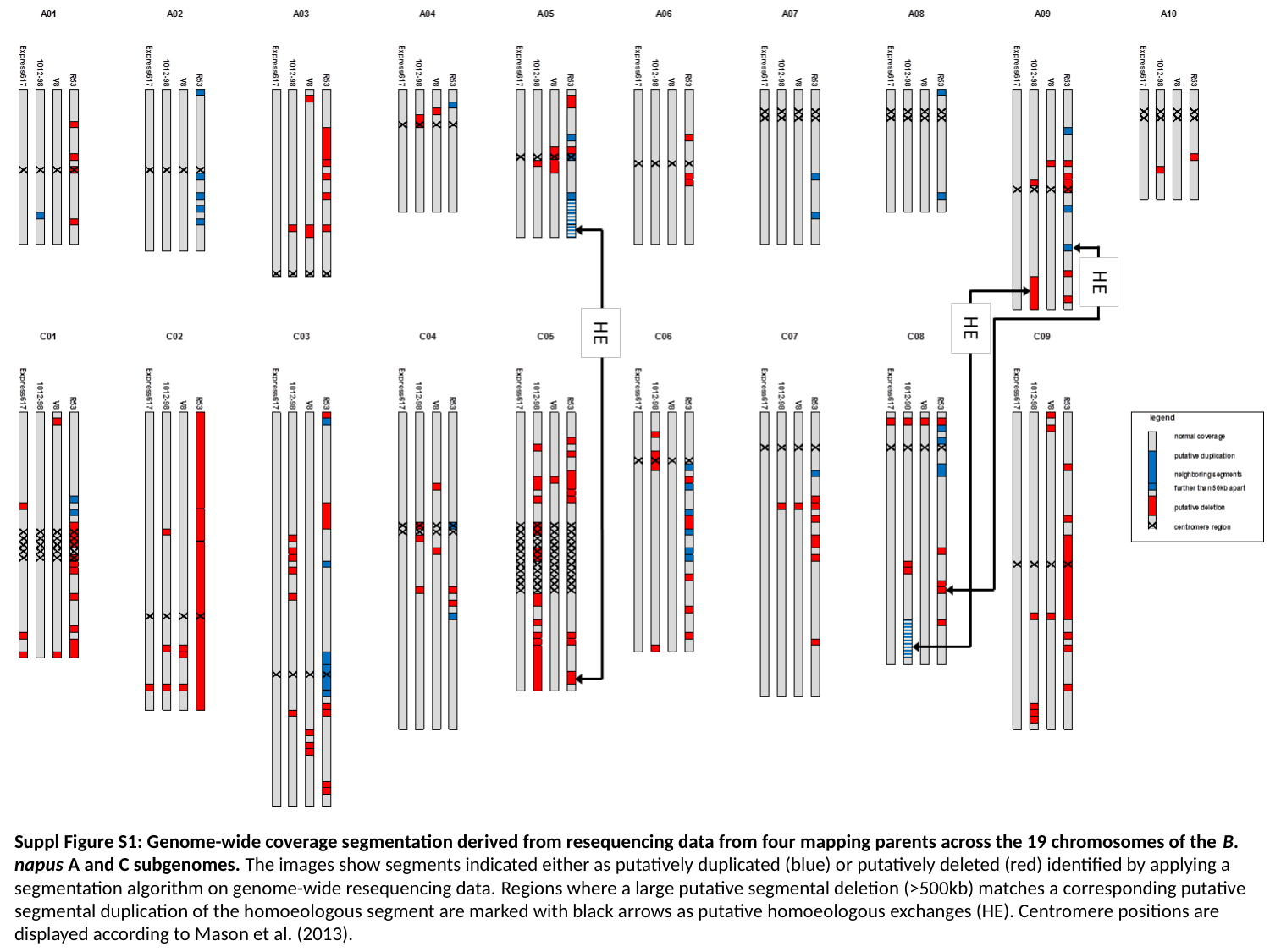

Suppl Figure S1: Genome-wide coverage segmentation derived from resequencing data from four mapping parents across the 19 chromosomes of the B. napus A and C subgenomes. The images show segments indicated either as putatively duplicated (blue) or putatively deleted (red) identified by applying a segmentation algorithm on genome-wide resequencing data. Regions where a large putative segmental deletion (>500kb) matches a corresponding putative segmental duplication of the homoeologous segment are marked with black arrows as putative homoeologous exchanges (HE). Centromere positions are displayed according to Mason et al. (2013).
