# Supplementary figures and images for: Mapping of homoeologous chromosome exchanges influencing quantitative trait variation in Brassica napus
Source: Plant Biotechnol J. 2017 Apr 27;15(11):1478–89. doi: 10.1111/pbi.12732 (PMC5633767; doi:10.1111/pbi.12732)

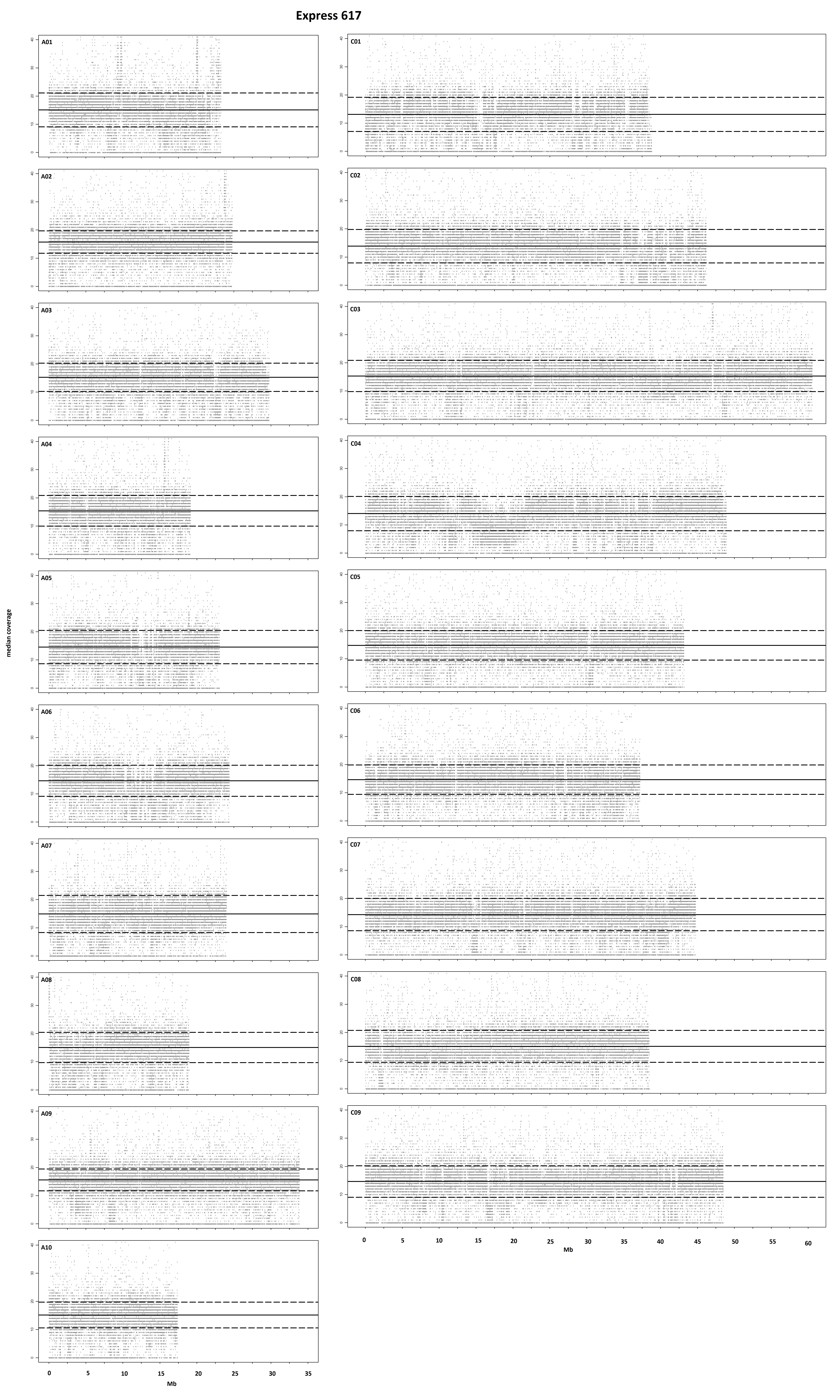

Supplement: Supplementary file 2 — Figure S2 Resequencing read coverage plots of genotype Express 617. [file PBI-15-1478-s008.tif]

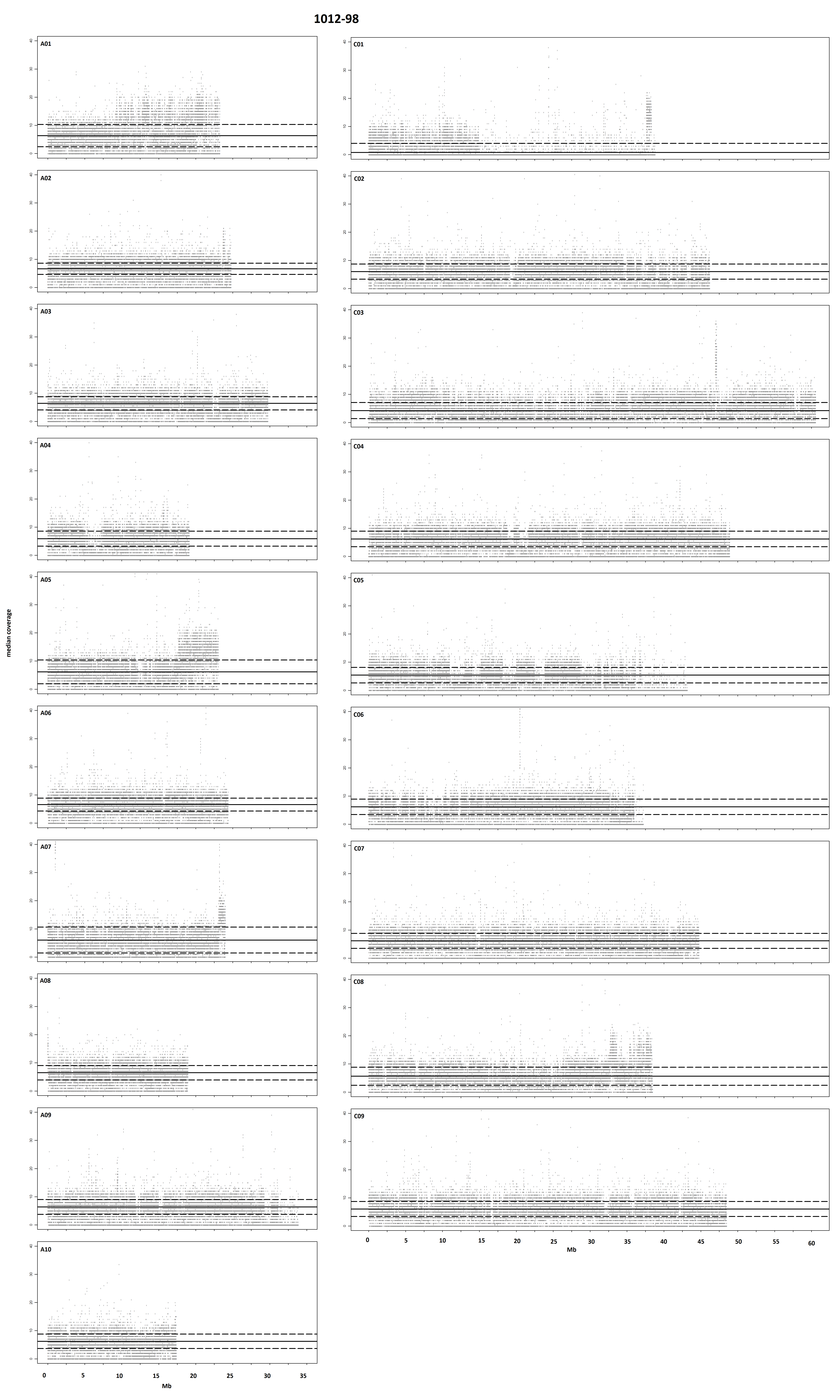

Supplement: Supplementary file 3 — Figure S3 Resequencing read coverage plots of genotype 1012‐98. [file PBI-15-1478-s007.tif]

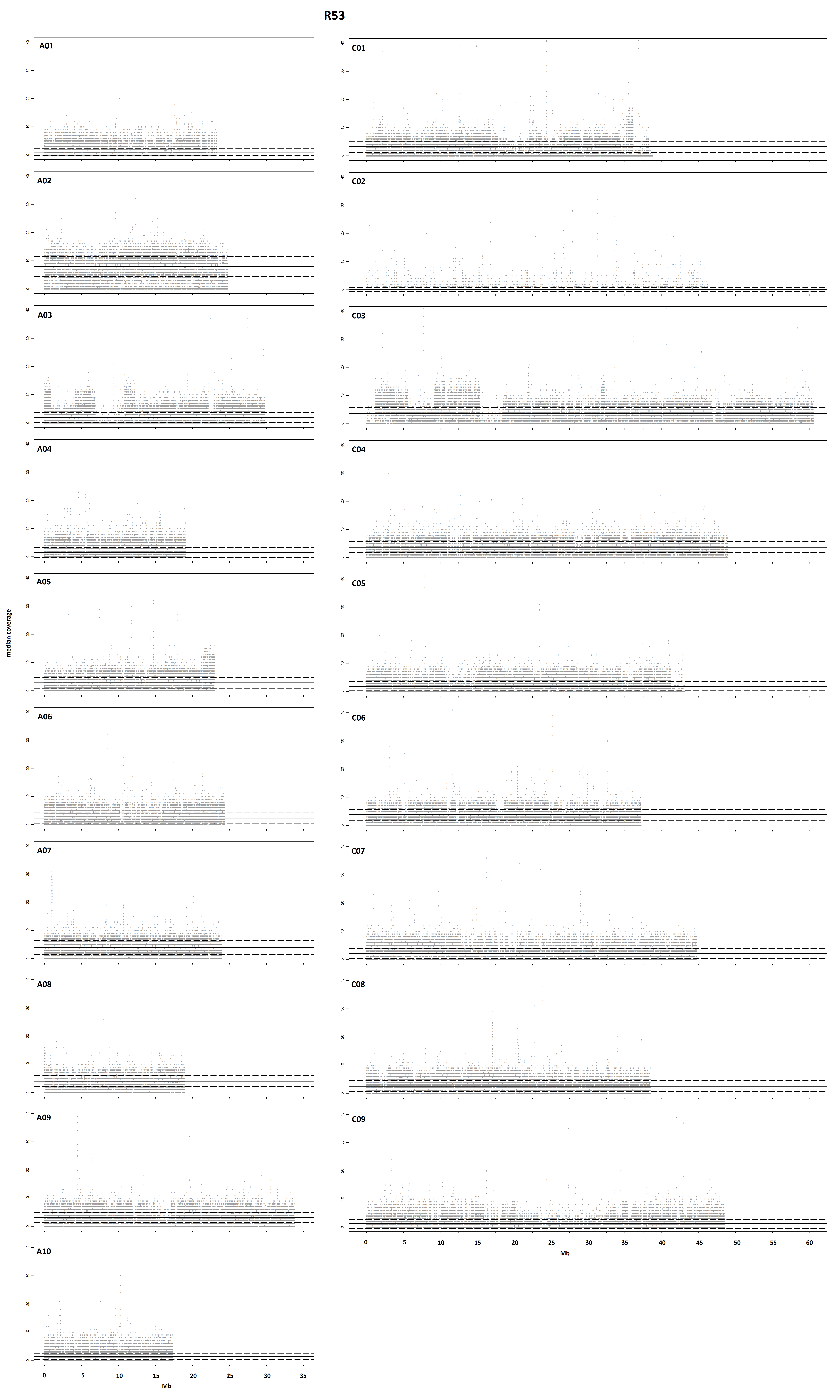

Supplement: Supplementary file 4 — Figure S4 Resequencing read coverage plots of genotype R53. [file PBI-15-1478-s009.tif]

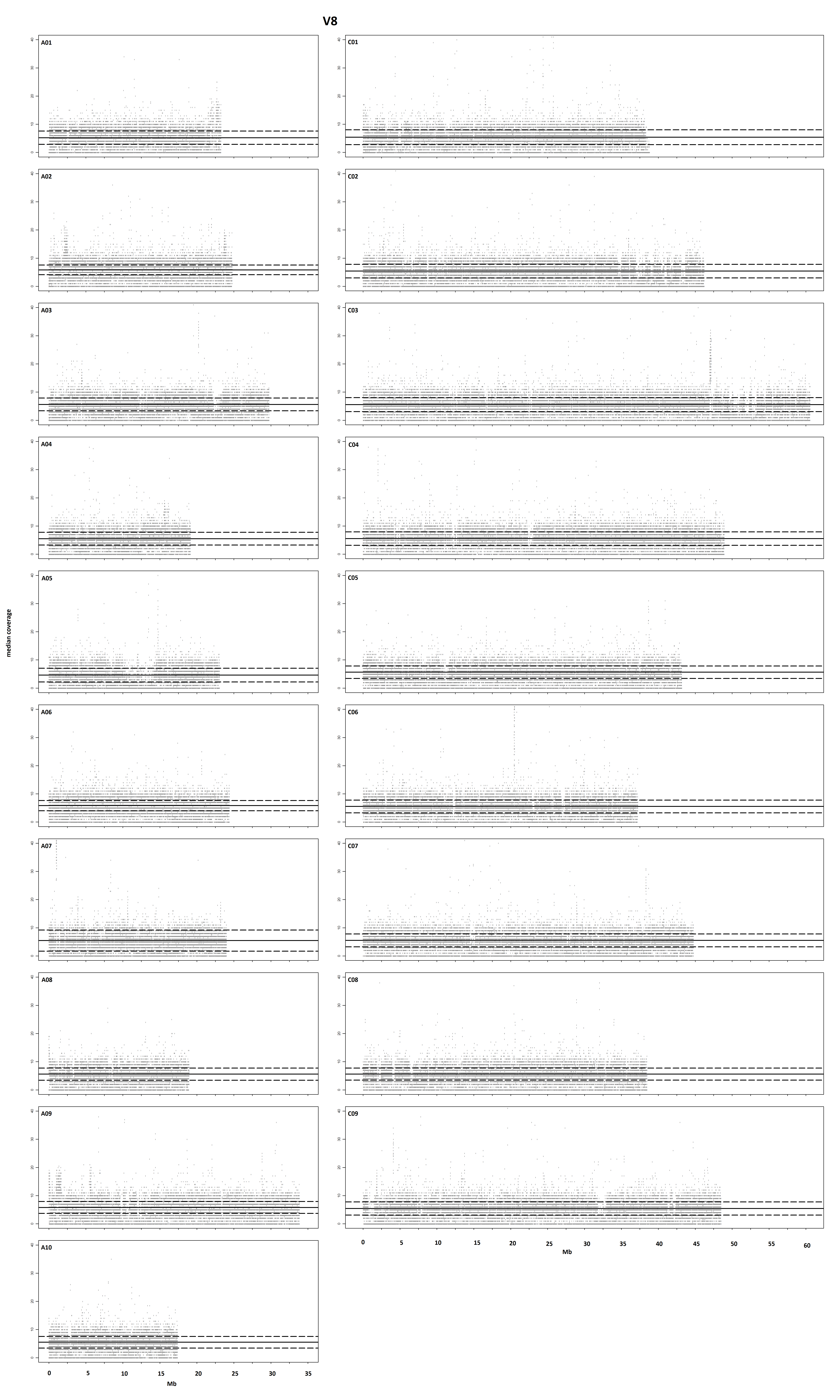

Supplement: Supplementary file 5 — Figure S5 Resequencing read coverage plots of genotype V8. [file PBI-15-1478-s001.tif]
