# Supplementary material for: Mapping of homoeologous chromosome exchanges influencing quantitative trait variation in Brassica napus
Source: Plant Biotechnol J. 2017 Apr 27;15(11):1478–89. doi: 10.1111/pbi.12732 (PMC5633767; doi:10.1111/pbi.12732)
Supplement: Supplementary file 6 — Figure S6 Genetic linkage maps of three half‐sib DH populations. [file PBI-15-1478-s002.pptx]

## Slide 1
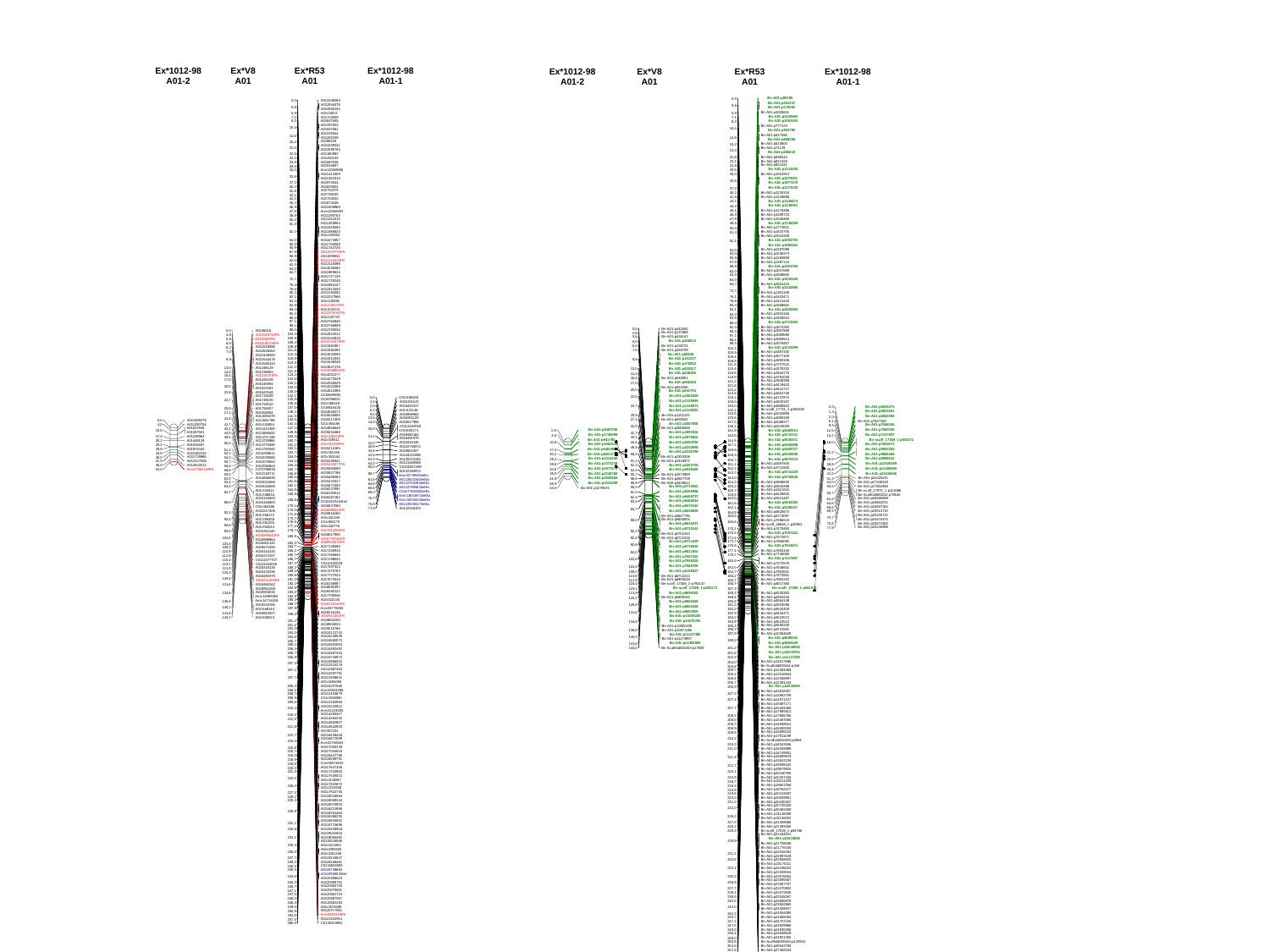

Ex*1012-98
A01-2
Bn-A01-p2067006
0,0
Bn-A01-p1745099
4,0
Bn-A01-p941792
10,8
Bn-A01-p1062520
Bn-A01-p648196
17,4
Bn-A01-p820317
20,2
Bn-A01-p1314440
29,2
Bn-A01-p1373239
29,8
Bn-A01-p1977270
34,6
Bn-A01-p2245780
38,0
Bn-A01-p2825565
41,9
Bn-A01-p3233299
48,9
Bn-A01-p3279678
53,0
Ex*V8
A01
Bn-A01-p442492
0,0
Bn-A01-p137869
4,8
Bn-A01-p436141
5,6
Bn-A01-p206610
6,0
Bn-A01-p138731
6,2
Bn-A01-p250759
7,0
Bn-A01-p80385
Bn-A01-p161237
8,9
Bn-A01-p176052
Bn-A01-p820317
13,0
Bn-A01-p648196
14,4
Bn-A01-p645951
16,4
Bn-A01-p688355
17,0
Bn-A01-p923356
20,5
Bn-A01-p941792
Bn-A01-p1062520
22,6
Bn-A01-p1125565
Bn-A01-p1145674
24,7
Bn-A01-p1149351
Bn-A01-p1152123
25,9
Bn-A01-p939202
27,1
Bn-A01-p2067006
41,0
Bn-A01-p2069488
Bn-A01-p1993302
41,7
Bn-A01-p1879401
43,1
Bn-A01-p2094793
44,5
Bn-A01-p2242890
49,4
Bn-A01-p2245780
50,2
Bn-A01-p2303308
Bn-A01-p2318972
51,1
Bn-A01-p2453758
52,1
Bn-A01-p2538180
54,2
Bn-A01-p2574969
55,7
Bn-A01-p2587709
56,6
Bn-A05-p3429612
58,8
Bn-A01-p2713583
59,3
Bn-A01-p5265598
81,5
Bn-A01-p5469727
82,4
Bn-A01-p5480514
83,0
Bn-A01-p5570110
84,7
Bn-A01-p5536695
Bn-A01-p5647795
Bn-A01-p5658004
88,0
Bn-A01-p5635471
Bn-A01-p5715141
92,2
Bn-A01-p5752342
Bn-A01-p5732226
92,4
Bn-A01-p5731429
92,6
Bn-A01-p5730640
Bn-A01-p5821651
94,0
Bn-A01-p7557422
102,6
Bn-A01-p7558345
Bn-A01-p7560785
103,4
Bn-A01-p6188347
108,2
Bn-A01-p6713213
110,8
Bn-A01-p6959248
112,9
Bn-scaff_17369_1-p794147
116,4
Bn-scaff_17369_1-p841371
119,1
Bn-A01-p9699152
121,9
Bn-A01-p9499100
125,3
Bn-A01-p9866468
129,4
Bn-A01-p9884449
Bn-A01-p9853365
131,6
Bn-A01-p10249225
Bn-A01-p10278752
134,8
Bn-A01-p10000230
Bn-A01-p10071456
136,8
Bn-A01-p11137395
140,2
Bn-A01-p11174997
Bn-A01-p11492050
141,6
Bn-Scaffold000253-p27839
143,2
Ex*R53
A01
Bn-A01-p80385
0,0
Bn-A01-p161237
0,8
Bn-A01-p176052
Bn-A01-p1026631
5,9
Bn-A01-p1125565
7,1
Bn-A01-p1062520
8,2
Bn-A01-p777123
10,4
Bn-A01-p941792
Bn-A01-p637586
14,6
Bn-A01-p688355
Bn-A01-p515600
15,2
Bn-A01-p71129
21,0
Bn-A01-p206610
Bn-A01-p598242
22,6
Bn-A01-p821328
23,1
Bn-A01-p951341
23,8
Bn-A01-p1314440
29,5
Bn-A01-p1551913
30,0
Bn-A01-p1879401
33,6
Bn-A01-p1977270
Bn-A01-p1373239
37,2
Bn-A01-p1235334
40,2
Bn-A01-p1149698
41,8
Bn-A01-p1145674
43,1
Bn-A01-p1149351
44,2
Bn-A01-p1276486
45,1
Bn-A01-p1409723
46,9
Bn-A01-p1548460
47,8
Bn-A01-p1745099
48,8
Bn-A01-p1772621
50,4
Bn-A01-p1823705
51,3
Bn-A01-p2042209
Bn-A01-p2094793
52,1
Bn-A01-p1993302
Bn-A01-p2187099
54,0
Bn-A01-p2246373
55,0
Bn-A01-p2260859
55,9
Bn-A01-p2387124
57,8
Bn-A01-p2453758
58,9
Bn-A01-p2637699
62,0
Bn-A01-p2608660
63,3
Bn-A01-p2538180
64,0
Bn-A01-p2501423
64,7
Bn-A01-p2242890
72,7
Bn-A01-p2251106
Bn-A01-p2403471
76,1
Bn-A01-p2421445
76,6
Bn-A01-p2688662
80,3
Bn-A01-p2825565
82,1
Bn-A01-p2934166
83,3
Bn-A01-p2928824
83,9
Bn-A01-p2713583
88,0
Bn-A01-p2874393
92,3
Bn-A01-p2957869
95,5
Bn-A01-p3059596
97,1
Bn-A01-p3050613
98,1
Bn-A01-p3078457
98,5
Bn-A01-p3233299
103,3
Bn-A01-p3487320
105,9
Bn-A01-p4077105
109,4
Bn-A01-p3859106
109,9
Bn-A01-p3737522
111,8
Bn-A01-p3378332
115,4
Bn-A01-p3644770
118,8
Bn-A01-p3791028
119,9
Bn-A01-p3948389
121,2
Bn-A01-p4419443
121,8
Bn-A01-p4641747
124,2
Bn-A01-p4651746
124,6
Bn-A01-p4747874
126,1
Bn-A01-p4820187
126,5
Bn-A01-p4908924
130,0
Bn-scaff_17731_1-p385358
132,1
Bn-A01-p5020890
133,8
Bn-A01-p4985329
135,6
Bn-A01-p5089377
137,5
Bn-A01-p5243029
138,1
Bn-A01-p5480514
141,9
Bn-A01-p5715141
143,5
Bn-A01-p5635471
144,4
Bn-A01-p5265598
147,1
Bn-A01-p5469727
149,8
Bn-A01-p5536695
150,3
Bn-A01-p5570110
150,7
Bn-A01-p5567840
151,1
Bn-A01-p5711920
152,1
Bn-A01-p5731429
153,7
Bn-A01-p5730640
154,0
Bn-A01-p5806635
154,2
Bn-A01-p5944398
155,2
Bn-A01-p6111016
155,7
Bn-A01-p6439605
156,8
Bn-A01-p5921497
160,5
Bn-A01-p5943392
161,6
Bn-A01-p6188347
162,1
Bn-A01-p6825673
164,9
Bn-A01-p6774097
165,8
Bn-A01-p7086543
168,8
Bn-scaff_26846_1-p30953
Bn-A01-p7479483
170,1
Bn-A01-p7557422
170,5
Bn-A01-p7573871
171,6
Bn-A01-p7850092
173,7
Bn-A01-p7834871
176,8
Bn-A01-p7852440
177,4
Bn-A01-p7745080
178,7
Bn-A01-p7137897
180,8
Bn-A01-p7372570
Bn-A01-p7646601
182,0
Bn-A01-p7953001
184,7
Bn-A01-p7974551
185,2
Bn-A01-p7982220
185,7
Bn-A01-p8017380
186,7
Bn-scaff_17369_1-p841371
187,3
Bn-A01-p8240363
188,3
Bn-A01-p8353426
189,4
Bn-A01-p8564149
190,8
Bn-A01-p9203096
191,3
Bn-A01-p9520409
192,2
Bn-A01-p9442471
192,9
Bn-A01-p9523572
193,2
Bn-A01-p8543512
194,9
Bn-A01-p9180340
195,3
Bn-A01-p9712591
196,7
Bn-A01-p10354645
197,9
Bn-A01-p9699152
199,2
Bn-A01-p9884449
Bn-A01-p10249225
201,2
Bn-A01-p10278752
201,6
Bn-A01-p11137395
203,3
Bn-A01-p11817698
204,0
Bn-Scaffold000444-p344
204,8
Bn-A01-p12484368
205,7
Bn-A01-p12516944
206,1
Bn-A01-p12308897
206,4
Bn-A01-p12391243
206,7
Bn-A01-p12535269
206,9
Bn-A01-p12618357
207,3
Bn-A01-p14802709
Bn-A01-p14071237
207,4
Bn-A01-p15087171
Bn-A01-p15494260
207,7
Bn-A01-p17885812
Bn-A01-p17906765
208,3
Bn-A01-p15487080
208,5
Bn-A01-p14948614
208,7
Bn-A01-p15000034
208,9
Bn-A01-p15695152
209,6
Bn-A01-p17911199
210,2
Bn-Scaffold000300-p4564
Bn-A01-p16343556
210,3
Bn-A01-p16358989
211,0
Bn-A01-p16749951
Bn-A01-p16692819
211,8
Bn-A01-p13622238
Bn-A01-p19598162
212,7
Bn-A01-p19879925
215,1
Bn-A01-p20240780
Bn-A01-p20207440
215,9
Bn-A01-p20211329
216,7
Bn-A01-p19607256
218,3
Bn-A01-p19762477
218,9
Bn-A01-p20133687
219,8
Bn-A01-p20839901
220,5
Bn-A01-p20430407
221,2
Bn-A01-p20745330
222,0
Bn-A01-p20482459
Bn-A01-p21145008
226,2
Bn-A01-p21136052
Bn-A01-p21309566
227,5
Bn-A01-p21393260
228,1
Bn-scaff_17036_1-p94786
229,3
Bn-A01-p21444041
Bn-A01-p21634646
229,9
Bn-A01-p21758046
Bn-A01-p21776155
Bn-A01-p22016353
231,1
Bn-A01-p22087629
Bn-A01-p22948925
232,6
Bn-A01-p23175111
Bn-A01-p23196022
233,1
Bn-A01-p23259034
Bn-A01-p23078554
235,3
Bn-A01-p23095567
236,8
Bn-A01-p23087707
Bn-A01-p23370802
237,7
Bn-A01-p23472646
238,1
Bn-A01-p23538267
238,5
Bn-A01-p23685978
240,5
Bn-A01-p23842865
243,0
Bn-A01-p24408637
Bn-A01-p24504495
244,3
Bn-A01-p24565084
245,7
Bn-A01-p24707425
247,1
Bn-A01-p24829988
247,5
Bn-A01-p24835306
248,0
Bn-A01-p24848629
248,4
Bn-A01-p24921355
249,0
Bn-Scaffold000164-p133924
250,8
Bn-A01-p26543758
254,5
Bn-A01-p27392534
257,5
Bn-A01-p27796877
260,5
Ex*1012-98
A01-1
Bn-A01-p5635471
0,0
Bn-A01-p5821651
1,3
Bn-A01-p5943392
2,0
Bn-A01-p7827260
6,1
Bn-A01-p7558345
9,5
Bn-A01-p7560785
12,5
Bn-A01-p7137897
14,2
Bn-scaff_17369_1-p841371
16,0
Bn-A01-p7834871
Bn-A01-p9853365
21,2
Bn-A01-p9866468
Bn-A01-p9699152
22,0
Bn-A01-p12535269
26,8
Bn-A01-p11492050
34,4
Bn-A01-p21634646
41,5
Bn-A01-p24425211
51,3
Bn-A01-p27348128
54,0
Bn-A01-p27854949
55,5
Bn-scaff_17972_1-p113066
58,7
Bn-Scaffold000215-p78165
Bn-A01-p28360889
61,5
Bn-A01-p28462071
64,6
Bn-A01-p28497362
65,6
Bn-A01-p25513710
69,3
Bn-A01-p25326722
70,7
Bn-A01-p25470470
Bn-A01-p25072800
75,8
Bn-A01-p25235998
77,9
Ex*1012-98
A01-2
Ex*V8
A01
Ex*R53
A01
Ex*1012-98
A01-1
A012449284
0,0
A012544476
0,8
A012560144
A01r24011
5,9
A01723009
7,1
A01647648
8,2
A01397553
10,4
A01557581
A01228344
14,6
A01282228
A0198316
15,2
A012439241
21,0
A012590705
A01180992
22,6
A01450140
23,1
A01567036
23,8
A01915697
29,5
Annr12569506
30,0
A011413459
33,6
A011482310
A01974344
37,2
A01833504
40,2
A01753379
41,8
A01749100
43,1
A01753032
44,2
A01874438
45,1
A011006969
46,9
Annr12566053
47,8
A011290704
48,8
A011311010
50,4
A011358952
51,3
A011539200
A011586633
52,1
A01r103884
A011672657
54,0
A011730558
55,0
A011744726
55,9
A011876775PA
57,8
A011939611
58,9
A012144328PA
62,0
A012115096
63,3
A012035080
64,0
A011989624
64,7
A011727139
72,7
A011735248
A011894127
76,1
A011912045
76,6
A012194592
80,3
A012327568
82,1
A01r143492
83,3
A01r148871PA
83,9
A012220711
88,0
A012376761PA
92,3
A01r125723
95,5
A012764840
97,1
A012756809
98,1
A012783526
98,5
A012922012
103,3
A013153918
105,9
A013740479PA
109,4
A013481957
109,9
A013391005
111,8
A013044599
115,4
A013312635
118,8
A013438048
119,9
A013567176
121,2
A014049901PA
121,8
A014261277
124,2
A014273529
124,6
A014350529
126,1
A014424388
126,5
A014512396
130,0
C016589693
132,1
C016796821
133,8
A01r265519
135,6
C016916435
137,5
A014826273
138,1
A015042606
141,9
A015217408
143,5
C01r456186
144,4
A014856648
147,1
A015034468
149,8
A01r346634PA
150,3
A01r319411
150,7
A01r321933PA
151,1
A015214185
152,1
A01r351255
153,7
A01r352044
154,0
A015285941
154,2
A015434377PA
155,2
A015594599
155,7
A015927786
156,8
A015409483
160,5
A015433317
161,6
A015671593
162,1
A016237991
164,9
A016193512
165,8
A016487162
168,8
C0352628244het
A016837560
170,1
A016898043PA
170,5
A016916160
171,6
A01r441438
173,7
C01r692173
176,8
A01r443776
177,4
A0136516
0,0
A012518742PA
4,8
A0129929PA
5,6
A012590705PA
6,0
A012519586
6,2
A012628362
7,0
A012449284
A012544476
8,9
A012560144
A01449129
13,0
A01238963
14,4
A01236733PA
16,4
A01282228
17,0
A01540994
20,5
A01557581
A01647648
22,6
A01723009
A01749100
24,7
A01753032
A01755807
25,9
A01554994
27,1
A011559279
41,0
A011561788
A01r103884
41,7
A011413459
43,1
A011586633
44,5
A011727139
49,4
A011729966
50,2
A011775948
A011793043
51,1
A011939611
52,1
A012035080
54,2
A012076602
55,7
A012094564
56,6
C073798925
58,8
A012220711
59,3
A014856648
81,5
A015034468
82,4
A015042606
83,0
A01r319411
84,7
A01r346634
A015154600
A015165863
88,0
C01r456186
A015217408
92,2
A01r356172
A01r350458
92,4
A01r351255
92,6
A01r352044
A015301143
94,0
A016898043PA
102,6
A016898964
A016901133
103,4
A015671593
108,2
A016154320
110,8
A016372207
112,9
C0111577707
116,4
C01r456186
0,0
A015301143
1,3
A015433317
2,0
A01r421146
6,1
A016898964
9,5
A016901133
12,5
A011559279
0,0
A016517958
14,2
A011290704
4,0
C0111542028
A01647648
16,0
C01r692173
10,8
A01557581
A018480162
A01238963
17,4
21,2
A018491979
A01449129
20,2
A018316136
22,0
A01915697
29,2
A0110745972
26,8
A01974344
29,8
A019851927
34,4
A011482310
34,6
A0118221986
41,5
A011729966
38,0
A0120223240
51,3
A012327568
41,9
A0121468568
54,0
A012922012
48,9
C0138257289
55,5
Annr37060149PA
53,0
A0122169874
58,7
Annr2279943hetEx
A0122615340hetEx
61,5
A0122750284hetEx
64,6
A0122790563hetEx
65,6
C0447785592hetEx
69,3
Annr13653671hetEx
70,7
Annr16333220hetEx
A0122535817hetEx
75,8
A0122206203
77,9
A017014266PA
178,7
A016517958
180,8
A016739746PA
A089943074PA
182,0
A017128068
184,7
A017156915
185,2
A017165840
185,7
A017198525
186,7
C0111542028
187,3
A017397322
188,3
A017470783
189,4
A017727524
190,8
A017873510
191,3
A018156987
192,2
A018092397
192,9
A018160121
193,2
A017708346
194,9
A01r522245
195,3
A018335140PA
196,7
Annr26778283
197,9
A018316136
199,2
A018511262PA
A018852208
201,2
A018904034
201,6
A019216766
203,3
A0110131743
204,0
A0110249535
204,8
A0110692073
205,7
A0110691053
206,1
A0110492492
206,4
A0110587334
206,7
A0110745972
206,9
A0110868515
207,3
A0112315278
A0111667464
207,4
A0112597791
A0112938831
207,7
A01r1695008
A0115207080
208,3
Annr33004288
208,5
A0112453679
208,7
C01r1506080
208,9
A0113102994
209,6
A0115220912
210,2
Annr21224005
A0114250417
210,3
A0114264234
211,0
A0114602807
A0114542903
211,8
A07407234
A0116438428
212,7
A0116671599
215,1
Annr42768634
A0117000139
215,9
A0117004019
216,7
C0111542028
119,1
A018316136
121,9
A018132236
125,3
A018491979
129,4
A018511262PA
A018480162
131,6
A018852208
A018904034
134,8
Annr12899384
Annr14718226
136,8
A019216766
140,2
A01r569144
A019851927
141,6
A01r826013
143,2
A0116447786
218,3
A0116559791
218,9
Cnnr56578232
219,8
A0117647106
220,5
A0117232920
221,2
A0117545013
222,0
A01r2116587
A0117823872
226,2
A01r2218346
A0117922735
227,5
A0118016584
228,1
A0118058342
229,3
A0118070953
A0118221986
229,9
A0118351464
A0118386235
A0118645552
231,1
A0118713686
A0119438918
232,6
A0119022504
A0119064492
233,1
A0119116586
A01r2411952
235,3
A01r2392625
236,8
A01r2401249
A0119243847
237,7
A0119346461
238,1
C0134626593
238,5
A0119738682
240,5
A0119908525het
243,0
A0120206623
A0120308731
244,3
A0120363718
245,7
A0120475601
247,1
A0120561724
247,5
A0120567057
248,0
A0120581194
248,4
A01r2519289
249,0
A0120717861
250,8
Annr9893318PA
254,5
A0121524914
257,5
C0138129852
260,5

## Slide 2
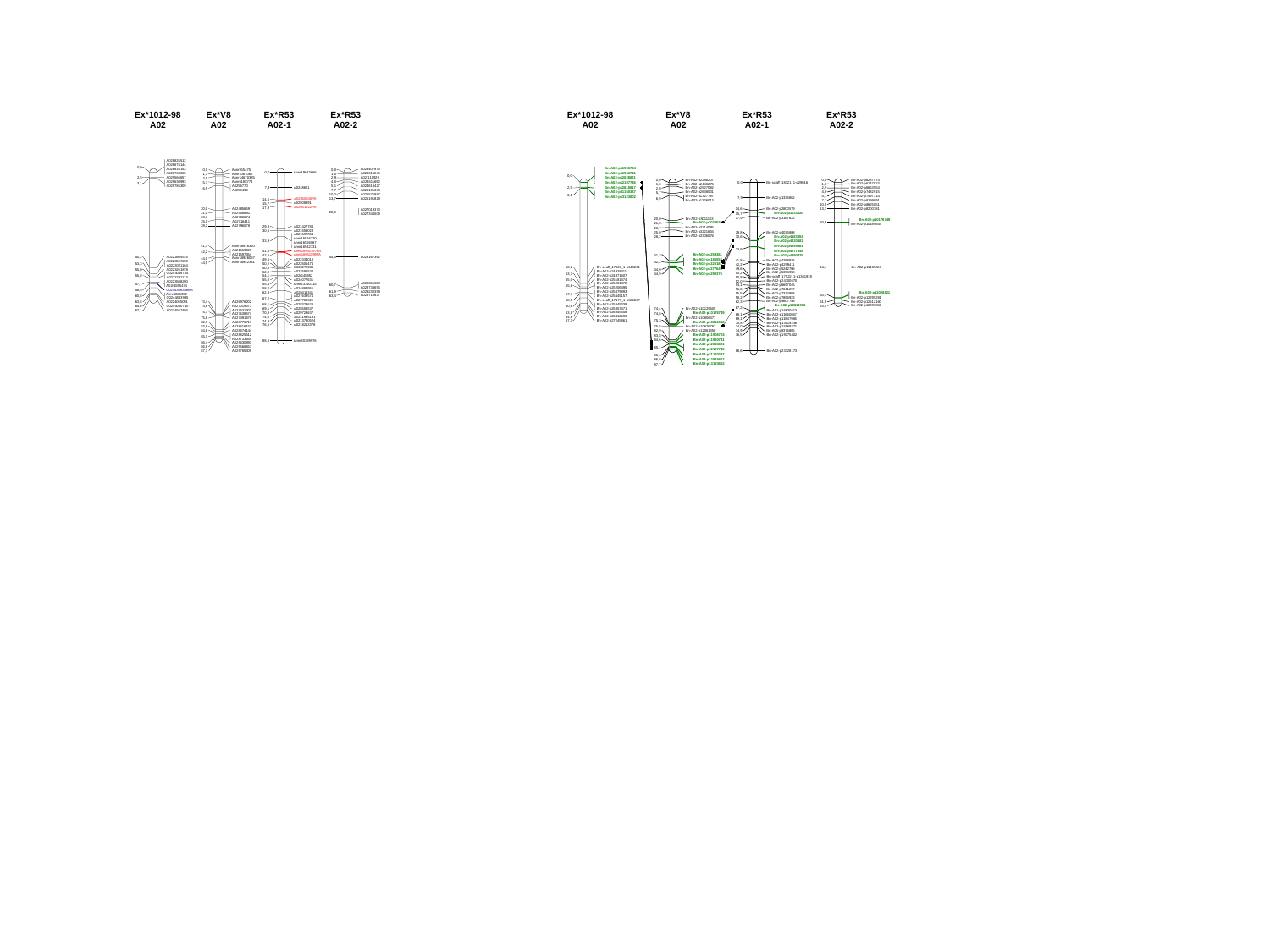

Ex*1012-98
A02
Ex*V8
A02
Ex*R53
A02-1
Ex*R53
A02-2
A028825512
A028674144
0,0
A028616153
A023407873
Annr915475
0,0
0,0
Annr19643666
0,0
A028733655
A023316245
Annr1061060
1,0
1,3
A029566657
2,5
A02r110929
Annr14670385
2,9
2,0
A029630993
A024511692
Annr6188774
4,0
5,7
3,1
A029785309
A024618427
A0256770
5,1
A0248621
7,9
6,9
A025191339
A0258490
7,7
A025575897
10,0
A025191829
13,7
A02350645PA
14,6
A02508891
15,7
A02651222PA
17,9
A02485659
20,0
A027019373
20,8
A02508891
21,0
A027344509
A02708874
23,7
A02716411
25,0
A02796078
28,2
A021427784
29,8
A021559029
30,6
A021597354
Annr16916320
33,9
Annr16926067
Annr16916320
41,3
Annr16942331
A021559029
Annr16950767PA
41,8
42,2
A021597354
Annr16951199PA
42,2
A028167362
44,4
A0222605515
50,2
Annr16926067
44,0
A022334018
49,6
A0223067298
Annr16942331
44,9
53,3
A022505474
50,2
A0223021164
C025273938
50,8
A0223261878
55,0
A023586534
52,0
C0244068754
A02r148852
55,8
54,1
A0223281113
A024077631
55,0
A0223508290
A029011503
Annr23340315
57,7
55,5
60,7
A02r1555474
A028733655
A024882955
58,2
C0244366386het
58,5
A029230359
61,9
A026411341
62,3
Annr6821952
A029710637
63,4
60,8
A027638574
C0244883995
67,2
A027788321
A026976202
A0224065091
74,4
63,8
A028378628
68,1
A027019373
74,8
C0245060740
64,6
A028558537
69,1
A027631301
A0224567493
67,1
75,3
A029728637
70,8
A027638574
A0211995191
74,0
A027491670
75,8
A0213790524
74,9
A028776717
82,9
A0218213378
76,5
A028616153
83,8
A028674144
84,6
A028825512
85,1
A028733655
Annr30389976
88,6
A029630993
86,4
A029566657
86,8
A029785309
87,7
Ex*1012-98
A02
Ex*V8
A02
Ex*R53
A02-1
Ex*R53
A02-2
Bn-A02-p11906704
Bn-A02-p11960741
0,0
Bn-A02-p12028821
Bn-A02-p6237474
Bn-A02-p2288207
0,0
0,0
Bn-scaff_18321_1-p29016
Bn-A02-p12107746
0,0
Bn-A02-p6147823
Bn-A02-p2443275
1,0
1,3
Bn-A02-p12815817
Bn-A02-p6653554
2,5
Bn-A02-p2527082
2,9
2,0
Bn-A02-p7482915
Bn-A03-p31160337
Bn-A02-p2638031
4,0
5,7
3,1
Bn-A02-p7597314
Bn-A02-p1327787
5,1
Bn-A02-p13123802
Bn-A02-p1335862
7,9
6,9
Bn-A02-p8199891
Bn-A02-p1326013
7,7
Bn-A02-p8625851
10,0
Bn-A02-p8200381
13,7
Bn-A02-p2800579
14,6
Bn-A02-p3033620
15,7
Bn-A02-p3167622
17,9
Bn-A02-p3011103
20,0
Bn-A02-p10176749
20,8
Bn-A02-p3033620
21,0
Bn-A02-p10485644
Bn-A02-p3214095
23,7
Bn-A02-p3221634
25,0
Bn-A02-p4035808
29,8
Bn-A02-p3306076
28,2
Bn-A02-p4183553
30,6
Bn-A02-p4220181
Bn-A02-p4259381
33,9
Bn-A02-p4277649
Bn-A02-p4259381
41,3
Bn-A02-p4290375
Bn-A02-p4183553
Bn-A02-p4298976
41,8
42,2
Bn-A02-p4220181
Bn-A02-p4299411
42,2
Bn-A02-p11455369
44,4
Bn-scaff_17623_1-p648231
50,2
Bn-A02-p5242706
Bn-A02-p4277649
49,6
44,0
Bn-A02-p24928311
Bn-A02-p5394958
50,2
53,3
Bn-A02-p4290375
44,9
Bn-A02-p24971607
Bn-scaff_17522_1-p1361919
50,8
Bn-A02-p25181474
55,0
Bn-A02-p24700670
52,0
Bn-A02-p25201372
Bn-A02-p6697045
54,1
55,8
Bn-A02-p25206495
Bn-A02-p7041297
55,0
Bn-A02-p25479893
Bn-A02-p12028821
Bn-A02-p7324958
55,5
57,7
60,7
Bn-A02-p25445157
Bn-A02-p7896923
58,2
Bn-A02-p12295281
Bn-scaff_17177_1-p836037
58,5
Bn-A02-p9607756
62,3
Bn-A02-p12512160
61,9
Bn-A02-p25845339
Bn-A02-p10811918
Bn-A02-p12999856
63,4
60,8
67,2
Bn-A02-p25857471
Bn-A02-p10129605
74,4
Bn-A01-p16630613
Bn-A02-p26345558
63,8
Bn-A02-p10176749
74,8
Bn-A02-p11660867
68,1
Bn-A02-p26412693
64,6
Bn-A02-p10806477
Bn-A02-p11847986
69,1
Bn-A02-p27245861
75,3
67,1
Bn-A02-p10811918
Bn-A02-p13019236
70,8
Bn-A02-p10626782
Bn-A02-p15569271
75,8
74,0
Bn-A02-p12061350
Bn-A05-p8376865
82,9
74,9
Bn-A02-p11906704
Bn-A02-p19175402
76,5
83,8
Bn-A02-p11960741
84,6
Bn-A02-p12028821
85,1
Bn-A02-p12107746
Bn-A02-p23728173
88,6
Bn-A03-p31160337
86,4
Bn-A02-p12815817
86,8
Bn-A02-p13123802
87,7

## Slide 3
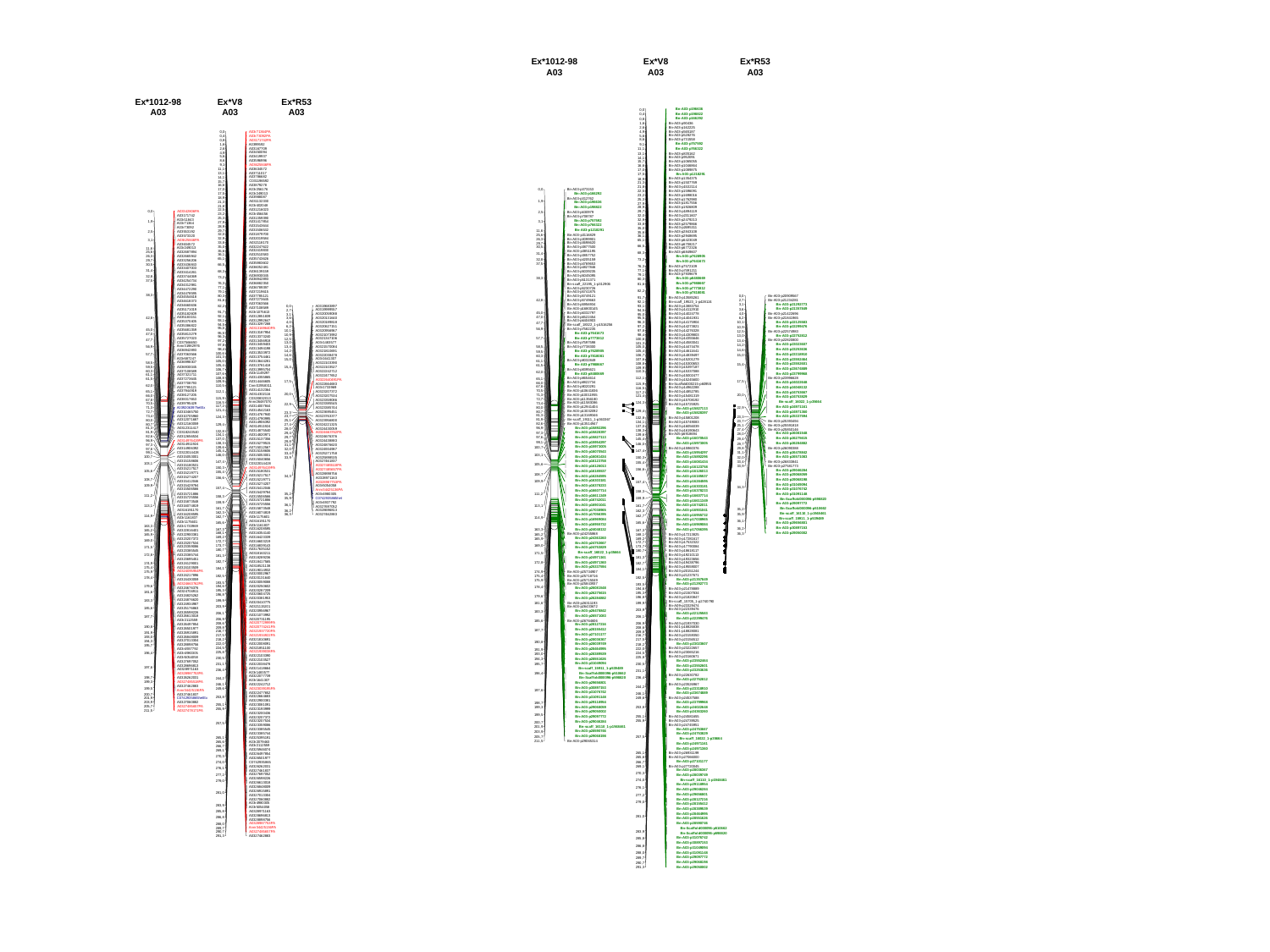

Ex*1012-98
A03
Ex*V8
A03
Ex*R53
A03
Bn-A03-p196636
0,0
Bn-A03-p198822
0,4
Bn-A03-p166292
0,8
Bn-A03-p90436
1,6
Bn-A03-p162225
2,6
Bn-A03-p565187
4,9
Bn-A03-p528276
5,6
Bn-A03-p731558
8,6
Bn-A03-p757592
9,1
Bn-A03-p766322
11,1
Bn-A03-p835162
13,1
Bn-A03-p952091
14,1
Bn-A03-p1065055
15,7
Bn-A03-p1046864
16,6
Bn-A03-p1089875
17,0
Bn-A03-p1218291
17,5
Bn-A03-p1354375
18,9
Bn-A03-p1507769
21,3
Bn-A03-p1532114
21,8
Bn-A03-p1596091
22,5
Bn-A03-p1698016
23,2
Bn-A03-p1762980
25,3
Bn-A03-p1817556
27,9
Bn-A03-p1926809
28,9
Bn-A03-p1894119
29,7
Bn-A03-p2311607
32,0
Bn-A03-p2479213
32,9
Bn-A03-p2579866
33,8
Bn-A03-p2695311
35,0
Bn-A03-p2843108
35,6
Bn-A03-p2948695
36,1
Bn-A03-p6423049
65,1
Bn-A03-p6708217
66,5
Bn-A03-p6772326
Bn-A03-p6849607
68,3
Bn-A03-p7628905
73,2
Bn-A03-p7641673
Bn-A03-p7572109
76,3
Bn-A03-p7491211
77,1
Bn-A03-p7939679
79,1
Bn-A03-p8480669
80,3
Bn-A03-p7988667
81,6
Bn-A03-p7773812
82,2
Bn-A03-p7818081
Bn-A03-p13585261
91,7
Bn-scaff_19523_1-p428124
92,1
Bn-A03-p13883754
93,1
Bn-A03-p14112910
94,5
Bn-A03-p14024778
95,0
Bn-A03-p14041931
95,5
Bn-A03-p14175904
96,3
Bn-A03-p14273821
Bn-A03-p373153
0,0
Bn-A03-p166292
Bn-A03-p312762
1,9
Bn-A03-p196636
Bn-A03-p198822
Bn-A03-p630979
2,5
Bn-A03-p708787
Bn-A03-p757592
3,1
Bn-A03-p766322
Bn-A03-p1218291
11,6
Bn-A03-p3116829
25,6
Bn-A03-p3099901
26,3
Bn-A03-p3698620
29,7
Bn-A03-p3877500
30,5
Bn-A03-p3851195
31,4
Bn-A03-p3857752
Bn-A03-p4205159
32,8
Bn-A03-p4769653
37,5
Bn-A03-p4827846
Bn-A03-p5039235
Bn-A03-p5045095
38,3
Bn-A03-p5131371
Bn-scaff_22195_1-p312906
Bn-A03-p5230726
Bn-A03-p5741875
Bn-A03-p5749121
Bn-A03-p5749663
42,8
Bn-A03-p5956934
Bn-A03-p16800145
Bn-A03-p6332797
45,0
Bn-A03-p6522454
47,0
Bn-A03-p6404903
47,7
Bn-scaff_18322_1-p1516256
Bn-A03-p7582235
56,9
Bn-A03-p7641673
Bn-A03-p7773812
57,7
Bn-A03-p7597598
Bn-A03-p7738300
58,5
Bn-A03-p7628905
59,5
Bn-A03-p7818081
60,3
Bn-A03-p8032849
61,1
Bn-A03-p7988667
61,5
Bn-A03-p8395621
62,0
Bn-A03-p8480669
Bn-A03-p8654614
65,1
Bn-A03-p8822734
66,0
Bn-A03-p9020291
67,8
Bn-A03-p10641836
70,5
Bn-A03-p10011955
71,3
Bn-A03-p11356180
72,7
Bn-A03-p11583086
73,4
Bn-A03-p12941404
80,0
Bn-A03-p13032092
80,7
Bn-A03-p13169346
81,3
Bn-scaff_19111_1-p363367
81,9
Bn-A03-p13514947
82,6
Bn-A03-p15892296
96,9
Bn-A03-p15828397
97,3
Bn-A03-p15827113
97,6
Bn-A03-p15954297
99,1
Bn-A03-p15973005
100,7
Bn-A03-p16070543
103,1
Bn-A03-p16081434
Bn-A03-p20909567
0,0
Bn-A03-p21234281
2,7
Bn-A03-p21292773
3,1
Bn-A03-p21357849
3,6
Bn-A03-p21422696
4,0
Bn-A03-p21841965
6,2
Bn-A03-p22125583
10,1
97,2
Bn-A03-p14276328
97,8
Bn-A03-p14309803
98,4
Bn-A03-p14355646
100,6
Bn-A03-p14583041
101,3
Bn-A03-p14474478
105,0
Bn-A03-p14611641
105,4
Bn-A03-p14839497
106,7
Bn-A03-p15201279
107,6
Bn-A03-p15300653
108,8
Bn-A03-p15397187
109,9
Bn-A03-p15337088
110,5
Bn-A03-p15002477
112,1
Bn-A03-p15245600
Bn-Scaffold000215-p60955
115,9
Bn-A03-p14962288
116,5
Bn-A03-p14852785
117,2
Bn-A03-p15481319
121,4
Bn-A03-p15708192
124,3
Bn-A03-p15725925
Bn-A03-p15827113
129,4
Bn-A03-p15828397
Bn-A03-p15801208
132,8
Bn-A03-p15749083
134,1
Bn-A03-p16056039
137,0
Bn-A03-p16190643
138,3
Bn-A05-p6918694
139,6
Bn-A03-p16070543
145,4
Bn-A03-p15973005
146,0
Bn-A03-p15960376
147,4
Bn-A03-p15954297
Bn-A03-p15892296
150,3
Bn-A03-p16081434
155,4
Bn-A03-p16123758
156,6
Bn-A03-p16126013
Bn-A03-p16185537
Bn-A03-p16284595
157,4
Bn-A03-p16303181
Bn-A03-p16378233
158,3
Bn-A03-p16607714
159,9
Bn-A03-p16611349
Bn-A03-p16742011
161,7
Bn-A03-p16901041
162,3
Bn-A03-p16955732
162,7
Bn-A03-p17038965
165,6
Bn-A03-p16969084
Bn-A03-p17056395
167,3
Bn-A03-p17213825
168,1
Bn-A03-p17281617
169,2
Bn-A03-p17532322
172,7
Bn-A03-p17790084
173,7
Bn-A03-p18618117
180,7
Bn-A03-p19210113
181,3
Bn-A03-p19323656
Bn-A03-p19438796
182,7
Bn-A03-p19559007
Bn-A03-p20151244
Bn-A03-p22299476
10,9
Bn-A03-p22574983
12,5
Bn-A03-p22752812
13,0
Bn-A03-p22820800
13,4
Bn-A03-p23023607
14,2
Bn-A03-p23253636
14,6
Bn-A03-p23318910
15,0
Bn-A03-p23552464
Bn-A03-p23552601
15,4
Bn-A03-p23674889
Bn-A03-p23799968
Bn-A03-p23996628
17,5
Bn-A03-p24022648
Bn-A03-p24048132
Bn-A03-p24753667
20,0
Bn-A03-p24753829
Bn-scaff_16022_1-p35664
Bn-A03-p24971161
22,9
Bn-A03-p24971360
Bn-A03-p25337594
23,3
Bn-A03-p25393494
23,7
Bn-A03-p25591818
25,1
Bn-A03-p25850146
27,4
Bn-A03-p26081548
28,0
Bn-A03-p26275615
29,4
Bn-A03-p26284882
29,7
Bn-A03-p26090388
29,8
Bn-A03-p26478842
31,1
Bn-A03-p26571083
32,0
Bn-A03-p16123758
Bn-A03-p26833841
33,4
105,6
Bn-A03-p27581773
33,9
Bn-A03-p16126013
Bn-A03-p29046284
Bn-A03-p16185537
108,7
Bn-A03-p29068059
Bn-A03-p16284595
Bn-A03-p29068198
Bn-A03-p16303181
109,9
Bn-A03-p31049094
Bn-A03-p16378233
34,3
Bn-A03-p31076742
Bn-A03-p16607714
111,2
Bn-A03-p31091148
Bn-A03-p16611349
Bn-Scaffold000096-p598820
Bn-A03-p16742011
Bn-A03-p29097772
Bn-A03-p16901041
113,1
Bn-Scaffold000096-p510662
35,2
Bn-A03-p17038965
184,1
Bn-A03-p21237671
192,5
Bn-A03-p21357849
Bn-A03-p21292773
193,5
Bn-A03-p21478889
194,6
Bn-A03-p21507834
195,3
Bn-A03-p21820847
196,8
Bn-scaff_15705_1-p2740790
199,9
Bn-A09-p23329474
Bn-A03-p22339476
203,9
Bn-A03-p22125583
206,1
Bn-A03-p22299476
Bn-A03-p21937030
206,9
Bn-A01-p18826838
208,6
Bn-A01-p18828081
209,8
Bn-A03-p23159350
216,7
Bn-A03-p23156512
217,5
Bn-A03-p23023607
218,2
Bn-A03-p23222657
222,0
Bn-A03-p23065216
224,5
Bn-A03-p23160671
225,8
Bn-A03-p23552464
230,5
Bn-A03-p23552601
Bn-A03-p23253636
231,1
Bn-A03-p22630782
236,4
Bn-A03-p22752812
Bn-A03-p23526967
244,2
Bn-A03-p23318910
Bn-A03-p23674889
246,1
Bn-A03-p24537588
249,6
Bn-A03-p23799968
Bn-A03-p24022648
253,8
Bn-A03-p24363260
Bn-A03-p24581655
255,1
Bn-A03-p24739525
255,9
Bn-A03-p24745951
Bn-A03-p24753667
Bn-A03-p24753829
257,5
Bn-scaff_16022_1-p35664
Bn-A03-p24971161
Bn-A03-p24971360
Bn-A03-p26931199
265,1
Bn-A03-p27066000
265,6
Bn-A03-p27101177
266,7
Bn-A03-p27720045
269,1
Bn-A03-p28036367
270,3
Bn-A03-p28039749
Bn-scaff_16110_1-p1948461
274,0
Bn-A03-p29118954
276,1
Bn-A03-p29046284
Bn-A03-p29656801
277,2
Bn-A03-p28127216
279,0
Bn-A03-p28155412
Bn-A03-p28389539
Bn-A03-p28464995
281,0
Bn-A03-p28551626
Bn-A03-p28590746
Bn-Scaffold000096-p510662
283,9
Bn-Scaffold000096-p598820
Bn-A03-p31076742
285,8
Bn-A03-p30897153
286,8
Bn-A03-p31049094
Bn-A03-p31091148
Bn-scaff_16110_1-p1948461
35,9
Bn-A03-p17056395
114,9
Bn-scaff_15911_1-p539489
Bn-A03-p16969084
36,1
Bn-A03-p29656801
Bn-A03-p16955732
Bn-A03-p30897153
36,2
Bn-A03-p24048132
163,3
Bn-A03-p29050002
36,3
Bn-A03-p24255868
165,2
Bn-A03-p24363260
165,9
Bn-A03-p24753667
169,0
Bn-A03-p24753829
Bn-scaff_16022_1-p35664
171,5
Bn-A03-p24971161
Bn-A03-p24971360
172,8
Bn-A03-p25337594
Bn-A03-p25734907
174,9
Bn-A03-p25718716
175,4
Bn-A03-p25715049
175,9
Bn-A03-p25843937
178,4
Bn-A03-p26081548
Bn-A03-p26275615
179,6
Bn-A03-p26284882
Bn-A03-p26311193
181,6
Bn-A03-p26433672
Bn-A03-p26478842
183,3
Bn-A03-p26571083
Bn-A03-p26764606
185,6
Bn-A03-p28127216
Bn-A03-p28155412
187,7
Bn-A03-p27101177
Bn-A03-p28036367
190,8
Bn-A03-p28039749
Bn-A03-p28464995
191,9
Bn-A03-p28389539
193,0
Bn-A03-p28551626
194,3
Bn-A03-p31049094
195,7
Bn-scaff_15911_1-p539489
Bn-Scaffold000096-p510662
196,4
Bn-Scaffold000096-p598820
Bn-A03-p29656801
Bn-A03-p30897153
197,6
Bn-A03-p31076742
Bn-A03-p31091148
Bn-A03-p29118954
198,7
Bn-A03-p29068059
199,3
Bn-A03-p29050002
199,5
Bn-A03-p29097772
Bn-A03-p29046284
200,7
Bn-scaff_16110_1-p1948461
201,9
Bn-A03-p28590746
203,9
Bn-A03-p29068198
205,7
Bn-A03-p29065314
211,5
288,0
Bn-A03-p29097772
289,7
Bn-A03-p29068198
290,7
Bn-A03-p29050002
291,3
Ex*1012-98
A03
Ex*V8
A03
Ex*R53
A03
A03r71364PA
0,0
A03r73092PA
0,4
A03171742PA
0,8
A0399592
1,6
A03167709
2,6
A03450094
4,9
A03419937
5,6
A03596996
8,6
A03625846PA
9,1
A03634572
11,1
A03711017
13,1
A03786682
14,1
C031196592
15,7
A03879278
16,6
A03r256176
17,0
A03r249013
17,5
A03988087
18,9
A031132330
21,3
A03r402048
21,8
A031216023
22,5
A03r456456
23,2
A031359390
25,3
A031417854
27,9
A031543644
28,9
A031506532
29,7
A031879700
32,0
A032019584
32,9
A032118170
33,8
A032247622
35,0
A032419830
35,6
A032510583
36,1
A035743626
65,1
A035983602
66,5
A036052481
A036139159
68,3
A036930045
73,2
A036942893
A036882350
76,3
A036789397
77,1
A037219615
79,1
A037785121
80,3
A037273645
81,6
A037063566
82,2
A037108589
A03r1075613
91,7
A0312851839
92,1
A0312992647
93,1
A0313297288
94,5
A0313169640PA
95,0
A0313187954
95,5
A0313373240
96,3
A0313456918
A03342806PA
0,0
A03171742
A03r11843
1,9
A03r71364
A03r73092
A03553192
2,5
A03573320
A03625846PA
3,1
A03634572
A03r249013
11,6
A032687894
25,6
A032665942
26,3
A033256206
29,7
A033436843
30,5
A033407003
31,4
A033414261
A033744069
32,8
A034254734
37,5
A034312981
A034472290
A034478595
38,3
A034554618
A034618373
A034660636
A035171019
A035182609
A035183151
42,8
A035370405
A035386822
A035681359
45,0
A035813279
47,0
A035727023
47,7
C037566650
Annr31982976
56,9
A036942893
A037063566
57,7
A03r687247
A036998307
58,5
A036930045
59,5
A037108589
60,3
A037321711
61,1
A037273645
61,5
A037708793
62,0
A037785121
A037944919
65,1
A038127205
66,0
A038317653
67,8
A039795429
70,5
A039206397hetEx
71,3
A0310465750
72,7
A0310705960
73,4
A0312071687
80,0
A0312160059
80,7
A0312311417
81,3
C0318243540
81,9
A0312656534
82,6
A0314976419PA
96,9
A0314911924
97,3
A0314906392
97,6
C0322014428
99,1
A0315053001
100,7
A0315158606
103,1
A0315180531
A0319683897
0,0
A0319999557
2,7
A0320059088
3,1
A0320131640
3,6
A0320189818
4,0
A0320627151
6,2
A0320956967
10,1
A0321073992
10,9
A0321347106
12,5
97,2
A0313459403
97,8
A0313494198
98,4
A0313533972
100,6
A0313754461
101,3
A0313644281
105,0
A0313781418
105,4
A0313995704
106,7
A03r1149297
107,6
A0314355865
108,8
A0314446605
109,9
Cnnr32958311
110,5
A0314132364
112,1
A0314302116
C0320832013
115,9
Annr26487070
116,5
A0314007844
117,2
A0314542163
121,4
A0314767940
124,3
A0314790995
A0314906392
129,4
A0314911924
A0314875540
132,8
A0314820971
134,1
A0315137356
137,0
A0315279515
138,3
A0715012567
139,6
A0315158606
145,4
A0315053001
146,0
A0315040656
147,4
C0322014428
A0314976419PA
150,3
A0315180531
155,4
A0315217517
156,6
A0315219771
A0315274207
A0315412046
157,4
A0315429784
A0315506566
158,3
A0315721886
159,9
A0315725556
A0315873548
161,7
A0316074819
162,3
A03r1175601
162,7
A0316191170
165,6
A03r1161837
A0316208595
167,3
A0316354140
168,1
A0316423339
169,2
A0316603219
172,7
A0316839143
173,7
A0317635152
180,7
A0318163211
181,3
A0318289236
A0318417565
182,7
A0318521138
A0319014932
A03r1483577
13,0
A0321570084
13,4
A0321810691
14,2
A0322038478
14,6
A03r1641307
15,0
A0322103390
A0322103527
15,4
A0322242712
A0322477652
A0322640491PA
17,5
A0322664683
A03r1733969
A0323207372
20,0
A0323207534
A0323359086
A0323385545
22,9
A0323385744
A0323695451
23,3
A0323763377
23,7
A0323956800
25,1
A0324221325
27,4
A0324430059
28,0
A0324663762PA
29,4
A0324676376
29,7
A0324438883
29,8
A0324876620
31,1
A0324934987
32,0
A0325271758
33,4
A0325898535
33,9
A0327461837
A0327485518PA
A0315217517
A0327485657PA
105,6
A0315219771
A0328898756
34,3
A0315274207
A0328971163
108,7
A0315412046
A0328987753PA
A0315429784
109,9
A03r5054058
A0315506566
Annr34425136PA
A0315721886
A03r4980305
35,2
111,2
A0315725556
C0742935865het
35,9
A0315873548
A03r4937792
36,1
A0316074819
113,1
A0327697052
184,1
A0320002967
192,5
A0320131640
A0320059088
193,5
A0320250602
194,6
A0320287209
195,3
A0320604725
196,8
A0320381953
199,9
A0320410775
A0321115201
203,9
A0320956967
206,1
A0321073992
A0320731195
206,9
A0320772999PA
208,6
A0320774241PA
209,8
A0321937720PA
216,7
A0321934922PA
217,5
A0321810691
218,2
A0322008091
222,0
A0321851100
224,5
A0321939038PA
225,8
A0322103390
230,5
A0322103527
A0322038478
231,1
A0321418664
236,4
A03r1483577
A0322077739
244,2
A03r1641307
A0322242712
246,1
A0323039195PA
249,6
A0322477652
A0322664683
253,8
A0322900381
A0323081091
255,1
A0323193999
255,9
A0323200436
A0323207372
A0323207534
257,5
A0323359086
A0323385545
A0323385744
A0325395191
265,1
A03r2079463
265,6
A03r2112559
266,7
A0325946074
269,1
A0326497854
270,3
A0326501977
C0742935865
274,0
A0326262031
276,1
A0327461837
A0327697052
277,2
A0326598226
279,0
A0326613018
A0326848009
A0326915891
281,0
A0327013304
A0327060882
A03r4980305
283,9
A03r5054058
A0328971163
285,8
A0328696813
286,8
A0328898756
A0328987753PA
A0316191170
A0328696813
36,2
A0316208595
A0327462883
36,3
114,9
A03r1161837
A03r1175601
A03r1733969
163,3
A0322816401
165,2
A0322900381
165,9
A0323207372
169,0
A0323207534
A0323359086
171,5
A0323385545
A0323385744
172,8
A0323695451
A0324129001
174,9
A0324103509
175,4
A0324095954PA
175,9
A0324217896
178,4
A0324430059
A0324663762PA
179,6
A0324676376
A0324704911
181,6
A0324825262
A0324876620
183,3
A0324934987
A0325176863
185,6
A0326598226
A0326613018
187,7
A03r2112559
A0326497854
190,8
A0326501977
A0326915891
191,9
A0326848009
193,0
A0327013304
194,3
A0328898756
195,7
A03r4937792
A03r4980305
196,4
A03r5054058
A0327697052
A0328696813
197,6
A0328971163
A0328987753PA
A0326262031
198,7
A0327485518PA
199,3
A0327462883
199,5
Annr34425136PA
A0327461837
200,7
C0742935865hetEx
201,9
A0327060882
203,9
A0327485657PA
205,7
A0327478171PA
211,5
288,0
Annr34425136PA
289,7
A0327485657PA
290,7
A0327462883
291,3

## Slide 4
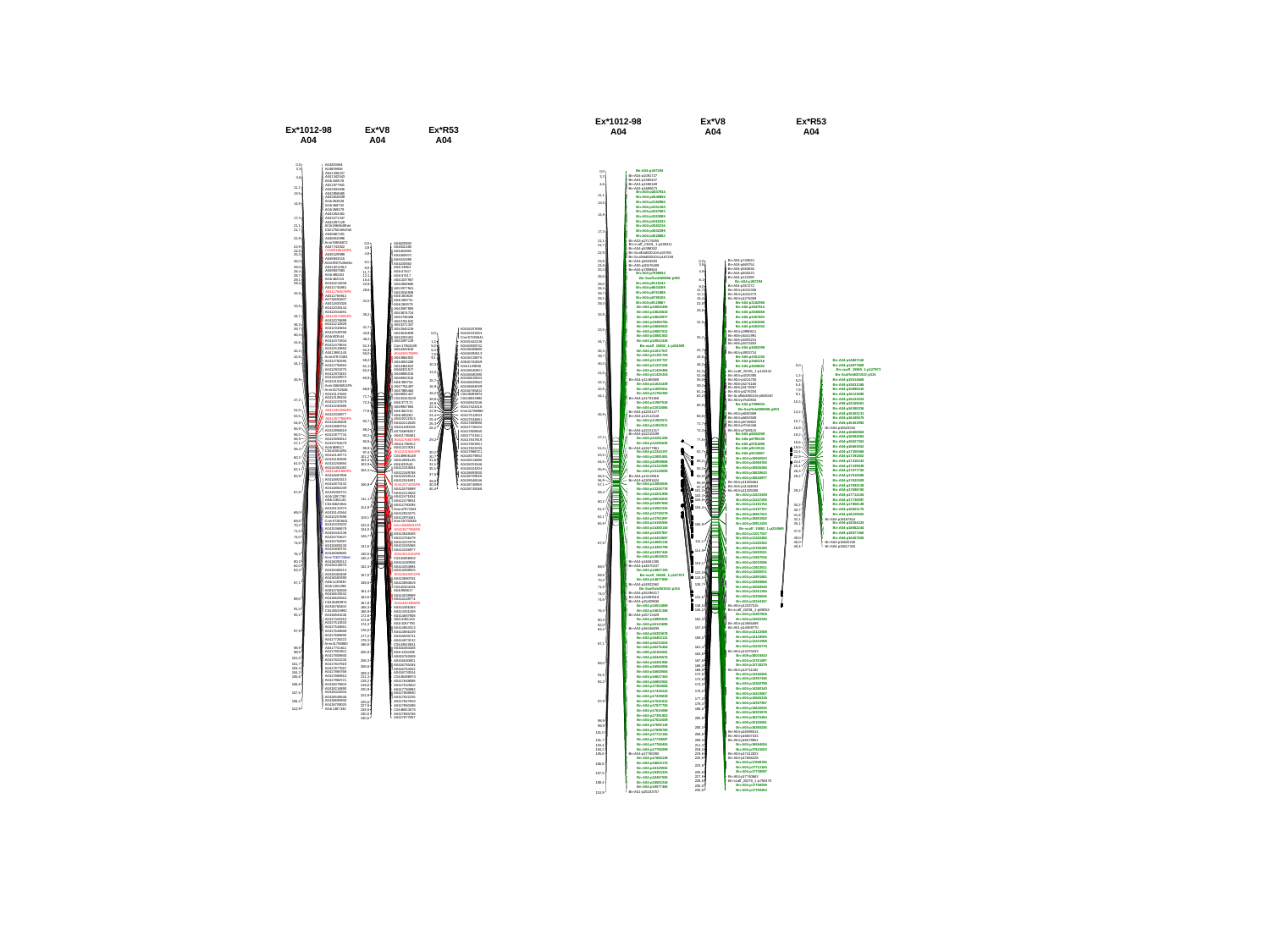

Ex*1012-98
A04
Ex*V8
A04
Ex*R53
A04
Bn-A04-p267294
0,0
Bn-A04-p1091727
3,3
Bn-A04-p1585447
Bn-A04-p1590169
4,6
Bn-A04-p1680673
Bn-A04-p2847914
11,1
Bn-A04-p2845855
Bn-A04-p3182956
13,5
Bn-A04-p3351150
Bn-A04-p3267803
14,8
Bn-A04-p3263085
Bn-A04-p3262432
Bn-A04-p3583216
Bn-A04-p3602299
17,3
Bn-A04-p3628682
Bn-A03-p27179255
21,1
Bn-scaff_23401_1-p188311
21,7
Bn-A04-p5398352
Bn-Scaffold000104-p33765
22,9
Bn-Scaffold000104-p167338
Bn-A04-p6533593
23,9
Bn-A05-p20676409
24,8
Bn-A04-p7808804
25,3
Bn-A04-p7598934
26,0
Bn-Scaffold005956-p293
Bn-A04-p9119142
28,0
Bn-A04-p8834259
28,4
Bn-A04-p8761898
28,7
Bn-A04-p8790105
29,1
Bn-A04-p9119867
29,4
Bn-A04-p10600455
Bn-A04-p10628643
32,9
Bn-A04-p10628977
Bn-A04-p10494765
Bn-A04-p10680523
33,5
Bn-A04-p10867512
Bn-A04-p10881502
Bn-A04-p10912425
34,7
Bn-scaff_15852_1-p234569
Bn-A04-p11017537
36,3
Bn-A04-p11191754
39,7
Bn-A04-p11197707
40,4
Bn-A04-p11247206
Bn-A04-p11420466
41,6
Bn-A04-p11425304
Bn-A04-p11465588
42,2
Bn-A04-p11633439
Bn-A04-p11693521
42,8
Bn-A04-p11765380
44,1
Bn-A04-p11791366
Bn-A04-p11957518
Bn-A04-p12010066
Bn-A04-p12051277
45,9
Bn-A04-p12112148
Bn-A04-p11900571
Bn-A04-p11923511
Bn-A04-p12151317
Bn-A04-p12210199
47,2
Bn-A04-p12251256
Bn-A04-p12256636
Bn-A04-p12677961
51,0
Bn-A04-p12344157
53,5
Bn-A04-p12891881
Bn-A04-p12959668
54,6
Bn-A04-p13122589
55,9
Bn-A04-p13129093
Bn-A04-p13119516
56,5
Bn-A04-p13091624
56,9
Bn-A04-p13068545
57,1
Bn-A04-p745024
0,0
Bn-A04-p665754
3,8
Bn-A04-p583036
4,8
Bn-A04-p600223
Bn-A04-p122280
8,1
Bn-A04-p267294
Bn-A04-p357272
9,0
Bn-A04-p1032336
11,7
Bn-A04-p1042273
12,1
Bn-A04-p1276308
15,4
Bn-A04-p3182956
22,8
Bn-A04-p2847914
28,6
Bn-A04-p2845855
Bn-A04-p3267803
Bn-A04-p3263085
31,5
Bn-A04-p3262432
Bn-A04-p3990611
Bn-A06-p5441991
35,2
Bn-A06-p5393231
Bn-A06-p5372653
Bn-A04-p3602299
41,7
Bn-A04-p3933714
Bn-A04-p3351150
43,8
Bn-A04-p3583216
46,2
Bn-A04-p3628682
Bn-scaff_20191_1-p143344
51,3
Bn-A04-p4325390
52,4
Bn-A04-p4224705
55,0
Bn-A06-p5274160
59,2
Bn-A06-p5179267
Bn-A04-p4276334
61,1
Bn-Scaffold000104-p563040
62,2
Bn-A04-p7540834
Bn-A04-p7598934
65,9
Bn-Scaffold005956-p293
Bn-A04-p6550369
68,8
Bn-A04-p6653508
Bn-A04-p6748822
71,7
Bn-A04-p7044446
Bn-A04-p7349115
72,3
Bn-A04-p8834259
Bn-A04-p8790105
77,6
Bn-A04-p8761898
Bn-A04-p9119142
82,7
Bn-A04-p9119867
Bn-A04-p10680523
88,2
Bn-A04-p10494765
Bn-A04-p10600455
92,2
Bn-A04-p10628643
92,8
Bn-A04-p10628977
Bn-A04-p11026664
95,9
Bn-A04-p11164883
97,4
Bn-A04-p11325008
101,2
Bn-A04-p11633439
103,3
Bn-A04-p11247206
103,9
Bn-A04-p11191754
104,4
Bn-A04-p11197707
Bn-A04-p10867512
Bn-A04-p10881502
Bn-A04-p10912425
106,9
Bn-scaff_15852_1-p234569
Bn-A04-p11017537
Bn-A04-p11420466
111,1
Bn-A04-p11425304
Bn-A04-p11765380
114,9
Bn-A04-p11693521
Bn-A04-p14807150
Bn-A04-p14877869
0,0
Bn-scaff_15585_1-p127973
Bn-Scaffold001532-p334
1,2
Bn-A04-p15514869
5,0
Bn-A04-p15631388
5,4
Bn-A04-p15895510
7,9
Bn-A04-p16123695
9,1
Bn-A04-p16233024
10,3
Bn-A09-p32165581
Bn-A04-p16365226
13,2
Bn-A04-p16403131
Bn-A04-p16445675
15,7
Bn-A04-p16451992
Bn-A04-p16523010
16,9
Bn-A04-p16608584
18,2
Bn-A04-p16584084
Bn-A04-p16627363
18,6
Bn-A04-p16663552
19,9
Bn-A04-p17300068
22,4
Bn-A04-p17391802
22,9
Bn-A04-p17416243
24,4
Bn-A04-p17439839
25,4
Bn-A04-p17577700
26,3
Bn-A04-p17615088
28,2
Bn-A04-p17632009
Bn-A04-p17692138
Bn-A04-p13245778
Bn-A04-p17698780
29,2
58,4
Bn-A04-p13241959
Bn-A04-p17712184
Bn-A09-p30016810
Bn-A04-p17738097
60,2
Bn-A04-p13497608
Bn-A04-p17858149
30,2
Bn-A04-p13583325
61,5
Bn-A04-p18003170
30,7
Bn-A04-p13738279
Bn-A04-p18129552
31,6
62,1
Bn-A04-p13761897
Bn-A04-p18167614
32,1
Bn-A04-p14156506
Bn-A04-p18354325
65,9
35,1
Bn-A04-p14380140
Bn-A04-p18562244
37,5
Bn-A04-p14397957
Bn-A04-p18577466
Bn-A04-p14410667
Bn-A04-p18457550
39,0
Bn-A04-p14565138
Bn-A04-p18620226
40,0
67,8
Bn-A04-p18617343
40,4
Bn-A04-p14360799
Bn-A04-p14357445
Bn-A04-p14630533
Bn-A04-p11957518
Bn-A04-p14661285
Bn-A04-p12010066
119,1
Bn-A04-p14670237
69,0
Bn-A04-p11923511
Bn-A04-p14807150
Bn-A04-p11900571
122,0
Bn-scaff_15585_1-p127973
69,6
Bn-A04-p12891881
124,0
Bn-A04-p14877869
70,2
Bn-A04-p12959668
Bn-A04-p14922562
128,7
Bn-A04-p13068545
71,5
Bn-Scaffold001532-p334
Bn-A04-p12251256
Bn-A04-p15296217
74,0
Bn-A04-p12256636
131,6
Bn-A04-p15399118
74,6
Bn-A04-p12344157
Bn-A04-p15409836
Bn-A04-p15514869
Bn-A04-p12037515
138,5
Bn-scaff_22091_1-p89053
145,2
Bn-A04-p15631388
76,4
Bn-A04-p13497608
Bn-A04-p15713429
Bn-A04-p13583325
Bn-A04-p15895510
152,3
80,3
Bn-A04-p13665489
Bn-A04-p16123695
82,0
Bn-A01-p14559770
157,0
Bn-A04-p16166839
83,4
Bn-A04-p13122589
Bn-A04-p16303678
Bn-A04-p13129093
159,0
Bn-A04-p16403131
Bn-A04-p13241959
Bn-A04-p16233024
87,1
Bn-A04-p13245778
161,4
Bn-A04-p16276464
Bn-A04-p13370433
Bn-A09-p32165581
163,6
Bn-A09-p30016810
Bn-A04-p16445675
Bn-A04-p13761897
167,8
Bn-A04-p16451992
89,0
Bn-A04-p13738279
168,3
Bn-A04-p16584084
Bn-A04-p13711282
168,9
Bn-A04-p16608584
Bn-A04-p14156506
172,8
91,5
Bn-A04-p16627363
Bn-A04-p14357445
173,6
Bn-A04-p16663552
92,2
Bn-A04-p14360799
174,3
Bn-A04-p17300068
Bn-A04-p14380140
Bn-A04-p17416243
176,0
Bn-A04-p14410667
Bn-A04-p17439839
Bn-A04-p14565138
177,2
Bn-A04-p17541623
97,9
Bn-A04-p14397957
178,3
Bn-A04-p17577700
Bn-A04-p14630533
186,6
Bn-A04-p17615088
Bn-A04-p16303678
Bn-A04-p17391802
Bn-A04-p16276464
205,9
Bn-A04-p17632009
98,9
Bn-A09-p32165581
Bn-A04-p17692138
99,9
Bn-A04-p16365226
208,3
Bn-A04-p17698780
Bn-A04-p16598514
101,0
208,8
Bn-A04-p17712184
Bn-A04-p16607433
Bn-A04-p17738097
101,7
Bn-A04-p16579551
209,3
Bn-A04-p17768404
Bn-A04-p16584084
103,4
211,3
Bn-A04-p17756059
Bn-A04-p17541623
104,2
218,2
Bn-A04-p17760280
Bn-A04-p17412823
105,6
219,6
Bn-A04-p17858149
Bn-A04-p17686228
220,9
Bn-A04-p17698780
Bn-A04-p18003170
106,6
223,9
Bn-A04-p17712184
Bn-A04-p18129552
Bn-A04-p17738097
225,6
Bn-A04-p18354325
107,5
Bn-A04-p17740663
227,8
Bn-A04-p18457550
Bn-scaff_20270_1-p792475
228,5
Bn-A04-p18562244
108,4
Bn-A04-p17756059
230,0
Bn-A04-p18577466
Bn-A04-p17768404
230,6
Bn-A01-p25183707
110,9
Ex*1012-98
A04
A04200564
0,0
A04839926
3,3
A041338227
A041342543
4,6
A04r150576
A021977941
11,1
A042534936
A042858685
13,5
A043016839
A04r263828
14,8
A04r268732
A04r269379
A043253401
A043271347
17,3
A043297129
A03r1969649het
21,1
C0227502652het
21,7
A045687201
A046810896
22,9
Annr19806872
A047743922
23,9
C0428186145PA
24,8
A049129999
25,3
A048943515
26,0
A04r390754hetEx
A0410211913
28,0
A049947883
28,4
A04r490263
28,7
A04r462315
29,1
A0410212638
29,4
A0411745881
A0411766578PA
32,9
A0411766912
A0716691607
A0411830026
33,5
A0412028144
A0412041691
A0412074005PA
34,7
A0412076899
A0412213828
36,3
A0412343654
39,7
A0412349788
40,4
A04r633544
A0412471834
41,6
A0412479834
A0412519684
42,2
A0412691145
Annr47972381
42,8
A0412780295
44,1
A0412792684
A0412923275
A0412970481
A0413028972
45,9
A0413103219
Annr15686911PA
Annr15702546
A0413137660
A0413198155
47,2
A0413237879
A0413245368
A0413402558PA
51,0
A0413326977
53,5
A0413577906PA
A0413648408
54,6
A0413880754
55,9
A0413894819
A0413877734
56,5
A0413850812
56,9
A0413761679
57,1
A04r859817
58,4
C0441504291
A0414148774
60,2
A0414160938
A0414254894
61,5
A0414381083
62,1
A0414401988PA
A0414687908
65,9
A0414853313
A0414873132
A0414881039
A0415005731
67,8
A04r1057790
A04r1061144
C0443620841
A0415132373
A0415141564
69,0
A0415257698
Cnnr57283641
69,6
A0415333203
70,2
A0415368678
71,5
A0415442136
A0415753637
74,0
A0415791897
74,6
A0415808130
A0415938732
A0416069880
76,4
Annr7363725het
A0416092513
80,3
A0416318675
82,0
A0416368214
83,4
A0416484408
A0416580390
A04r1139830
87,1
A04r1204398
A0815762659
A0416619242
A0416625563
89,0
C0445498974
A0416765402
91,5
C0445535992
A0416843246
92,2
A0417424310
A0417512833
A0417534681
A0417648688
97,9
A0417689690
A0417726222
Annr41786880
A0417741611
98,9
A0417801901
99,9
A0417808940
101,0
A0417822235
A0417847919
101,7
A0417877587
103,4
A0417865768
104,2
A0417869910
105,6
A0417966721
A0418079803
106,6
A0418214890
A0418424204
107,5
A0418546546
A0418693930
108,4
A0418709025
A04r1387330
110,9
Ex*V8
A04
A04620930
0,0
A04542168
3,8
A04462855
4,8
A04480973
A04104399
8,1
A04200564
A04r14963
9,0
A04r47447
11,7
A04r37417
12,1
A041037957
15,4
A042858685
22,8
A021977941
28,6
A042534936
A04r263828
A04r268732
31,5
A04r269379
A043587905
A043674724
35,2
A043748408
A043761542
A043271347
41,7
A043540219
A043016839
43,8
A043253401
46,2
A043297129
Cnnr17943249
51,3
A044443618
52,4
A044305768PA
55,0
A043864002
59,2
A044053269
A044364522
61,1
A046321517
62,2
A048885015
A048943515
65,9
A04r390754
A047754487
68,8
A047885464
A048001447
71,7
C0433343529
A04r377172
72,3
A049947883
A04r462315
77,6
A04r490263
A0410211913
82,7
A0410212638
A0411830026
88,2
A0716691607
A0411745881
92,2
A0411766578PA
92,8
A0411766912
A0412223051
95,9
A0412315824PA
97,4
C0438906149
101,2
A0412691145
103,3
A04r633544
103,9
A0412343654
104,4
A0412349788
A0412028144
A0412041691
A0412074005PA
106,9
A0412076899
A0412213828
A0412471834
111,1
A0412479834
A0412780295
114,9
Annr47972381
A0412923275
A0412970481
119,1
Annr15702546
Annr15686911PA
122,0
A0413577906PA
124,0
A0413648408
128,7
A0413761679
A0413237879
A0413245368
131,6
A0413326977
A0413015334PA
138,5
C0441686052
145,2
A0414160938
A0414254894
152,3
A0414308915
A0413825972PA
157,0
A0413880754
A0413894819
159,0
C0441504291
A04r859817
161,4
A0414038989
163,6
A0414148774
A0414401988PA
167,8
A0414381083
168,3
A0414351459
168,9
A0414687908
172,8
A04r1061144
173,6
A04r1057790
174,3
A0414853313
176,0
A0414881039
A0415005731
177,2
A0414873132
178,3
C0443620841
186,6
A0416484408
A04r1204398
205,9
A0815762659
A0416540801
208,3
A0416755284
208,8
A0416764252
A0416733534
209,3
C0445498974
211,3
A0417648688
218,2
A0417510910
219,6
A0417794982
220,9
A0417808940
223,9
A0417822235
A0417847919
225,6
A0417850498
227,8
C0446923075
228,5
A0417865768
230,0
A0417877587
230,6
Ex*R53
A04
A0415257698
A0415333203
0,0
Cnnr57283641
A0415442136
1,2
A0415938732
5,0
A0416069880
5,4
A0416092513
7,9
A0416318675
9,1
A0815762659
10,3
A04r1139830
A0416540801
13,2
A0416580390
A0416619242
15,7
A0416625563
A0416684039
16,9
A0416765402
18,2
C0445498974
C0445535992
18,6
A0416843246
19,9
A0417424310
22,4
Annr41786880
22,9
A0417512833
24,4
A0417534681
25,4
A0417689690
26,3
A0417726222
28,2
A0417808940
A0417741611
A0417847919
29,2
A0417801901
A0417822235
A0417966721
30,2
A0418079803
30,7
A0418214890
31,6
A0418251546
32,1
A0418424204
35,1
A0418693930
37,5
A0418709025
A0418546546
39,0
A0418746955
40,0
A0418745068
40,4

## Slide 5
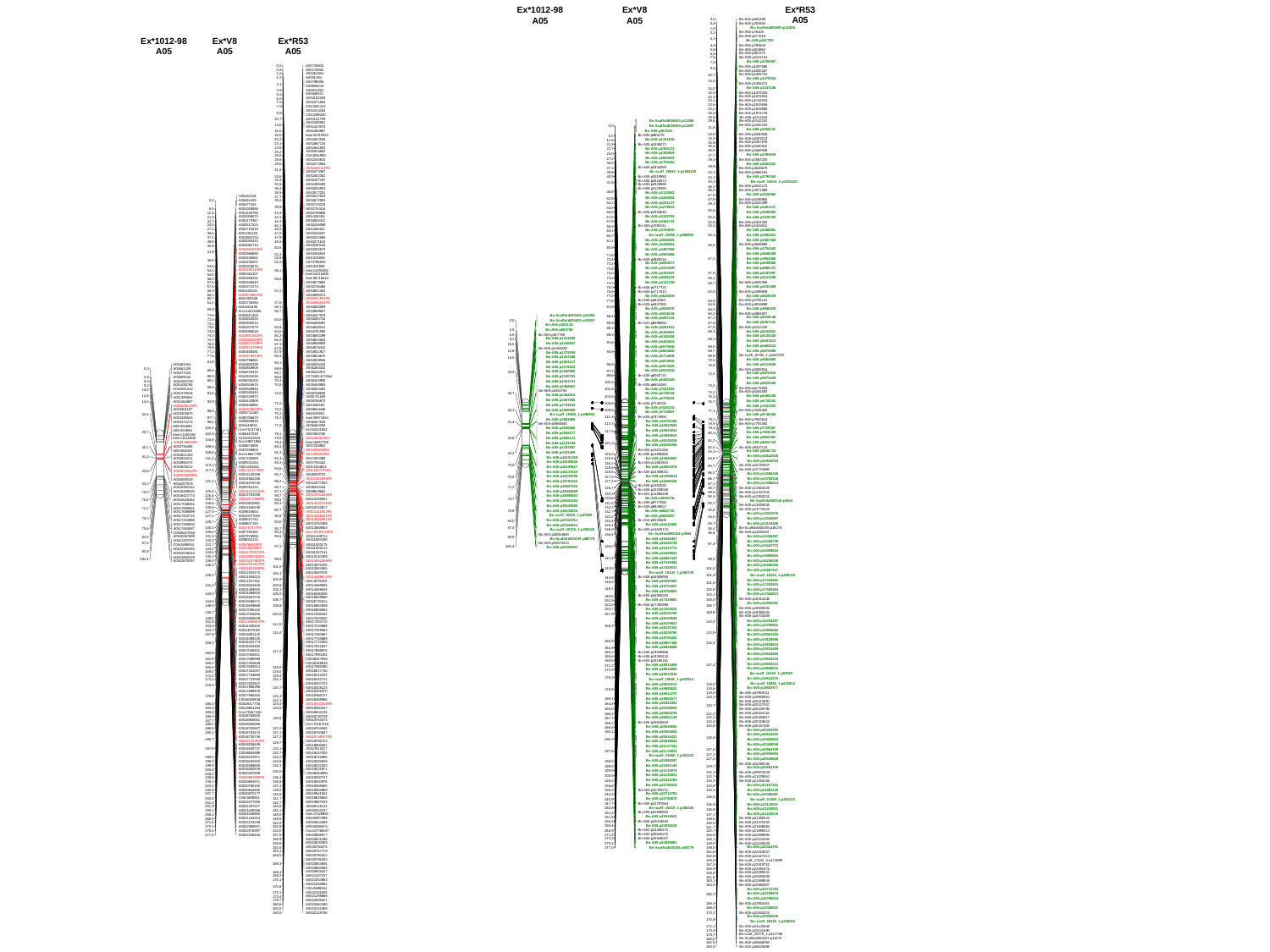

Ex*R53
A05
Bn-A05-p600196
0,0
Bn-A05-p253584
0,8
Bn-Scaffold000403-p12455
1,6
Bn-A05-p76428
2,2
Bn-A05-p677619
4,1
Bn-A05-p687790
Bn-A05-p780844
4,6
Bn-A05-p816963
5,6
Bn-A05-p967575
6,0
Bn-A05-p1134104
7,5
Bn-A05-p1196947
7,9
Bn-A05-p1097088
9,8
Bn-A05-p1035167
Bn-A05-p1006754
10,7
Bn-A05-p1279304
14,0
Bn-A05-p1308471
Bn-A05-p1347246
15,0
Bn-A05-p1479325
16,0
Bn-A05-p1676024
20,3
Bn-A05-p1744534
23,1
Bn-A05-p1818846
23,6
Bn-A05-p1919060
25,2
Bn-A05-p1974178
26,0
Bn-A05-p2111165
28,6
Bn-A05-p2142102
29,6
Bn-A05-p2345233
31,8
Bn-A05-p2350131
Bn-A05-p2280549
33,6
Bn-A05-p2303112
34,3
Bn-A05-p2367876
35,9
Bn-A05-p2440512
36,4
Bn-A05-p2469338
36,9
Bn-A05-p2394919
37,7
Bn-A05-p2563320
39,4
Bn-A05-p2604531
39,9
Bn-A05-p2648679
Bn-A05-p2688101
43,3
Bn-A05-p2794364
44,1
Bn-scaff_16534_1-p1394120
45,3
Bn-A05-p3042173
46,1
Bn-A05-p3071868
46,5
Bn-A05-p3130062
47,0
Bn-A05-p3198955
47,9
Bn-A05-p3242299
49,3
Bn-A05-p3251117
50,6
Bn-A05-p3280592
Bn-A05-p3320783
52,4
Bn-A05-p3401393
52,9
Bn-A05-p3429612
53,4
Bn-A05-p3456661
Bn-A05-p3482813
55,1
Bn-A05-p3467060
Bn-A05-p3649985
56,6
Bn-A05-p3754543
Bn-A05-p3846005
Bn-A05-p3953066
Bn-A05-p4036806
57,2
Bn-A05-p4085121
Bn-A05-p4197697
57,6
Bn-A05-p4121158
58,1
Bn-A05-p4093306
58,7
Bn-A05-p4333409
Bn-A05-p4399949
63,0
Bn-A05-p4625019
Bn-A05-p4794141
63,9
Bn-A05-p4919899
64,8
Bn-A05-p4940676
65,2
Bn-A05-p4984837
66,2
Bn-A05-p5038148
67,1
Bn-A05-p5057141
67,5
Bn-A05-p5102120
67,9
Bn-A05-p5104821
68,3
Bn-A05-p5135426
69,1
Bn-A05-p5251613
Bn-A05-p5483612
69,5
Bn-A05-p5575695
69,7
Bn-scaff_15705_1-p1533707
69,9
Bn-A05-p5684860
70,3
Bn-A05-p5714836
70,8
Bn-A05-p5838524
Bn-A05-p5970916
72,0
Bn-A05-p5973528
Bn-A05-p6020205
73,2
Bn-A05-p6176943
Bn-A05-p6284593
74,2
Bn-A05-p6480168
75,2
Bn-A05-p6739341
76,7
Bn-A05-p7031053
Bn-A05-p7038463
77,3
Bn-A05-p6748150
Bn-A05-p7552614
78,1
Bn-A05-p7791550
78,9
Bn-A05-p7729367
79,4
Bn-A05-p7826230
80,4
Bn-A05-p9602897
81,3
Bn-A05-p9602710
Bn-A05-p9347113
82,4
Bn-A05-p9646716
83,3
Bn-A05-p10622526
84,6
Bn-A05-p10625034
Bn-A05-p10783057
85,7
Bn-A05-p11776888
86,2
Bn-A05-p11856345
Bn-A05-p10752346
86,7
Bn-A05-p11088814
87,1
Bn-A05-p13853329
88,7
Bn-A05-p12447042
89,5
Bn-A05-p12930251
90,3
Bn-Scaffold000324-p3944
90,7
Bn-A05-p13000538
Bn-A05-p13778123
92,8
Bn-A05-p12021976
94,0
Bn-A05-p14042687
Bn-A05-p14144406
94,7
Bn-Scaffold000285-p18278
95,4
Bn-A05-p14592407
96,6
Bn-A05-p15426487
Bn-A05-p15446739
97,3
Bn-A05-p15447772
Bn-A05-p15459604
Bn-A05-p15894915
98,5
Bn-A05-p16245038
Bn-A05-p16260356
101,0
Bn-A05-p16867347
Bn-scaff_18140_1-p385729
101,4
Bn-A05-p17419591
101,8
Bn-A05-p17202622
Bn-A05-p17420484
102,5
Bn-A05-p17420613
103,1
Bn-A05-p18214148
106,0
Bn-A05-p18391581
106,7
Bn-A05-p18505935
Bn-A05-p18590326
108,8
Bn-A05-p18704009
Bn-A05-p18753257
110,0
Bn-A05-p18768801
Bn-A05-p18958083
112,5
Bn-A05-p19001293
Bn-A05-p19128595
115,4
Bn-A05-p19238534
Bn-A05-p19514459
Bn-A05-p19532660
Bn-A05-p19623018
Bn-A05-p19650213
117,4
Bn-A05-p19668632
Bn-scaff_16516_1-p57960
Bn-A05-p19612275
Bn-scaff_18826_1-p632914
118,0
Bn-A05-p19802577
118,6
Bn-A05-p19919111
119,4
Bn-A05-p19930910
120,1
Bn-A05-p20113630
Bn-A05-p20127547
120,7
Bn-A05-p20128796
Bn-A05-p20163118
121,3
Bn-A05-p20283817
122,1
Bn-A05-p20319622
124,4
Bn-A05-p20337405
125,6
Bn-A05-p20443393
Bn-A05-p20534431
126,8
Bn-A05-p20563653
Bn-A05-p20449058
Bn-A05-p20564729
127,0
Bn-A05-p20550694
127,1
Bn-A05-p20549826
127,2
Bn-A05-p22298146
129,7
Bn-A05-p20601139
Bn-A05-p20972649
132,1
Bn-A05-p21029910
132,7
Bn-A05-p21058156
133,2
Bn-A05-p21107181
133,8
Bn-A05-p21081145
134,3
Bn-A05-p21065087
135,0
Bn-scaff_21369_1-p381222
Bn-A05-p21176831
136,4
Bn-A05-p21243931
136,8
Bn-A05-p21210078
137,1
Bn-A05-p21385524
139,5
Bn-A05-p21370435
140,8
Bn-A05-p21646684
141,7
Bn-A05-p21699314
142,7
Bn-A05-p22089650
144,6
Bn-A05-p22141046
145,1
Bn-A05-p22182628
148,0
Bn-A05-p22324761
149,5
Bn-A05-p22454557
151,8
Bn-A05-p22427412
152,9
Bn-scaff_17441_3-p173899
154,0
Bn-A05-p22519751
157,5
Bn-A05-p22591072
158,9
Bn-A05-p22599241
159,8
Bn-A05-p22682639
161,6
Bn-A05-p22689848
163,1
Bn-A05-p22682587
164,5
Bn-A05-p22712783
Bn-A05-p22780670
166,3
Bn-A05-p22789344
Bn-A05-p22853415
168,4
Bn-A05-p23044521
169,2
Bn-A05-p22943215
170,1
Bn-A05-p22938228
170,8
Bn-scaff_20219_1-p308326
Bn-A05-p23143046
172,1
Bn-A05-p23141080
173,4
Bn-scaff_20376_1-p121796
179,7
Bn-Scaffold000191-p11670
180,6
Bn-A02-p26658350
182,5
Bn-A02-p26625696
183,0
Ex*1012-98
A05
Bn-Scaffold000403-p12455
Bn-Scaffold000403-p12697
0,0
Bn-A05-p363138
Bn-A05-p687790
3,5
Bn-A05-p917769
5,5
Bn-A05-p1311652
9,2
Bn-A05-p1196947
10,5
Bn-A05-p1260228
12,6
Bn-A05-p1279304
Bn-A05-p1347246
14,5
Bn-A05-p3251117
Bn-A05-p3278023
Bn-A05-p3280592
34,4
Bn-A05-p3320783
Bn-A05-p3361741
Bn-A05-p3456661
Bn-A05-p3454754
35,7
Bn-A05-p3482813
Bn-A05-p3467060
Bn-A05-p3754543
Bn-A05-p3846005
40,1
Bn-scaff_15908_1-p498900
Bn-A05-p3953066
41,4
Bn-A05-p3966848
Bn-A05-p4036806
Bn-A05-p4068277
Bn-A05-p4085121
42,6
Bn-A05-p4121158
Bn-A05-p4197697
Bn-A05-p4333409
43,2
Bn-A05-p18101769
Bn-A05-p18109536
70,0
Bn-A05-p18109527
Bn-A05-p18137410
Bn-A05-p18229790
70,6
Bn-A05-p18793322
Bn-A05-p18807426
71,2
Bn-A05-p18828589
Bn-A05-p18958083
73,7
Bn-A05-p19001293
Bn-A05-p19128595
Bn-A05-p19238534
75,6
Bn-scaff_16516_1-p57960
Bn-A05-p22324761
84,0
Bn-A05-p23044521
87,4
Bn-scaff_20219_1-p308326
Bn-A02-p26854880
93,9
Bn-Scaffold000191-p89779
Bn-A05-p23873413
100,4
Bn-A05-p23855991
Ex*V8
A05
Bn-Scaffold000403-p12455
Bn-Scaffold000403-p12697
0,0
Bn-A05-p363138
Bn-A05-p895276
6,5
Bn-A05-p1311652
11,6
Bn-A05-p1898273
21,3
Bn-A05-p2350131
22,7
Bn-A05-p2394919
24,0
Bn-A05-p2604531
27,2
Bn-A05-p2794364
36,6
Bn-A05-p2844059
37,1
Bn-scaff_16534_1-p1394120
39,8
Bn-A05-p3019980
40,9
Bn-A05-p2924872
41,9
Bn-A05-p2929698
Bn-A05-p3129492
48,8
Bn-A05-p3130062
Bn-A05-p3280592
53,8
Bn-A05-p3251117
54,2
Bn-A05-p3278023
54,5
Bn-A05-p3330640
56,5
Bn-A05-p3320783
57,0
Bn-A05-p3361741
57,5
Bn-A05-p3394311
58,3
Bn-A05-p3754543
60,1
Bn-scaff_15908_1-p498900
60,7
Bn-A05-p3846005
61,1
Bn-A05-p3456661
65,9
Bn-A05-p3467060
Bn-A05-p3953066
71,6
Bn-A05-p3988218
72,4
Bn-A05-p4068277
73,1
Bn-A05-p4333409
73,6
Bn-A05-p4197697
74,0
Bn-A05-p4085121
74,3
Bn-A05-p4121158
74,7
Bn-A05-p4717725
76,0
Bn-A05-p4717815
76,8
Bn-A05-p4625019
77,2
Bn-A05-p4632407
77,6
Bn-A05-p4937055
81,8
Bn-A05-p4940676
Bn-A05-p5038148
86,4
Bn-A05-p5057141
Bn-A01-p8596650
88,9
Bn-A05-p5251613
89,1
Bn-A05-p5104821
89,3
Bn-A05-p5135426
Bn-A05-p5483612
91,6
Bn-A05-p5575695
Bn-A05-p5684860
93,9
Bn-A05-p5714836
Bn-A05-p5970916
96,8
Bn-A05-p5973528
Bn-A05-p6020205
97,7
Bn-A05-p6063715
98,6
Bn-A05-p6480168
100,4
Bn-A05-p6654285
Bn-A05-p7031053
102,8
Bn-A05-p6748150
104,6
Bn-A05-p6739341
Bn-A05-p7246705
108,6
Bn-A05-p7826230
109,6
Bn-A05-p7729367
Bn-A05-p7874994
111,4
Bn-A05-p10752346
113,3
Bn-A05-p10622526
Bn-A05-p10625034
117,5
Bn-A05-p15894915
Bn-A05-p16245038
121,2
Bn-A05-p16260356
Bn-A05-p11151246
Bn-A05-p14890882
125,0
Bn-A05-p14042687
125,6
Bn-A05-p11961924
126,1
Bn-A05-p12021976
126,6
Bn-A05-p15369031
126,8
Bn-A05-p11088814
127,0
Bn-A05-p11856345
127,2
Bn-A08-p1382630
128,7
Bn-A05-p10269046
Bn-A01-p11996408
130,3
Bn-A05-p9646716
130,6
Bn-A05-p9777066
131,5
Bn-A05-p9849822
132,2
Bn-A05-p9602710
132,7
Bn-A05-p9602897
133,2
Bn-A10-p5525609
134,4
Bn-A05-p14144406
135,2
Bn-A05-p11626174
136,0
Bn-Scaffold000324-p3944
136,4
Bn-A05-p15426487
Bn-A05-p15446739
138,0
Bn-A05-p15447772
Bn-A05-p15459604
Bn-A05-p16867347
141,4
Bn-A05-p17420484
Bn-A05-p17420613
143,0
Bn-scaff_18140_1-p385729
Bn-A05-p16909956
144,6
Bn-A05-p18391581
146,0
Bn-A05-p18753257
146,7
Bn-A05-p18768801
Bn-A05-p16938263
149,5
Bn-A05-p17419591
151,8
Bn-A05-p17392998
153,0
Bn-A05-p17202622
153,7
Bn-A05-p18101769
157,9
Bn-A05-p18109536
Bn-A05-p18109527
158,3
Bn-A05-p18137410
Bn-A05-p18229790
Bn-A05-p18793322
160,8
Bn-A05-p18807426
Bn-A05-p18828589
161,9
Bn-A05-p19209866
165,1
Bn-A05-p19359433
165,6
Bn-A05-p19195181
169,5
Bn-A05-p19514459
172,2
Bn-A05-p19532660
173,2
Bn-A05-p19623018
176,3
Bn-scaff_18826_1-p632914
Bn-A05-p19650213
Bn-A05-p19668632
178,8
Bn-A05-p19612275
Bn-A05-p19802577
180,3
Bn-A05-p20443393
184,4
Bn-A05-p20449058
185,0
Bn-A05-p20564729
186,4
Bn-A05-p20601139
187,7
Bn-A05-p20626818
188,2
Bn-A05-p20550694
188,8
Bn-A05-p20563653
190,1
Bn-A05-p20534431
190,7
Bn-A05-p20549826
Bn-A05-p21107181
Bn-A05-p21176831
197,6
Bn-scaff_21369_1-p381222
Bn-A05-p21065087
198,5
Bn-A05-p21081145
199,0
Bn-A05-p21210078
199,9
Bn-A05-p21243931
200,8
Bn-A05-p22324761
228,2
Bn-A05-p22789344
236,6
Bn-A05-p22789711
238,2
Bn-A05-p22712783
243,2
Bn-A05-p22780670
245,9
Bn-A05-p22797664
247,7
Bn-scaff_20219_1-p308326
250,8
Bn-A05-p22969550
251,4
Bn-A05-p23044521
251,9
Bn-A05-p23019620
255,2
Bn-A05-p22938228
256,4
Bn-A02-p23280472
260,9
Bn-A02-p26640222
271,8
Bn-A05-p23848037
273,3
Bn-A05-p23855991
275,4
Bn-Scaffold000191-p89779
277,0
Ex*1012-98
A05
Ex*V8
A05
Ex*R53
A05
A05720802
0,0
A05270865
0,8
A05461184
1,6
A0591392
2,2
A05799088
4,1
A05809244
A05913353
4,6
A05949321
5,6
A051102236
6,0
A051271943
7,5
C041591412
7,9
A051234154
9,8
C041299250
A051151799
10,7
A051395561
14,0
A051423575
A051464867
15,0
Annr32315053
16,0
A051823545
20,3
A051887134
23,1
A051941091
23,6
A052024603
25,2
C042554990
26,0
A052240815
28,6
A052273504
29,6
A052468112PA
31,8
A052473367
A052402352
33,6
A052427197
34,3
A052490459
35,9
A052554522
36,4
A052577251
36,9
A052517523
37,7
A052672991
39,4
A052715218
39,9
A052752416
A052798808
43,3
A05r192155
44,1
A053055412
45,3
A053106089
46,1
A05r284322
46,5
A053156207
47,0
A053222565
47,9
A053271162
49,3
A053280144
50,6
A053303675
A053346543
52,4
A05r333059
52,9
C073798925
53,4
A05r351690
Annr14191592
55,1
Annr14215406
Annr36716610
56,6
A053670692
A053736494
A053821303
A053895474
A05461184
A05461428
0,0
A05477324
A051028686
6,5
A051426794
11,6
A052006072
21,3
A052473367
22,7
A052517523
24,0
A052715218
27,2
A05r192155
36,6
A052904734
37,1
A053055412
39,8
A053082710
40,9
A052981825PA
41,9
A052986695
A053155661
48,8
A053156207
A053303675
53,8
A053280144PA
54,2
A053301107
54,5
A053356331
56,5
A053346543
57,0
A053372274
57,5
A05r340101
58,3
A053670692PA
60,1
A05r393188
60,7
A053736494
61,1
A05r351690
65,9
Annr14215406
A053821303
71,6
A053853825
72,4
A053929212
73,1
A054207579
73,6
A054066519
74,0
A053951562PA
74,3
A054000089PA
74,7
A054574709PA
76,0
A054574799PA
76,8
A054468491
77,2
A054478433PA
77,6
A054796651
81,8
A054800399
A054858909
86,4
A054876415
A042401616
88,9
A055036103
89,1
A054924675
89,3
A054949946
A055260443
91,6
A055325972
A055433909
93,9
A055459983
A055708838PA
96,8
A055711169
A055766873
97,7
A055808916
98,6
57,2
A053951562PA
A054066519PA
57,6
A054000089
58,1
A053959827
58,7
A054207579
A054265716
63,0
A054468491
A054648214
63,9
A054781592
64,8
A054800399
65,2
A054815645
66,2
A054858909
67,1
A054876415
67,5
A054921917
67,9
A054924675
68,3
A054949946
69,1
A055036103
A055260443
69,5
A055325972
69,7
C0734824376het
69,9
A055433909
70,3
A055459983
70,8
A055563282
A055708838
72,0
A055711169
A055766873
73,2
A05r569162
A055844448
74,2
A05r619051
75,2
Annr39971854
76,7
A056457049
A056464294
77,3
A1010431924
A057060766
78,1
A05r848353PA
78,9
Annr16657759
79,4
A057206803
80,4
A05r969296PA
81,3
A05r969483PA
A057403358
82,4
A057750162
83,3
A05r1104811
84,6
A05r1107275PA
A059359723
85,7
A0510311089PA
86,2
A0510377052
A059333154
86,7
A059819654
87,1
A0513591268PA
88,7
A0512438921
89,5
A0514020745PA
90,3
A0510703617
90,7
A0514111802PA
A0513518401PA
92,8
A0510832881PA
94,0
A0513783289
A0513888653
94,7
A05461184
A05461428
0,0
A05477324
A05809244
3,5
A051050739
5,5
A051426794
9,2
C041591412
10,5
A051376516
12,6
A051395561
A051464867
14,5
A053280144PA
A053301107
A053303675
34,4
A053346543
A053372274
A05r619051
100,4
Cnnr70747351
A056457049
102,8
A1010431924
104,6
Annr39971854
A056670898
108,6
A057206803
109,6
Annr16657759
A057245889
111,4
A059333154
113,3
A05r1104811
A05r1107275PA
117,5
A0512140359
A0514962268
121,2
A0514975235
A059762134
A0511422312PA
125,0
A0513783289
125,6
A0510772808PA
126,1
A0510832881
126,6
A0511840238
126,8
A059819654
127,0
A0510377052
127,2
A058521742
128,7
A058837153
A05r787477PA
130,3
A057750162
130,6
A057976959
131,5
A058052132
132,2
A05r969483PA
132,7
A05r969296PA
133,2
A0511793247PA
134,4
A0513888653PA
135,2
A0510157901PA
136,0
A0510703617PA
136,4
A0511907488PA
A0511933175
138,0
A0511934213
A0511937161
A0515563455
141,4
A0514469505
A0514469634
143,0
A0515587576
A0515586271
144,6
A0516659668
146,0
A0517006162
146,7
A0517025635
A0515606529
149,5
A0514468612PA
151,8
A0514435442
153,0
A05r1670159
153,7
A0516381142
157,9
A0516389220
A0516423774
158,3
A0516520462
A0517048451
160,8
A0517065821
A0517086999
161,9
A0517453639
165,1
A0517605513
165,6
A05r351690
A05r353568
35,7
Annr14191592
Annr14215406
A053670692PA
A053736494
40,1
A05r393188
A053821303
41,4
A053834211
A053895474
A053929212
A053951562PA
42,6
A054000089PA
A054066519
A054207579
43,2
A0516381142
A0516389220
70,0
Annr16385754PA
95,4
A0511189714
96,6
A0511907488
A0511933175
97,3
A0511934213
A0511937161
A0512140359
98,5
A0514962268PA
A0514975235
101,0
A0515563455
A0515587576
101,4
A0514468612PA
101,8
A05r1670159
A0514469505
102,5
A0514469634
103,1
A0516490538
106,0
A0516659668
106,7
A0516781811
A0516862858
108,8
A0516969064
A0517006162
110,0
A0517025635
A0517203715
112,5
A0517234988
A0517359503
115,4
A0517484587
A0517704669
A0517723350
A0517822847
A0517869976
117,4
A0517890204
C0536027650
C0536348916
A0517986390
118,0
A0518017730
118,6
A0518142243
119,4
A0518152712
120,1
A0518307723
A0518328123
120,7
A0518329370
A0518366727
121,3
A0518489956
122,1
A0518531816PA
124,4
A0518550347
125,6
A0518651194
A0518730739
126,8
A0518763474
Cnnr70167416
A0518764550
127,0
A0518750607
127,1
A0518749707PA
127,2
A0518796723
129,7
A0518805551
A0519111527
132,1
A0519187925
132,7
A0519215988
133,2
A0519255639
133,8
A0519232253
134,3
A0519222971
135,0
C0538564890
A0519330737
136,4
A0519402976
136,8
A0519369605
137,1
A0519554969
139,5
A0519541534
140,8
A0516423774
A0516520462
70,6
A0517048451
A0517065821
71,2
A0517086999
A0517203715
73,7
A0517234988
A0517359503
A0517484587
75,6
C0536027650
A0520387699
84,0
A0521207237
87,4
C05r3699924
A0522381945
93,9
A0522536444
A0522850229
100,4
A0522876397
A0517441037
169,5
A0517704669
172,2
A0517723350
173,2
A0517822847
176,3
A0517986390
A0517869976
A0517890204
178,8
C0536348916
A0518017730
180,3
A0518651194
184,4
Cnnr70167416
185,0
A0518764550
186,4
A0518805551
187,7
A0518830588
188,2
A0518750607
188,8
A0518763474
190,1
A0518730739
190,7
A0518749707PA
A0519255639
A0519330737
197,6
C0538564890
A0519222971
198,5
A0519232253
199,0
A0519369605
199,9
A0519402976
200,8
A0520387699
228,2
A0520863605PA
236,6
A0520863971
238,2
A0520786182
243,2
A0520854945
245,9
A0520872177
247,7
C05r3699924
A0519829926
250,8
141,7
A0521077005
A0519887033
251,4
142,7
A0521207237
A0520211014
251,9
144,6
A0521180556
A0520252157
255,2
145,1
A0521048892
Annr17628692
256,4
148,0
A0521444314
A0520387699
260,9
149,5
A0522133249
271,8
A0520524269
151,8
A0522885557
A0520498474
273,3
152,9
A0522876397
Cnnr22736647
275,4
154,0
A0522536444
A0520558577
277,0
157,5
A0520621296
158,9
A0520630559
159,8
A0520760474
161,6
A0520767719
163,1
A0520760422
164,5
A0520786182
A0520854945
166,3
A0520863605
A0520926157
168,4
A0521207237
169,2
A0521053903
170,1
A0521048892
170,8
C05r3699924
A0521301835
172,1
A0521299868
173,4
A0522008227
179,7
A0522464190
180,6
A0522151069
182,5
A0522118788
183,0

## Slide 6
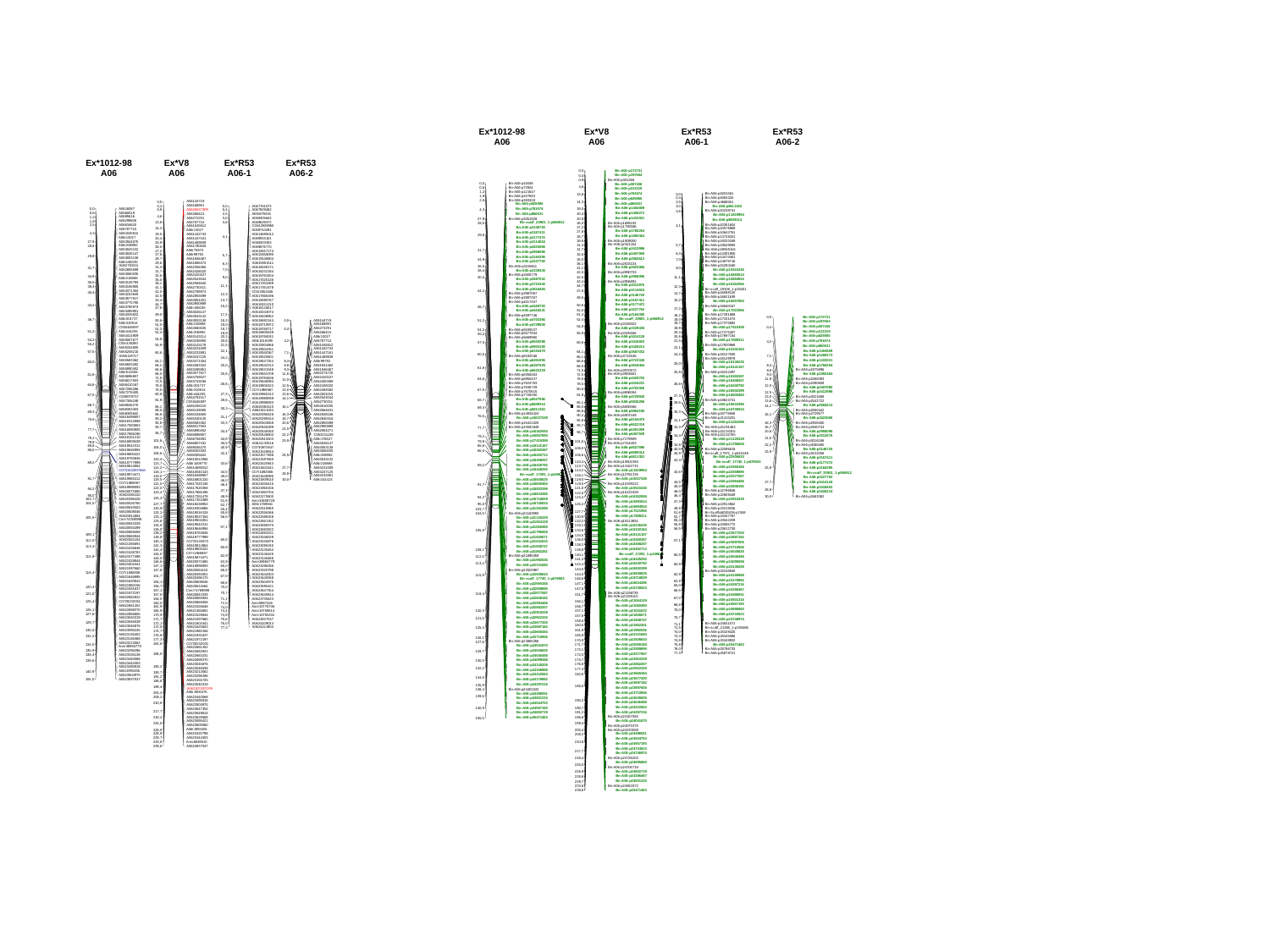

Ex*1012-98
A06
Ex*V8
A06
Ex*R53
A06-1
Ex*R53
A06-2
Bn-A06-p172731
0,0
Bn-A06-p197604
0,4
Bn-A06-p301366
0,9
Bn-A06-p307436
4,6
Bn-A06-p313319
Bn-A06-p781674
13,8
Bn-A06-p825900
14,3
Bn-A06-p860631
Bn-A06-p1404859
19,5
Bn-A06-p1408273
20,4
Bn-A06-p1433021
20,9
Bn-A06-p1685192
26,0
Bn-A06-p1793586
27,2
Bn-A06-p1768254
27,8
Bn-A06-p1892464
28,7
Bn-A06-p1909050
29,6
Bn-A06-p2341154
31,3
Bn-A06-p2412998
31,7
Bn-A06-p2407080
32,6
Bn-A06-p2582613
35,0
Bn-A06-p2633224
36,1
Bn-A06-p2823066
41,1
Bn-A06-p2890703
42,0
Bn-A06-p2958095
42,5
Bn-A06-p2956891
43,4
Bn-A06-p3012876
44,7
Bn-A06-p3114818
47,6
Bn-A06-p3146720
49,0
Bn-A06-p3167411
Bn-A06-p3177472
50,6
Bn-A06-p3227752
51,0
Bn-A06-p3184395
51,3
Bn-scaff_20901_1-p566912
52,4
Bn-A06-p3325502
54,8
Bn-A06-p3339435
Bn-A06-p3325566
55,9
Bn-A06-p3414128
Bn-A06-p3416803
60,6
Bn-A06-p3428214
Bn-A06-p3587032
64,1
Bn-A06-p3712646
66,1
Bn-A06-p3723340
66,6
Bn-A06-p3818484
68,5
Bn-A06-p3970972
71,5
Bn-A06-p3953661
72,5
Bn-A06-p4428703
78,0
Bn-A06-p4418231
78,5
Bn-A06-p4703366
80,8
Bn-A06-p4896084
81,9
Bn-A06-p4729500
Bn-A06-p5830298
85,1
Bn-A06-p5690066
86,0
Bn-A06-p5904190
88,8
Bn-A06-p5907449
90,2
Bn-A06-p15048
0,0
Bn-A06-p73924
0,6
Bn-A06-p121547
1,2
Bn-A06-p337583
1,9
Bn-A06-p393328
2,5
Bn-A06-p825900
Bn-A06-p781674
4,3
Bn-A06-p860631
Bn-A06-p3051928
27,9
Bn-scaff_20901_1-p566912
28,5
Bn-A06-p3146720
Bn-A06-p3167411
29,8
Bn-A06-p3177472
Bn-A06-p3114818
Bn-A06-p2823066
31,7
Bn-A06-p2958095
Bn-A06-p3184395
34,9
Bn-A06-p3227752
Bn-A06-p3318611
36,5
Bn-A06-p3339435
38,4
Bn-A06-p3405779
40,4
Bn-A06-p3587032
Bn-A06-p3723340
Bn-A06-p3818484
44,2
Bn-A06-p3987067
Bn-A06-p3997307
Bn-A06-p4517457
Bn-A06-p4428703
46,7
Bn-A06-p4418231
Bn-A06-p4597746
Bn-A06-p4703366
51,2
Bn-A06-p4729500
Bn-A06-p5535537
54,2
Bn-A06-p5577934
55,2
Bn-A06-p5669960
Bn-A06-p5830298
57,5
Bn-A06-p5904190
Bn-A06-p6154470
60,0
Bn-A06-p6182046
Bn-A06-p6451055
Bn-A06-p6457529
61,8
Bn-A06-p6622319
Bn-A06-p6356463
Bn-A06-p6955437
64,6
Bn-A06-p7624783
Bn-A06-p7636729
67,5
Bn-A06-p7670615
Bn-A06-p7726350
Bn-A06-p8527596
68,7
Bn-A06-p8809314
69,3
Bn-A06-p8811363
Bn-A06-p14834424
76,5
Bn-A06-p15037349
Bn-A06-p15421020
Bn-A06-p15581849
77,7
Bn-A06-p16362555
Bn-A06-p16657894
79,1
Bn-A06-p17415359
79,9
Bn-A06-p18141167
85,8
Bn-A06-p18260387
86,5
Bn-A06-p18402713
Bn-A06-p18408257
Bn-A06-p18430792
89,2
Bn-A06-p18445250
Bn-scaff_17291_1-p249622
Bn-A06-p18500625
Bn-A06-p18600460
91,7
Bn-A06-p18502399
Bn-A06-p18634396
94,2
Bn-A06-p18718829
Bn-A06-p18740524
95,0
Bn-A06-p21281098
103,7
Bn-A06-p8201581
0,0
Bn-A06-p8263325
0,4
Bn-A08-p4668561
2,5
Bn-A06-p8811363
3,0
Bn-A06-p10238741
3,6
Bn-A06-p11819954
Bn-A06-p8809314
Bn-A06-p10351604
4,1
Bn-A06-p10578969
Bn-A06-p10642761
Bn-A06-p13728321
Bn-A06-p19333349
Bn-A06-p19549093
5,7
Bn-A06-p19555116
Bn-A06-p14391905
6,3
Bn-A06-p14474461
7,0
Bn-A06-p14676716
Bn-A06-p15294160
9,0
Bn-A06-p15515345
Bn-A06-p15893014
11,1
Bn-A06-p15894910
Bn-A06-p16362555
12,3
Bn-scaff_19109_1-p33401
Bn-A06-p16588120
13,7
Bn-A06-p16621639
15,2
Bn-A06-p16657894
Bn-A06-p16840167
17,2
Bn-A06-p17021956
Bn-A06-p17291868
18,2
Bn-A06-p17331074
19,0
Bn-A06-p17375882
19,7
Bn-A06-p17415359
19,9
Bn-A06-p17375407
20,3
Bn-A06-p17697154
20,8
Bn-A06-p17889311
21,5
Bn-A06-p17920968
22,1
Bn-A06-p18102164
Bn-A06-p18127830
24,2
Bn-A06-p18129979
Bn-A06-p18136235
25,0
Bn-A06-p18141167
Bn-A06-p18152497
25,8
Bn-A06-p18260387
Bn-A06-p18408257
26,6
Bn-A06-p18430792
Bn-A06-p18502399
Bn-A06-p18600460
27,3
Bn-A06-p18624741
28,6
Bn-A06-p18634396
Bn-A06-p18740524
30,3
Bn-A06-p18776948
Bn-A06-p21415201
31,1
Bn-A06-p21281098
Bn-A06-p21191463
33,3
Bn-A06-p21134315
Bn-A06-p21132765
34,4
Bn-A06-p21126329
Bn-A06-p21796008
34,8
Bn-A06-p22096615
40,5
Bn-scaff_17972_1-p610149
40,9
Bn-A06-p22505810
42,4
Bn-scaff_17740_1-p676503
Bn-A06-p22555168
Bn-A06-p22559895
43,6
Bn-A06-p22577587
Bn-A06-p22594488
44,5
Bn-A06-p172731
0,0
Bn-A06-p197604
Bn-A06-p307436
0,4
Bn-A06-p313319
Bn-A06-p825900
Bn-A06-p781674
4,2
Bn-A06-p860631
Bn-A06-p1404859
Bn-A06-p1408273
7,1
Bn-A06-p1433021
Bn-A06-p1768254
8,4
Bn-A06-p1875896
8,8
Bn-A06-p1892464
9,3
Bn-A06-p2268393
11,6
Bn-A06-p2390609
12,0
Bn-A06-p2407080
Bn-A06-p2412998
12,5
Bn-A06-p2521668
13,0
Bn-A06-p2542722
13,6
Bn-A06-p2582613
14,1
Bn-A06-p2650442
Bn-A06-p2729577
16,1
Bn-A06-p6154470
92,8
Bn-A06-p6622319
93,7
Bn-A06-p6451055
94,7
Bn-A06-p6457529
Bn-A06-p7279909
101,6
Bn-A06-p7314403
Bn-A06-p8527596
106,0
Bn-A06-p8809314
106,6
Bn-A06-p8811363
Bn-A06-p19501594
110,4
Bn-A06-p14163715
113,7
Bn-A06-p11819954
114,2
Bn-A06-p10781335
116,1
Bn-A06-p15037349
119,0
Bn-A06-p15339222
119,5
Bn-A06-p15515345
121,0
Bn-A06-p16133328
122,0
Bn-A06-p16362555
123,4
Bn-A06-p15893014
125,3
Bn-A06-p15894910
Bn-A06-p17021956
127,7
Bn-A06-p17889311
130,9
Bn-A06-p18113924
132,2
Bn-A06-p18136235
133,1
Bn-A06-p18102164
133,6
Bn-A06-p18141167
134,5
Bn-A06-p18260387
136,0
Bn-A06-p18408257
138,2
Bn-A06-p18402713
138,8
Bn-scaff_17291_1-p249622
140,1
Bn-A06-p18445250
141,3
Bn-A06-p18430792
142,4
Bn-A06-p18502399
144,0
Bn-A06-p18500625
144,5
Bn-A06-p18718829
146,6
Bn-A06-p18634396
147,1
Bn-A06-p18740524
147,6
Bn-A06-p21196739
151,7
Bn-A06-p21195115
Bn-A06-p21064129
156,1
Bn-A06-p21060490
156,7
Bn-A06-p21041632
157,1
Bn-A06-p21826671
157,6
Bn-A06-p21848747
158,5
Bn-A06-p21852261
160,5
Bn-A06-p21992536
161,9
Bn-A06-p22331680
165,9
Bn-A06-p22505810
170,9
Bn-A06-p22555168
171,7
Bn-A06-p22559895
172,1
Bn-A06-p22577587
172,5
Bn-A06-p22910339
174,7
Bn-A06-p2823066
Bn-A06-p2938450
18,3
Bn-A06-p2940743
18,7
Bn-A06-p2958095
20,6
Bn-A06-p3012876
21,0
Bn-A06-p3016186
Bn-A06-p3088460
22,2
Bn-A06-p3146720
23,9
Bn-A06-p3132058
Bn-A06-p3167411
Bn-A06-p3177472
Bn-A06-p3184395
25,9
Bn-scaff_20901_1-p566912
Bn-A06-p3227752
Bn-A06-p3414128
27,7
Bn-A06-p22608192
45,0
Bn-A06-p3416803
28,8
Bn-A06-p22786948
46,0
Bn-A06-p3428214
Bn-A06-p22809449
46,4
Bn-A06-p3483382
30,0
Bn-A06-p22910339
47,3
Bn-A06-p22913862
Bn-A06-p23219336
49,9
Bn-Scaffold000436-p7458
51,8
Bn-A06-p21163993
104,5
Bn-A06-p23347767
52,7
Bn-A06-p21126329
Bn-A06-p23542209
55,4
Bn-A06-p21064129
Bn-A06-p23585772
55,8
Bn-A06-p21060490
Bn-A06-p23612730
56,5
105,9
Bn-A06-p21796008
Bn-A06-p23677200
Bn-A06-p21826671
Bn-A06-p23697182
57,1
Bn-A06-p21041632
Bn-A06-p23697605
Bn-A06-p21848747
Bn-A06-p23710506
109,1
Bn-A06-p21852261
Bn-A06-p24045825
60,5
Bn-A06-p21896359
112,0
Bn-A06-p24046488
Bn-A06-p21992536
Bn-A06-p24099288
113,4
60,9
Bn-A06-p22331680
Bn-A06-p24145208
Bn-A06-p22503987
Bn-A06-p24156940
Bn-A06-p22505810
62,9
115,9
Bn-A06-p24156988
Bn-scaff_17740_1-p676503
Bn-A06-p24178982
63,9
Bn-A06-p22555168
Bn-A06-p24297216
65,0
Bn-A06-p22559895
Bn-A06-p24386467
66,5
Bn-A06-p22577587
118,4
Bn-A06-p24496051
67,0
Bn-A06-p22608192
Bn-A06-p24501315
Bn-A06-p22594488
Bn-A06-p24557193
68,6
Bn-A06-p22882207
Bn-A06-p24695860
70,0
120,3
Bn-A06-p22910339
Bn-A06-p24744523
70,7
Bn-A06-p22922325
121,0
Bn-A06-p24746974
Bn-A06-p23677200
Bn-A06-p24831071
71,1
Bn-A06-p23697182
125,4
Bn-scaff_21268_1-p193486
71,5
Bn-A06-p23665384
Bn-A06-p25329425
74,0
Bn-A06-p25345586
74,4
Bn-A06-p23710506
126,1
Bn-A06-p25349932
74,8
Bn-A06-p23865356
127,6
Bn-A06-p25471463
75,6
Bn-A06-p24041870
Bn-A06-p25786733
76,0
Bn-A06-p24045825
129,7
Bn-A06-p25878721
77,3
Bn-A06-p24046488
Bn-A06-p24099288
130,5
Bn-A06-p22882207
176,8
Bn-A06-p24145208
132,2
Bn-A06-p22922325
177,3
Bn-A06-p24156988
Bn-A06-p23665384
182,6
Bn-A06-p24222064
134,0
Bn-A06-p23677200
Bn-A06-p24178982
Bn-A06-p23697182
Bn-A06-p24297216
135,9
186,0
Bn-A06-p23697605
Bn-A06-p24401543
138,4
Bn-A06-p23710506
Bn-A06-p24496051
139,6
Bn-A06-p24045825
Bn-A06-p24501315
190,2
Bn-A06-p24046488
Bn-A06-p24544753
Bn-A06-p24222064
Bn-A06-p24557193
190,7
140,9
Bn-A06-p24297216
Bn-A06-p24602719
191,2
Bn-A06-p24167592
196,8
Bn-A06-p25471463
155,0
Bn-A06-p24041870
199,4
Bn-A06-p24075375
Bn-A06-p24370009
205,4
Bn-A06-p24496051
208,2
Bn-A06-p24544753
210,8
Bn-A06-p24557193
Bn-A06-p24744523
217,7
Bn-A06-p24746974
Bn-A06-p24726203
218,4
Bn-A06-p24695860
225,0
Bn-A06-p24705718
Bn-A06-p24602719
226,9
Bn-A06-p24386467
228,8
Bn-A06-p24501315
229,7
Bn-A06-p24922072
233,8
Bn-A06-p25471463
239,6
Ex*1012-98
A06
A0616087
0,0
A0660019
0,6
A0685616
1,2
A06299826
1,9
A06834620
2,5
A06787710
A061045812
4,3
A06r13027
A062944875
27,9
A06r208992
28,5
A063043132
A063026147
29,8
A063053138
A06r165035
A062730311
31,7
A062855599
A063060035
34,9
A06r218888
A063135793
36,5
A063156905
38,4
A063371304
40,4
A063222645
A063577817
A063775795
44,2
A063785973
A063495951
A064335822
A06r301737
46,7
A06r310914
C055446597
A06r444255
51,2
A064414909
A065007677
54,2
C05r428280
55,2
A065334805
A065258210
57,5
A065119717
A065583362
60,0
A065603282
A065890452
A06r512506
61,8
A065896857
A066017553
A066410167
64,6
A067266286
A067278405
67,5
C059079757
A067306246
A068046270
68,7
A068303303
69,3
A068305642
A0616498857
76,5
A0616312688
A0617083863
A0616890805
77,7
A0617866196
A0618151210
79,1
A0618809439
79,9
A0619542312
85,8
A0619646994
86,5
A0619800422
A0619783636
A0619777999
89,2
A0619814864
C0733133873het
A0619874471
A0619965312
91,7
C07r1965967
A0619998593
94,2
A0620071865
A0620084110
95,0
A0620396428
103,7
A0620526760
104,5
A0620815503
A0620608046
A0620611684
105,9
Cnnr74769999
A0620843339
A0620555399
A0620869458
109,1
A0620865944
A0620921104
112,0
A0621365691
113,4
A0621026646
A0621526763
A0621577498
115,9
A0621528844
A0621602441
A0621597660
C07r1692806
118,4
A0621646995
A0621620843
A0621960156
120,3
A0621931637
A0621972197
121,0
A0622682822
C0728232031
125,4
A0622661352
A0622698370
126,1
A0622856805
127,6
A0623040319
A0623046539
129,7
A0623045876
A0623095245
130,5
A0623135452
132,2
A0623146468
A0623213062
134,0
Annr38984779
A0623296286
135,9
A0623335138
138,4
A0623440068
139,6
A0623444303
A0623495918
A06r1993455
140,9
A0623504975
A0623837537
155,0
Ex*V8
A06
A06144719
0,0
A06168991
0,4
A06260373PA
0,9
A06266415
4,6
A06272291
A06787710
13,8
A061045812
14,3
A06r13027
A061442743
19,5
A061447161
20,4
A061469839
20,9
A061780626
26,0
A06r75970
27,2
A06r99792
27,8
A061846467
28,7
A061866374
29,6
A062356360
31,3
A062426020
31,7
A062420107
32,6
A062543044
35,0
A062594640
36,1
A062730311
41,1
A062788973
42,0
A062855599
42,5
A062854201
43,4
A062900889
44,7
A06r165035
47,6
A063026147
49,0
A063043132
A063053138
50,6
A06r218888
51,0
A063060035
51,3
A06r208992
52,4
A063143114
54,8
A063156905
A063143178
55,9
A063231009
A063232881
60,6
A063247125
A063371304
64,1
A063483322
66,1
A063495951
66,6
A063577817
68,5
A063759027
71,5
A063743036
72,5
A06r301737
78,0
A06r310914
78,5
A06r444255
80,8
A064791517
81,9
C055446597
A065258210
85,1
A065138085
86,0
A065334805
88,8
A065340130
90,2
A065583362
92,8
A066017553
93,7
A065890452
94,7
A065896857
A066758391
101,6
A066807432
A068046270
106,0
A068303303
106,6
A068305642
A0615512066
110,4
A06r1409770
113,7
A0614695012
114,2
A0610040143
116,1
A0616498857
119,0
A0616801220
119,5
A0617020136
121,0
A0617620358
122,0
A0617866196
123,4
A0617351478
125,3
A0617353389
A0618438952
127,7
A0619304866
130,9
A0619516128
132,2
A0619537354
133,1
A0619504351
133,6
A0619542312
134,5
A0619646994
136,0
A0619783636
138,2
A0619777999
138,8
C0733133873
140,1
A0619814864
141,3
A0619800422
142,4
C07r1965967
144,0
A0619874471
144,5
A0620071865
146,6
A0619998593
147,1
A0620084110
147,6
A0620494551
151,7
A0620496170
A0620608046
156,1
A0620611684
156,7
Cnnr74769999
157,1
A0620843339
157,6
A0620865944
158,5
A0620869458
160,5
A0621026646
161,9
A0621365691
165,9
A0621528844
170,9
A0621597660
171,7
A0621602441
172,1
A0621620843
172,5
A0621960156
174,7
A0621931637
176,8
A0621972197
177,3
C0728232031
182,6
A0622661352
A0622682822
186,0
A0622683231
A0622698370
A0623045876
190,2
A0623046539
A0623213062
190,7
A0623296286
191,2
A0623155725
196,8
A0623040319
199,4
A0623074971PA
A06r1900475
205,4
A0623440068
208,2
A0623495918
210,8
A0623504975
A0623647354
217,7
A0623649814
A0623629468
218,4
A0623595421
225,0
A0623605060
A06r1993455
226,9
A0623320798
228,8
A0623444303
229,7
Annr6049010
233,8
A0623837537
239,6
Ex*R53
A06-1
A067781073
0,0
A067829462
0,4
A08r879435
2,5
A068305642
3,0
A069639372
3,6
C0342939950
A069743291
A0614695012
4,1
A069933101
A068303303
A069876770
A0615557174
A0615358090
5,7
A0615548804
A0615951511
6,3
A0616038171
7,0
A0616232184
A0616763454
9,0
A0617020136
A0617353389
11,1
A0617351478
C03r2551506
12,3
A0617866196
A0618090767
13,7
A0618151210
15,2
A0618116017
A0618243074
17,2
A0618438952
A0618682144
18,2
A0618719972
19,0
A0618768717
19,7
A0618809439
19,9
A0618768241
20,3
A06r1516399
20,8
A0619304866
21,5
A0619504351
22,1
A0619340367
A0619529401
24,2
A0619537354
A0619542312
25,0
A0619531548
A0619553709
25,8
A0619783636
A0619646994
26,6
A0619800422
C07r1965967
A0619965312
27,3
A0619988939
28,6
A0619998593
A0620084110
30,3
A0620111194
A0620396428
31,1
A0620298154
A0620503658
33,3
A0620555399
A0620548350
34,4
A0620546804
A0620815503
34,8
A0621132016
40,5
C0730970547
40,9
A0621528844
42,4
A0621577498
A0621597660
A0621620843
43,6
A0621602441
C07r1692806
44,5
A0621646995
45,0
A0621809510
46,0
A0621836415
46,4
A0621960156
47,3
A0621963704
A0622270600
49,9
Annr33809729
51,8
A06r1788944
52,7
A0622518960
55,4
A0622556366
55,8
A0622588158
56,5
A0622661352
A0622698370
57,1
A0622682822
A0622683231
A0623046539
60,5
A0623045876
A0623095245
60,9
A0623135452
A0623146420
62,9
A0623146468
Annr38984779
63,9
A0623296286
65,0
A0623320798
66,5
A0623444303
67,0
A0623440068
A0623504975
68,6
A0623595421
70,0
A0623647354
70,7
A0623649814
A0623739423
71,1
Annr5997426
71,5
Annr10776746
74,0
Annr10759614
74,4
Annr10755332
74,8
A0623837537
75,6
A0624109014
76,0
A0624211904
77,3
Ex*R53
A06-2
A06144719
0,0
A06168991
A06272291
0,4
A06266415
A06r13027
A06787710
4,2
A061045812
A061442743
A061447161
7,1
A061469839
A06r99792
8,4
A061841462
8,8
A061846467
9,3
A062274725
11,6
A062420107
12,0
A062400369
A062426020
12,5
A062484592
13,0
A062501015
13,6
A062543044
14,1
A062730311
A062610035
16,1
A062664631
A062838106
18,3
A062840416
18,7
A062855599
20,6
A062900889
21,0
A062904271
C053221159
22,2
A06r176527
23,9
A063026147
A063053138
A063060035
A06r208992
25,9
A063043132
A06r218888
A063231009
27,7
A063247125
28,8
A063232881
A06r241423
30,0

## Slide 7
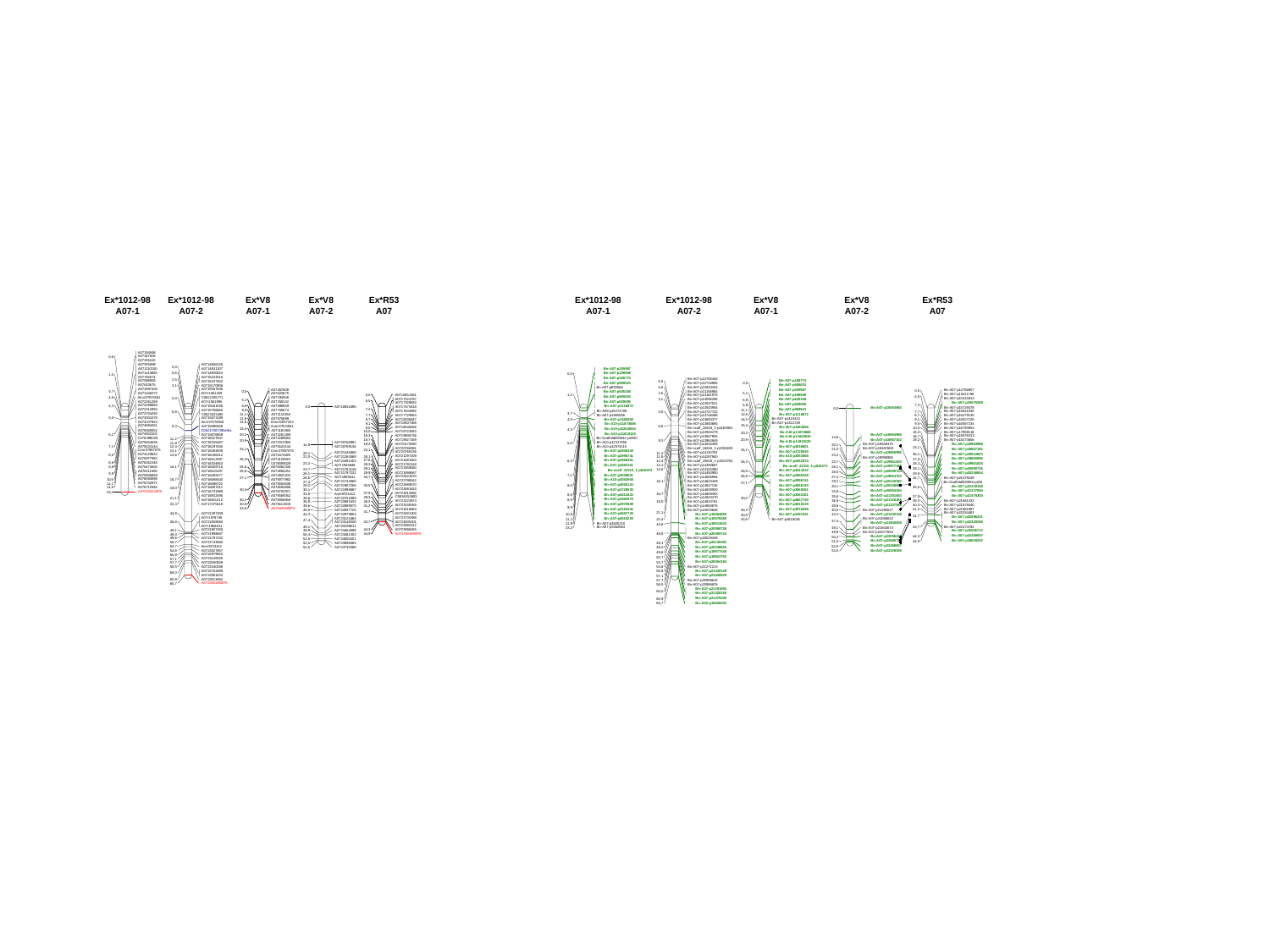

Ex*1012-98
A07-1
Ex*1012-98
A07-2
Ex*V8
A07-1
Ex*V8
A07-2
Ex*R53
A07
Bn-A07-p255987
Bn-A07-p199589
0,0
Bn-A07-p188774
Bn-A07-p12705404
Bn-A07-p188774
0,0
Bn-A07-p959523
Bn-A07-p12734895
0,0
Bn-A07-p665003
Bn-A07-p12833434
Bn-A07-p930068
0,6
Bn-A07-p255987
Bn-A07-p14766907
0,0
Bn-A07-p13156984
Bn-A07-p645188
5,1
2,5
Bn-A07-p15411766
Bn-A07-p199589
1,2
Bn-A07-p13161975
Bn-A07-p665003
6,5
Bn-A07-p15415913
Bn-A07-p13096286
3,1
Bn-A07-p645188
6,9
Bn-A07-p829095
Bn-A07-p15578269
Bn-A07-p13537021
Bn-A07-p829095
8,9
7,3
Bn-A07-p1118872
Bn-A07-p15729378
Bn-A07-p13543954
Bn-A07-p15054860
0,0
Bn-A07-p959523
11,1
Bn-A09-p18274708
Bn-A07-p15821030
7,7
Bn-A07-p13747722
5,0
3,7
Bn-A07-p1118872
12,8
Bn-A07-p1800556
Bn-A07-p16379135
Bn-A07-p13748498
8,7
Bn-A07-p1415413
Bn-A07-p1482858
14,5
Bn-A07-p16527225
9,1
4,0
Bn-A07-p13693377
Bn-A07-p1341729
Bn-A07-p16567234
9,5
Bn-A10-p11874688
Bn-A07-p13840665
15,4
6,8
Bn-A07-p1482858
Bn-A07-p16793852
Bn-scaff_15818_1-p2165992
10,0
Bn-A10-p11843630
4,3
Bn-A07-p17959118
Bn-A10-p11874688
18,3
Bn-A07-p13943479
20,2
Bn-A10-p11819120
Bn-A07-p18954998
Bn-A07-p18076313
Bn-A07-p13947955
18,7
Bn-A10-p11843630
14,8
Bn-Scaffold000632-p2930
Bn-A07-p18957162
20,9
Bn-A07-p18274656
19,2
Bn-A07-p13963849
9,3
Bn-A10-p11819120
Bn-A10-p12317050
5,0
Bn-A07-p18954998
Bn-A07-p14016405
Bn-A07-p19458370
20,1
Bn-A07-p3538601
Bn-A10-p12574115
23,1
Bn-scaff_15818_1-p2355639
Bn-A07-p19487659
21,0
Bn-A07-p18957162
25,1
Bn-A07-p2946329
Bn-A07-p3728535
Bn-A07-p14110753
Bn-A07-p19865996
11,2
Bn-A07-p19512603
26,1
23,2
Bn-A07-p2996741
Bn-A10-p3053568
Bn-A07-p14287940
11,8
Bn-A07-p19995985
Bn-A07-p19865996
27,8
Bn-A07-p3568361
Bn-scaff_15818_1-p3310794
6,2
12,4
Bn-A07-p3482570
26,3
Bn-A07-p19961825
23,7
Bn-A07-p19961825
28,3
Bn-A07-p14390667
13,1
Bn-A07-p3583161
Bn-scaff_22310_1-p815372
Bn-A07-p19977445
26,1
Bn-A07-p20090726
29,1
Bn-A07-p14325380
14,6
Bn-scaff_22310_1-p815372
Bn-A07-p4414432
26,6
Bn-A07-p20005724
26,5
Bn-A07-p14450900
Bn-A07-p20248654
29,9
Bn-A07-p3538601
Bn-A07-p2946329
7,1
26,8
Bn-A07-p19924753
27,4
Bn-A07-p14606994
Bn-A07-p21078249
34,7
Bn-A10-p3053568
Bn-A07-p2996741
Bn-A07-p20135392
Bn-A07-p14623449
16,1
29,2
Bn-Scaffold000856-p200
27,1
Bn-A07-p4050120
Bn-A07-p4050120
8,0
Bn-A07-p14627135
Bn-A07-p20248654
Bn-A07-p21328168
30,1
35,5
Bn-A07-p3728535
Bn-A07-p14628930
Bn-A07-p3568361
Bn-A07-p20554194
Bn-A07-p21337094
33,8
Bn-A07-p14809393
16,7
Bn-A07-p4414432
Bn-A07-p3583161
8,6
Bn-A07-p21191683
Bn-A07-p21475300
35,6
37,9
Bn-A07-p14823470
30,0
Bn-A07-p3482570
Bn-A07-p4687740
8,9
Bn-A07-p21328168
Bn-A07-p21681332
36,9
39,3
Bn-A07-p14823761
18,0
Bn-A07-p3975589
Bn-A07-p4815239
Bn-A07-p21678440
40,3
Bn-A07-p21337094
39,6
Bn-A07-p14863875
9,9
Bn-A07-p4191541
Bn-A07-p21961987
41,2
Bn-A07-p3975589
Bn-A07-p15033648
30,3
Bn-A07-p21280027
40,5
21,1
Bn-A07-p22034462
Bn-A07-p4687740
Bn-A07-p15054860
Bn-A07-p21430149
Bn-A07-p4191541
10,5
44,3
30,6
41,7
Bn-A07-p22096241
Bn-A07-p4815239
Bn-A07-p15578269
Bn-A07-p21586613
Bn-A07-p3618045
11,1
33,6
22,4
47,4
Bn-A07-p22329359
Bn-A07-p21600829
Bn-A07-p4825210
11,8
Bn-A07-p19512603
44,8
Bn-A07-p22474782
43,7
Bn-A07-p3262864
33,2
Bn-A07-p21844673
49,1
Bn-A07-p20090726
Bn-A07-p22586713
Bn-A07-p22077654
49,9
Bn-A07-p20005724
48,8
Bn-A07-p22488507
Bn-A07-p22096241
44,3
50,4
Bn-A07-p20029449
Bn-A02-p15628202
Bn-A07-p22586713
44,9
51,5
Bn-A07-p20135392
49,1
Bn-A07-p22488507
52,0
Bn-A07-p20248654
49,4
Bn-A07-p22329359
52,5
Bn-A07-p19977445
49,6
Bn-A07-p19924753
50,7
Bn-A07-p20554194
53,7
Bn-A07-p21271213
54,6
Bn-A07-p21430149
55,8
Bn-A07-p21600829
57,1
Bn-A07-p20999615
57,7
Bn-A07-p20986876
58,5
Bn-A07-p21191683
60,0
Bn-A07-p21328168
Bn-A07-p21475300
60,9
Bn-A02-p15628202
65,7
Ex*1012-98
A07-1
Ex*1012-98
A07-2
Ex*V8
A07-1
Ex*V8
A07-2
Ex*R53
A07
A07194948
A07267608
0,0
A07255342
A07975899
A0714806126
0,0
A071110150
A0714831307
A071145826
A0714930824
0,6
1,2
A07755874
A0715243016
2,5
A07598859
A0715237452
A07623675
A0715170906
3,1
A071897406
A0715557946
A07267608
3,7
0,0
A071346272
A07r1561209
A07623675
A0716651461
0,0
Annr37913561
4,0
C0621335771
A07194948
5,0
A0717324787
5,1
6,5
A072261269
A07r1561985
A07255342
A0717328832
A072289684
4,3
A0715551035
A07598859
6,9
A0716932495
0,0
A0717475418
7,3
A072312955
A0715786850
A07755874
8,9
A0717634892
6,8
A072704430
C0621523483
A071110150
11,1
A0717729854
7,7
A073001078
5,0
A0715873199
A07975899
12,8
A0718348847
8,7
A073247924
Annr37076816
Annr31957322
14,5
A0718507368
9,1
A074894251
A0715890559
Annr37913561
9,3
A0718545826
9,5
15,4
A075569352
C0621758739hetEx
A071182365
A0718723843
10,0
A074922202
6,2
A0715876859
A072261269
20,2
A0719806736
18,3
C078399029
A0716027847
A072289684
11,2
A0719927169
18,7
20,9
A075554636
A0716155487
A072312955
A0720766981
11,8
A0720170580
19,2
14,8
A075524144
7,1
A0716197455
A0720769136
12,4
A075524144
A0720766981
25,1
23,1
Cnnr37987075
A0716264839
Cnnr37987075
13,1
A0720769136
A0721245856
20,1
A074129822
8,0
A0716196312
14,6
A075474820
A0721297309
26,1
A0721264568
21,0
A075977982
A0716513397
A074129822
26,3
A0721681453
27,8
A0721681453
A076362345
8,6
A0716334853
C078399029
23,2
A0721782148
28,3
A07r1945949
A075474820
8,9
A0716509716
A076362345
16,1
26,6
A0721859060
29,1
A0721782148
23,7
A076111508
A0716515197
A074894251
26,8
A0721998687
29,9
A0721797232
9,9
26,1
A075906859
A0716492477
A074922202
A0722624670
34,7
A07r1955631
26,5
27,1
A076594698
10,5
A0716688440
A075977982
16,7
A0722798542
A0721743560
27,4
A076702971
11,1
A0716696722
A075554636
A0722889570
35,5
A0721907258
29,2
A076712945
11,8
A0716697012
A076594698
18,0
A0722881633
A0721998687
30,1
30,0
A0716731989
A075218416PA
A076702971
33,2
A0723013692
37,9
Annr9703412
33,8
A0716932495
A075569352
C0636153603
39,3
A0722752089
21,1
35,6
A0716911212
A075906859
30,3
A0723223874
40,3
A0722881633
36,9
A0717475418
A076111508
22,4
30,6
A0723349355
41,2
A0722889570
39,6
A075593585PA
33,6
A0723418664
A0722837720
40,5
41,7
A0723481303
A0721297309
44,8
A0722979853
44,3
A0723730388
A07r1978746
A0723124464
47,4
A0724005201
A0721859060
43,7
48,8
A0723149328
A0723885312
A07r1955631
A0723298011
49,1
A0723896065
A0721907258
44,3
49,1
A0723464598
49,9
A0721998687
A0723924890PA
44,9
49,4
A0723481303
50,4
A0721797232
49,6
A0724005201
51,5
A0721743560
50,7
A0723896065
52,0
Annr9703412
53,7
A0723730388
52,5
A0722827857
54,6
A0722979853
55,8
A0723149328
57,1
A0722550509
57,7
A0722540188
58,5
A0722752089
60,0
A0722881633
A0723013692
60,9
A0723924890PA
65,7

## Slide 8
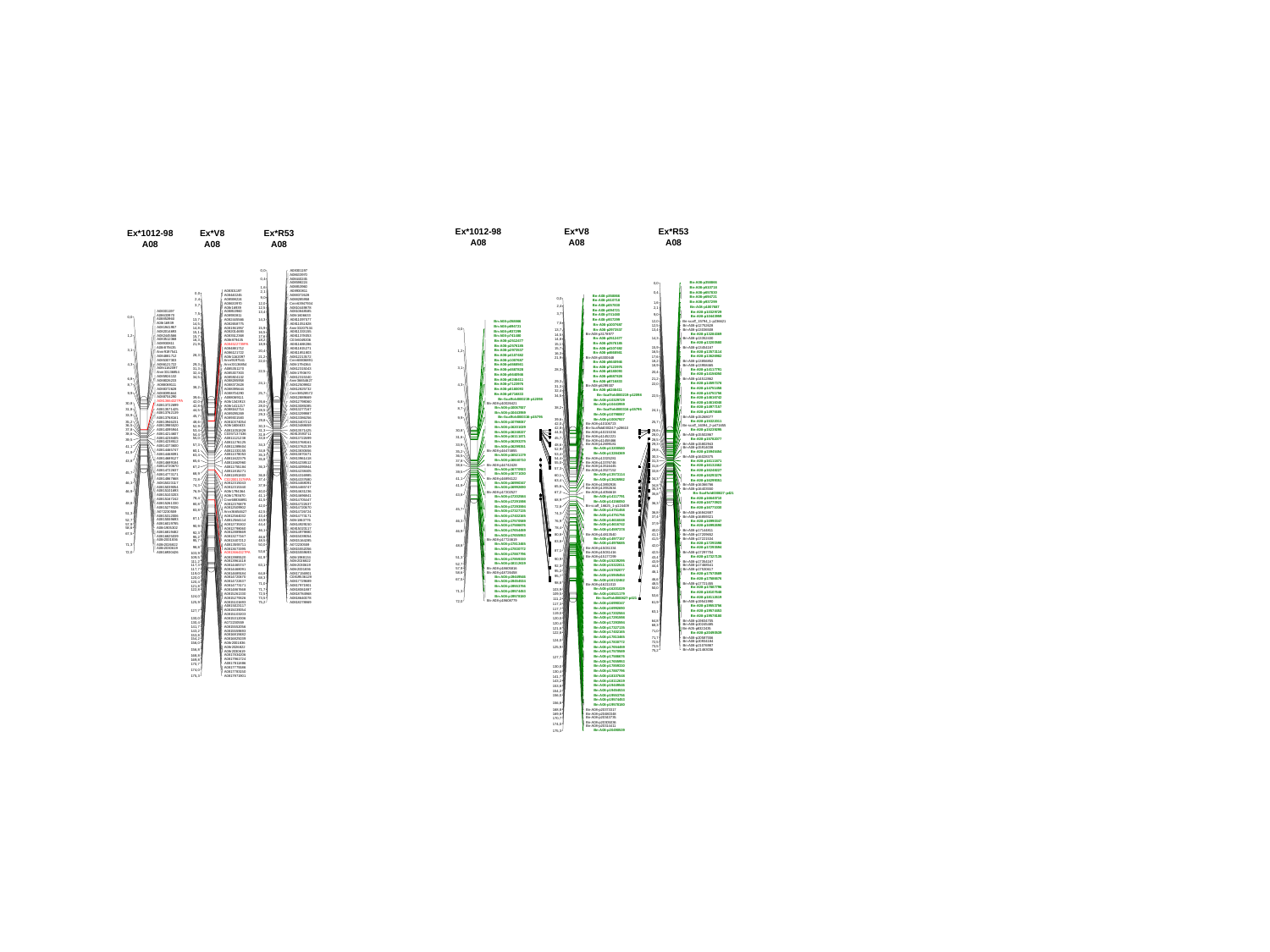

Ex*1012-98
A08
Ex*V8
A08
Ex*R53
A08
Bn-A08-p358866
0,0
Bn-A08-p510718
Bn-A08-p657830
0,4
Bn-A08-p694721
Bn-A08-p937299
1,6
Bn-A08-p1007687
2,1
Bn-A08-p10329729
9,0
Bn-A08-p10443959
Bn-scaff_15794_1-p236621
12,0
Bn-A08-p12752628
12,5
Bn-A08-p13036508
13,4
Bn-A08-p13284369
Bn-A08-p13352430
14,3
Bn-A08-p13200580
Bn-A08-p13454167
15,9
Bn-A08-p13573114
16,5
Bn-A08-p13626982
17,6
Bn-A08-p13856852
18,2
Bn-A08-p13958465
18,9
Bn-A08-p14117791
20,4
Bn-A08-p14156050
Bn-A08-p14512862
21,2
Bn-A08-p14597378
22,0
Bn-A08-p14751458
Bn-A08-p14751756
22,5
Bn-A08-p14616742
Bn-A08-p14616048
Bn-A08-p14977157
24,1
Bn-A08-p14976685
Bn-A08-p15268077
Bn-A08-p15322011
25,7
Bn-scaff_16394_2-p471655
Bn-A08-p15239295
26,6
Bn-A08-p15502867
28,0
Bn-A08-p15782077
28,5
Bn-A08-p15802943
29,3
Bn-A08-p15914038
29,8
Bn-A08-p15945454
Bn-A08-p16028375
30,3
Bn-A08-p16111871
31,3
Bn-A08-p16132462
31,8
Bn-A08-p16248227
33,8
Bn-A08-p16293275
34,3
Bn-A08-p16299351
Bn-A08-p16366766
34,8
Bn-A08-p16403550
35,3
Bn-Scaffold000827-p421
35,8
Bn-A08-p16640710
Bn-A08-p16770923
36,3
Bn-A08-p16771030
Bn-A08-p16842687
36,8
Bn-A08-p16859321
37,4
Bn-A08-p16990347
37,9
Bn-A08-p16992690
Bn-A08-p17144811
40,0
Bn-A08-p17209652
41,1
Bn-A08-p17221534
41,5
Bn-A08-p17291598
42,0
Bn-A08-p17293594
Bn-A08-p17297704
42,5
Bn-A08-p17327135
Bn-A08-p358866
0,0
Bn-A08-p510718
Bn-A08-p657830
2,4
Bn-A08-p694721
3,7
Bn-A08-p741480
Bn-A08-p937299
7,5
Bn-A08-p1007687
Bn-A08-p2973537
13,7
Bn-A08-p3178977
14,5
Bn-A08-p2512477
14,8
Bn-A08-p2576185
15,1
Bn-A08-p4107482
15,7
Bn-A08-p4668561
16,3
Bn-A08-p5300648
21,9
Bn-A08-p5640946
Bn-A08-p7123976
28,3
Bn-A08-p8180093
Bn-A08-p4687828
Bn-A08-p8716833
29,3
Bn-A08-p6299307
31,3
Bn-A08-p6246411
32,4
Bn-Scaffold000219-p12098
34,5
Bn-A08-p10329729
Bn-A08-p10443959
38,2
Bn-Scaffold000316-p15795
Bn-A08-p10798067
Bn-A08-p10067927
39,6
Bn-A08-p10106723
42,0
Bn-Scaffold000247-p28610
42,8
Bn-A08-p10215334
44,5
Bn-A08-p11452221
45,7
Bn-A08-p11458466
Bn-A08-p12699181
48,6
Bn-A08-p13200580
52,9
Bn-A08-p13284369
53,4
Bn-A08-p13325281
54,4
Bn-A08-p13376746
55,0
Bn-A08-p13516445
57,3
Bn-A08-p13527152
Bn-A08-p13573114
60,1
Bn-A08-p13626982
63,4
Bn-A08-p13892926
65,6
Bn-A08-p13932634
Bn-A08-p14056618
67,2
Bn-A08-p14117791
68,9
Bn-A08-p14156050
Bn-scaff_16625_1-p124409
72,8
Bn-A08-p14751458
74,3
Bn-A08-p14751756
Bn-A08-p14616048
76,9
Bn-A08-p14616742
78,4
Bn-A08-p14597378
Bn-A08-p14813540
80,8
Bn-A08-p14977157
83,8
Bn-A08-p14976685
Bn-A08-p15031334
87,1
Bn-A08-p15031416
Bn-A08-p15177299
90,9
Bn-A08-p15239295
Bn-A08-p15322011
92,3
Bn-A08-p358866
Bn-A08-p694721
0,0
Bn-A08-p937299
Bn-A08-p741480
Bn-A08-p2512477
Bn-A08-p2576185
Bn-A08-p2973537
1,2
Bn-A08-p4107482
Bn-A08-p1007687
Bn-A08-p4668561
3,1
Bn-A08-p4687828
Bn-A08-p5640946
Bn-A08-p6246411
Bn-A08-p7123976
4,3
Bn-A08-p8180093
Bn-A08-p8716833
Bn-Scaffold000219-p12098
6,8
Bn-A08-p10026421
Bn-A08-p10067927
8,7
Bn-A08-p10443959
Bn-Scaffold000316-p15795
9,9
Bn-A08-p10798067
Bn-A08-p16201029
30,8
Bn-A08-p16248227
Bn-A08-p16111871
31,8
Bn-A08-p16293275
33,9
Bn-A08-p16299351
Bn-A08-p16474855
35,2
Bn-A08-p16521179
36,5
Bn-A08-p16640710
37,8
Bn-A08-p16742428
38,6
Bn-A08-p16770923
39,5
Bn-A08-p16771030
Bn-A08-p16894122
41,1
Bn-A08-p16990347
41,9
Bn-A08-p16992690
Bn-A08-p17202527
43,8
Bn-A08-p17202584
Bn-A08-p17291598
Bn-A08-p17293594
45,7
Bn-A08-p17327135
Bn-A08-p17402165
Bn-A08-p17570589
46,3
Bn-A08-p17586676
Bn-A08-p17654459
46,9
Bn-A08-p17655953
Bn-A08-p17724619
Bn-A08-p17813465
48,8
Bn-A08-p17830772
Bn-A08-p17887796
51,3
43,4
Bn-A08-p17859330
Bn-A08-p17354167
43,9
Bn-A08-p18112619
52,7
Bn-A08-p17468541
44,4
Bn-A08-p18605816
57,9
Bn-A08-p17530617
Bn-A08-p15782077
95,2
46,1
Bn-A08-p18726458
58,6
Bn-A08-p17570589
Bn-A08-p15945454
95,7
Bn-A08-p19449546
Bn-A08-p17586676
46,6
67,5
Bn-A08-p16132462
Bn-A08-p19454534
98,8
Bn-A08-p17721455
48,5
Bn-A08-p16211013
Bn-A08-p19553756
Bn-A08-p17887796
50,0
Bn-A08-p16201029
103,9
Bn-A08-p19574453
71,3
Bn-A08-p18107648
Bn-A08-p16521179
109,5
53,6
Bn-A08-p19578180
Bn-A08-p18112619
Bn-Scaffold000827-p421
111,2
Bn-A08-p19608779
72,0
Bn-A08-p19541990
61,9
Bn-A08-p16990347
117,3
Bn-A08-p19553756
Bn-A08-p16992690
117,7
Bn-A08-p19574453
63,1
Bn-A08-p17202584
119,0
Bn-A08-p19578180
Bn-A08-p17291598
120,0
Bn-A08-p19834705
64,8
Bn-A08-p17293594
120,4
Bn-A08-p20245485
68,3
Bn-A08-p17327135
121,8
Bn-A05-p8322435
71,0
Bn-A08-p17402165
Bn-A08-p20490539
122,8
Bn-A08-p17813465
Bn-A08-p20587556
71,7
124,0
Bn-A08-p20934184
72,5
Bn-A08-p17830772
Bn-A08-p21076987
73,5
Bn-A08-p17654459
125,9
Bn-A08-p21463036
75,2
Bn-A08-p17570589
Bn-A08-p17586676
127,7
Bn-A08-p17655953
Bn-A08-p17859330
130,0
Bn-A08-p17887796
130,4
Bn-A08-p18107648
141,7
Bn-A08-p18112619
143,2
Bn-A08-p19449546
153,8
Bn-A08-p19454534
154,2
Bn-A08-p19553756
156,0
Bn-A08-p19574453
156,8
Bn-A08-p19578180
Bn-A08-p20373317
168,8
Bn-A08-p20480348
169,8
Bn-A08-p20343735
170,7
Bn-A08-p20305036
174,0
Bn-A08-p20314411
Bn-A08-p20490539
175,3
Ex*1012-98
A08
Ex*V8
A08
Ex*R53
A08
A08301197
0,0
A08633970
A08440245
0,4
A08598224
A08853960
1,6
A08930811
2,1
A088372628
9,0
A088285958
Cnnr63947934
12,0
A0810449878
12,5
A0810848585
13,4
A08r1606633
A0811097577
14,3
A0811051828
Annr33207534
15,9
A0811333155
16,5
A0811378053
17,6
C03r6049206
18,2
A0811688286
18,9
A0811815271
20,4
A0811851803
A0812213572
21,2
Cnnr68836891
22,0
A08r1794364
A0812315043
22,5
A08r1793670
A0812315340
Annr36654627
24,1
A0812509902
A0812825732
Cnnr38528572
25,7
A0812889669
A0812798060
26,6
A0813085085
28,0
A0813277167
28,5
A0813299987
29,3
A0813386256
29,8
A0813407212
A0813488659
30,3
A0813571425
31,3
A0813593711
31,8
A0813722699
33,8
A0813768161
34,3
A0813762139
A0813830656
34,8
A0813870471
35,3
A0813961418
35,8
A0814238512
A0814095944
36,3
A0814238405
A0814316985
36,8
A0814337680
37,4
A0814468091
37,9
A0814465747
A0814631236
40,0
A0814696841
41,1
A0814705447
41,5
A0814722637
42,0
A0814720670
A0814726724
42,5
A0814773171
A08301197
0,0
A08440245
A08598224
2,4
A08633970
3,7
A08r16939
A08853960
7,5
A08930811
A082445566
13,7
A082658775
14,5
A081941957
14,8
A082014693
15,1
A083512368
15,7
A08r879435
16,3
A084522739PA
21,9
A084881712
A086121722
28,3
A08r1162097
Annr9197541
Annr33136854
29,3
A085351170
31,3
A085307303
32,4
A085924132
34,5
A088285958
A088372628
38,2
A088395644
A088704290
A088069111
39,6
A08r1343913
42,0
A08r1411217
42,8
A088162714
44,5
A089295269
45,7
A089301583
A0810374054
48,6
A08r1606633
52,9
A0811051828
53,4
C0357137436
54,4
A0811121238
55,0
A0811276125
57,3
A0811289604
A0811333155
60,1
A0811378053
63,4
A0811622375
65,6
A0811662960
A0811755184
67,2
A0811815271
68,9
A0811851803
C0220013178PA
72,8
A0812315043
74,3
A0812315340
A08r1794364
76,9
A08r1793670
78,4
Cnnr68836891
A0812376879
80,8
A0812509902
83,8
Annr36654627
A0812564032
87,1
A0812564114
A0812735302
90,9
A0812798060
A0812889669
92,3
A08301197
A08633970
0,0
A08853960
A08r16939
A081941957
A082014693
A082445566
1,2
A083512368
A08930811
A08r879435
3,1
Annr9197541
A084881712
A085307303
A086121722
4,3
A08r1162097
Annr33136854
A085924132
6,8
A088026233
A088069111
8,7
A088372628
A088395644
9,9
A088704290
A0813664327PA
30,8
A0813722699
A0813571425
31,8
A0813762139
33,9
A0813768161
A0813946231
35,2
A0813985520
36,5
A0814095944
37,8
A0814214607
38,6
A0814238405
39,5
A0814238512
A0814373600
41,1
A0814465747
41,9
A0814468091
A0814689127
43,8
A0814689184
A0814720670
A0814722637
45,7
A0814773171
A0814867668
A0815023117
46,3
A0815039054
A0815101693
46,9
A0815103203
A0815167242
A0815261330
48,8
A0815279326
A072230559
51,3
A0815312006
43,4
A0815559693
52,7
A08r1863776
43,9
A0816019785
57,9
A0814929150
44,4
A08r1905302
58,6
A0815023117
46,1
A0816819482
A0814979880
67,5
A0816825039
A0813277167
A0815039054
95,2
46,6
A08r2001836
A0813407212
A0815164285
95,7
48,5
A08r2026822
71,3
A0813593711
A072230559
50,0
98,8
A08r2030619
A0813673395
A0815552056
53,6
A0816930426
72,0
A0813664327PA
A0815559693
103,9
A0813985520
A08r1988134
109,5
61,9
A0813961418
A08r2026822
111,2
A0814465747
A08r2030619
117,3
63,1
A0814468091
A08r2001836
117,7
A0814689184
A0817156901
119,0
64,8
A0814720670
C0819536129
120,0
68,3
A0814722637
A0817729689
120,4
71,0
A0814773171
A0817971901
121,8
A0814867668
A0818061887
122,8
71,7
A0815261330
A0818784968
72,5
124,0
A0815279326
A0818640078
73,5
A0815101693
A0818278869
125,9
75,2
A0815023117
A0815039054
127,7
A0815103203
A0815312006
130,0
A072230559
130,4
A0815552056
141,7
A0815559693
143,2
A0816819482
153,8
A0816825039
154,2
A08r2001836
156,0
A08r2026822
156,8
A08r2030619
A0817834206
168,8
A0817961724
169,8
A0817811886
170,7
A0817775586
174,0
A0817783150
A0817971901
175,3

## Slide 9
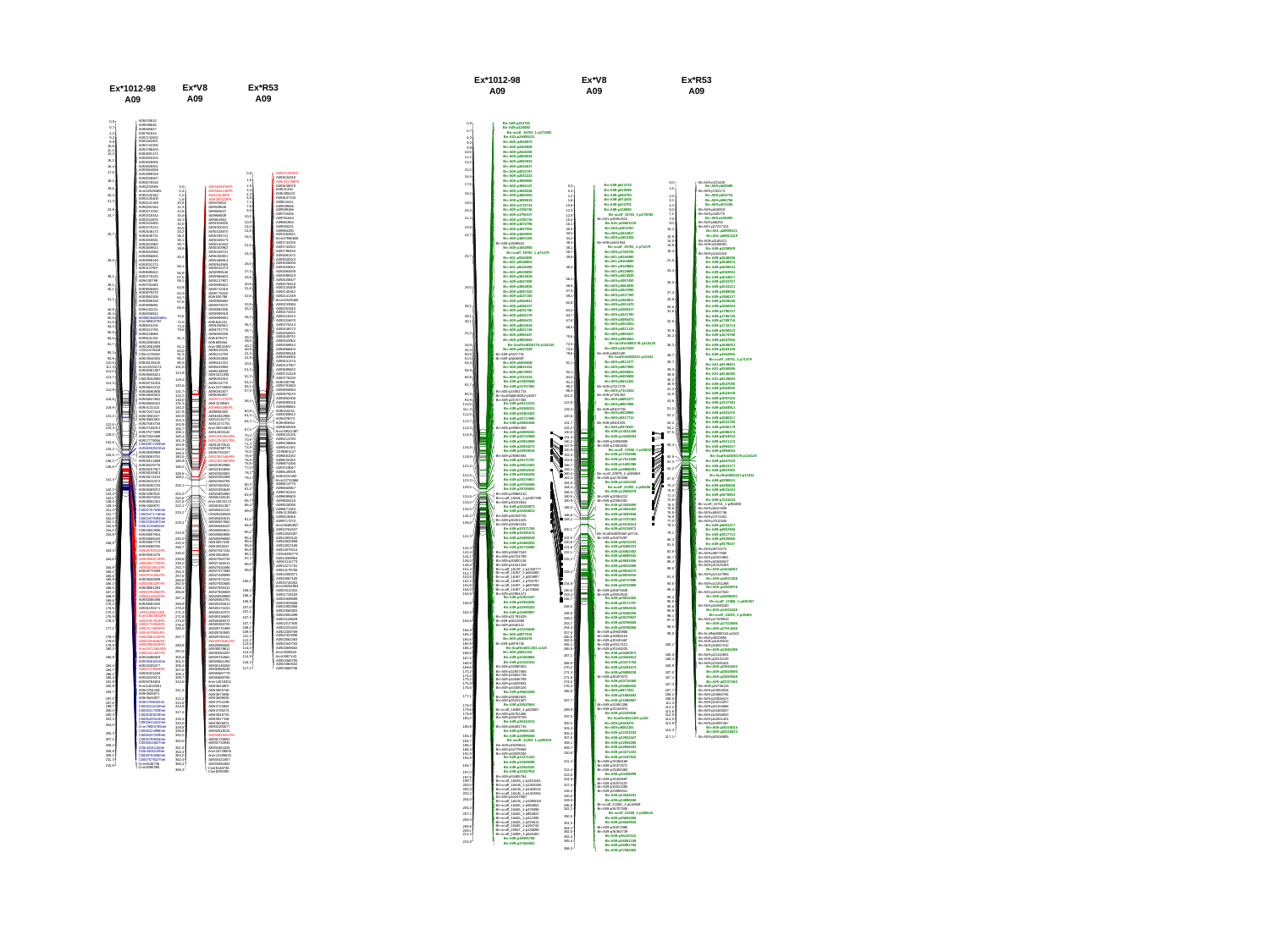

Ex*1012-98
A09
Ex*V8
A09
Ex*R53
A09
Bn-A09-p222701
0,0
Bn-A09-p135892
0,7
Bn-scaff_15783_1-p273092
Bn-A01-p26990131
4,2
Bn-A09-p2548974
9,2
Bn-A09-p2208929
9,9
Bn-A09-p2548356
10,6
Bn-A09-p2609043
11,2
Bn-A09-p2083933
13,2
Bn-A09-p2044817
15,2
Bn-A09-p2033767
Bn-A09-p2033221
16,4
Bn-A09-p1989556
17,6
Bn-A09-p1956137
Bn-A09-p1929245
18,2
Bn-A09-p1860063
Bn-A09-p1690633
19,5
Bn-A09-p1715714
Bn-A09-p1786726
20,4
Bn-A09-p1796337
21,3
Bn-A09-p1785718
Bn-A09-p1674796
23,8
Bn-A09-p1657554
Bn-A09-p1608051
24,7
Bn-A09-p1603105
Bn-A09-p1588624
Bn-A09-p1552993
Bn-scaff_19783_1-p71479
25,7
Bn-A01-p9150695
Bn-A01-p9149601
Bn-A01-p9146490
Bn-A01-p9129693
Bn-A09-p3024539
Bn-A09-p3047090
Bn-A09-p3064935
29,5
Bn-A09-p3097420
Bn-A09-p3237193
Bn-A09-p4384911
Bn-A09-p4286317
38,1
Bn-A09-p4241780
Bn-A09-p4206179
39,1
Bn-A09-p4096474
40,1
Bn-A09-p4024534
Bn-A09-p4021115
41,2
Bn-A09-p3994437
Bn-A09-p3994064
Bn-Scaffold000178-p118125
44,8
Bn-A09-p4447029
45,4
Bn-A09-p5327731
50,5
Bn-A09-p5406597
51,5
Bn-A09-p5455608
54,4
Bn-A09-p5631441
58,9
Bn-A09-p5678953
Bn-A09-p7331034
60,8
Bn-A09-p23693948
81,7
Bn-A09-p23707462
Bn-A05-p15551715
90,3
Bn-Scaffold000282-p6257
Bn-A09-p25747305
93,9
Bn-A09-p28212233
110,5
Bn-A09-p28266331
111,3
Bn-A09-p28463402
112,0
Bn-A09-p28717690
113,7
Bn-A09-p28681656
Bn-A09-p28802268
114,3
Bn-A09-p28695042
114,9
Bn-A09-p28722989
Bn-A09-p321430
0,0
Bn-A09-p620595
1,6
Bn-A09-p702173
Bn-A09-p614715
2,5
Bn-A09-p594794
3,3
Bn-A09-p574205
4,3
Bn-A09-p616919
5,0
Bn-A09-p160775
7,1
Bn-A09-p135892
7,8
Bn-A09-p86293
9,0
Bn-A01-p27017324
10,2
Bn-A01-p26990131
Bn-A01-p26921219
10,9
Bn-A09-p1165212
14,0
Bn-A09-p1188091
14,8
Bn-A09-p2208929
16,4
Bn-A09-p1404415
Bn-A09-p2548356
21,5
Bn-A09-p2548974
Bn-A09-p2609043
24,4
Bn-A09-p2083933
Bn-A09-p2044817
Bn-A09-p2033767
26,0
Bn-A09-p2033221
Bn-A09-p1989556
27,4
Bn-A09-p1956137
28,9
Bn-A09-p1929245
Bn-A09-p1860063
30,6
Bn-A09-p1796337
31,6
Bn-A09-p1786726
Bn-A09-p1785718
32,6
Bn-A09-p1715714
Bn-A09-p1690633
33,9
Bn-A09-p1674796
35,2
Bn-A09-p1657554
Bn-A09-p1608051
36,3
Bn-A09-p1603105
Bn-A09-p1552993
36,7
Bn-scaff_19783_1-p71479
38,7
Bn-A01-p9149601
Bn-A01-p9150695
39,3
Bn-A01-p9146490
39,8
Bn-A01-p9129693
40,2
Bn-A09-p3047090
40,9
Bn-A09-p3064935
41,3
Bn-A09-p3024539
41,9
Bn-A09-p3097420
43,6
Bn-A09-p3237193
Bn-A09-p4384911
51,2
Bn-A09-p4351470
52,2
Bn-A09-p4286317
Bn-A09-p4241780
54,2
Bn-A09-p4206179
55,1
Bn-A09-p4096474
57,5
Bn-A09-p4024534
Bn-A09-p4021115
58,4
Bn-A09-p3994437
Bn-A09-p3994064
Bn-Scaffold000178-p118125
60,8
Bn-A09-p4447029
61,3
Bn-A09-p614715
0,0
Bn-A09-p620595
0,4
Bn-A09-p594794
1,2
Bn-A09-p574205
1,6
Bn-A09-p222701
10,8
Bn-A09-p135892
12,3
Bn-scaff_15783_1-p273092
12,8
Bn-A01-p26914512
15,4
Bn-A01-p26921219
16,1
Bn-A09-p2033767
30,0
Bn-A09-p2044817
30,5
Bn-A09-p1603105
34,2
Bn-A09-p1631944
36,3
Bn-scaff_19783_1-p71479
38,1
Bn-A09-p1786726
38,7
Bn-A01-p9146490
39,8
Bn-A01-p9150695
Bn-A01-p9149601
45,4
Bn-A01-p9129693
Bn-A09-p3024539
56,1
Bn-A09-p3097420
Bn-A09-p3064935
56,8
Bn-A09-p3047090
57,5
Bn-A09-p3237193
59,1
Bn-A09-p4384911
62,8
Bn-A09-p4351470
Bn-A09-p4286317
63,4
Bn-A09-p4241780
64,7
Bn-A09-p4096474
67,6
Bn-A09-p4024534
69,4
Bn-A09-p4021115
Bn-A09-p3994437
70,6
Bn-A09-p3994064
Bn-Scaffold000178-p118125
72,5
Bn-A09-p4447029
73,4
Bn-A09-p4850180
79,6
Bn-Scaffold000321-p12341
Bn-A09-p4913177
81,1
Bn-A09-p4927893
Bn-A09-p5298521
82,1
Bn-A09-p5455608
84,0
Bn-A09-p5631441
91,3
Bn-A09-p7317278
95,2
Bn-A09-p7331034
96,3
Bn-A09-p7191362
101,0
Bn-A09-p8891277
113,9
Bn-A09-p8957856
Bn-A09-p9103739
119,4
Bn-A09-p9325966
120,6
Bn-A09-p9317714
Bn-A09-p9101925
121,7
Bn-A09-p9379347
122,2
Bn-A09-p14031358
130,0
Bn-A09-p14246053
131,3
Bn-A09-p13836909
140,2
Bn-A09-p13824652
Bn-A09-p28910059
Bn-A09-p28918272
116,8
Bn-A09-p28919016
Bn-A09-p29092594
119,9
Bn-A09-p29171797
Bn-A09-p29012402
121,2
Bn-A09-p29052616
Bn-A09-p29186255
122,6
Bn-A09-p29275947
123,3
Bn-A09-p29756068
129,5
Bn-A09-p29785600
Bn-A09-p29968210
132,6
Bn-scaff_16361_1-p3397399
Bn-A09-p30233844
133,2
Bn-A09-p30280973
134,5
Bn-A09-p30284813
Bn-A09-p30339735
136,2
Bn-A09-p30431025
138,8
Bn-A09-p30463234
Bn-A09-p30371768
Bn-A09-p30391674
141,3
Bn-A09-p25899239
Bn-A09-p30466030
Bn-A09-p30718480
142,3
Bn-A09-p30627540
143,4
Bn-A09-p30724789
144,1
Bn-A09-p30800136
146,0
Bn-A09-p31041328
149,4
Bn-scaff_16197_1-p1348777
151,3
Bn-scaff_16197_1-p561660
151,7
Bn-scaff_16197_1-p553997
152,0
Bn-scaff_16197_1-p784797
152,2
Bn-scaff_16197_1-p697008
152,6
Bn-scaff_16197_1-p178006
154,0
Bn-A09-p32954471
154,8
Bn-A09-p32923447
Bn-A09-p32944206
156,9
Bn-A09-p32945202
Bn-A09-p31890957
163,3
Bn-A09-p31781629
Bn-A09-p5013589
164,6
Bn-A09-p5040112
Bn-A09-p32225646
164,9
Bn-A09-p5977032
165,2
Bn-A09-p5168276
165,6
Bn-A09-p4976736
165,9
Bn-Scaffold011301-p120
166,2
Bn-A09-p5062101
166,5
Bn-A09-p31884693
167,2
Bn-A09-p32321534
168,8
Bn-A09-p32590303
169,6
Bn-A09-p32937856
170,2
Bn-A09-p33942716
174,5
Bn-A09-p34046789
175,2
Bn-A09-p34029934
175,9
Bn-A09-p34308105
176,5
Bn-A09-p35604358
177,1
Bn-A09-p36062825
Bn-A09-p35221507
Bn-A09-p35520944
178,3
147,9
Bn-scaff_17888_1-p435307
150,9
Bn-A09-p17332686
153,3
Bn-A09-p17511656
153,9
Bn-A09-p11692396
156,7
Bn-A09-p16998291
158,1
Bn-scaff_20979_1-p309863
160,4
Bn-A09-p14783306
161,3
Bn-A09-p14432426
163,8
Bn-scaff_23293_1-p25406
164,4
Bn-A09-p12929078
166,4
Bn-A09-p23554212
180,5
Bn-A09-p23554351
180,9
Bn-A09-p23525690
186,0
Bn-A09-p23554402
Bn-A09-p23693948
188,6
Bn-A09-p23707462
189,2
Bn-A09-p25316014
Bn-A09-p25319672
200,1
Bn-Scaffold000460-p5716
Bn-A09-p25575397
202,0
Bn-A09-p28212233
220,6
Bn-A09-p28266331
221,6
Bn-A09-p28463402
222,1
Bn-A09-p28695042
225,2
Bn-A09-p28681656
Bn-A09-p28910059
Bn-A09-p28918272
229,4
Bn-A09-p28919016
Bn-A09-p28717690
234,9
Bn-A09-p28722989
Bn-A09-p28670849
240,0
Bn-A09-p29019543
243,4
Bn-A09-p29012402
246,7
Bn-A09-p29171797
248,0
Bn-A09-p29052616
Bn-A09-p29186255
248,6
Bn-A09-p29275947
249,2
Bn-A09-p29785600
253,7
Bn-A09-p29756068
254,3
Bn-A09-p29630906
257,6
Bn-A09-p30058110
260,6
Bn-A09-p30105467
262,5
Bn-A09-p30117412
265,1
Bn-A09-p30145025
265,8
Bn-A09-p30280973
267,1
Bn-A09-p30284813
Bn-A09-p30371768
268,9
Bn-A09-p30391674
270,2
Bn-A09-p25899239
271,3
Bn-A09-p30437673
271,8
Bn-A09-p30718480
274,0
Bn-A09-p30466030
276,4
Bn-A09-p5977032
286,5
Bn-A09-p31884693
Bn-A09-p31890957
287,7
Bn-A09-p31891306
Bn-A09-p4913177
64,2
Bn-A09-p4927893
Bn-Scaffold000321-p12341
67,5
Bn-A09-p5298521
Bn-A09-p5455608
70,4
Bn-A09-p5631441
70,9
Bn-A09-p5678953
71,3
Bn-A09-p7331034
72,8
Bn-scaff_15701_1-p994892
76,5
Bn-A09-p6021939
76,6
Bn-A09-p8591746
76,8
Bn-A09-p7472401
76,9
Bn-A09-p7510345
77,0
Bn-A09-p8891277
78,2
Bn-A09-p8957856
79,1
Bn-A09-p9317714
Bn-A09-p9325966
80,3
Bn-A09-p9379347
81,5
Bn-A09-p9724274
82,8
Bn-A09-p9877560
Bn-A09-p10023881
85,7
Bn-A09-p10046047
86,4
Bn-A09-p10325493
89,0
Bn-A09-p14246053
Bn-A09-p14147959
91,0
Bn-A09-p14031358
Bn-A09-p13251498
94,6
Bn-A09-p12929078
95,2
Bn-A05-p18147040
Bn-A09-p16998291
95,4
Bn-scaff_17888_1-p435307
95,5
Bn-A09-p15090180
95,6
Bn-A09-p14432426
95,8
Bn-scaff_23293_1-p25406
96,1
Bn-A09-p17509910
97,3
Bn-A09-p17332686
98,5
Bn-A09-p17511656
Bn-Scaffold000344-p1622
99,5
Bn-A08-p9323955
Bn-A09-p11630515
100,1
Bn-A09-p19057312
Bn-A09-p11692396
Bn-A09-p23144931
106,0
Bn-A09-p23331135
106,4
Bn-A09-p23445424
106,9
Bn-A09-p23554402
Bn-A09-p23525690
107,0
Bn-A09-p23693948
107,1
Bn-A09-p23707462
107,3
Bn-A09-p23756115
Bn-A09-p23852034
107,7
Bn-A09-p23865794
108,1
Bn-A09-p23928417
109,5
Bn-A09-p24014257
111,3
Bn-A09-p24104666
112,2
Bn-A09-p24400007
113,6
Bn-A09-p24556003
114,0
Bn-A09-p32104974
289,9
Bn-scaff_16389_1-p428067
179,6
Bn-A09-p32225646
Bn-A09-p35753486
179,9
297,5
Bn-A09-p35879759
180,2
Bn-Scaffold011301-p120
Bn-A09-p24921401
114,5
Bn-A09-p36410312
Bn-A09-p5168276
Bn-A09-p24987467
114,9
300,4
Bn-A09-p36491715
180,8
Bn-A09-p25316014
Bn-A09-p5062101
301,0
116,3
Bn-A09-p36551108
Bn-A09-p25319672
Bn-A09-p32321534
305,4
Bn-A09-p34995048
184,4
Bn-A09-p25536905
117,1
Bn-A09-p32923447
307,6
Bn-scaff_21269_1-p285510
184,7
Bn-A09-p32944206
308,1
Bn-A09-p35299111
186,2
Bn-A09-p32945202
308,7
Bn-A09-p34779068
186,4
Bn-A09-p33271433
310,6
Bn-A09-p33029336
191,9
Bn-A09-p33347915
Bn-A09-p33271433
192,9
311,3
Bn-A09-p33358168
Bn-A09-p33459299
194,7
Bn-A09-p33372571
Bn-A09-p33544191
Bn-A09-p33400463
312,4
Bn-A09-p33347915
197,0
Bn-A09-p33459299
313,6
Bn-A09-p33485784
197,8
Bn-A09-p33428597
314,9
Bn-scaff_16445_1-p1451181
199,7
Bn-A09-p33574107
Bn-scaff_16445_1-p1450260
317,4
200,0
Bn-A09-p33521094
Bn-scaff_16445_1-p1459241
200,3
Bn-A09-p33595011
318,2
Bn-scaff_16445_1-p1342951
203,3
Bn-A09-p33544191
320,0
Bn-A09-p34247590
204,0
Bn-A09-p34995048
329,8
Bn-scaff_16445_1-p1085018
Bn-scaff_21269_1-p118949
336,8
Bn-scaff_16445_1-p584652
205,3
Bn-A09-p35707505
342,2
Bn-scaff_16445_1-p478398
Bn-scaff_21269_1-p285510
Bn-scaff_16445_1-p984822
207,1
350,5
Bn-scaff_16445_1-p312395
Bn-A09-p35604358
208,4
Bn-scaff_16445_1-p225513
Bn-A09-p35520944
351,5
Bn-scaff_16445_1-p194744
208,8
Bn-A09-p35872885
354,3
Bn-scaff_20947_1-p139098
209,1
Bn-A09-p36363739
362,0
Bn-scaff_16389_1-p526483
211,3
Bn-A09-p36410312
362,4
Bn-A09-p36991758
Bn-A09-p36551108
365,4
215,9
Bn-A09-p37082082
Bn-A09-p36991758
368,3
Bn-A09-p37082082
Ex*V8
A09
Ex*R53
A09
Ex*1012-98
A09
A09476810
0,0
A09539846
0,7
A09565847
A09761644
4,2
A091742822
9,2
A091382821
9,9
A091742205
10,6
A091796434
11,2
A091881472
13,2
A091920224
15,2
A091928005
A091928551
16,4
A091964559
17,6
A091999319
A092026507
18,2
A092076518
A092233585
19,5
Annr32529405
A092140342
20,4
A092135409
21,3
A092141349
A092261544
23,8
A092274332
A092318314
24,7
A092324670
A092343400
A092375213
A092448173
25,7
A092449731
A092450831
A092453962
A092469614
A092944565
A092966603
A092999148
29,5
A093031274
A093137907
A093695622
A093776220
38,1
A09r530798
A093791683
39,1
A093906694
40,1
A093979270
A093982306
41,2
A093999318
A093999691
A09r610241
44,8
A094358812
45,4
A095008448hetEx
50,5
Cnnr58302787
51,5
A095015235
54,4
A095213793
58,9
A095238666
A095541331
60,8
A0922065803
81,7
A0922081699
C0511478118
90,3
C05r1078380
A0923840550
93,9
A0926138145
110,5
Annr15533174
111,3
A0926361367
112,0
A0926655621
113,7
C0829160069
A0926734204
114,3
A0926632132
114,9
A0926660856
A09371063PA
0,0
A091634318
1,6
A09r231786PA
A091638078
2,5
A09r21430
3,3
A09r308423
4,3
A091637130
5,0
A09515411
7,1
A09539846
7,8
A09599166
9,0
A09733456
10,2
A09761644
A09861964
10,9
A09940331
14,0
A09963280
14,8
A091382821
16,4
Annr47696826
A091742205
21,5
A091742822
A091796434
24,4
A091881472
A091920224
A091928005
26,0
A091928551
A091964559
27,4
A091999319
28,9
A092026507
A092076518
30,6
A092135409
31,6
A092140342
A092141349
32,6
Annr32529405
A092233585
33,9
A092261544
35,2
A092274332
A092318314
36,3
A092324670
A092375213
36,7
A092448173
38,7
A092450831
A092449731
39,3
A092453962
39,8
A092469614
40,2
A092966603
40,9
A092999148
41,3
A092944565
41,9
A093031274
43,6
A093137907
A093695622
51,2
A093722419
52,2
A093776220
A09r530798
54,2
A093791683
55,1
A093906694
57,5
A093979270
A093982306
58,4
A093999318
A093999691
A09r610241
60,8
A094358812
61,3
A091638078PA
0,0
A091634318PA
0,4
A09r21430PA
1,2
A09r308423PA
1,6
A09476810
10,8
A09539846
12,3
A09565847
12,8
A09866809
15,4
A09861964
16,1
A091928005
30,0
A091920224
30,5
A092324670
34,2
A092294741
36,3
A092448173
38,1
A092140342
38,7
A092453962
39,8
A092449731
A092450831
45,4
A092469614
A092944565
56,1
A093031274
A092999148
56,8
A092966603
57,5
A093137907
59,1
A093695622
62,8
A093722419
A093776220
63,4
A09r530798
64,7
A093906694
67,6
A093979270
69,4
A093982306
A093999318
70,6
A093999691
A09r610241
72,5
A094358812
73,4
A094757775
79,6
A094850038
A09r679073
81,1
A09r693654
Annr39532497
82,1
A095015235
84,0
A095213793
91,3
A095553806
95,2
A095541331
96,3
A095643968
101,0
A098149008
113,9
A09r1031392
A098283324
119,4
A098414770
120,6
Annr22733866
A098281507
121,7
A098460857
122,2
A099717275PA
130,0
A09r1139563
131,3
A099944096PA
140,2
A099950392
A0926840515
A0926847662
116,8
A0926848422
A09r3132115
119,9
A0927047244
A09r3050247
121,2
A09r3084563
A0927063736
122,6
A0927166013
123,3
A0927577699
129,5
A0927620498
A0927779056
132,6
C0830871209het
A0928002503het
133,2
A0928059989
134,5
A0928063791
A0928121888
136,2
A0928229776
138,8
A0928257927
A0928155813
A0928174153
141,3
A0928210372
A0928260725
A0928469372
142,3
A09r3390919
143,4
A0928475632
144,1
A0928552451
146,0
A09r3458870
149,4
C0832767625het
151,3
C0833471716het
151,7
C0833479393het
152,0
C0833306357het
152,2
C08r4133606het
152,6
C0833832656
154,0
A0930697654
154,8
A0930668240
A0930687779
156,9
A0930688780
A0929790102PA
163,3
A0929453276
A0929598719PA
164,6
A0929637783PA
A0930078611PA
164,9
A0929770499
165,2
A0929744561PA
165,6
A0929555599
165,9
A0930084297PA
166,2
A0929661294
166,5
A0929782892PA
167,2
A0930146204PA
168,8
A0930386496
169,6
A0930681505
170,2
A0931193171
174,5
A0931299111PA
175,2
Annr42605842PA
175,9
A0931557619PA
176,5
A0932733945PA
A0933176693PA
177,1
A09r4075901PA
A0932661635PA
178,3
147,9
A0910842958
150,9
A0913114773
153,3
A0913271701
153,9
Annr39254903
156,7
A0912833142
158,1
A0912253815PA
160,4
A0912355017PA
161,3
A0912675514
163,8
C0918290779
164,4
A0910761537
166,4
A0921933466PA
180,5
A0921933605PA
180,9
A0921902968
186,0
A0921933656
A0922065803
188,6
A0922081699
189,2
A0923360705
A0923363532
200,1
A0923394648
A0923692880
202,0
A0926138145
220,6
Annr15533174
221,6
A0926361367
222,1
A0926632132
225,2
C0829160069
A0926840515
A0926847662
229,4
A0926848422
A0926655621
234,9
A0926660856
A0926599069
240,0
A09r3057439
243,4
A09r3050247
246,7
A0927047244
248,0
A09r3084563
A0927063736
248,6
A0927166013
249,2
A0927620498
253,7
A0927577699
254,3
A0927449599
257,6
A0927873120
260,6
A0927920568
262,5
A0927935312
265,1
A0927948009
265,8
A0928059989
267,1
A0928063791
A0928155813
268,9
A0928174153
270,2
A0928210372
271,3
A0928236600
271,8
A0928469372
274,0
A0928260725
276,4
A0929770499
286,5
A0929782892
A0929790102
287,7
A0929790451PA
A09r679073
64,2
A09r693654
A094850038
67,5
Annr39532497
A095015235
70,4
A095213793
70,9
A095238666
71,3
A095541331
72,8
C099401127
76,5
A096661032
76,6
A098020182
76,8
A096974166
76,9
A097019047
77,0
A098149008
78,2
A09r1031392
79,1
Annr22733866
A098414770
80,3
A098460857
81,5
A098782103
82,8
A098899405
A099008110
85,7
A099028295
86,4
A099571283
89,0
A09r1139563
A099216946
91,0
A099717275
Annr38460947
94,6
A0910761537
95,2
A0912040387
A0912833142
95,4
A0910842958
95,5
A0912023106
95,6
A0912675514
95,8
C0918290779
96,1
A0913269954
97,3
A0913114773
98,5
A0913271701
A0914179756
99,5
A0914393571
A0915067165
100,1
A0915745353
Annr39254903
A0921512422
106,0
A0921725183
106,4
A0921849699
106,9
A0921933656
A0921902968
107,0
A0922065803
107,1
A0922081699
107,3
A0922128629
A0922217935
107,7
A0922231633
108,1
A0922308789
109,5
A0922323588
111,3
A0923062383
112,2
A0922443783
113,6
A0922608602
114,0
A0933256060PA
179,6
A0932900829PA
A0929995505
179,9
289,9
Annr16722653PA
A0930078611
180,2
297,5
Annr3930652
114,5
A0933421857PA
A0930084297
Annr3867143
114,9
A0933496929
A0929744561
180,8
300,4
A0923360705
A0933561852het
A0929661294
301,0
116,3
A0923363532
A0932205577
184,4
A0930146204
305,4
A0923660706
117,1
A0932729583PA
A0930668240
184,7
307,6
A0932431188
A0930687779
186,2
308,1
A0932025074
A0930688780
186,4
308,7
A0930784818
Annr14018301
191,9
310,6
Annr14018301
A09r3644807
192,9
A09r3754199
A09r3655746
311,3
194,7
A09r3826871
A09r3672696
A09r3644807
A09r3698030
197,0
312,4
A09r3769632het
A09r3754199
197,8
313,6
C0835316120het
A09r3722852
199,7
314,9
C0835317039het
A09r3795273
200,0
317,4
C0835309219het
A09r3849715
200,3
C0835430413het
A09r3917158
203,3
318,2
C0835614441het
A09r3826871
320,0
204,0
Cnnr78925785het
A0932205577
329,8
C0836224998het
A0932618215
336,8
205,3
C0836337029het
A0932845031PA
342,2
C0835798934het
A0932729583
207,1
350,5
C0836510627het
A0932733945
208,4
C08r4434216het
A0932661635
351,5
C08r4402519het
Annr16729005
208,8
354,3
C0836704060het
Annr13186635
209,1
362,0
C0837579227het
211,3
A0933421857
362,4
Cnnr8148736
A0933561852
365,4
215,9
Cnnr8290395
Cnnr8148736
368,3
Cnnr8290395

## Slide 10
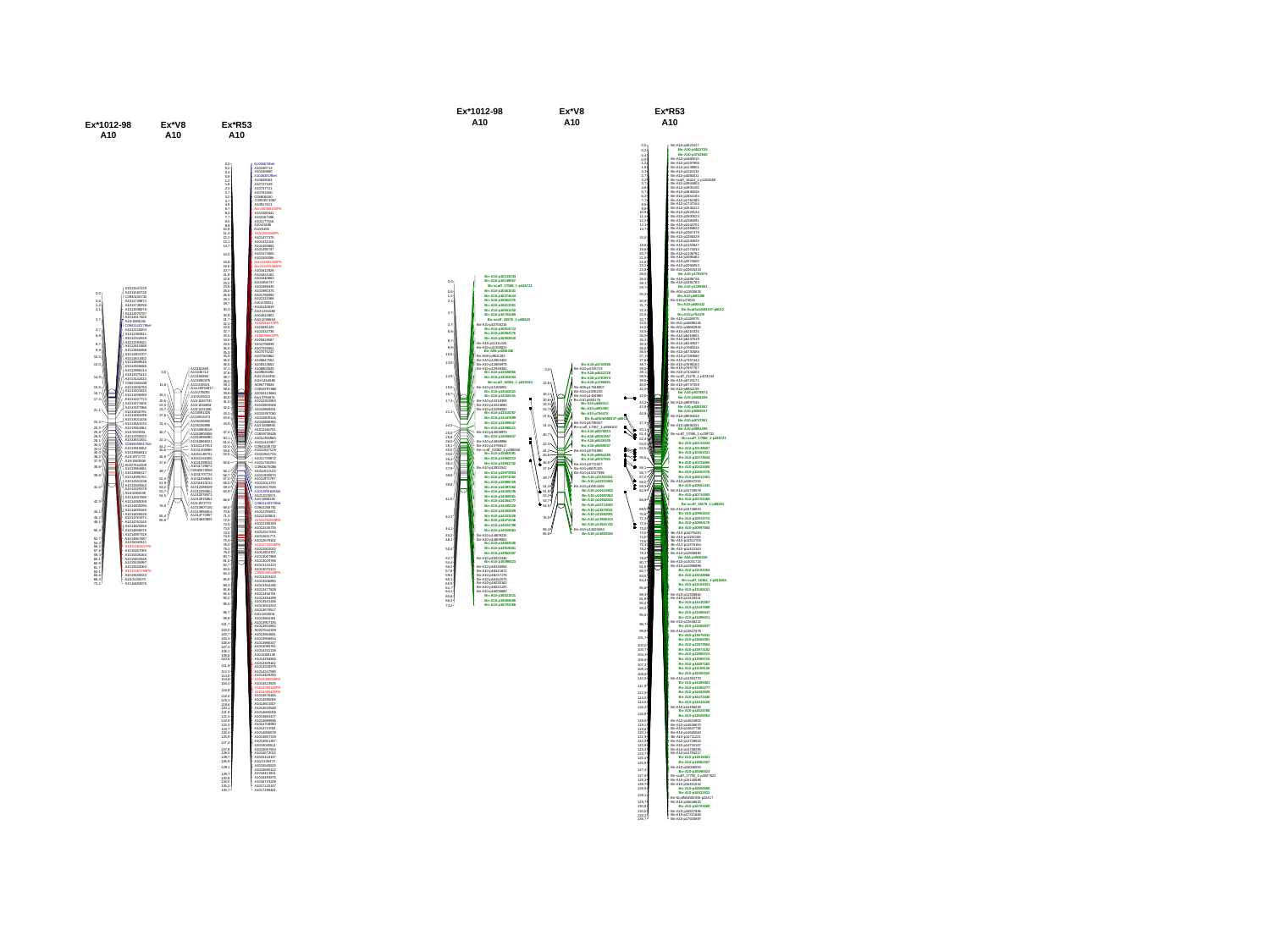

Ex*1012-98
A10
Ex*V8
A10
Ex*R53
A10
Bn-A10-p4821527
0,0
Bn-A10-p4622728
0,2
Bn-A10-p4742940
0,4
Bn-A10-p4480010
0,9
Bn-A10-p4197906
1,3
Bn-A10-p4138801
1,8
Bn-A10-p4118433
2,3
Bn-A10-p4080011
2,7
Bn-scaff_16414_1-p1208159
3,2
Bn-A10-p3944803
3,7
Bn-A10-p3905100
4,8
Bn-A10-p3845546
5,7
Bn-A10-p2852183
6,2
Bn-A10-p2762385
7,7
Bn-A10-p2747164
8,5
Bn-A10-p2545412
9,8
Bn-A10-p2529144
10,9
Bn-A10-p2500523
11,4
Bn-A10-p2385591
12,2
Bn-A10-p2444751
13,1
Bn-A10-p2369622
14,7
Bn-A10-p2367479
Bn-A10-p2266029
15,5
Bn-A10-p2240635
Bn-A10-p2153847
18,8
Bn-A10-p2174043
19,6
Bn-A10-p2106781
20,7
Bn-A10-p2096482
21,8
Bn-A10-p2074000
22,6
Bn-A10-p2056053
23,2
Bn-A02-p23925334
23,8
Bn-A10-p1791974
26,0
Bn-A10-p1698746
26,5
Bn-A10-p1582783
28,1
Bn-A10-p1196851
29,7
Bn-A04-p12600635
30,3
Bn-A10-p693390
Bn-A10-p73025
30,9
Bn-A10-p688412
31,7
Bn-Scaffold000337-p6012
32,3
Bn-A10-p754475
32,5
Bn-A10-p1108975
32,7
Bn-A01-p18896245
33,5
Bn-A01-p18662925
34,0
Bn-A10-p5210325
34,5
Bn-A10-p6418601
35,0
Bn-A10-p6247519
35,3
Bn-A10-p6240637
35,5
Bn-A10-p7065016
36,0
Bn-A10-p6730588
36,5
Bn-A10-p7260660
37,1
Bn-A10-p7357442
37,6
Bn-A10-p10133034
Bn-A10-p10149557
0,0
Bn-scaff_17088_1-p445721
Bn-A10-p10361031
0,6
Bn-A10-p10374644
1,2
Bn-A10-p10562376
3,1
Bn-A10-p10613361
Bn-A10-p10651432
3,7
Bn-A10-p10703469
Bn-scaff_15576_1-p89263
Bn-A10-p10755216
4,7
Bn-A10-p10914713
6,8
Bn-A10-p10954176
Bn-A10-p10902810
8,7
Bn-A10-p11311425
Bn-A10-p11358033
9,9
Bn-A08-p4936436
10,5
Bn-A08-p4902282
Bn-A10-p12863402
13,0
Bn-A10-p12885978
Bn-A10-p12938355
Bn-A10-p13038966
14,9
Bn-A10-p13104354
Bn-scaff_16362_1-p815264
Bn-A10-p13165001
15,5
Bn-A10-p13162021
16,7
Bn-A10-p13165101
Bn-A10-p13218309
17,4
Bn-A10-p13243690
Bn-A10-p13299250
21,1
Bn-A10-p13410287
Bn-A10-p13447899
Bn-A10-p13495547
24,3
Bn-A10-p13498211
Bn-A10-p13508970
25,0
Bn-A10-p13606837
25,8
Bn-A10-p13659996
26,5
Bn-A10-p13796417
28,1
Bn-scaff_22082_1-p296550
30,5
Bn-A10-p13882091
33,0
Bn-A10-p13965313
35,4
Bn-A10-p13941742
36,2
Bn-A10-p13932541
37,9
Bn-A10-p13973954
38,6
Bn-A10-p13973182
Bn-A10-p13995725
39,8
Bn-A10-p14097282
Bn-A10-p14159136
Bn-A10-p14365581
41,0
Bn-A10-p14384277
Bn-A10-p14300320
Bn-A10-p14402509
Bn-A10-p14433226
42,3
Bn-A10-p14472446
Bn-A10-p14524788
44,1
Bn-A10-p14525063
Bn-A10-p14809234
45,2
Bn-A10-p14809502
46,1
Bn-A10-p14883536
Bn-A10-p14919461
50,4
Bn-A10-p14962307
Bn-A10-p15022346
52,7
Bn-A10-p15096523
54,4
Bn-A10-p16326058
56,2
Bn-A10-p16421672
57,8
Bn-A10-p16237178
59,3
Bn-A10-p7698184
38,7
Bn-A10-p7657767
39,0
Bn-A10-p7444203
39,1
Bn-scaff_21276_1-p574134
39,5
Bn-A10-p8723171
39,8
Bn-A10-p8737359
40,0
Bn-A10-p8951109
41,0
Bn-A10-p9378974
42,0
Bn-A10-p9436205
Bn-A10-p8997545
43,2
Bn-A10-p9381557
43,8
Bn-A10-p9459037
44,9
Bn-A10-p9594548
Bn-A10-p9747551
47,4
Bn-A10-p9696331
Bn-A10-p9854395
50,1
Bn-scaff_17088_1-p239732
51,4
Bn-scaff_17088_1-p445721
52,4
Bn-A10-p10133034
53,0
Bn-A10-p10149557
53,5
Bn-A10-p10361031
Bn-A10-p10374644
55,6
Bn-A10-p10331665
Bn-A10-p10423500
56,1
Bn-A10-p10562376
56,7
Bn-A10-p10613361
57,2
Bn-A10-p10647252
58,0
Bn-A10-p10651432
59,3
Bn-A10-p10728078
62,9
Bn-A10-p10714593
Bn-A10-p10703469
66,8
Bn-scaff_15576_1-p89263
Bn-A10-p10748653
69,0
Bn-A10-p10902810
70,6
Bn-A10-p10914713
71,3
Bn-A10-p10954176
72,5
Bn-A10-p10997884
73,0
Bn-A10-p11075240
73,5
Bn-A10-p11192168
74,0
Bn-A10-p11312705
74,5
Bn-A10-p11376164
75,3
Bn-A10-p11423143
76,2
Bn-A10-p12856990
78,3
Bn-A08-p4936436
79,0
Bn-A10-p13031723
80,7
Bn-A10-p12996989
81,5
Bn-A10-p13104354
82,7
Bn-A10-p13038966
83,5
Bn-scaff_16362_1-p815264
84,3
Bn-A10-p13162021
85,6
Bn-A10-p13165101
Bn-A10-p13308862
89,3
Bn-A10-p13429321
91,8
Bn-A10-p13410287
92,4
Bn-A10-p4742940
Bn-A10-p4725713
0,0
Bn-A10-p4622728
Bn-A10-p1791974
Bn-A10-p1196851
13,8
Bn-A08-p17848907
Bn-A10-p1595235
18,1
Bn-A10-p1484980
Bn-A10-p585176
20,6
Bn-A10-p688412
23,3
Bn-A10-p693390
23,7
Bn-A10-p754475
27,6
Bn-Scaffold000337-p6012
Bn-A10-p5799347
31,4
Bn-scaff_17367_1-p866103
Bn-A10-p9378974
40,7
Bn-A10-p9381557
Bn-A10-p9436205
42,3
Bn-A10-p9459037
Bn-A10-p9751890
44,2
Bn-A10-p9854395
45,6
Bn-A10-p9747551
46,8
Bn-A10-p9772527
Bn-A10-p9675325
47,6
Bn-A10-p10327896
Bn-A10-p10361031
49,7
Bn-A10-p10331665
Bn-A10-p10454385
51,4
Bn-A10-p10423500
51,9
Bn-A10-p10997884
53,2
Bn-A10-p10902810
53,7
Bn-A10-p10714593
54,5
Bn-A10-p13875041
Bn-A10-p13882091
76,0
Bn-A10-p13965313
Bn-A10-p13941742
Bn-A10-p14820463
85,4
Bn-A10-p14883536
85,8
Bn-A10-p16162575
60,1
Bn-A10-p16030162
60,9
Bn-A10-p16032125
61,7
Bn-A10-p16005880
64,1
Bn-A10-p16021831
65,6
Bn-A10-p15605556
66,3
Bn-A10-p16703268
73,2
Bn-A10-p13447899
93,2
Bn-A10-p13495547
95,5
Bn-A10-p13498211
Bn-A10-p13638222
96,7
Bn-A10-p13606837
Bn-A10-p13827678
99,8
Bn-A10-p13875041
101,7
Bn-A10-p13882091
Bn-A10-p13973954
103,0
Bn-A10-p13973182
103,7
Bn-A10-p13965313
104,3
Bn-A10-p13995725
106,6
Bn-A10-p14097282
107,3
Bn-A10-p14159136
108,1
Bn-A10-p14300320
108,6
Bn-A10-p14353770
110,5
Bn-A10-p14365581
111,9
Bn-A10-p14384277
Bn-A10-p14402509
112,3
Bn-A10-p14472446
114,0
Bn-A10-p14433226
114,8
Bn-A10-p14490038
116,4
Bn-A10-p14524788
116,8
Bn-A10-p14525063
Bn-A10-p14623802
118,0
Bn-A10-p14636670
119,1
Bn-A10-p14647792
119,6
Bn-A10-p14665064
120,1
Bn-A10-p14711221
121,9
Bn-A10-p14728923
122,4
Bn-A10-p14734107
122,8
Bn-A10-p14756285
123,3
Bn-A10-p14794217
123,7
Bn-A10-p14919461
125,4
Bn-A10-p14962307
125,9
Bn-A10-p15006053
127,4
Bn-A10-p15096523
Bn-scaff_17750_1-p2007623
127,8
Bn-A10-p15140586
128,3
Bn-A10-p16451834
128,7
Bn-A10-p15605556
128,9
Bn-A10-p16021831
129,1
Bn-Scaffold000306-p22417
Bn-A10-p16626615
129,7
Bn-A10-p16703268
130,8
Bn-A10-p16927895
133,0
Bn-A10-p17321665
135,2
Bn-A10-p17500897
135,7
Ex*1012-98
A10
Ex*V8
A10
Ex*R53
A10
A1056676het
0,0
A10248713
0,2
A10150860
0,4
A10363819het
0,9
A10638064
1,3
A10727329
1,8
A10747721
2,3
A10781500
2,7
C05806180
3,2
C0353671067
3,7
A10917621
4,8
Annr36369222PA
5,7
A101080441
6,2
A101167496
7,7
A101177516
8,5
A10r26186
9,8
A10r5195
10,9
A101381556PA
11,4
A101477375
12,2
A101432110
13,1
A101493884
14,7
A101495747
A101572605
15,5
A101592086
Annr13391320PA
18,8
Annr13415364PA
19,6
A101612829
20,7
A101624161
21,8
A101640663
22,6
A101656737
23,2
A101889430
23,8
A101980375
26,0
A101795993
26,5
A102121566
28,1
A10r239321
29,7
A104140819
30,3
A10r1331086
A104542801
30,9
A10r1036654
31,7
A102916473PA
32,3
A102691425
32,5
A102432736
32,7
A106098602PA
33,5
A105819587
34,0
A104798839
34,5
A107249551
35,0
A107075242
35,3
A107065862
35,5
A108647552
36,0
A108323653
36,5
A108903549
37,1
A109020292
37,6
A1011547129
A1011562735
0,0
C0940236742
A1011729872
0,6
A1011745250
1,2
A1011938070
3,1
A1011975797
A1012017625
3,7
A10r1868156
C0941140179het
A1012115003
4,7
A1012305811
6,8
A1012342619
A1012293821
8,7
A1012610488
A1012660896
9,9
A1012804707
10,5
A1012841553
A1012908615
13,0
A1012926685
A1012986214
A1013075112
14,9
A1013142413
C0943348439
A1013206793
15,5
A1013203423
16,7
A1013206893
A1013247713
17,4
A1013273400
A1013337986
21,1
A1013456791
A1013494299
A1013531436
24,3
A1013553233
A1013564961
25,0
A10r1933551
25,8
A1013700523
26,5
A1013831831
28,1
C0944559517het
30,5
A1013915852
33,0
A1013956814
35,4
A10r1972772
36,2
A10r1963559
37,9
A0327544109
38,6
A1013964681
A1013986317
39,8
A1014095761
A1014152138
A1014309162
41,0
A1014329379
A10r2069149
A1014347590
A1014380059
42,3
A1014409294
A1014458160
44,1
A1014458435
A1014761871
45,2
A1014762140
46,1
A1014820935
A1014856578
50,4
A1014897319
A1014967697
52,7
A1015040511
54,4
A1015262607PA
56,2
A1015167085
57,8
A1015346264
59,3
A10161850
A10248713
0,0
A10r1524355
38,7
A10r1454586
39,0
A095779828
39,1
C0936797860
39,5
A1010123684
39,8
Annr3794874
40,0
A1010352963
41,0
A1010805558
42,0
A1010869231
A1010397090
43,2
A1010808116
43,8
A1010898392
44,9
A10r1838806
A1011146701
47,4
C0939728625
A1011300985
50,1
A1011410387
51,4
C0940236742
52,4
A1011547129
53,0
A1011562735
53,5
A1011729872
A1011745250
55,6
C0940575065
A1011813132
56,1
A1011938070
56,7
A1011975797
57,2
A1012013370
58,0
A1012017625
59,3
A1012091626het
62,9
A1012076571
A10r1868156
66,8
C0941140179het
C0941208761
69,0
A1012293821
70,6
A1012305811
71,3
A1012342619PA
72,5
A1012388329
73,0
A1012436735
73,5
A1012527034
74,0
A1012611771
74,5
A1012679162
75,3
A1012742926PA
76,2
A1012902002
78,3
A1012804707
79,0
A1013067969
80,7
A1013029766
81,5
A1013142413
82,7
A1013075112
83,5
C0943348439PA
84,3
A1013203423
85,6
A1013206893
A1013354246
89,3
A1013477829
91,8
A1013456791
92,4
A10150860
A101980375
A10r239321
13,8
Annr19254917
A10r279293
18,1
A102109113
A10r1040700
20,6
A10r1036654
23,3
A10r1331086
23,7
A102691425
27,6
A102916473
A105436592
31,4
A105436896
A1010808116
40,7
A1010805558
A1010898392
42,3
A1010869231
A1011147914
44,2
A1011300985
45,6
A1011146701
46,8
A1011161805
A1011099104
47,6
A1011729872
C0940575065
49,7
A1011707724
A1011834653
51,4
A1011813132
51,9
A1012388329
53,2
A1012293821
53,7
A1012076571
54,5
A1013915852
A10r1972772
76,0
A1013907135
A1013956814
A1014772957
85,4
A1014820935
85,8
A1015402638
60,1
A1015535997
60,9
A1015534062
61,7
A1015561799PA
64,1
A1015545533
65,6
A10r2135473
66,3
A1016493875
73,2
A1013494299
93,2
A1013531436
95,5
A1013553233
A1013678517
96,7
A10r1933551
A1013864381
99,8
A1013907135
101,7
A1013915852
A0327544109
103,0
A1013964681
103,7
A1013956814
104,3
A1013986317
106,6
A1014095761
107,3
A1014152138
108,1
A10r2069149
108,6
A1014294904
110,5
A1014309162
111,9
A1014329379
A1014347590
112,3
A1014409294
114,0
A1014380059PA
114,8
A1014423505
116,4
A1014458160PA
116,8
A1014458435PA
A1014576445
118,0
A1014590065
119,1
A1014601507
119,6
A1014620549
120,1
A1014665006
121,9
A1014684427
122,4
A1014689598
122,8
A1014708990
123,3
A1014747091
123,7
A1014856578
125,4
A1014897319
125,9
A1014951387
127,4
A1015040511
A1015067554
127,8
A1015072022
128,3
A1015143157
128,7
A10r2135473
128,9
A1015545533
129,1
A1015685122
A1016421911
129,7
A1016493875
130,8
A1016733209
133,0
A1017125147
135,2
A1017299401
135,7

## Slide 11
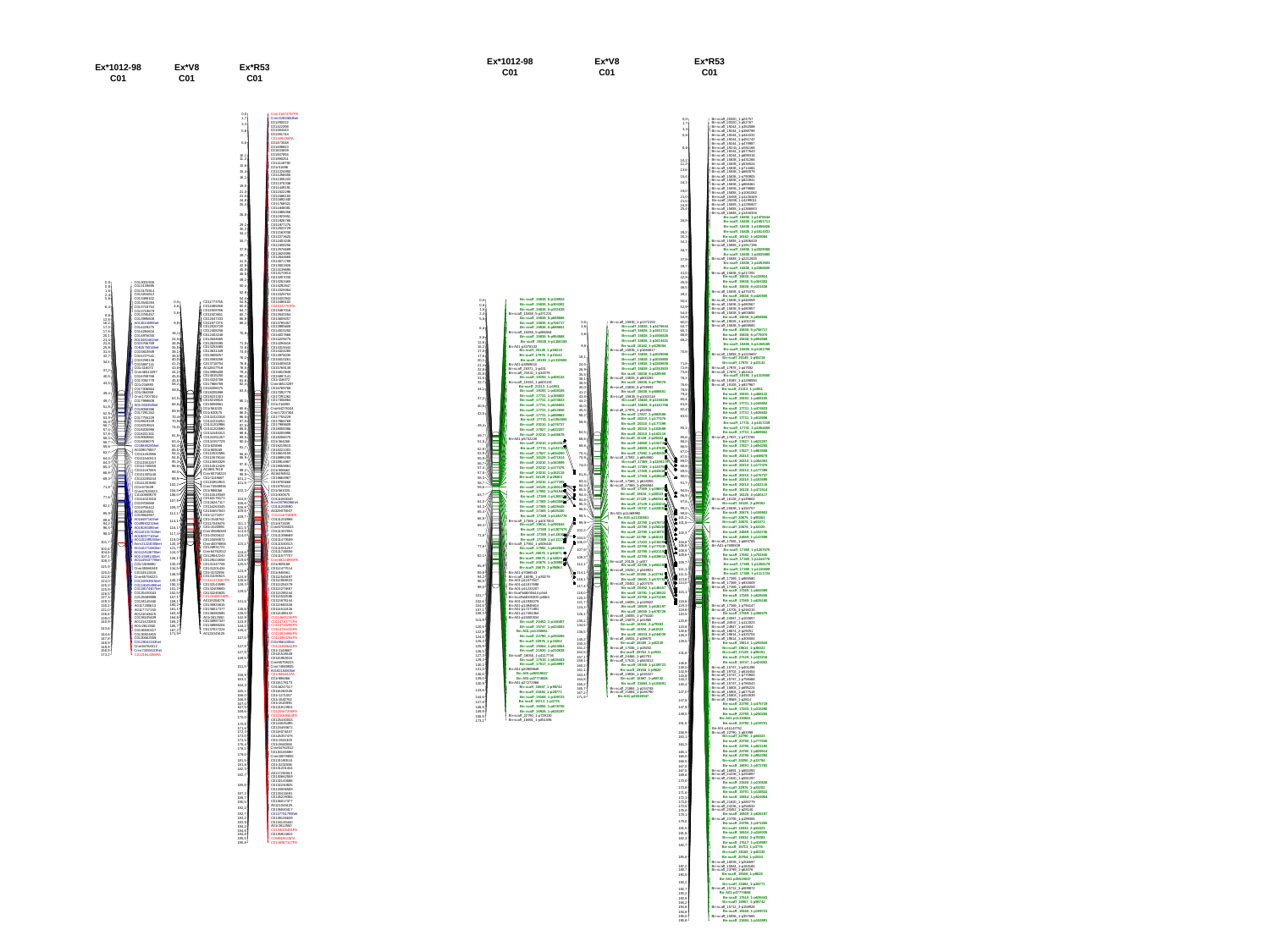

Ex*1012-98
C01
Ex*V8
C01
Ex*R53
C01
Bn-scaff_20320_1-p24757
0,0
Bn-scaff_20320_1-p52767
1,7
Bn-scaff_19244_1-p352588
3,3
Bn-scaff_19244_1-p388798
Bn-scaff_19244_1-p444332
5,6
Bn-scaff_19244_1-p451742
Bn-scaff_19244_1-p479987
Bn-scaff_19244_1-p551168
6,6
Bn-scaff_19244_1-p577643
Bn-scaff_19244_1-p699318
Bn-scaff_15838_1-p431266
10,1
Bn-scaff_15838_1-p536524
11,2
Bn-scaff_15838_1-p714484
13,6
Bn-scaff_15838_1-p680079
Bn-scaff_15838_1-p790905
15,4
Bn-scaff_15838_1-p822841
16,1
Bn-scaff_15838_1-p868461
Bn-scaff_15838_1-p979888
19,0
Bn-scaff_15838_1-p1061062
Bn-scaff_15838_1-p1126349
21,0
Bn-scaff_15838_1-p1199011
21,5
Bn-scaff_15838_1-p1295927
24,9
Bn-scaff_15838_1-p1366843
25,4
Bn-scaff_15838_1-p1440326
Bn-scaff_15838_1-p1476644
26,8
Bn-scaff_15838_1-p1551711
Bn-scaff_15838_1-p1556826
Bn-scaff_15838_1-p1614431
29,2
Bn-scaff_16182_1-p629084
30,3
Bn-scaff_15838_1-p1806419
34,1
Bn-scaff_15838_1-p1917196
Bn-scaff_15838_1-p2029088
34,7
Bn-scaff_15838_1-p2035980
Bn-scaff_15838_1-p2212925
37,9
Bn-scaff_15838_1-p2253503
39,7
Bn-scaff_15838_1-p2268600
Bn-scaff_15838_5-p217291
41,5
Bn-scaff_15838_5-p128924
42,9
Bn-scaff_15838_5-p304302
45,9
Bn-scaff_15838_5-p331638
46,5
Bn-scaff_15838_5-p375370
49,2
Bn-scaff_15838_5-p420555
Bn-scaff_15838_5-p446059
50,4
Bn-scaff_15838_5-p480567
52,9
Bn-scaff_15838_5-p480957
Bn-scaff_15838_5-p603655
54,4
Bn-scaff_15838_5-p658666
54,9
Bn-scaff_19026_1-p101219
60,0
Bn-scaff_15838_5-p659580
64,7
Bn-scaff_15838_5-p706717
65,7
Bn-scaff_15838_5-p779070
66,8
Bn-scaff_15838_5-p954588
68,2
Bn-scaff_15838_5-p1158185
Bn-scaff_15838_5-p1181758
70,8
Bn-scaff_15838_5-p1215697
Bn-scaff_25149_1-p55218
Bn-scaff_17978_1-p23142
71,8
Bn-scaff_17978_1-p47592
72,8
Bn-scaff_17978_1-p52413
73,8
Bn-scaff_19193_1-p1319500
76,2
Bn-scaff_19193_1-p1288594
Bn-scaff_19193_1-p927967
76,6
Bn-scaff_15838_5-p128924
0,0
Bn-scaff_15838_5-p304302
0,8
Bn-scaff_15838_5-p331638
1,6
Bn-scaff_15838_5-p371231
2,4
Bn-scaff_15838_5-p658666
5,6
Bn-scaff_15838_5-p706717
Bn-scaff_15838_5-p885651
6,4
Bn-scaff_15838_5-p886564
Bn-scaff_15838_5-p954588
8,8
Bn-scaff_15838_5-p1158185
12,6
Bn-A01-p3378332
16,2
Bn-scaff_25149_1-p55218
17,0
Bn-scaff_17978_1-p23142
17,6
Bn-scaff_19193_1-p1319500
20,1
Bn-A01-p3859610
21,4
Bn-scaff_28371_1-p431
22,8
Bn-scaff_23432_1-p10078
25,8
Bn-scaff_19193_1-p488132
31,5
Bn-scaff_19193_1-p603133
32,7
Bn-scaff_21313_1-p1951
34,1
Bn-scaff_19193_1-p408105
Bn-scaff_17731_1-p165802
37,2
Bn-scaff_17731_1-p474833
Bn-scaff_17731_1-p536602
40,5
Bn-scaff_17731_1-p812656
43,5
Bn-scaff_17731_1-p889862
Bn-scaff_17731_1-p1054055
Bn-scaff_20210_1-p276737
49,4
Bn-scaff_17827_1-p622207
Bn-scaff_20210_1-p448678
49,7
Bn-A01-p5732226
51,8
Bn-scaff_20210_1-p384363
Bn-scaff_17731_1-p1017239
52,9
Bn-scaff_17827_1-p654250
53,9
Bn-scaff_16128_1-p471514
55,9
Bn-scaff_20210_1-p163599
56,7
Bn-scaff_20210_1-p177476
57,4
Bn-scaff_20210_1-p162115
57,8
Bn-scaff_16128_2-p29363
58,3
Bn-scaff_20210_1-p177399
58,7
Bn-scaff_16128_2-p180117
59,6
Bn-scaff_17592_1-p781545
63,7
Bn-scaff_17369_1-p1208179
Bn-scaff_17369_1-p843089
64,0
Bn-scaff_17369_1-p829445
64,3
Bn-scaff_17369_1-p625180
65,4
Bn-scaff_17369_1-p1244776
66,8
Bn-scaff_17369_1-p1017003
Bn-scaff_19614_1-p292844
69,3
Bn-scaff_17369_1-p1207575
Bn-scaff_17369_1-p1130809
71,8
Bn-scaff_17369_1-p1111724
Bn-scaff_17592_1-p506418
77,6
Bn-scaff_17592_1-p642504
Bn-scaff_20675_1-p61871
82,1
Bn-scaff_20675_1-p32025
Bn-scaff_20675_1-p159903
85,9
Bn-scaff_20675_1-p95063
Bn-scaff_15838_1-p1372150
0,0
Bn-scaff_15838_1-p1476644
4,6
Bn-scaff_15838_1-p1551711
5,6
Bn-scaff_15838_1-p1556826
Bn-scaff_15838_1-p1614431
Bn-scaff_16182_1-p629084
9,8
Bn-scaff_15838_1-p1684617
Bn-scaff_15838_1-p2029088
18,1
Bn-scaff_15838_1-p2035980
Bn-scaff_15838_1-p2268600
24,5
Bn-scaff_15838_1-p2253503
26,9
Bn-scaff_15838_5-p420555
35,5
Bn-scaff_15838_5-p800293
38,1
Bn-scaff_15838_5-p779070
38,5
Bn-scaff_15838_5-p759592
40,0
Bn-scaff_15838_5-p885651
41,2
Bn-scaff_15838_5-p1043118
43,8
Bn-scaff_15838_5-p1158185
44,2
Bn-scaff_15838_5-p1181758
45,0
Bn-scaff_17978_1-p52856
45,5
Bn-scaff_17827_1-p963588
58,2
Bn-scaff_20210_1-p177476
59,8
Bn-scaff_20210_1-p177399
Bn-scaff_20210_1-p163599
63,5
Bn-scaff_20210_1-p162115
Bn-scaff_16128_2-p29363
68,6
Bn-scaff_24869_1-p152736
69,8
Bn-scaff_24869_1-p147699
Bn-scaff_17592_1-p642504
70,4
Bn-scaff_17592_1-p654560
70,9
Bn-scaff_17369_1-p1208179
74,0
Bn-scaff_17369_1-p1207575
Bn-scaff_17369_1-p843089
81,8
Bn-scaff_17369_1-p829445
Bn-scaff_17369_1-p818355
83,4
Bn-scaff_17369_1-p366584
84,4
Bn-scaff_17369_1-p386575
85,5
Bn-scaff_19614_1-p36023
94,4
Bn-scaff_27129_1-p98351
94,8
Bn-scaff_27129_1-p103216
95,3
Bn-scaff_15747_1-p424053
96,6
Bn-A01-p11469992
98,5
Bn-A01-p11330661
Bn-scaff_22790_1-p475719
99,9
Bn-scaff_22790_1-p250265
102,2
Bn-scaff_22790_1-p219701
Bn-scaff_22790_1-p84023
104,5
Bn-scaff_17203_1-p315290
106,0
Bn-scaff_22790_1-p777045
107,9
Bn-scaff_22790_1-p821192
Bn-scaff_22790_1-p829614
109,3
Bn-scaff_20126_1-p457
112,1
Bn-scaff_22790_1-p952392
Bn-scaff_20250_1-p348921
114,1
Bn-scaff_20250_2-p12794
Bn-scaff_16691_1-p572782
116,1
Bn-scaff_20452_1-p207078
117,4
Bn-scaff_20452_1-p146457
Bn-scaff_21313_1-p1951
78,5
Bn-scaff_19193_1-p488132
79,4
Bn-scaff_19193_1-p408105
80,4
Bn-scaff_17731_1-p165802
81,8
Bn-scaff_17731_1-p474833
82,4
Bn-scaff_17731_1-p536602
83,5
Bn-scaff_17731_1-p812656
Bn-scaff_17731_1-p1017239
Bn-scaff_17731_1-p1054055
85,1
Bn-scaff_17731_1-p889862
Bn-scaff_17827_1-p372765
85,6
Bn-scaff_17827_1-p622207
86,0
Bn-scaff_17827_1-p654250
86,5
Bn-scaff_17827_1-p963588
87,0
Bn-scaff_20210_1-p448678
87,5
Bn-scaff_20210_1-p384363
88,0
Bn-scaff_20210_1-p177476
88,8
Bn-scaff_20210_1-p177399
89,5
Bn-scaff_20210_1-p276737
90,0
Bn-scaff_20210_1-p163599
91,7
Bn-scaff_20210_1-p162115
Bn-scaff_16128_1-p471514
94,8
Bn-scaff_16128_2-p180117
96,9
Bn-scaff_16128_2-p149602
97,8
Bn-scaff_16128_2-p29363
Bn-scaff_20638_1-p150757
99,2
Bn-scaff_20675_1-p159903
99,9
Bn-scaff_20675_1-p95063
101,2
Bn-scaff_20675_1-p61871
101,8
Bn-scaff_20675_1-p32025
Bn-scaff_24869_1-p152736
102,3
Bn-scaff_24869_1-p147699
Bn-scaff_17592_1-p683765
104,8
Bn-A01-p7800839
106,6
Bn-scaff_17369_1-p1207575
108,9
Bn-scaff_17592_1-p781545
109,6
Bn-scaff_17369_1-p1244776
109,7
Bn-scaff_17369_1-p1208179
Bn-scaff_17369_1-p1130809
111,1
Bn-scaff_17369_1-p1111724
111,5
Bn-scaff_17369_1-p965580
113,6
Bn-scaff_17369_1-p933509
114,0
Bn-scaff_17369_1-p858259
Bn-scaff_17369_1-p843089
115,1
Bn-scaff_17369_1-p829445
Bn-scaff_17369_1-p625180
118,6
Bn-scaff_17369_1-p794147
119,3
Bn-scaff_15705_3-p246215
119,6
Bn-scaff_17369_1-p386575
120,5
Bn-scaff_24947_1-p103657
123,8
Bn-scaff_24947_1-p112023
Bn-scaff_24947_1-p43804
124,8
Bn-scaff_16874_2-p29251
126,6
Bn-scaff_19614_1-p325704
128,3
Bn-scaff_19614_1-p305584
129,5
Bn-scaff_19614_1-p292844
Bn-scaff_19614_1-p36023
Bn-scaff_27129_1-p98351
131,6
Bn-scaff_27129_1-p103216
Bn-scaff_15747_1-p424053
136,6
Bn-scaff_15747_1-p401496
138,0
Bn-scaff_19702_1-p616454
142,9
Bn-scaff_15747_1-p770900
143,8
Bn-scaff_15747_1-p758466
144,2
Bn-scaff_15747_1-p785543
145,4
Bn-scaff_15803_1-p695224
Bn-scaff_15803_1-p677518
147,0
Bn-scaff_15803_1-p452639
Bn-scaff_19569_1-p2814
147,8
Bn-scaff_22790_1-p475719
147,9
Bn-scaff_17203_1-p315290
Bn-scaff_22790_1-p250265
149,5
Bn-A01-p11330661
Bn-scaff_22790_1-p219701
151,5
Bn-A01-p11142752
Bn-scaff_22790_1-p83398
156,9
Bn-scaff_22790_1-p84023
163,1
Bn-scaff_22790_1-p777045
164,3
Bn-scaff_22790_1-p821192
Bn-scaff_22790_1-p829614
165,1
Bn-scaff_22790_1-p952392
166,0
Bn-scaff_20250_2-p12794
166,5
Bn-scaff_16691_1-p572782
167,0
Bn-scaff_16691_1-p881093
167,5
Bn-scaff_24236_1-p293897
169,6
Bn-scaff_21820_1-p591297
170,0
Bn-scaff_21820_1-p210838
Bn-scaff_22976_1-p23202
170,8
Bn-scaff_15791_1-p138522
171,6
Bn-scaff_19364_1-p524064
172,3
Bn-scaff_21820_1-p280779
173,0
Bn-scaff_24236_1-p258532
173,5
Bn-scaff_20452_1-p28140
176,4
Bn-scaff_16929_1-p626197
178,1
Bn-scaff_23795_1-p199305
179,0
Bn-scaff_23795_1-p471265
Bn-scaff_16534_2-p51823
181,5
Bn-scaff_16534_2-p244035
181,8
Bn-scaff_16534_2-p79383
182,3
Bn-scaff_17517_1-p419997
182,7
Bn-scaff_15713_1-p3776
Bn-scaff_28165_1-p40330
Bn-scaff_20764_1-p2534
185,6
Bn-scaff_18039_1-p206597
Bn-scaff_15844_1-p184165
187,2
Bn-scaff_23799_1-p62076
188,7
Bn-scaff_19168_1-p9620
190,5
Bn-A01-p26519047
192,2
Bn-scaff_21884_1-p28771
Bn-scaff_15712_3-p509872
192,7
Bn-A01-p27774666
193,2
Bn-scaff_17515_1-p635443
193,9
Bn-scaff_16567_1-p98742
194,2
Bn-scaff_15712_3-p158928
194,6
Bn-scaff_19168_1-p109723
194,8
Bn-A01-p7086543
89,6
Bn-scaff_16095_1-p30079
94,2
Bn-A01-p11077627
96,5
Bn-A01-p11817698
98,5
Bn-A01-p11233207
Bn-scaff_15791_1-p138522
Bn-Scaffold000444-p344
118,0
101,7
Bn-Scaffold000300-p4564
Bn-scaff_23795_1-p471265
120,3
Bn-A01-p11938379
102,6
Bn-scaff_16055_1-p119822
121,7
Bn-A01-p14948614
104,6
Bn-scaff_16929_1-p626197
124,3
Bn-A01-p17271891
107,1
Bn-scaff_16055_1-p678726
Bn-A01-p17494364
108,3
126,1
Bn-scaff_16055_1-p776100
Bn-A01-p15000034
115,9
Bn-scaff_15879_1-p31965
130,2
Bn-scaff_20452_1-p146457
Bn-scaff_16534_2-p79383
134,5
Bn-scaff_15747_1-p424053
120,5
Bn-scaff_16534_2-p51823
Bn-A01-p11330661
122,8
136,5
Bn-scaff_16534_2-p244035
Bn-scaff_22790_1-p250265
124,0
Bn-scaff_16534_2-p38670
145,2
Bn-scaff_22976_1-p23202
125,3
Bn-scaff_28165_1-p40330
150,3
Bn-scaff_19364_1-p524064
125,9
Bn-scaff_17036_1-p19202
151,2
Bn-scaff_21820_1-p210838
126,5
Bn-scaff_20764_1-p2534
152,5
Bn-scaff_16055_1-p1117736
127,3
Bn-scaff_24466_1-p62701
157,1
Bn-scaff_17515_1-p635443
129,3
Bn-scaff_17515_1-p583013
159,1
Bn-scaff_17517_1-p419997
130,2
Bn-scaff_19168_1-p109723
160,2
Bn-A01-p20909649
131,0
Bn-scaff_19168_1-p9620
161,1
Bn-A01-p26519047
136,6
Bn-scaff_15936_1-p245327
163,4
Bn-A01-p27774666
Bn-scaff_16567_1-p98742
139,0
164,8
Bn-A01-p27272066
140,9
Bn-scaff_21884_1-p153891
165,2
Bn-scaff_16567_1-p98742
Bn-scaff_21884_1-p154748
165,7
143,6
Bn-scaff_21884_1-p28771
Bn-scaff_21884_1-p185790
167,2
Bn-A01-p26519047
Bn-scaff_19168_1-p109723
171,9
144,6
Bn-scaff_15713_1-p3776
147,8
Bn-scaff_16055_1-p678726
148,9
Bn-scaff_16929_1-p626197
149,9
Bn-scaff_22790_1-p729130
158,9
Bn-scaff_16691_1-p851595
173,2
Bn-scaff_15936_1-p357665
195,0
Bn-scaff_21884_1-p153891
195,8
Ex*1012-98
C01
Ex*V8
C01
Ex*R53
C01
Cnnr21874797PA
0,0
Cnnr21903658het
1,7
C01290222
3,3
C01322398
C01384343
5,6
C01391744
C01409330PA
C01473548
6,6
C01498823
C01633839
C01937855
10,1
C01990251
11,2
C011148790
13,6
C01r51696
C011224850
15,4
C011256655
16,1
C011305222
C011375336
19,0
C011449191
C011522296
21,0
C011588183
21,5
C011692440
24,9
C011768521
25,4
C011846081
C011885058
26,8
C011923651
C011928766
C011977275
29,2
C012020729
30,3
C012162038
34,1
C012273625
C012403248
34,7
C012408256
C012576689
37,9
C012629395
39,7
C012644665
C013071789
41,5
C013001926
42,9
C013139695
45,9
C013170914
46,5
C013207030
49,2
C013253465
C013292847
50,4
C013328364
52,9
C013328753
C013432943
54,4
C013486102
54,9
C043322757PA
60,0
C013487016
64,7
C013540284
65,7
C013609257
66,8
C013785457
68,2
C013995608
C014015250
70,8
C014037868
C014229275
C014294616
71,8
C014319444
72,8
C014324265
73,8
C014976030
76,2
C015010261
C015409419
76,6
C013001926
0,0
C013139695
0,8
C013170914
1,6
C013204813
2,4
C013486102
5,6
C013540284
C013718754
6,4
C013719679
C013785457
8,8
C013995608
12,6
A013044599het
16,2
C014229275
17,0
C014294616
17,6
C014976030
20,1
A013482461het
21,4
C015766769
22,8
C081576516het
25,8
C015810949
31,5
C015727541
32,7
C015769138
34,1
C015897141
C01r116072
37,2
Cnnr46513297
C016769766
40,5
C017092779
43,5
C01r216893
C017306864
C01r364268
49,4
Cnnr17207304
C017999609
49,7
A01r350458het
51,8
C018058386
C017291262
52,9
C017755229
53,9
C018619159
55,9
C018219815
56,7
C018205998
57,4
C018221301
57,8
C019059861
58,3
C018206075
58,7
C018885285het
59,6
A0329075937
63,7
C0111202986
C0111540313
64,0
C0111551257
64,3
C0111746556
65,4
C0111167065
66,8
C0111305146
C0113285244
69,3
C0111203590
C01r672639
71,8
Cnnr67535023
C0110869579
77,6
C0111023318
C019700668
82,1
C019755412
A016294551
85,9
C019664967
C011773755
0,0
C011885058
4,6
C011928766
5,6
C011923651
C012047333
C011977275
9,8
C012020729
C012408256
18,1
C012403248
C012644665
24,5
C012629395
26,9
C013253465
35,5
C013631449
38,1
C013609257
38,5
C013593256
40,0
C013718754
41,2
A012847759
43,8
C013995608
44,2
C014015250
45,0
C014324708
45,5
C017866769
58,2
C018206075
59,8
C018205998
C018221301
63,5
C018219815
C019059861
68,6
C01r561025
69,8
C01r630575
C0111023318
70,4
C0111034251
70,9
C0111202986
74,0
C0111203590
C0111540313
81,8
C0111551257
C0111557729
83,4
C01r825966
84,4
C01r809168
85,5
C0113532596
94,4
C0113678144
94,8
C0113683328
95,3
C0114512426
96,6
A019817818
98,5
Cnnr65758223
C01r1148667
99,9
C0115953915
102,2
Cnnr74949935
C01r986066
104,5
C0115519349
106,0
C0116178173
107,9
C0116247117
C0116263345
109,3
C0116657043
112,1
C01r1271057
C01r1540762
114,1
C0117049476
C01r1520990
116,1
Cnnr45586349
117,4
C01r2501622
C015769138
78,5
C015810949
79,4
C015897141
80,4
C01r116072
81,8
Cnnr46513297
82,4
C016769766
83,5
C017092779
C017291262
C017306864
85,1
C01r216893
Cnnr64278104
85,6
Cnnr17207304
86,0
C017755229
86,5
C017866769
87,0
C017999609
87,5
C018058386
88,0
C018205998
88,8
C018206075
89,5
C01r364268
90,0
C018219815
91,7
C018221301
C018619159
94,8
C018885285
96,9
C018914987
97,8
C019059861
C01r506662
99,2
A016294551
99,9
C019664967
101,2
C019700668
101,8
C019755412
C01r561025
102,3
C01r630575
C0111063040
104,8
Annr39796066het
106,6
C0111203590
108,9
A0329075937
109,6
C0111167065PA
109,7
C0111202986
C01r672639
111,1
Cnnr67535023
111,5
C0111367853
113,6
C0111398669
114,0
C0111473509
C0111540313
115,1
C0111551257
C0111746556
118,6
C0111577707
119,3
Cnnr68224995PA
119,6
C01r809168
120,5
C0112477514
123,8
C01r846561
C0112543497
124,8
C0112804823
126,6
C0113254379
128,3
C0113273867
129,5
C0113285244
C0113532596
C0113678144
131,6
C0113683328
C0114512426
136,6
C0114495132
138,0
C0114685236PA
142,9
C0114730773PA
143,8
C0114718298PA
144,2
C0114745415PA
145,4
C0114916866PA
C0114963294PA
147,0
C01r956100het
C0115388644PA
147,8
C01r1148667
147,9
C0115519349
C0115953915
149,5
Cnnr65758223
Cnnr74949935
151,5
A019218480het
C01r985441PA
156,9
C01r986066
163,1
C0116178173
164,3
C0116247117
C0116263345
165,1
C01r1271057
166,0
C01r1540762
166,5
C01r1520990
167,0
C0121813924
167,5
C0120567208PA
169,6
C0125066964PA
170,0
C0125430043
C0124045495
170,8
C0124493672
171,6
C0118074407
172,3
C0125357478
173,0
C01r1924329
173,5
C01r2663588
176,4
Cnnr64762012
178,1
C0130165590
179,0
Cnnr40078855
C0131190515
181,5
C01r3232596
181,8
C0131201434
182,3
A0117200613
182,7
C0130662559
C0132143598
C0132240825
185,6
C0133006559
C0133411681
187,2
C0135229355
188,7
C0136017377
190,5
A0121043425
192,2
C0136840417
C0137781799het
192,7
C0138105639
193,2
C0134145440
193,9
A01r2612550
194,2
C0138005481PA
194,6
C0135924815
A016487162het
89,6
C049983232het
94,2
A019180495het
96,5
A0110131743het
98,5
A019297710het
C0124493672
118,0
A0110249535het
101,7
Cnnr40078855
120,3
Annr21224005het
C0128931721
121,7
A0110271080het
102,6
Cnnr64762012
124,3
A0112453679het
104,6
C0129042243
A01r1559140het
107,1
126,1
C0129102658
A0114943779het
108,3
C0131347708
130,2
C01r1506080
115,9
C0131201434
134,5
Cnnr45586349
C01r3232596
C0114512426
120,5
136,5
C0131190515
Cnnr65758223
122,8
C0131173367PA
145,2
C0115953915het
124,0
C0132143598
150,3
C0124045495het
125,3
C0132409693
151,2
C0118074407het
125,9
C0132240825
152,5
C0125430043
126,5
C01r3600016PA
157,1
C0129489998
127,3
A0119204076
159,1
C0134145440
129,3
C0135924815
160,2
A0117200613
130,2
C0136017377
161,1
A0117717150
131,0
C0136380585
163,4
A0121043425
136,6
A01r2612550
164,8
C0138105639
139,0
C0136987347
165,2
A0121423280
140,9
C0136988204
165,7
A01r2612550
143,6
C0137027224
167,2
C0136840417
A0121043425
171,9
C0135924815
144,6
C0130662559
147,8
C0129042243het
148,9
Cnnr64762012
149,9
Cnnr73083533het
158,9
C0121844358PA
173,2
194,8
C059508115PA
195,0
C0136987347PA
195,8

## Slide 12
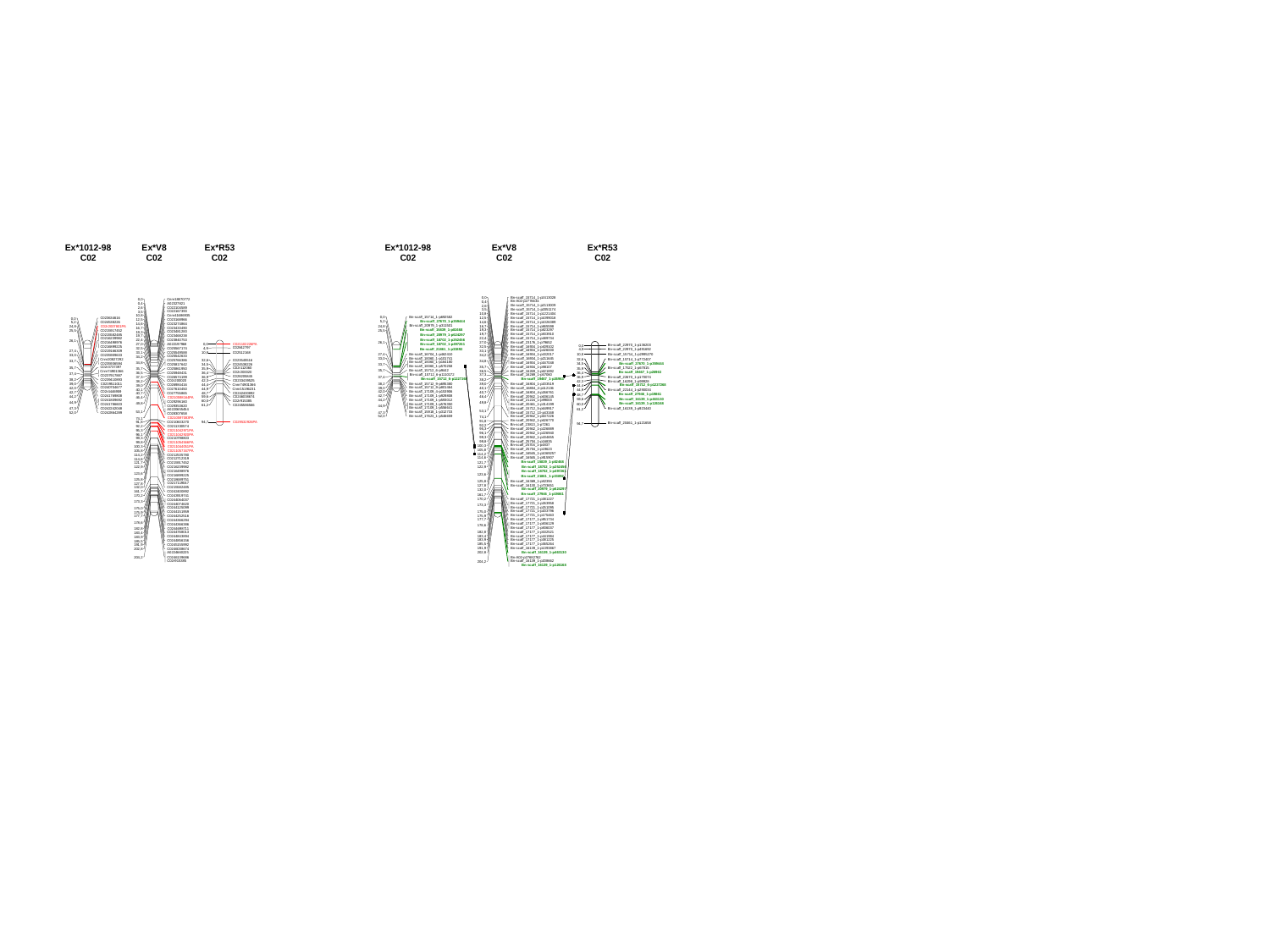

Ex*1012-98
C02
Ex*V8
C02
Ex*R53
C02
Cnnr18870772
0,0
A02327821
0,4
C022104589
2,6
C022167393
3,5
Cnnr41686935
10,8
C023168966
12,5
C023274864
14,6
C023433490
16,7
C023481283
19,3
C023468238
19,7
C023840753
22,4
A022197968
27,0
C025567174
32,5
C025549588
33,1
C025542633
34,2
C025766386
34,8
C025817642
C025861950
35,7
C025948431
36,5
C026574199
37,3
C02r200020
38,2
C026984416
39,0
C027610450
40,1
C027755865
40,7
C0210598164PA
46,4
C029296160
49,8
C029353620
A0220655454
53,1
C028307658
C0210597083PA
74,1
C0210603270
91,8
C0211330574
92,2
C0211042971PA
95,3
C0211042920PA
96,1
C0210798803
99,3
C0211054566PA
99,8
C0211044051PA
100,3
C0211057347PA
105,8
C0212535780
114,2
C0212712319
114,6
C0215917452
121,7
C0216239982
122,9
C0216498976
123,6
C0216899225
C0218689751
125,8
C0217119047
127,8
C0215582485
132,0
C0242400892
161,7
C0243919741
170,2
C0244064037
173,3
C0244074620
C0244125099
175,0
C0244151959
C023634616
0,0
C024538226
5,2
C02r2007801PA
24,8
C0215917452
25,5
C0215582485
C0216239982
26,1
C0216498976
C021102226PA
0,0
C0216899225
C02842797
4,9
C0219166309
27,4
C02512168
10,8
C0235909633
33,0
Cnnr20827292
C023545516
32,6
33,7
C0235936594
C024538226
34,5
C02r3737397
35,7
C02r112060
35,9
Cnnr74901366
C02r200020
36,4
37,4
C0237917887
C028205845
36,9
C0239410893
38,2
C0233439525
42,3
C0239511011
39,0
Cnnr74901366
44,4
C0240704677
42,0
Cnnr15196231
44,9
C02r4446959
42,7
C0242400892
48,7
C0241769808
44,2
C0246038674
59,6
C0241809692
C02r915385
60,0
44,9
C0241786603
C0245586566
61,2
C0242242048
47,3
C0242864289
52,0
C029532926PA
94,7
175,9
C0244252516
177,7
C0244366294
178,6
C0244366386
C0244698711
182,8
C0244768013
183,4
C0244843894
183,9
C0244856156
185,5
C0245155992
191,9
C0246038674
202,8
A0224660225
C0246139686
204,2
C02r915385
Ex*1012-98
C02
Ex*V8
C02
Ex*R53
C02
Bn-scaff_15714_1-p2413028
0,0
Bn-A02-p2776634
0,4
Bn-scaff_15714_1-p2113009
2,6
Bn-scaff_15714_1-p2051174
3,5
Bn-scaff_15714_1-p1221404
10,8
Bn-scaff_15714_1-p1099018
12,5
Bn-scaff_15714_1-p1026389
14,6
Bn-scaff_15714_1-p865598
16,7
Bn-scaff_15714_1-p823297
19,3
Bn-scaff_15714_1-p833910
19,7
Bn-scaff_15714_1-p489734
22,4
Bn-scaff_23176_1-p79652
27,0
Bn-scaff_16934_1-p409102
32,5
Bn-scaff_16934_1-p426000
33,1
Bn-scaff_16934_1-p432017
34,2
Bn-scaff_16934_1-p211645
34,8
Bn-scaff_16934_1-p167048
Bn-scaff_16934_1-p98107
35,7
Bn-scaff_16269_1-p621892
36,5
Bn-scaff_16269_1-p57060
37,3
Bn-scaff_19467_1-p26963
38,2
Bn-scaff_16804_1-p203519
39,0
Bn-scaff_16804_4-p112136
40,1
Bn-scaff_16804_4-p266761
40,7
Bn-scaff_20942_1-p636145
46,4
Bn-scaff_21224_1-p99003
49,8
Bn-scaff_20461_1-p314199
Bn-scaff_15712_5-p649917
53,1
Bn-scaff_15712_13-p43168
Bn-scaff_20942_1-p637226
74,1
Bn-scaff_20942_1-p626770
91,8
Bn-scaff_23813_1-p7261
92,2
Bn-scaff_20942_1-p226889
95,3
Bn-scaff_20942_1-p226940
96,1
Bn-scaff_20942_1-p434655
99,3
Bn-scaff_25704_1-p16835
99,8
Bn-scaff_25704_1-p4837
100,3
Bn-scaff_25704_1-p19623
105,8
Bn-scaff_16565_1-p1069257
114,2
Bn-scaff_16565_1-p915907
114,6
Bn-scaff_15839_1-p82468
121,7
Bn-scaff_18702_1-p252456
122,9
Bn-scaff_18702_1-p497261
123,6
Bn-scaff_21861_1-p33892
Bn-scaff_16369_1-p82394
125,8
Bn-scaff_16130_1-p733651
127,8
Bn-scaff_20979_1-p624297
132,0
Bn-scaff_27946_1-p19861
161,7
Bn-scaff_17721_1-p381227
170,2
Bn-scaff_17721_1-p263958
173,3
Bn-scaff_17721_1-p251095
Bn-scaff_17721_1-p203796
175,0
Bn-scaff_17721_1-p175463
Bn-scaff_15714_1-p692582
0,0
Bn-scaff_27670_1-p359444
5,2
Bn-scaff_20979_1-p311501
24,8
Bn-scaff_15839_1-p82468
25,5
Bn-scaff_20979_1-p624297
Bn-scaff_18702_1-p252456
26,1
Bn-scaff_18702_1-p497261
Bn-scaff_22970_1-p136203
0,0
Bn-scaff_22970_1-p491692
Bn-scaff_21861_1-p33892
4,9
Bn-scaff_15714_1-p2995270
Bn-scaff_16704_1-p462410
10,8
27,4
Bn-scaff_18360_1-p101741
33,0
Bn-scaff_15714_1-p772407
32,6
Bn-scaff_18360_1-p164180
Bn-scaff_27670_1-p359444
34,5
33,7
Bn-scaff_18360_1-p570258
Bn-scaff_17522_1-p67815
35,9
Bn-scaff_15712_6-p9442
35,7
Bn-scaff_19467_1-p26963
36,4
Bn-scaff_15712_6-p1110172
Bn-scaff_22670_1-p170074
37,4
36,9
Bn-scaff_15712_6-p1227268
Bn-scaff_16298_1-p99928
42,3
Bn-scaff_15712_9-p695380
38,2
Bn-scaff_15712_6-p1227268
44,4
Bn-scaff_15712_9-p801464
39,0
Bn-scaff_22144_1-p290034
44,9
Bn-scaff_17109_4-p153906
42,0
Bn-scaff_27946_1-p19861
48,7
Bn-scaff_17109_1-p929808
42,7
Bn-scaff_16139_1-p463130
59,6
Bn-scaff_17109_1-p593012
44,2
Bn-scaff_16139_1-p126168
Bn-scaff_17109_1-p576350
60,0
44,9
Bn-scaff_17109_1-p556641
Bn-scaff_16139_1-p923440
61,2
Bn-scaff_15918_1-p312733
47,3
Bn-scaff_17623_1-p546659
52,0
Bn-scaff_20461_1-p121658
94,7
175,9
Bn-scaff_17177_1-p951734
177,7
Bn-scaff_17177_1-p836129
178,6
Bn-scaff_17177_1-p836037
Bn-scaff_17177_1-p532521
182,8
Bn-scaff_17177_1-p441984
183,4
Bn-scaff_17177_1-p381225
183,9
Bn-scaff_17177_1-p365264
185,5
Bn-scaff_16139_1-p1393867
191,9
Bn-scaff_16139_1-p463130
202,8
Bn-A02-p27682762
Bn-scaff_16139_1-p208662
204,2
Bn-scaff_16139_1-p126168

## Slide 13
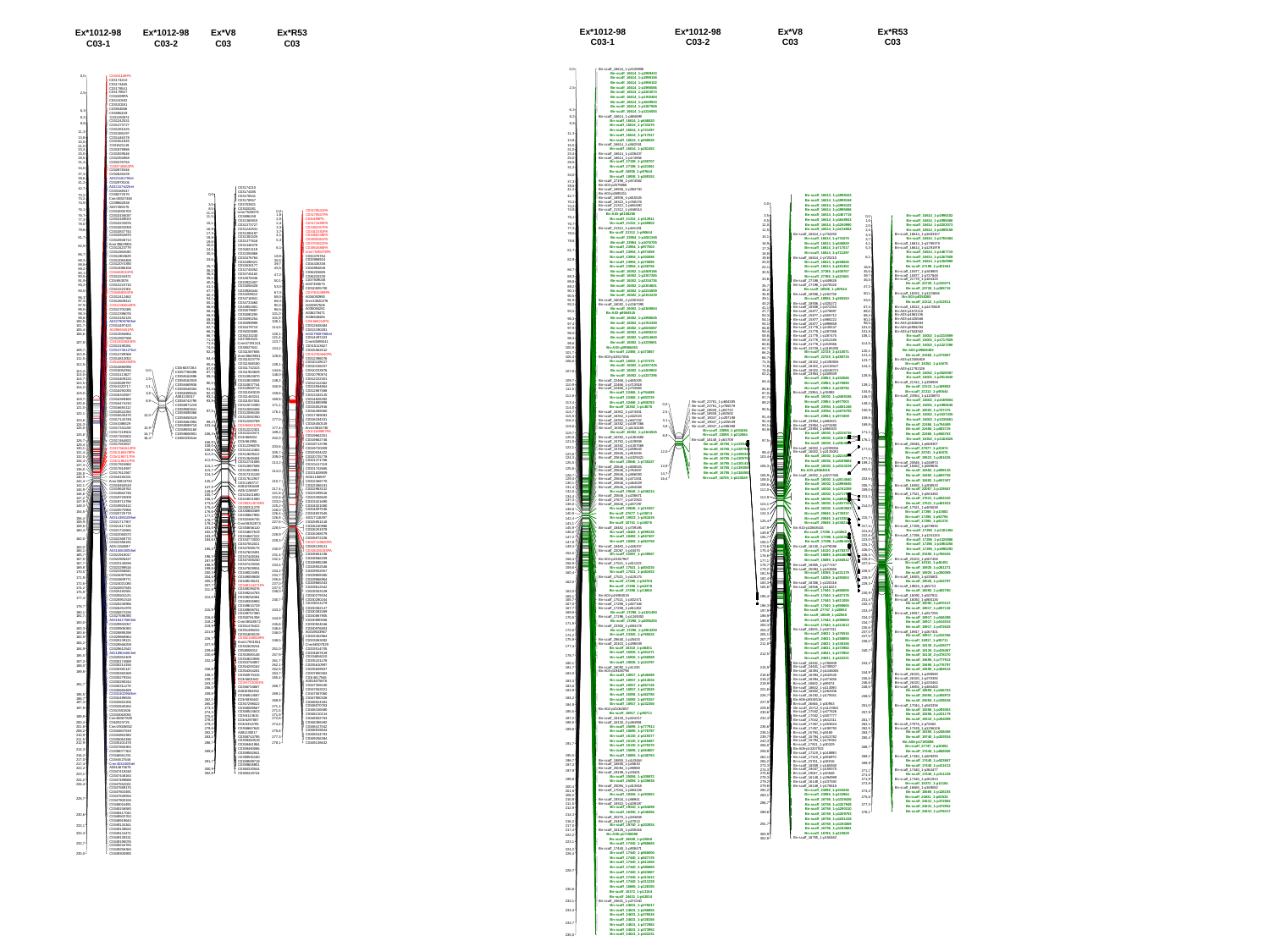

Ex*1012-98
C03-1
Ex*1012-98
C03-2
Ex*V8
C03
Ex*R53
C03
Bn-scaff_16614_1-p2138906
0,0
Bn-scaff_16614_1-p1999433
Bn-scaff_16614_1-p1999158
Bn-scaff_16614_1-p1995102
Bn-scaff_16614_1-p1995086
2,5
Bn-scaff_16614_1-p2203073
Bn-scaff_16614_1-p1765484
Bn-scaff_16614_1-p1649933
Bn-scaff_16614_1-p1267508
6,3
Bn-scaff_16614_1-p1216083
Bn-scaff_16614_1-p884699
8,2
Bn-scaff_16614_1-p846820
8,8
Bn-scaff_16614_1-p731075
Bn-scaff_16614_1-p721297
11,3
Bn-scaff_16614_1-p717017
Bn-scaff_16614_1-p658026
13,8
Bn-scaff_16614_1-p562041
15,5
Bn-scaff_16614_1-p281452
21,0
Bn-scaff_16614_1-p226437
23,4
Bn-scaff_16614_1-p174856
25,0
Bn-scaff_27198_1-p300707
28,5
Bn-scaff_27198_1-p421561
31,2
Bn-scaff_18936_1-p97644
34,0
Bn-scaff_18936_1-p269153
Bn-scaff_27198_1-p574582
37,3
Bn-A03-p2579866
39,6
Bn-scaff_18936_1-p384730
41,2
Bn-A03-p2695311
42,7
Bn-scaff_18936_1-p615525
Bn-scaff_18322_1-p768276
70,2
Bn-scaff_21312_1-p681690
74,2
Bn-scaff_21312_1-p546514
74,9
Bn-A03-p8158265
76,1
Bn-scaff_21312_1-p313911
Bn-scaff_21312_1-p169902
76,7
Bn-scaff_21312_1-p161201
77,3
Bn-scaff_21312_1-p90644
78,0
Bn-scaff_23954_1-p1081348
79,8
Bn-scaff_23954_1-p1076705
Bn-scaff_23954_1-p977003
81,7
Bn-scaff_23954_1-p971859
Bn-scaff_23954_1-p302686
82,9
Bn-scaff_23954_1-p270890
Bn-scaff_23954_1-p228756
86,7
Bn-scaff_16002_1-p2469186
Bn-scaff_16002_1-p2307265
88,3
Bn-scaff_16002_1-p2316736
88,8
Bn-scaff_16002_1-p2304601
89,2
Bn-scaff_16002_1-p2215859
90,1
Bn-scaff_16002_1-p2153239
90,5
Bn-scaff_16002_1-p2201513
91,9
Bn-scaff_16002_1-p2167390
93,2
Bn-scaff_16002_1-p2169804
93,5
Bn-A03-p9564515
Bn-scaff_16002_1-p1998645
96,3
Bn-scaff_16002_1-p1762359
97,4
Bn-scaff_16002_1-p2026087
97,9
Bn-scaff_16002_1-p1688212
98,0
Bn-scaff_16002_1-p2014840
98,3
Bn-scaff_16002_1-p1235661
98,6
Bn-scaff_16614_1-p1999433
Bn-scaff_16614_1-p1999158
0,0
Bn-scaff_16614_1-p1995102
Bn-scaff_16614_1-p1995086
Bn-scaff_16614_1-p1467715
3,5
Bn-scaff_16614_1-p1649933
8,5
Bn-scaff_16614_1-p1250990
11,0
Bn-scaff_16614_1-p1216083
11,5
Bn-scaff_16614_1-p734250
15,1
Bn-scaff_16614_1-p731075
Bn-scaff_16614_1-p846820
16,9
Bn-scaff_16614_1-p717017
17,3
Bn-scaff_16614_1-p721297
18,8
Bn-scaff_16614_1-p725210
19,6
Bn-scaff_16614_1-p658026
20,0
Bn-scaff_16614_1-p281452
24,1
Bn-scaff_27198_1-p300707
30,5
Bn-scaff_27198_1-p421561
33,6
Bn-scaff_27198_1-p449626
Bn-scaff_27198_1-p578320
35,7
Bn-scaff_18936_1-p97644
36,2
Bn-scaff_18936_1-p102755
36,8
Bn-scaff_18936_1-p269153
39,1
Bn-scaff_18936_1-p325272
40,2
Bn-scaff_18936_1-p472353
41,1
Bn-scaff_15877_1-p278897
47,7
Bn-scaff_15877_1-p555712
50,3
Bn-scaff_15877_1-p985222
54,1
Bn-scaff_15877_1-p999859
55,1
Bn-scaff_21778_1-p149147
56,8
Bn-scaff_21778_1-p287068
58,4
Bn-scaff_21778_1-p287475
58,8
Bn-scaff_21778_1-p312180
59,3
Bn-scaff_21778_1-p319366
60,6
Bn-scaff_22728_1-p1185330
62,7
Bn-scaff_22728_1-p310071
66,2
Bn-scaff_22728_1-p285715
66,7
Bn-scaff_18322_1-p1390926
71,3
Bn-scaff_18322_1-p1315547
71,8
Bn-scaff_18322_1-p1046721
74,0
Bn-scaff_23954_1-p209939
82,2
Bn-scaff_23954_1-p302686
84,4
Bn-scaff_23954_1-p270890
Bn-scaff_23954_1-p228756
85,6
Bn-scaff_23954_1-p76992
87,0
Bn-scaff_16002_1-p2469186
87,7
Bn-scaff_23954_1-p977003
89,2
Bn-scaff_23954_1-p1081348
90,5
Bn-scaff_23954_1-p1076705
Bn-scaff_23954_1-p971859
91,4
Bn-scaff_23954_1-p882621
92,4
Bn-scaff_23954_1-p373490
93,1
Bn-scaff_23954_1-p865303
93,9
Bn-scaff_16002_1-p2316736
Bn-scaff_16002_1-p2307265
Bn-scaff_16002_1-p2304601
Bn-scaff_16614_1-p1995102
0,0
Bn-scaff_16614_1-p1995086
1,6
Bn-scaff_16614_1-p2203073
2,0
Bn-scaff_16614_1-p1999158
2,4
Bn-scaff_16614_1-p1683107
3,3
Bn-scaff_16614_1-p1765484
3,7
Bn-scaff_16614_1-p1785370
4,1
Bn-scaff_16614_1-p1291979
5,3
Bn-scaff_16614_1-p1467715
Bn-scaff_16614_1-p1267508
6,1
Bn-scaff_16614_1-p1250990
Bn-scaff_27198_1-p421561
18,8
Bn-scaff_15877_1-p349800
35,5
Bn-scaff_15877_1-p737848
39,7
Bn-scaff_21778_1-p184325
45,5
Bn-scaff_22728_1-p310071
47,2
Bn-scaff_22728_1-p285715
Bn-scaff_18322_1-p1123656
50,1
Bn-A03-p8158265
53,0
Bn-scaff_21312_1-p313911
Bn-scaff_18322_1-p1470659
67,4
Bn-A03-p4572110
88,0
Bn-A03-p4861136
88,4
Bn-A03-p4425566
90,2
Bn-A03-p5626694
96,5
Bn-A03-p6994294
101,0
Bn-A03-p7543362
101,8
Bn-scaff_16002_1-p2215859
108,1
Bn-scaff_16002_1-p1717929
114,5
Bn-scaff_16002_1-p1227296
Bn-A03-p29866453
120,1
Bn-scaff_22466_1-p373867
121,4
Bn-A03-p13306819
123,7
Bn-scaff_16352_1-p14678
124,2
Bn-A03-p11792328
Bn-scaff_16002_1-p2026087
128,8
Bn-scaff_16002_1-p2014840
Bn-scaff_21312_1-p198928
139,1
Bn-scaff_21312_1-p169902
Bn-scaff_21312_1-p90644
144,6
Bn-scaff_23954_1-p1108870
146,0
Bn-scaff_16002_1-p2169804
149,2
Bn-scaff_16002_1-p1998645
Bn-scaff_16002_1-p727475
150,9
Bn-scaff_16002_1-p1537435
159,6
Bn-scaff_16002_1-p1235661
Bn-scaff_22466_1-p754489
169,8
Bn-scaff_22466_1-p803725
171,1
Bn-scaff_22466_1-p945763
Bn-scaff_16352_1-p1164525
176,1
Bn-scaff_20646_1-p668307
Bn-scaff_27677_1-p22874
177,0
Bn-scaff_20741_1-p16076
Bn-scaff_19523_1-p261625
177,4
Bn-scaff_20646_1-p246974
189,2
Bn-scaff_18482_1-p699636
202,0
Bn-scaff_18482_1-p699135
Bn-scaff_18482_1-p663758
Bn-A03-p29866453
100,8
Bn-scaff_22466_1-p373867
101,7
Bn-A03-p10517056
105,4
Bn-scaff_16002_1-p727475
106,6
Bn-scaff_16002_1-p1537435
Bn-scaff_16002_1-p1400602
107,8
Bn-scaff_16002_1-p1227296
Bn-scaff_22466_1-p655205
109,7
Bn-scaff_22466_1-p713918
110,9
Bn-scaff_22466_1-p733665
111,5
Bn-scaff_22466_1-p754489
112,8
Bn-scaff_22466_1-p803725
Bn-scaff_22466_1-p945763
113,4
Bn-scaff_16352_1-p14678
114,0
Bn-scaff_16352_1-p274501
114,7
Bn-scaff_16352_1-p442023
115,5
Bn-scaff_16352_1-p647232
116,2
Bn-scaff_16352_1-p1097366
119,0
Bn-scaff_16352_1-p1154186
Bn-scaff_16352_1-p1164525
119,7
Bn-scaff_16352_1-p1261680
120,0
Bn-scaff_15782_1-p129938
121,0
Bn-scaff_16352_1-p1307068
122,1
Bn-scaff_15782_1-p259643
Bn-scaff_20646_1-p915635
123,6
Bn-scaff_20646_1-p1029421
124,3
Bn-scaff_20646_1-p735337
125,0
Bn-scaff_20646_1-p838525
125,8
Bn-scaff_20646_1-p764847
Bn-scaff_20646_1-p586593
126,7
Bn-scaff_20646_1-p471831
129,4
Bn-scaff_20646_1-p464029
130,1
Bn-scaff_20646_1-p264068
131,4
Bn-scaff_20646_1-p246214
132,0
Bn-scaff_20646_1-p238671
134,4
Bn-scaff_27677_1-p272923
137,3
Bn-scaff_20646_1-p207297
138,0
Bn-scaff_20646_1-p210307
138,8
Bn-scaff_27677_1-p22874
140,9
Bn-scaff_19523_1-p261625
142,4
Bn-scaff_20741_1-p16076
143,1
Bn-scaff_18482_1-p739195
145,9
Bn-scaff_18482_1-p699135
146,6
Bn-scaff_18482_1-p657407
147,2
Bn-scaff_18482_1-p663758
147,9
Bn-scaff_18482_1-p445207
149,3
Bn-scaff_22067_1-p10470
154,5
Bn-scaff_22067_1-p139587
Bn-A03-p15347967
156,4
Bn-scaff_17521_1-p511223
158,9
Bn-scaff_17521_1-p804330
159,6
Bn-scaff_17521_1-p481933
160,4
Bn-scaff_17521_1-p125175
Bn-scaff_17298_1-p52794
162,0
Bn-scaff_17298_1-p62378
Bn-scaff_23761_1-p604385
0,0
Bn-scaff_23761_1-p765578
Bn-scaff_18559_1-p93723
2,5
Bn-scaff_18559_1-p93500
Bn-scaff_19047_1-p297198
3,1
Bn-scaff_19047_1-p225539
Bn-scaff_19047_1-p196388
3,8
Bn-scaff_23098_1-p304246
97,5
Bn-scaff_16002_1-p2299056
Bn-scaff_16002_1-p2135081
99,4
Bn-scaff_16002_1-p2215859
103,4
Bn-scaff_16002_1-p2169804
Bn-scaff_16002_1-p2153239
106,3
Bn-A03-p9564515
Bn-scaff_16002_1-p2227436
106,9
Bn-scaff_16002_1-p2014840
108,0
Bn-scaff_16002_1-p1998645
108,6
Bn-scaff_16002_1-p1762359
112,0
Bn-scaff_16002_1-p1717929
112,9
Bn-scaff_16002_1-p1688212
Bn-scaff_16002_1-p1537435
115,1
Bn-scaff_16002_1-p1400602
115,7
Bn-scaff_20646_1-p735337
124,3
Bn-scaff_20646_1-p210307
125,4
Bn-scaff_20646_1-p246214
Bn-A10-p10848415
147,9
Bn-scaff_17298_1-p13882
149,8
Bn-scaff_17298_1-p1320886
155,7
Bn-scaff_17298_1-p1964250
156,1
Bn-scaff_16130_2-p376596
173,6
Bn-scaff_16130_2-p378370
175,4
Bn-scaff_15695_1-p564807
176,6
Bn-scaff_15695_1-p384512
177,1
Bn-scaff_18855_1-p177157
178,7
Bn-scaff_25094_1-p153866
179,2
Bn-scaff_18356_1-p231179
181,5
Bn-scaff_18356_1-p293063
182,4
Bn-scaff_18356_1-p220316
183,3
Bn-scaff_18356_1-p116223
184,6
Bn-scaff_17440_1-p868006
Bn-scaff_17440_1-p827176
195,4
Bn-scaff_17440_1-p811055
Bn-scaff_17440_1-p958665
196,3
Bn-scaff_27747_1-p20852
197,9
Bn-scaff_18849_1-p22568
198,9
Bn-scaff_17440_1-p590665
199,8
Bn-scaff_17440_1-p411613
200,3
Bn-scaff_24631_1-p187311
204,4
Bn-scaff_24631_1-p276516
205,1
Bn-scaff_24631_1-p258855
207,7
Bn-scaff_24631_1-p338156
211,8
Bn-scaff_24631_1-p372982
Bn-scaff_24631_1-p373952
212,5
Bn-scaff_24631_1-p422231
Bn-scaff_24631_1-p785809
Bn-scaff_24631_1-p739507
215,9
Bn-scaff_16394_2-p1446065
Bn-scaff_16394_2-p542540
216,6
Bn-scaff_16394_2-p471655
218,2
Bn-scaff_18602_1-p89074
219,9
Bn-scaff_18602_1-p113090
221,6
Bn-scaff_18602_1-p262006
Bn-scaff_16182_1-p175551
226,7
Bn-A08-p8334516
227,9
Bn-scaff_26846_1-p30953
Bn-scaff_23098_1-p232984
8,9
Bn-scaff_16148_1-p63709
Bn-scaff_16755_1-p1339426
Bn-scaff_16755_1-p1327949
12,0
Bn-scaff_16755_1-p1290310
Bn-scaff_16755_1-p1209753
Bn-scaff_16755_1-p1201419
13,9
Bn-scaff_16755_1-p1194069
Bn-scaff_16755_1-p1184581
14,7
203,6
Bn-scaff_18482_1-p657407
Bn-scaff_18482_1-p308533
205,7
Bn-scaff_22067_1-p139587
209,0
Bn-scaff_17521_1-p843450
213,2
Bn-scaff_17521_1-p804330
Bn-scaff_17521_1-p481933
214,0
Bn-scaff_17521_1-p405038
Bn-scaff_17298_1-p13882
Bn-scaff_17298_1-p52794
215,7
Bn-scaff_17298_1-p62378
Bn-scaff_17298_1-p679830
217,4
Bn-scaff_17298_1-p1181494
221,8
Bn-scaff_17298_1-p1251153
222,6
Bn-scaff_17298_1-p1320886
223,0
Bn-scaff_17298_1-p1964250
225,2
Bn-scaff_17298_1-p2096293
226,0
Bn-scaff_23350_1-p765525
226,4
Bn-scaff_20103_1-p507458
226,8
Bn-scaff_16310_1-p46451
227,6
Bn-scaff_16925_1-p261271
228,5
Bn-scaff_16925_1-p250089
Bn-scaff_16925_1-p235601
228,9
Bn-scaff_19026_1-p324797
229,3
Bn-scaff_19026_1-p55713
Bn-scaff_16092_1-p462780
230,9
Bn-scaff_16092_1-p507811
Bn-scaff_16092_1-p865136
231,4
Bn-scaff_16092_1-p870157
232,4
Bn-scaff_18917_1-p857136
233,4
Bn-scaff_18917_1-p817255
Bn-scaff_18917_1-p548498
234,2
Bn-scaff_18917_1-p514534
234,7
Bn-scaff_18917_1-p472625
235,6
Bn-scaff_18917_1-p267401
237,0
Bn-scaff_18917_1-p232356
237,5
Bn-scaff_18917_1-p90711
238,0
Bn-scaff_16130_2-p419377
Bn-scaff_16130_2-p418467
240,7
Bn-scaff_16130_2-p378370
Bn-scaff_15695_1-p777913
243,2
Bn-scaff_15695_1-p776797
Bn-scaff_15695_1-p384512
244,9
Bn-scaff_26320_1-p298590
Bn-scaff_26320_1-p270392
245,6
Bn-scaff_26320_1-p220462
246,6
Bn-scaff_18855_1-p480400
248,0
Bn-scaff_18855_1-p246763
Bn-scaff_25094_1-p305972
248,5
Bn-scaff_25094_1-p339638
Bn-scaff_17044_1-p545336
251,0
Bn-scaff_18356_1-p293063
257,9
Bn-scaff_18356_1-p231179
Bn-scaff_19310_1-p254099
261,7
Bn-scaff_27076_1-p78402
262,1
Bn-scaff_17249_1-p1256126
262,5
Bn-scaff_16350_1-p248456
263,7
Bn-scaff_19740_1-p203934
265,4
Bn-A08-p17190098
Bn-scaff_27747_1-p20852
268,7
Bn-scaff_17440_1-p868006
Bn-scaff_16755_1-p223829
16,4
Bn-scaff_17298_1-p13882
163,0
Bn-A03-p15983019
165,1
Bn-scaff_17521_1-p302471
165,7
Bn-scaff_17298_1-p627166
167,0
Bn-scaff_17298_1-p811262
167,7
Bn-scaff_17298_1-p1181494
169,8
Bn-scaff_17298_1-p1249353
170,5
Bn-scaff_17298_1-p2096293
171,9
Bn-scaff_23350_1-p594178
Bn-scaff_17298_1-p1964250
172,6
Bn-scaff_23350_1-p765525
174,2
Bn-scaff_28640_1-p25433
175,9
Bn-scaff_20103_1-p298039
177,4
Bn-scaff_16310_1-p46451
Bn-scaff_16925_1-p261271
178,7
Bn-scaff_16925_1-p250089
Bn-scaff_19026_1-p324797
180,1
Bn-scaff_16092_1-p11235
181,7
Bn-A03-p19438796
183,0
Bn-scaff_18917_1-p548498
Bn-scaff_18917_1-p514534
183,3
Bn-scaff_18917_1-p857136
183,6
Bn-scaff_18917_1-p472625
183,9
Bn-scaff_16092_1-p462780
184,2
Bn-scaff_16092_1-p870157
Bn-scaff_18917_1-p232356
184,9
Bn-A02-p21062857
185,5
Bn-scaff_18917_1-p90711
Bn-scaff_16130_2-p524217
187,2
Bn-scaff_16130_2-p484981
188,0
Bn-scaff_15695_1-p777913
189,8
Bn-scaff_15695_1-p776797
Bn-scaff_16130_2-p419377
Bn-scaff_16130_2-p418467
191,7
Bn-scaff_16130_2-p378370
Bn-scaff_15695_1-p564807
Bn-scaff_18855_1-p246763
195,5
Bn-scaff_18855_1-p143464
196,7
Bn-scaff_18855_1-p25630
197,3
Bn-scaff_25094_1-p95988
197,9
Bn-scaff_19109_1-p33401
Bn-scaff_25094_1-p305972
199,8
Bn-scaff_25094_1-p339638
Bn-scaff_25094_1-p413818
200,4
Bn-scaff_17044_1-p594126
201,8
Bn-scaff_18356_1-p293063
208,2
Bn-scaff_19310_1-p86941
210,9
Bn-scaff_19310_1-p209197
211,5
Bn-scaff_19310_1-p254099
212,9
Bn-scaff_16350_1-p248456
214,3
Bn-scaff_20270_1-p194658
Bn-scaff_23407_1-p27013
216,2
Bn-scaff_19740_1-p203934
217,0
Bn-scaff_16135_1-p230416
217,4
Bn-A08-p17190098
222,2
Bn-scaff_18849_1-p22568
223,1
Bn-scaff_17440_1-p958665
Bn-scaff_15712_5-p1123924
229,6
Bn-scaff_17042_1-p477626
230,6
Bn-scaff_17042_1-p507777
232,4
Bn-scaff_17042_1-p642311
Bn-scaff_17457_1-p253824
236,6
Bn-scaff_17457_1-p190793
Bn-scaff_15794_3-p9180
238,1
Bn-scaff_15794_1-p313782
239,7
Bn-scaff_15794_1-p178361
243,3
Bn-scaff_17821_1-p30329
256,0
Bn-A08-p13207923
259,8
Bn-scaff_17119_1-p148890
Bn-scaff_17440_1-p829293
269,1
Bn-scaff_17119_1-p386970
262,1
Bn-scaff_17440_1-p523867
Bn-scaff_23761_1-p28016
265,2
269,9
Bn-scaff_18559_1-p165940
Bn-scaff_17440_1-p411613
273,3
Bn-scaff_19047_1-p159375
274,3
Bn-scaff_17440_1-p364477
271,1
Bn-scaff_19047_1-p10848
275,6
Bn-scaff_17440_1-p311228
271,5
Bn-scaff_16148_1-p294989
278,3
Bn-scaff_17440_1-p261914
271,9
Bn-scaff_16148_1-p237592
279,2
Bn-scaff_16372_1-p13184
272,8
Bn-scaff_16148_1-p178616
279,6
Bn-scaff_16665_1-p169062
Bn-scaff_23098_1-p304246
282,2
274,4
Bn-scaff_16665_1-p128193
Bn-scaff_23098_1-p232984
283,1
Bn-scaff_24631_1-p63534
275,6
Bn-scaff_16755_1-p1339426
Bn-scaff_24631_1-p372982
286,7
Bn-scaff_16755_1-p1327949
277,3
Bn-scaff_24631_1-p373952
Bn-scaff_16755_1-p1290310
Bn-scaff_24631_1-p276017
289,6
278,1
Bn-scaff_16755_1-p1209753
Bn-scaff_16755_1-p1201419
Bn-scaff_16755_1-p1194069
291,7
Bn-scaff_16755_1-p1184581
Bn-scaff_16755_1-p223829
300,9
Bn-scaff_16755_1-p150462
302,8
Bn-scaff_17440_1-p938471
224,2
Bn-scaff_17440_1-p868006
226,4
Bn-scaff_17440_1-p827176
Bn-scaff_17440_1-p811055
Bn-scaff_17440_1-p590665
228,7
Bn-scaff_17440_1-p523867
Bn-scaff_17440_1-p411613
Bn-scaff_17440_1-p311228
Bn-scaff_16665_1-p128193
230,6
Bn-scaff_16372_1-p13184
Bn-scaff_24631_1-p63534
Bn-scaff_24631_1-p271540
233,1
Bn-scaff_24631_1-p276017
Bn-scaff_24631_1-p258855
233,3
Bn-scaff_24631_1-p276516
Bn-scaff_24631_1-p338156
233,7
Bn-scaff_24631_1-p372982
Bn-scaff_24631_1-p373952
Bn-scaff_24631_1-p422231
235,0
Ex*1012-98
C03-1
Ex*1012-98
C03-2
Ex*V8
C03
Ex*R53
C03
C03r81108PA
0,0
C03174210
C03174485
C03178541
C03178557
2,5
C032499PA
C03410192
C03520281
C03854586
6,3
C03896159
C031193874
8,2
C031242531
8,8
C031373727
C031381525
11,3
C031385197
C031446378
13,8
C031551845
15,5
C031821119
21,0
C031878986
23,4
C031929546
25,0
C032355968
28,5
C032476764
31,2
C032740052PA
34,0
C032878346
C032626439
37,3
A032118170het
39,6
C032978106
41,2
A032247622het
42,7
C033188347
C038272570
70,2
Cnnr36537484
74,2
C039862838
74,9
A037455575
76,1
C0310005700
C0310156037
76,7
C0310169023
77,3
C0310233876
78,0
C0310833059
79,8
C0310837744
C0310943870
81,7
C0310948713
Annr39629931
82,9
C0311523779
C0311568180
86,7
C0311903829
C0312083458
88,3
C0312074390
88,8
C0312086158
89,2
C03r869210PA
90,1
C0312243471
90,5
C03r883578
91,9
C0312224715
93,2
C0312222301
93,5
C03r868024PA
C0312412462
96,3
C0312609412
97,4
C0312384840PA
97,9
C0312701085
98,0
C0312396076
98,3
C0313182125
98,6
C03174210
C03174485
0,0
C03178541
C03178557
C03703921
3,5
C03520281
8,5
Unnr7585078
11,0
C03896159
11,5
C031365915
15,1
C031373727
C031242531
16,9
C031385197
17,3
C031381525
18,8
C031377616
19,6
C031446378
20,0
C031821119
24,1
C032355968
30,5
C032476764
33,6
C032498421
C032630177
35,7
C032740052
36,2
C032745162
36,8
C032878346
39,1
C032922407
40,2
C033058428
41,1
C033926444
47,7
C034209612
50,3
C034716941
54,1
C034731660
55,1
C034951901
56,8
C035079887
58,4
C035080293
58,8
C035093254
59,3
C035099999
60,6
C035479710
62,7
C036203685
66,2
C036233230
66,7
C037681523
71,3
Cnnr57291321
71,8
C038027841
74,0
C0311587656
82,2
Annr39629931
84,4
C0311523779
C0311568180
85,6
C0311732323
87,0
C0311903829
87,7
C0310943870
89,2
C0310833059
90,5
C0310837744
C0310948713
91,4
C0311040019
92,4
C0311463241
93,1
C0311057004
93,9
C0312074390
C0312083458
C0312086158
C03178541PA
0,0
C03178557PA
1,6
C032499PA
2,0
C03174485PA
2,4
C03492357PA
3,3
C03410192PA
3,7
C03390238PA
4,1
C03830061PA
5,3
C03703921PA
C03854586PA
6,1
Unnr7585078PA
C032476764
18,8
C033988924
35,5
C034435338
39,7
C034984848
45,5
C036203685
47,2
C036233230
C037939048
50,1
A037455575
53,0
C0310005700
C037615389PA
67,4
A034092990
88,0
Annr33520276
88,4
A033957506
90,2
A035065261
96,5
A036278471
101,0
A036848465
101,8
C03r869210PA
108,1
C0312648484
114,5
C0313190281
A0327908788het
120,1
C0314497423
121,4
Cnnr63895541
123,7
C0315113627
124,2
C0315562512
C0312384840PA
128,8
C0312396076
C0310128017
139,1
C0310156037
C0310233876
144,6
C0310790874
146,0
C0312222301
149,2
C0312412462
C0313594864
150,9
C0312887588
159,6
C0313182125
C0314826392
169,8
C0314885998
171,1
C0315052916
C0316369460
176,1
C0317389083
C0318194331
177,0
C0318450519
Annr33818730
177,4
C03r1189957PA
189,2
C0319662251
202,0
C0319662746
C0319713798
A0327908788het
100,8
C0314497423
101,7
A039693451PA
105,4
C0313594864
106,6
C0312887588
C0313023881PA
107,8
C0313190281
C0314736127het
109,7
C0314789368
110,9
C0314813052
111,5
C0314826392PA
112,8
C0314885998
C0315052916
113,4
C0315113627
114,0
C0315409133
114,7
C0315589797
115,5
C03r1023717
116,2
C0316291093
119,0
C0316343927
C0316369460
119,7
C0316474210
120,0
C0316684112
121,0
C0316522282
122,1
C0316535470
C0317143749
123,6
C03r1086529
124,3
C0317331159
125,0
C0317219504
125,8
C0317302602
C0317464822
126,7
C0317553817
129,4
C0317561613PA
130,1
C03r1165578PA
131,4
C03r1190717PA
132,0
C03r1196157PA
134,4
C0317924860
137,3
C0317615937
138,0
C0317612947
138,8
C0318194331
140,9
Annr33818730
142,4
C0318450519
143,1
C0319626762
145,9
C0319662746
146,6
C0319720009
147,2
C0319713798
147,9
C0319920412
149,3
C0320575958
154,5
C0320725778
A0314385109het
156,4
C0321717907
158,9
C0321417119
159,6
C0321742665
160,4
C0322065573
C0322368770
162,0
C0322386191
C03r6037263
0,0
C0357766096
C0358162696
2,5
C0358162919
C0358469936
3,1
C0358348304
C0358499044
3,8
A081130017
C0358743795
8,9
C0358971219
97,5
C0312092350
C0312260769
99,4
C03r869210PA
103,4
C0312222301
C0312243471
106,3
C03r868024
C03r861955
106,9
C0312396076
108,0
C0312412462
108,6
C0312609412
112,0
C0312648484
112,9
C0312701085
C0312887588
115,1
C0313023881
115,7
C0317331159
124,3
C0317612947
125,4
C03r1190717
A0812591669
147,9
A03r1156597
149,8
C0323421690
155,7
C0324021080
156,1
C0330313076PA
173,6
C0330311279
175,4
C0330660489
176,6
C0330867905
177,1
C0332466745
178,7
Cnnr59252873
179,2
C0334656110
181,5
C0334607619
182,4
C0334667222
183,3
C0334773020
184,6
C0347552021
C0347589175
195,4
C0347603491
C0347448164
196,3
C0347358240
197,9
C0347419248
198,9
C0347828934
199,8
C0348024491
200,3
C0349059638
204,4
C0349139131
205,1
C0349124471PA
207,7
C0349195076
211,8
C0349244783
C0349256384
212,5
C0349305993
C0349615729
C0349658751
215,9
C0349757380
C0350761358
216,6
Cnnr38528572
218,2
C0351476422
219,9
C0351498232
221,6
C0351659128
C0352108549PA
226,7
Annr17931551
227,9
C0352628244
C0359582841
C0359490066
12,0
C0359450519
C0359461984
C0359599719
13,9
C0359591160
C0359606901
14,7
C0360330544
16,4
203,6
C0319720009
C0320034422
205,7
C0320725778
209,0
C0321371766
213,2
C0321417119
C0321742665
214,0
C0321828809
A03r1156597
C0322368770
215,7
C0322386191
C0322963312
217,4
C0323299516
221,8
C0323358620
222,6
C0323421690
223,0
C0324021080
225,2
C0324097536
226,0
C0324937645
226,4
A0317118397
226,8
C0325951518
227,6
C0326240986
228,5
C0326251979
C0326268079
228,9
C0326872226
229,3
C0327129820PA
C0328139121
230,9
C0328185193PA
C0328561236
231,4
C0328566259
232,4
C0328895298
233,4
C0328932548
C0329932057
234,2
C0329908360
234,7
C0329866964
235,6
C0329665343
237,0
C0329612542
237,5
C0329353109
238,0
C0330279334
C0330280244
240,7
C0330311279
C0330382147
243,2
C0330383269
C0330867905
244,9
C0330890556
C0330924046
245,6
C0330976853
246,6
A0319633967
248,0
C0332402964
C0333063092
248,5
Cnnr66507829
C0333514705
251,0
C0334607619
257,9
C0334656110
C0335101478
261,7
C0335610997
262,1
C0335849937
262,5
C0337850363
263,7
C03r4617546
265,4
A0814675678
C0347358240
268,7
C0347552021
A03r1156597
163,0
A0315063005het
165,1
C0321918017
165,7
C0322906437
167,0
C0323140836
167,7
C0323299516
169,8
C0323356841
170,5
C0324097536
171,9
C0324809771
C0324021080
172,6
C0324937645
174,2
C0325192581
175,9
C0325501123
177,4
C0325951518
C0326240986
178,7
C0326251979
C0326872226
180,1
C0327596390
181,7
A0318417565het
183,0
C0329932057
C0329908360
183,3
C0328895298
183,6
C0329866964
183,9
C0328139121
184,2
C0328566259
C0329612542
184,9
A0218904482het
185,5
C0329353109
C0330174889
187,2
C0330214194
188,0
C0330382147
189,8
C0330383269
C0330279334
C0330280244
191,7
C0330311279
C0330660489
C0332402964het
195,5
C0332498535
196,7
C0332652206
197,3
C0332840264
197,9
C03r2551506
C0333063092
199,8
Cnnr66507829
C062023725
200,4
Cnnr29046552
201,8
C0334607619
208,2
C0334963380
210,9
C0335064196
211,5
C0335101478
212,9
C0337850363
214,3
C0338077302
C0346561231
216,2
C03r4617546
217,0
Cnnr4032405het
217,4
A0814675678
222,2
C0347419248
223,1
C0347448164
C058008312
229,6
C0353580148
230,6
C0353624936
232,4
C0353750857
C0354293162
236,6
C0354354281
C0355570415
238,1
C03r5601542
239,7
C03r5722091PA
243,3
C0356704667
256,0
A0810953352
259,8
C0347587060
269,1
C0356814587
C0347900326
C03r5836442
262,1
269,9
C0348024491
C0357298022
265,2
C0348070763
271,1
C0358088567
273,3
C0348156580
271,5
C0358524623
274,3
C0348210214
271,9
C03r6113615
275,6
C0348502763
272,8
C03r6297887
278,3
C0348388492
C03r6334705
279,2
274,4
C0348417042
C0358667842
279,6
C0348919644
275,6
A081130017
282,2
C0349244783
C0358743795
283,1
277,3
C0349256384
C0359450519
286,7
C0349138632
278,1
C0359461984
C0359490066
289,6
C0359582841
C0359591160
C0359599719
291,7
C0359606901
C0360330544
300,9
C0360418744
302,8
C0347489688
224,2
C0347552021
226,4
C0347589175
C0347603491
C0347828934
228,7
C0347900326
C0348024491
C0348156580
C0348417042
230,6
C0348502763
C0348919644
C0349134161
233,1
C0349138632
C0349124471
233,3
C0349139131
C0349195076
233,7
C0349244783
C0349256384
C0349305993
235,0

## Slide 14
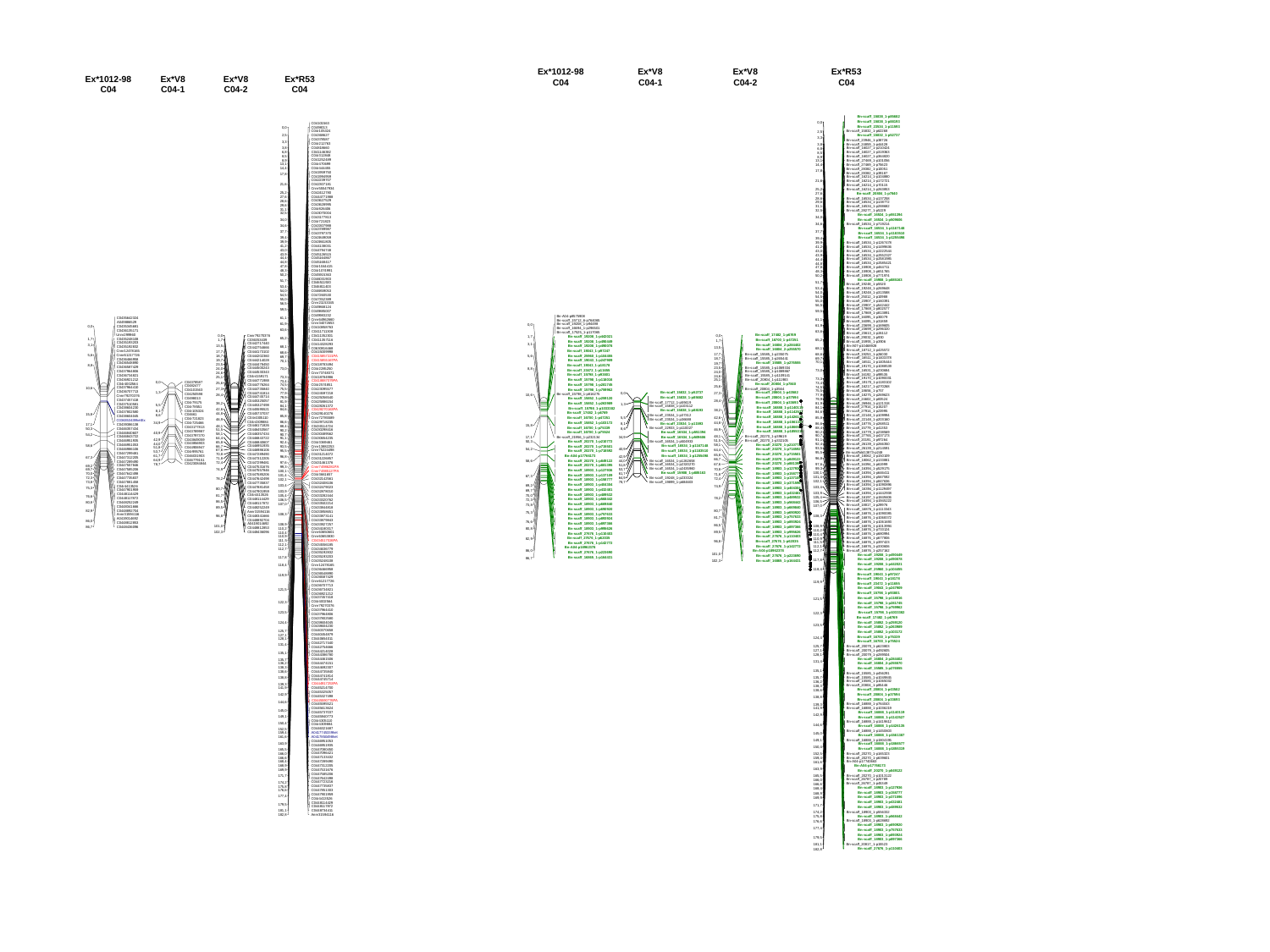

Ex*1012-98
C04
Ex*V8
C04-1
Ex*V8
C04-2
Ex*R53
C04
Bn-scaff_15838_1-p85682
Bn-scaff_15838_1-p88193
0,0
Bn-scaff_23534_1-p11593
Bn-scaff_15832_1-p62268
2,5
Bn-scaff_15832_1-p52737
3,3
Bn-scaff_23946_1-p38726
Bn-scaff_24859_1-p44428
3,8
Bn-scaff_16027_1-p210424
6,8
Bn-scaff_16027_1-p319363
8,5
Bn-scaff_16027_1-p364820
8,9
Bn-scaff_27469_1-p101056
13,1
Bn-scaff_27469_1-p75623
14,4
Bn-scaff_28382_1-p10051
17,8
Bn-scaff_28382_1-p39187
Bn-scaff_16214_1-p104880
Bn-scaff_16214_1-p172721
21,6
Bn-scaff_16214_1-p70115
Bn-scaff_16214_1-p263853
25,2
Bn-scaff_20804_1-p7840
27,6
Bn-scaff_16534_1-p137258
28,6
Bn-scaff_16534_1-p138772
29,6
Bn-scaff_16534_1-p288682
31,1
Bn-scaff_28277_1-p5229
32,5
Bn-scaff_16534_1-p551394
34,0
Bn-scaff_16534_1-p509606
Bn-scaff_16534_1-p719214
34,6
Bn-scaff_16534_1-p1167148
37,7
Bn-scaff_16534_1-p1183510
Bn-scaff_16534_1-p1255498
39,4
Bn-scaff_16534_1-p1267478
39,9
Bn-scaff_16534_1-p1499636
41,2
Bn-scaff_16534_1-p2222544
43,0
Bn-scaff_16534_1-p2552327
43,9
Bn-scaff_16534_1-p2581985
44,4
Bn-scaff_16534_1-p2585421
44,8
Bn-scaff_15908_1-p464711
47,8
Bn-scaff_15908_1-p651765
48,3
Bn-scaff_15908_1-p771974
50,2
Bn-scaff_15908_1-p885163
51,7
Bn-scaff_19248_1-p5520
Bn-scaff_19248_1-p269648
53,4
Bn-scaff_19248_1-p313588
54,0
Bn-scaff_25012_1-p10988
54,5
Bn-scaff_23907_1-p160391
55,0
Bn-scaff_23907_1-p542442
56,5
Bn-scaff_17869_1-p802577
59,5
Bn-scaff_17869_1-p813891
Bn-scaff_16095_1-p30079
61,1
Bn-scaff_16095_1-p31859
Bn-scaff_23699_1-p169605
61,9
Bn-scaff_23699_1-p296320
63,6
Bn-scaff_23613_1-p26112
Bn-scaff_29032_1-p930
65,2
Bn-scaff_15993_1-p3906
Bn-A07-p10468928
68,1
Bn-scaff_18712_1-p425572
Bn-scaff_19253_1-p26030
68,6
Bn-scaff_16511_1-p1403378
Bn-A04-p8575908
Bn-scaff_15712_6-p764065
Bn-scaff_19208_1-p94498
0,0
Bn-scaff_16694_1-p296501
Bn-scaff_17525_1-p117065
Bn-scaff_17482_1-p6769
0,0
Bn-scaff_19208_1-p442021
1,7
Bn-scaff_16700_1-p47251
1,7
Bn-scaff_19208_1-p490449
3,1
Bn-scaff_16804_2-p284402
Bn-scaff_19208_1-p490078
13,5
Bn-scaff_16804_2-p255570
Bn-scaff_19043_1-p97247
Bn-scaff_15585_1-p235075
17,7
Bn-scaff_25960_1-p104455
5,6
69,7
Bn-scaff_16511_1-p1405444
70,1
Bn-scaff_19170_1-p1088539
Bn-scaff_16531_1-p230684
73,0
Bn-scaff_16192_1-p99535
Bn-scaff_19170_1-p1090031
73,3
Bn-scaff_19170_1-p1183102
73,4
Bn-scaff_16217_1-p270268
74,5
Bn-scaff_18656_1-p757
75,5
Bn-scaff_18275_1-p269623
77,9
Bn-scaff_24602_1-p83524
79,9
Bn-scaff_26946_1-p121318
81,9
Bn-scaff_27914_1-p15207
84,1
Bn-scaff_27914_1-p23995
84,6
Bn-scaff_22148_1-p109994
85,8
Bn-scaff_22148_1-p203160
Bn-scaff_18776_1-p268511
86,6
Bn-scaff_15779_1-p41353
88,4
Bn-scaff_15779_1-p238569
90,2
Bn-scaff_27765_1-p437173
90,7
Bn-scaff_20191_1-p97264
91,1
Bn-scaff_26139_1-p266350
92,4
Bn-scaff_26139_1-p214591
93,5
Bn-scaffold13873-p248
95,5
Bn-scaff_18062_1-p150109
96,8
Bn-scaff_18062_1-p133881
Bn-scaff_16394_1-p61999
97,6
Bn-scaff_16394_1-p529175
99,3
Bn-scaff_16394_1-p646411
100,1
Bn-scaff_16394_1-p567592
101,4
Bn-scaff_16394_1-p847636
102,1
Bn-scaff_16394_1-p1090896
103,4
Bn-scaff_16394_1-p1129497
Bn-scaff_16394_1-p1442938
103,9
Bn-scaff_16197_1-p1849406
105,4
Bn-scaff_16394_1-p1945222
106,5
Bn-scaff_18817_1-p29976
107,0
Bn-scaff_16876_1-p1113343
Bn-scaff_16876_1-p1090085
108,3
Bn-scaff_16876_1-p1068372
Bn-scaff_16876_1-p1061693
Bn-scaff_16876_1-p1013994
108,9
Bn-scaff_16876_1-p733124
110,2
Bn-scaff_16876_1-p680994
110,4
Bn-scaff_16876_1-p677806
110,9
Bn-scaff_16876_1-p397423
111,5
Bn-scaff_16876_1-p330606
112,1
Bn-scaff_16876_1-p257162
112,7
Bn-scaff_19208_1-p490449
Bn-scaff_19208_1-p490078
117,8
Bn-scaff_19208_1-p442021
Bn-scaff_25960_1-p104455
118,4
Bn-scaff_19043_1-p97247
Bn-scaff_19043_1-p18178
119,9
Bn-scaff_23472_1-p11655
Bn-scaff_19043_1-p247909
Bn-scaff_15798_1-p93801
Bn-scaff_15798_1-p118016
121,5
Bn-scaff_15798_1-p281745
Bn-scaff_15798_1-p759962
Bn-scaff_15798_1-p1033382
Bn-scaff_15585_1-p265441
18,7
Bn-scaff_19043_1-p247909
Bn-scaff_15585_1-p276555
19,7
Bn-scaff_19043_1-p18178
Bn-scaff_15585_1-p1069334
23,5
8,8
Bn-scaff_23472_1-p11655
Bn-scaff_15585_1-p1089867
24,0
Bn-scaff_15798_1-p93801
Bn-scaff_15585_1-p1109141
24,6
Bn-scaff_20804_1-p111983
Bn-scaff_15798_1-p118016
25,1
Bn-scaff_20804_1-p7840
Bn-scaff_15798_1-p281745
25,6
Bn-scaff_20804_1-p3564
Bn-scaff_15798_1-p759962
Bn-scaff_15832_1-p52737
Bn-scaff_20804_1-p43582
0,0
27,3
Bn-scaff_15798_1-p816275
10,6
Bn-scaff_15838_1-p85682
Bn-scaff_20804_1-p37594
Bn-scaff_15852_1-p259120
28,4
Bn-scaff_17712_1-p59419
Bn-scaff_20804_1-p33693
Bn-scaff_15852_1-p263989
1,3
Bn-scaff_15838_1-p103112
Bn-scaff_16888_1-p1140119
Bn-scaff_15798_1-p1033382
Bn-scaff_15838_1-p88193
38,2
Bn-scaff_16888_1-p1142527
Bn-scaff_17482_1-p6769
Bn-scaff_23534_1-p37312
Bn-scaff_16888_1-p1426135
5,5
42,6
Bn-scaff_16700_1-p47251
Bn-scaff_23534_1-p36688
Bn-scaff_16888_1-p1561387
44,8
Bn-scaff_15852_1-p103172
Bn-scaff_23534_1-p11593
8,1
15,8
Bn-scaff_16888_1-p1866577
Bn-scaff_16700_1-p75339
Bn-scaff_22933_1-p110027
8,6
46,9
Bn-scaff_16888_1-p1855319
Bn-scaff_16700_1-p75524
Bn-scaff_16534_1-p551394
Bn-scaff_20270_1-p39619
48,1
Bn-scaff_16534_1-p509606
Bn-scaff_21956_1-p232134
17,1
34,9
Bn-scaff_20270_1-p332105
51,5
Bn-scaff_20270_1-p210772
50,3
Bn-scaff_16534_1-p504933
Bn-scaff_20270_1-p210772
58,1
Bn-scaff_16534_1-p1167148
Bn-scaff_20270_1-p715501
54,2
40,9
Bn-scaff_20270_1-p718592
64,4
Bn-scaff_16534_1-p1183510
Bn-scaff_20270_1-p718592
Bn-scaff_20270_1-p715501
65,8
Bn-A04-p17766173
Bn-scaff_16534_1-p1255498
42,9
Bn-scaff_20270_1-p849122
66,7
Bn-scaff_20270_1-p849122
Bn-scaff_16534_1-p1262658
44,0
58,6
Bn-scaff_20270_1-p881395
67,6
Bn-scaff_16534_1-p2320270
51,8
Bn-scaff_20270_1-p881395
Bn-scaff_18903_1-p127936
Bn-scaff_16534_1-p2435960
53,7
70,8
Bn-scaff_18903_1-p127936
Bn-scaff_15908_1-p885163
61,7
Bn-scaff_18903_1-p158777
71,8
Bn-scaff_18903_1-p137109
67,3
Bn-scaff_19248_1-p233324
64,9
Bn-scaff_18903_1-p137109
72,4
Bn-scaff_18903_1-p158777
Bn-scaff_19899_1-p684603
78,7
Bn-scaff_18903_1-p371596
Bn-scaff_18903_1-p404304
69,2
74,9
Bn-scaff_18903_1-p404304
Bn-scaff_18903_1-p432481
69,7
Bn-scaff_18903_1-p432481
Bn-scaff_18903_1-p489532
70,0
Bn-scaff_18903_1-p489532
78,2
Bn-scaff_18903_1-p568442
72,3
Bn-scaff_18903_1-p568442
Bn-scaff_18903_1-p669840
73,8
Bn-scaff_18903_1-p669840
Bn-scaff_18903_1-p690920
80,7
75,3
Bn-scaff_18903_1-p690920
Bn-scaff_18903_1-p757633
Bn-scaff_18903_1-p757633
81,7
Bn-scaff_18903_1-p893924
76,6
Bn-scaff_18903_1-p893924
Bn-scaff_18903_1-p897366
86,5
Bn-scaff_18903_1-p897366
Bn-scaff_18903_1-p995626
80,8
Bn-scaff_18903_1-p995626
89,5
Bn-scaff_27676_1-p110403
Bn-scaff_27676_1-p110403
Bn-scaff_27676_1-p62035
82,9
Bn-scaff_27676_1-p62035
96,8
Bn-scaff_27676_1-p142772
Bn-scaff_27676_1-p142772
Bn-A04-p18962378
86,0
Bn-A04-p18962378
Bn-scaff_27676_1-p223690
101,0
Bn-scaff_27676_1-p223690
Bn-scaff_16865_1-p164431
86,7
Bn-scaff_16865_1-p164431
102,3
122,3
Bn-scaff_17482_1-p6769
Bn-scaff_15852_1-p259120
123,5
Bn-scaff_15852_1-p263989
Bn-scaff_15852_1-p103172
Bn-scaff_16700_1-p75339
124,4
Bn-scaff_16700_1-p75524
Bn-scaff_20079_1-p623803
125,7
Bn-scaff_20079_1-p492605
127,1
Bn-scaff_20079_1-p289504
128,1
Bn-scaff_16804_2-p284402
131,4
Bn-scaff_16804_2-p255570
Bn-scaff_15585_1-p276555
135,1
Bn-scaff_15585_1-p456291
Bn-scaff_15585_1-p1049845
135,7
Bn-scaff_15585_1-p1065032
136,2
Bn-scaff_20804_1-p85446
138,3
Bn-scaff_20804_1-p43582
138,6
Bn-scaff_20804_1-p37594
138,8
Bn-scaff_20804_1-p33693
Bn-scaff_16888_1-p764043
139,3
Bn-scaff_16888_1-p1036219
141,9
Bn-scaff_16888_1-p1140119
142,9
Bn-scaff_16888_1-p1142527
Bn-scaff_16888_1-p1419612
144,6
Bn-scaff_16888_1-p1426135
Bn-scaff_16888_1-p1450603
145,0
Bn-scaff_16888_1-p1561387
Bn-scaff_16888_1-p1804195
149,1
Bn-scaff_16888_1-p1866577
150,4
Bn-scaff_16888_1-p1855319
Bn-scaff_20270_1-p165323
152,5
Bn-scaff_20270_1-p639601
159,4
Bn-A04-p17740663
161,6
Bn-A04-p17766173
163,9
Bn-scaff_20270_1-p849122
Bn-scaff_20270_1-p1013122
165,5
Bn-scaff_26787_1-p20789
166,0
Bn-scaff_26787_1-p45349
166,6
Bn-scaff_18903_1-p127936
168,4
Bn-scaff_18903_1-p158777
168,9
Bn-scaff_18903_1-p371596
169,9
Bn-scaff_18903_1-p432481
171,7
Bn-scaff_18903_1-p489532
Bn-scaff_18903_1-p556332
174,2
Bn-scaff_18903_1-p568442
175,8
Bn-scaff_18903_1-p628692
176,6
Bn-scaff_18903_1-p690920
177,4
Bn-scaff_18903_1-p757633
Bn-scaff_18903_1-p893924
179,5
Bn-scaff_18903_1-p897366
Bn-scaff_20817_1-p15523
181,1
Bn-scaff_27676_1-p110403
182,8
Ex*1012-98
C04
Ex*V8
C04-1
Ex*V8
C04-2
Ex*R53
C04
C04101563
C0498013
0,0
C04r105324
C04368627
2,5
C04378587
3,3
C04r212763
C04818660
3,8
C041146382
6,8
C04r312848
8,5
C041252489
8,9
C04r470699
13,1
C04r444455
14,4
C041959750
17,8
C041994959
C042239707
C042307181
21,6
Cnnr55547934
C042412780
25,2
C0444771988
27,6
C043627529
28,6
C043628995
29,6
C04r826406
31,1
C043070004
32,5
C043177813
34,0
C04r721823
C043307988
34,6
C043789987
37,7
C043797370
C043849059
39,4
C043861805
39,9
C044138031
41,2
C044794748
43,0
C045126515
43,9
C045164867
44,4
C045168417
44,8
C04r1044415
47,8
C04r1074991
48,3
C045915363
50,2
C046031903
51,7
C046511920
C046811403
53,4
C046859053
54,0
C047260530
54,5
C047352389
55,0
Cnnr21153345
56,5
C049868124
59,5
C049885007
C049983232
61,1
Cnnr64962660
Cnnr34072653
61,9
C0410858763
63,6
C0411711308
C0411352301
65,2
C0411357116
C0414328293
68,1
C0630816468
C0415009998
68,6
C0415857221PA
C0435642324
A049886529
C0435345681
0,0
C0436135171
Unnr299960
Cnnr79270376
0,0
C0435248108
1,7
C036353439
1,7
C0435193203
C0442717440
3,1
13,5
C0435192832
C0442754666
Cnnr12478165
C0444173102
17,7
Cnnr61217726
5,6
C0444202360
18,7
69,7
C0415855167PA
70,1
C0418783494
C04r2285250
73,0
Cnnr73748371
C0418784986
73,3
C0418887079PA
73,4
C04r2574851
74,5
C0423095577
75,5
C0424597218
77,9
C0425056540
79,9
C0425886154
81,9
C0428261372
84,1
C0428270163PA
84,6
C0429140276
85,8
Cnnr72785589
C0429718235
86,6
C0430114724
88,4
C0430298416
90,2
C0430489562
90,7
C0430654235
91,1
C04r3369461
92,4
Cnnr13882253
93,5
Cnnr75224498
95,5
C0431214072
96,8
C0431226857
C0431461376
97,6
Cnnr74986281PA
99,3
Cnnr74985127PA
100,1
C04r3661657
101,4
C0432142561
102,1
C0432408106
103,4
C0432479023
C0432878010
103,9
C0433282444
105,4
C0433320762
106,5
C0433502214
107,0
C0433844818
C0433858651
108,3
C0433873141
C0433879843
C0433927257
108,9
C0434180317
110,2
Cnnr63650601
110,4
Cnnr63653830
110,9
C0434517026PA
111,5
C0434556185
112,1
C0434636779
112,7
C0435192832
C0435193203
117,8
C0435248108
Cnnr12478165
118,4
C0436466958
C0436548890
119,9
C0436587429
Cnnr61217726
C0436707713
C0436734821
121,5
C0436921212
C0437457418
C04r4002564
C0436466958
C0444214028
19,7
C0436548890
C0444478450
23,5
8,8
C0436587429
C0444500243
24,0
C0437964806
C0444530343
24,6
C0436734821
C04r4159171
25,1
C0436921212
C0444771988
C04378587
0,0
25,6
C04r4002564
C0444776264
C0482477
C0437964410
10,6
C0444735840
27,3
C04101563
C0436707713
1,3
C0444741814
C04250598
28,4
Cnnr79270376
C0444745714
C0498013
C0437457418
C0445325057
C04r79175
38,2
C0437510581
5,5
C0445327498
C04r78551
C0438604230
C0445595521
42,6
C04r105324
8,1
C0437802580
C0445737037
44,8
15,8
C04661
8,6
C0438604045
C04r4305110
C04r721823
46,9
C036353439hetEx
C04r4309884
C04r725466
34,9
C0439386138
17,1
C0446171826
48,1
C043177813
C0446357434
50,3
C0446453567
51,5
C043789987
C0446840607
40,9
C0446357434
58,1
54,2
C043797370
C0446843722
C0446843722
64,4
C043849059
42,9
C0446951935
C0446840607
65,8
C043856903
44,0
C0446951053
58,6
C0446951935
66,7
C044906947
51,8
C0446986106
C0446986106
67,6
C04r995761
53,7
C0447299481
C0447289490
70,8
C046031903
61,7
C0447312205
67,3
C0447312205
71,8
C046779151
64,9
C0447289490
C0447299481
72,4
C0423084844
78,7
C0447557846
69,2
C0447531676
74,9
C0447585206
69,7
C0447557846
C0447642498
70,0
C0447585206
C0447735837
72,3
C0447642498
78,2
C0447881458
73,8
C0447735837
C04r4413526
C0447881458
75,3
80,7
C0447901959
C0447901959
C0448114429
C04r4413526
81,7
76,6
C0448117872
C0448114429
86,5
C0448252249
80,8
C0448117872
C0448341666
C0448252249
89,5
C0448892704
82,9
Annr31594116
Annr31594116
C0448341666
96,8
A0419014692
C0448892704
86,0
C0448812853
A0419014692
101,0
C0448436096
86,7
C0448812853
C0448436096
102,3
122,3
Cnnr79270376
C0437964410
123,5
C0437964806
C0437802580
C0438604045
124,4
C0438604230
C0440370658
125,7
C0440454879
127,1
C0440654011
128,1
C0442717440
131,4
C0442754666
C0444214028
135,1
C0444386780
C0444461506
135,7
C0444474151
136,2
C0444692307
138,3
C0444735840
138,6
C0444741814
138,8
C0444745714
C0444917253PA
139,3
C0445214700
141,9
C0445325057
142,9
C0445327498
C0445590776PA
144,6
C0445595521
C0445613624
145,0
C0445737037
C0445940773
149,1
C04r4305110
150,4
C04r4309884
C0446321687
152,5
A0417745339het
159,4
A0417850498het
161,6
C0446951053
163,9
C0446951935
C0447080450
165,5
C0447096421
166,0
C0447133432
166,6
C0447289490
168,4
C0447312205
168,9
C0447531676
169,9
C0447585206
171,7
C0447642498
C0447723216
174,2
C0447735837
175,8
C0447851303
176,6
C0447901959
177,4
C04r4413526
C0448114429
179,5
C0448117872
C0448734411
181,1
Annr31594116
182,8

## Slide 15
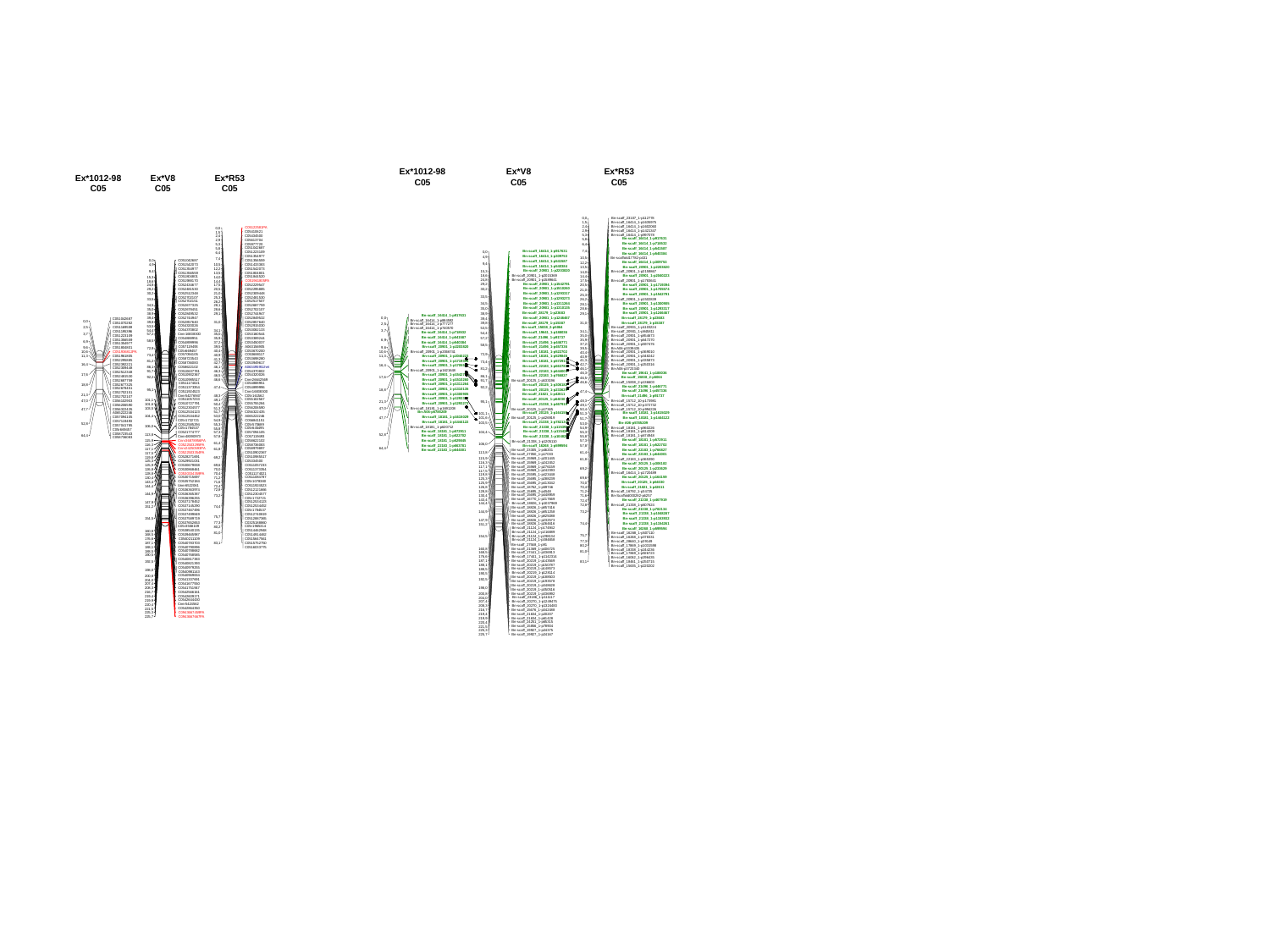

Ex*1012-98
C05
Ex*V8
C05
Ex*R53
C05
Bn-scaff_23107_1-p112778
0,0
Bn-scaff_16414_1-p1635975
1,5
Bn-scaff_16414_1-p1602060
2,4
Bn-scaff_16414_1-p1421347
2,9
Bn-scaff_16414_1-p997078
5,3
Bn-scaff_16414_1-p917631
5,8
Bn-scaff_16414_1-p718532
6,4
Bn-scaff_16414_1-p541587
7,4
Bn-scaff_16414_1-p540384
Bn-scaffold17782-p431
10,5
Bn-scaff_16414_1-p309753
12,2
Bn-scaff_20901_1-p2203820
13,5
Bn-scaff_20901_1-p2159867
14,0
Bn-scaff_20901_1-p2040223
14,4
Bn-scaff_20901_1-p1783641
17,5
Bn-scaff_20901_1-p1719394
20,5
Bn-scaff_20901_1-p1705574
21,0
Bn-scaff_20901_1-p1542791
25,3
Bn-scaff_20901_1-p1502839
26,2
Bn-scaff_20901_1-p1300905
28,1
Bn-scaff_20901_1-p1293317
28,6
Bn-scaff_20901_1-p1246467
29,1
Bn-scaff_28179_1-p23683
Bn-scaff_28179_1-p15387
31,0
Bn-scaff_20901_1-p1103224
Bn-scaff_20901_1-p945811
34,1
Bn-scaff_20901_1-p854673
35,0
Bn-scaff_20901_1-p647270
35,9
Bn-scaff_20901_1-p597476
37,2
Bn-A06-p3339435
39,5
Bn-scaff_20901_1-p369010
40,4
Bn-scaff_20901_1-p348242
40,9
Bn-scaff_20901_1-p335873
41,3
Bn-scaff_20901_1-p250316
42,7
Bn-A06-p3723340
46,1
Bn-scaff_19641_1-p188036
46,3
Bn-scaff_15838_2-p5064
46,5
Bn-scaff_15838_2-p136603
46,6
Bn-scaff_21496_1-p446771
Bn-scaff_21496_1-p457336
47,4
Bn-scaff_21496_1-p91737
Bn-scaff_15712_10-p173981
48,3
Bn-scaff_15712_10-p372732
49,1
Bn-scaff_15712_10-p994226
50,4
Bn-scaff_18181_1-p1615029
51,3
Bn-scaff_18181_1-p1444122
51,7
Bn-A06-p5785209
53,0
Bn-scaff_18181_1-p960226
54,9
Bn-scaff_18181_1-p814209
55,3
Bn-scaff_18181_1-p674948
55,8
Bn-scaff_18181_1-p572911
57,3
Bn-scaff_18181_1-p522702
57,8
Bn-scaff_22183_1-p766827
61,4
Bn-scaff_22183_1-p644001
Bn-scaff_16414_1-p917631
0,0
Bn-scaff_16414_1-p309753
4,9
Bn-scaff_16414_1-p541587
9,4
Bn-scaff_16414_1-p540384
Bn-scaff_20901_1-p2203820
15,3
Bn-scaff_20901_1-p2015369
18,6
Bn-scaff_20901_1-p1589641
24,8
Bn-scaff_20901_1-p1542791
29,2
Bn-scaff_20901_1-p1510260
30,2
Bn-scaff_20901_1-p1293317
33,5
Bn-scaff_20901_1-p1293273
Bn-scaff_20901_1-p1311264
34,5
Bn-scaff_20901_1-p1310135
35,0
Bn-scaff_28179_1-p23683
38,9
Bn-scaff_20901_1-p1246467
39,4
Bn-scaff_28179_1-p15387
39,8
Bn-scaff_15838_2-p5064
53,5
Bn-scaff_19641_1-p188036
54,4
Bn-scaff_21496_1-p91737
57,2
Bn-scaff_21496_1-p446771
58,5
Bn-scaff_21496_1-p457336
Bn-scaff_18181_1-p522702
72,9
Bn-scaff_18181_1-p529845
Bn-scaff_18181_1-p572911
73,4
Bn-scaff_22183_1-p663781
81,2
Bn-scaff_22183_1-p644001
Bn-scaff_22183_1-p766827
86,1
Bn-scaff_20125_1-p533196
91,7
Bn-scaff_20125_1-p306182
92,2
Bn-scaff_20125_1-p233629
Bn-scaff_21821_1-p42611
Bn-scaff_20125_1-p64030
95,1
Bn-scaff_21338_1-p467919
Bn-scaff_20125_1-p17365
Bn-scaff_20125_1-p164159
101,1
Bn-scaff_20125_1-p426919
101,6
Bn-scaff_21338_1-p782134
103,5
Bn-scaff_21338_1-p1153932
Bn-scaff_21338_1-p1154261
104,4
Bn-scaff_21338_1-p1048287
Bn-scaff_21338_1-p1205110
106,0
Bn-scaff_16268_1-p599594
Bn-scaff_23305_1-p46201
113,8
Bn-scaff_27050_1-p17033
Bn-scaff_15969_1-p201445
115,9
Bn-scaff_15969_1-p242452
116,3
Bn-scaff_15969_1-p276159
117,1
Bn-scaff_15969_1-p242393
117,5
Bn-scaff_25595_1-p423448
119,8
Bn-scaff_15695_1-p288239
125,3
Bn-scaff_15695_2-p413042
125,9
Bn-scaff_18762_1-p59746
126,8
Bn-scaff_15695_2-p4548
128,8
Bn-scaff_15695_2-p448959
130,4
Bn-scaff_16770_1-p217669
143,4
Bn-scaff_18826_1-p1037969
144,4
Bn-scaff_18826_1-p857416
Bn-scaff_18826_1-p851258
144,9
Bn-scaff_18826_1-p825088
Bn-scaff_16414_1-p917631
0,0
Bn-scaff_16414_1-p884592
Bn-scaff_16414_1-p777277
2,5
Bn-scaff_16414_1-p740570
3,7
Bn-scaff_16414_1-p718532
Bn-scaff_16414_1-p541587
6,9
Bn-scaff_16414_1-p540384
Bn-scaff_20901_1-p2203820
9,6
Bn-scaff_20901_1-p2066741
10,6
Bn-scaff_20901_1-p2040223
11,3
Bn-scaff_20901_1-p1719394
Bn-scaff_20901_1-p1705574
16,4
Bn-scaff_20901_1-p1621648
Bn-scaff_20901_1-p1542791
17,6
Bn-scaff_20901_1-p1510260
Bn-scaff_20901_1-p1311264
Bn-scaff_20901_1-p1310135
18,8
Bn-scaff_20901_1-p1300905
Bn-scaff_20901_1-p1293317
21,3
Bn-scaff_20901_1-p1293273
Bn-scaff_18181_1-p1691208
47,0
Bn-A06-p5785209
Bn-scaff_18181_1-p1615029
47,7
Bn-scaff_18181_1-p1444122
Bn-scaff_18181_1-p620712
Bn-scaff_18181_1-p572911
52,8
Bn-scaff_18181_1-p522702
Bn-scaff_18181_1-p529845
Bn-scaff_22183_1-p663781
64,4
Bn-scaff_22183_1-p644001
Bn-scaff_22183_1-p365390
61,8
Bn-scaff_20125_1-p306182
Bn-scaff_20125_1-p233629
69,2
Bn-scaff_16414_1-p1720489
Bn-scaff_20125_1-p164159
69,6
Bn-scaff_20125_1-p64030
70,0
Bn-scaff_21821_1-p42611
70,4
Bn-scaff_16792_1-p34725
71,2
Bn-Scaffold000282-p6257
71,6
Bn-scaff_21338_1-p467919
72,4
Bn-scaff_21338_1-p607624
72,8
Bn-scaff_21338_1-p782134
73,2
Bn-scaff_21338_1-p1048287
Bn-scaff_21338_1-p1153932
Bn-scaff_18826_1-p232573
147,9
Bn-scaff_21338_1-p1154261
Bn-scaff_18826_1-p264616
74,4
151,2
Bn-scaff_21124_1-p174942
Bn-scaff_16268_1-p599594
Bn-scaff_21124_1-p216889
Bn-scaff_16268_1-p507110
75,7
Bn-scaff_21124_1-p298134
154,5
Bn-scaff_16268_1-p378331
Bn-scaff_21124_1-p384658
Bn-scaff_28640_1-p29149
77,3
Bn-scaff_27568_1-p91
Bn-scaff_17869_1-p1031598
80,2
Bn-scaff_21369_1-p406725
160,8
Bn-scaff_18338_1-p244236
81,0
Bn-scaff_17441_1-p336913
168,5
Bn-scaff_17869_1-p926723
Bn-scaff_17441_1-p1142314
176,6
Bn-scaff_16062_1-p296435
Bn-scaff_20219_1-p143569
187,1
Bn-scaff_18461_1-p250715
83,1
Bn-scaff_20219_1-p150787
188,1
Bn-scaff_15635_1-p220202
Bn-scaff_20219_1-p148573
188,5
Bn-scaff_20219_1-p128114
190,5
Bn-scaff_20219_1-p188503
192,5
Bn-scaff_20219_1-p193578
Bn-scaff_20219_1-p348628
198,0
Bn-scaff_20219_1-p350516
Bn-scaff_20219_1-p336992
200,8
Bn-scaff_23186_1-p111117
204,0
Bn-scaff_20270_1-p1249475
207,4
Bn-scaff_20270_1-p1324483
208,3
Bn-scaff_15676_1-p342488
216,7
Bn-scaff_21634_1-p20207
219,4
Bn-scaff_21634_1-p61428
219,9
Bn-scaff_24251_1-p65315
220,4
Bn-scaff_15856_1-p78904
221,5
Bn-scaff_19927_1-p24375
225,3
Bn-scaff_19927_1-p24167
225,7
Ex*1012-98
C05
Ex*V8
C05
Ex*R53
C05
C05122581PA
0,0
C05410621
1,5
C05434500
2,4
C05613704
2,9
C05977720
5,3
C051042687
5,8
C051223109
6,4
C051354977
7,4
C051356559
C051433383
10,5
C051542073
12,2
C051804801
13,5
C051844520
14,0
C051961805PA
14,4
C052229547
17,5
C052295885
20,5
C052309448
21,0
C052481530
25,3
C052517507
26,2
C052687759
28,1
C052702107
28,6
C052744947
29,1
C052849532
C052857640
31,0
C052915030
C053082133
34,1
C053160544
35,0
C053389244
35,9
C053450037
37,2
A063156905
39,5
C053670200
40,4
C053688117
40,9
C053698280
41,3
C053949617
42,7
A063495951het
46,1
C054370802
46,3
C054320026
46,5
Cnnr26642569
46,6
C054888951
C054899996
47,4
Cnnr16838300
C05r161562
48,3
C055102567
49,1
C055755284
50,4
C056206590
51,3
C056322435
51,7
A065222246
53,0
C056841151
54,9
C05r570689
55,3
C05r645495
55,8
C057094105
57,3
C057119493
57,8
C058622102
61,4
C058736083
C051042687
0,0
C051542073
4,9
C051354977
9,4
C051356559
C051804801
15,3
C051988170
18,6
C052434677
24,8
C052481530
29,2
C052512348
30,2
C052702107
33,5
C052702151
C052677325
34,5
C052678451
35,0
C052849532
38,9
C052744947
39,4
C052857640
39,8
C054320026
53,5
C054370802
54,4
Cnnr16838300
57,2
C054888951
58,5
C054899996
C057119493
72,9
C05r669457
C057094105
73,4
C058723543
81,2
C058736083
C058622102
86,1
C0510637781
91,7
C0510902367
92,2
C0510985517
C0511174021
C0511373354
95,1
C0511924523
Cnnr54276947
C0511057233
101,1
C0510727791
101,6
C0512304577
103,5
C0512534123
C0512534452
104,4
C05r1722721
C0512585294
106,0
C05r1784537
C0521774777
113,8
Cnnr48390973
Cnnr36878956PA
115,9
C0522503295PA
116,3
Cnnr24250593PA
117,1
C0522503354PA
117,5
C0528271691
119,8
C0529921031
125,3
C0530679838
125,9
C0530868461
126,8
C0530334359PA
128,8
C0530715697
130,4
C0535752184
143,4
Unnr6522061
144,4
C0536363974
C0536365387
144,9
C0536396255
C051042687
0,0
C051070282
C051169559
2,5
C051195396
3,7
C051223109
C051356559
6,9
C051354977
C051804801
9,6
C051936812PA
10,6
C051961805
11,3
C052295885
C052392221
16,4
C052309448
C052512348
17,6
C052481530
C052687759
C052677325
18,8
C052678451
C052702151
21,3
C052702107
C056102903
47,0
C056206590
C056322435
47,7
A065222246
C057094105
C057119493
52,8
C057041785
C05r669457
C058723543
64,4
C058736083
C058979897
61,8
C0510902367
C0510985517
69,2
C05334500
C0511057233
69,6
C0511373354
70,0
C0511174021
70,4
C0511084787
71,2
C05r1078380
71,6
C0511924523
72,4
C0512121666
72,8
C0512304577
73,2
C05r1722721
C0537178452
C0512534123
147,9
C0537145290
C0512534452
151,2
74,4
C0537467496
C05r1784537
C0537499849
C0512740819
75,7
C0537589719
C0512887365
154,5
C0537652653
C0325188860
77,3
C05r3558109
C05r1965314
80,2
C0538540135
C0514462948
160,8
81,0
C0539465987
C0514914462
168,5
C0540211109
C0515647561
176,6
C0540783703
C0515752750
187,1
83,1
C0540790896
C0516033775
188,1
C0540788682
188,5
C0540768585
190,5
C0540817383
192,5
C0540821393
C0540979255
198,0
C0540981143
C0540969834
200,8
C0541337691
204,0
C0541677550
207,4
C0541751947
208,3
C0542566161
216,7
C0542609171
219,4
C0542644430
219,9
Cnnr5424562
220,4
C0542864350
221,5
C0943667459PA
225,3
C0943667667PA
225,7

## Slide 16
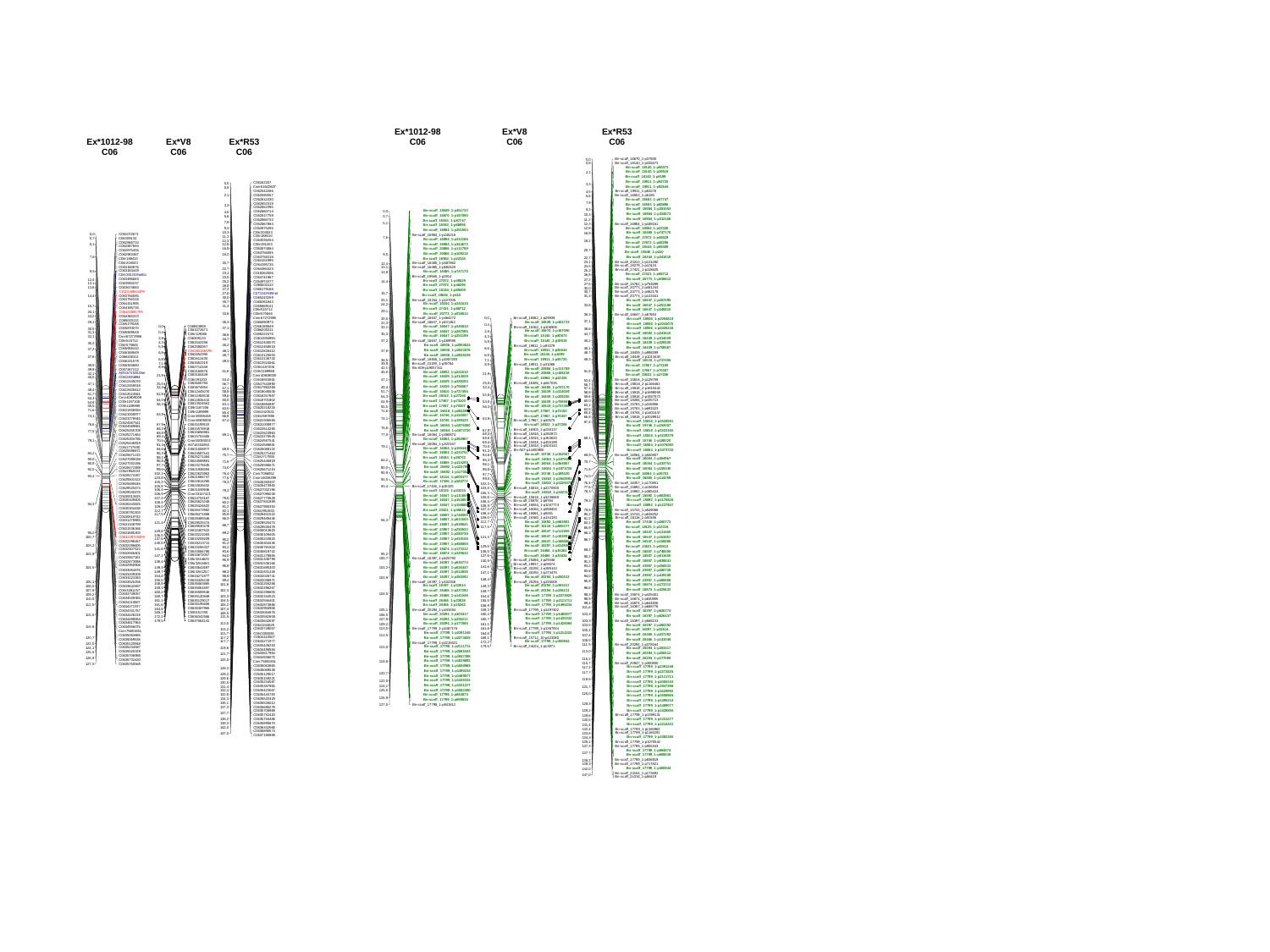

Ex*1012-98
C06
Ex*V8
C06
Ex*R53
C06
Bn-scaff_16670_1-p37930
0,0
Bn-scaff_18140_1-p335573
0,8
Bn-scaff_18140_1-p80073
Bn-scaff_18140_1-p39519
2,1
Bn-scaff_18140_1-p8199
Bn-scaff_19911_1-p92720
3,3
Bn-scaff_19911_1-p92644
Bn-scaff_19911_1-p84276
4,5
Bn-scaff_16553_1-p6185
5,6
Bn-scaff_16553_1-p87747
7,9
Bn-scaff_16553_1-p88896
Bn-scaff_16984_1-p351553
9,3
Bn-scaff_16984_1-p324673
10,3
Bn-scaff_16984_1-p312166
11,2
Bn-scaff_16984_1-p188161
12,3
Bn-scaff_16984_1-p22326
12,8
Bn-scaff_16485_1-p747170
16,9
Bn-scaff_27872_1-p48529
18,2
Bn-scaff_27872_1-p48296
Bn-scaff_18344_1-p89409
20,7
Bn-scaff_19646_1-p410
Bn-scaff_18344_1-p341615
22,7
Bn-scaff_20210_1-p124280
23,1
Bn-scaff_18279_1-p10124
23,5
Bn-scaff_27421_1-p118625
25,2
Bn-scaff_27421_1-p88712
26,8
Bn-scaff_20773_1-p538812
27,2
Bn-scaff_15762_1-p754699
27,6
Bn-scaff_20773_1-p481244
30,0
Bn-scaff_20773_1-p462178
30,7
Bn-scaff_20773_1-p123341
31,3
Bn-scaff_16647_1-p287095
Bn-scaff_16647_1-p251198
33,8
Bn-scaff_16647_1-p345810
Bn-scaff_16647_1-p67654
36,3
Bn-scaff_18936_1-p2054624
37,1
Bn-scaff_18936_1-p2043076
Bn-scaff_18936_1-p2026238
38,6
Bn-scaff_18002_1-p241610
44,7
Bn-scaff_18439_1-p314029
45,2
Bn-scaff_18439_1-p329200
Bn-scaff_18439_1-p706567
48,1
Bn-scaff_18439_1-p998299
48,7
Bn-scaff_18439_1-p1013430
49,3
Bn-scaff_16510_1-p737455
Bn-scaff_17867_1-p73100
Bn-scaff_17867_1-p70307
51,8
Bn-scaff_28522_1-p27206
Bn-scaff_15818_2-p128759
53,4
Bn-scaff_15818_2-p1166462
56,7
Bn-scaff_15818_2-p1610442
57,1
Bn-scaff_15818_2-p1698069
58,8
Bn-scaff_15818_2-p1927573
59,6
Bn-scaff_18206_1-p435713
60,0
Bn-scaff_15763_1-p316906
63,1
Bn-scaff_15763_1-p681523
63,5
Bn-scaff_15763_1-p1401447
65,4
Bn-scaff_18549_1-p451733
0,0
Bn-scaff_16670_1-p167090
0,7
Bn-scaff_16553_1-p87747
5,1
Bn-scaff_16553_1-p88896
Bn-scaff_16984_1-p351553
Bn-scaff_16984_1-p245218
7,6
Bn-scaff_16984_1-p312166
Bn-scaff_16984_1-p324673
Bn-scaff_20866_1-p111789
Bn-scaff_20866_1-p108216
9,5
Bn-scaff_16984_1-p22326
Bn-scaff_16485_1-p587862
12,4
Bn-scaff_16485_1-p682420
13,1
Bn-scaff_16485_1-p747170
13,8
Bn-scaff_19569_1-p2814
Bn-scaff_27872_1-p48529
14,4
Bn-scaff_27872_1-p48296
Bn-scaff_18344_1-p89409
15,7
Bn-scaff_19646_1-p410
Bn-scaff_18344_1-p227005
20,1
Bn-scaff_18344_1-p341615
24,2
Bn-scaff_27421_1-p88712
29,1
Bn-scaff_20773_1-p538812
Bn-scaff_16647_1-p464272
30,5
Bn-scaff_16647_1-p431162
31,3
Bn-scaff_16647_1-p345810
32,1
Bn-scaff_16647_1-p287095
35,3
Bn-scaff_16647_1-p251198
Bn-scaff_16647_1-p169595
37,2
Bn-scaff_18936_1-p2054624
Bn-scaff_18936_1-p2043076
37,8
Bn-scaff_18936_1-p2026238
Bn-scaff_18936_1-p1957293
38,5
Bn-scaff_23199_1-p39784
39,9
Bn-A09-p19057312
42,1
Bn-scaff_18002_1-p241610
45,0
Bn-scaff_18439_1-p314029
47,1
Bn-scaff_18439_1-p329200
Bn-scaff_18439_1-p706567
48,4
Bn-scaff_16510_1-p737455
51,7
Bn-scaff_28522_1-p27206
54,3
Bn-scaff_17867_1-p73100
54,9
Bn-scaff_17867_1-p70307
55,5
Bn-scaff_15818_1-p2528591
71,6
Bn-scaff_15746_1-p152007
74,1
Bn-scaff_15746_1-p189320
Bn-scaff_16064_1-p1076060
76,6
Bn-scaff_16064_1-p1073720
Bn-scaff_16064_1-p380874
77,5
Bn-scaff_16064_1-p364967
Bn-scaff_16064_1-p322157
79,1
Bn-scaff_16064_1-p155340
Bn-scaff_16064_1-p233751
Bn-scaff_16064_1-p26703
84,2
Bn-scaff_16865_1-p114259
Bn-scaff_15892_1-p1237907
90,0
Bn-scaff_15892_1-p1170828
90,9
Bn-scaff_16116_1-p605377
92,5
Bn-scaff_17436_1-p260772
Bn-scaff_17436_1-p26325
93,4
Bn-scaff_18125_1-p33315
Bn-scaff_16547_1-p111855
Bn-scaff_16547_1-p153857
Bn-scaff_16547_1-p158886
Bn-scaff_23821_1-p45612
Bn-scaff_18807_1-p748005
Bn-scaff_18807_1-p611600
Bn-scaff_18807_1-p539841
Bn-scaff_18362_1-p28939
0,0
Bn-scaff_18549_1-p451733
0,4
Bn-scaff_18362_1-p106906
Bn-scaff_16670_1-p167090
2,8
Bn-scaff_18140_1-p80073
4,3
Bn-scaff_18140_1-p39519
5,9
Bn-scaff_19911_1-p93379
6,6
Bn-scaff_19911_1-p92644
Bn-scaff_18140_1-p8199
6,8
Bn-scaff_19911_1-p92720
7,1
Bn-scaff_19911_1-p31068
8,9
Bn-scaff_20866_1-p111789
Bn-scaff_20866_1-p108216
21,9
Bn-scaff_16984_1-p22326
Bn-scaff_16485_1-p647035
25,5
Bn-scaff_16485_1-p747170
32,4
Bn-scaff_18439_1-p314029
52,8
Bn-scaff_18439_1-p329200
Bn-scaff_18439_1-p706567
54,0
Bn-scaff_16510_1-p737455
56,3
Bn-scaff_17867_1-p73100
Bn-scaff_17867_1-p70307
63,9
Bn-scaff_17867_1-p40575
Bn-scaff_28522_1-p27206
Bn-scaff_16903_1-p230137
67,9
Bn-scaff_15818_1-p292872
68,3
Bn-scaff_15818_1-p363620
68,8
Bn-scaff_15818_1-p455199
69,4
Bn-scaff_15818_1-p503143
70,5
Bn-A07-p14450900
91,3
Bn-scaff_15746_1-p152007
94,6
Bn-scaff_16064_1-p1076060
95,3
Bn-scaff_16064_1-p364967
96,1
Bn-scaff_16064_1-p1073720
96,8
Bn-scaff_15746_1-p189320
97,7
Bn-scaff_15818_1-p2528591
99,0
Bn-scaff_15818_1-p3229379
102,3
Bn-scaff_15818_1-p2270500
103,8
Bn-scaff_15818_1-p3223160
105,3
Bn-scaff_15818_1-p3239659
105,8
Bn-scaff_25878_1-p8784
106,4
Bn-scaff_16064_1-p1047774
106,9
Bn-scaff_16064_1-p938404
107,4
Bn-scaff_16064_1-p9281
108,4
Bn-scaff_18360_1-p134193
109,0
Bn-scaff_15892_1-p563981
112,7
Bn-scaff_16116_1-p605377
117,5
Bn-scaff_16547_1-p111855
Bn-scaff_16547_1-p153857
121,4
Bn-scaff_16547_1-p158886
Bn-scaff_16397_1-p514936
129,6
Bn-scaff_25466_1-p15362
136,5
Bn-scaff_25466_1-p33838
137,6
Bn-scaff_25466_1-p29346
140,8
Bn-scaff_19937_1-p28874
141,6
Bn-scaff_20294_1-p395143
Bn-scaff_20294_1-p273475
Bn-scaff_15818_1-p2029912
66,8
Bn-scaff_15818_1-p2528591
67,4
Bn-scaff_15746_1-p152007
Bn-scaff_15818_1-p3223160
Bn-scaff_15818_1-p3229379
69,1
Bn-scaff_15746_1-p189320
Bn-scaff_16064_1-p1076060
Bn-scaff_16064_1-p1073720
Bn-scaff_16064_1-p550807
69,9
Bn-scaff_16064_1-p364967
70,7
Bn-scaff_16064_1-p233751
Bn-scaff_16064_1-p155340
71,8
Bn-scaff_16064_1-p26703
74,0
Bn-scaff_16865_1-p114259
Bn-scaff_16357_1-p170851
76,4
Bn-scaff_15892_1-p156854
77,6
Bn-scaff_15892_1-p365424
78,3
Bn-scaff_15892_1-p563981
Bn-scaff_15892_1-p1170828
79,3
Bn-scaff_15892_1-p1237907
Bn-scaff_15743_1-p549066
79,8
Bn-scaff_15743_1-p638252
80,2
Bn-scaff_16116_1-p50186
81,2
Bn-scaff_17436_1-p260772
82,1
Bn-scaff_18125_1-p33315
85,9
Bn-scaff_16547_1-p111855
86,3
Bn-scaff_16547_1-p153857
86,7
Bn-scaff_16547_1-p158886
Bn-scaff_23821_1-p45612
89,2
Bn-scaff_18807_1-p748005
Bn-scaff_18807_1-p611600
90,2
Bn-scaff_18807_1-p539841
91,2
Bn-scaff_23957_1-p250533
93,2
Bn-scaff_23957_1-p280738
93,6
Bn-scaff_23957_1-p419108
94,0
Bn-scaff_23957_1-p488858
95,9
Bn-scaff_16874_1-p173312
96,6
Bn-scaff_16874_1-p325632
Bn-scaff_16874_1-p425401
98,3
Bn-scaff_16874_1-p630905
98,9
Bn-scaff_16874_1-p644836
99,4
Bn-scaff_16397_1-p668776
101,6
Bn-scaff_16397_1-p626774
102,3
Bn-scaff_16397_1-p626437
Bn-scaff_16397_1-p568133
103,3
Bn-scaff_16397_1-p360292
104,5
Bn-scaff_16397_1-p32614
105,2
Bn-scaff_25466_1-p237292
107,4
Bn-scaff_25466_1-p141048
108,5
Bn-scaff_20294_1-p378164
111,5
Bn-scaff_20294_1-p303417
113,0
Bn-scaff_20294_1-p250512
Bn-scaff_20294_1-p177085
115,2
Bn-scaff_20947_1-p300800
115,7
Bn-scaff_17799_1-p2391248
117,2
Bn-scaff_17799_1-p2273825
117,7
Bn-scaff_17799_1-p2111711
119,6
Bn-scaff_17799_1-p2065343
Bn-scaff_17799_1-p1947398
121,7
94,3
Bn-scaff_23957_1-p250533
Bn-scaff_23957_1-p280738
Bn-scaff_23957_1-p419108
Bn-scaff_23957_1-p488858
Bn-scaff_16874_1-p173312
Bn-scaff_16874_1-p325632
95,2
Bn-scaff_16397_1-p625792
100,7
Bn-scaff_16397_1-p626774
Bn-scaff_16397_1-p626437
103,2
Bn-scaff_16397_1-p514936
147,1
Bn-scaff_16397_1-p360292
Bn-scaff_20294_1-p250512
103,9
148,4
Bn-scaff_16397_1-p102356
Bn-scaff_20294_1-p235008
Bn-scaff_20294_1-p303417
Bn-scaff_16397_1-p32614
149,3
Bn-scaff_20294_1-p236211
Bn-scaff_25466_1-p237292
149,7
104,5
Bn-scaff_17799_1-p2273825
Bn-scaff_25466_1-p141048
154,6
Bn-scaff_17799_1-p2111711
Bn-scaff_25466_1-p33838
155,5
Bn-scaff_17799_1-p1495234
Bn-scaff_25466_1-p15362
158,9
Bn-scaff_17799_1-p1497402
Bn-scaff_20294_1-p443494
159,3
105,1
Bn-scaff_17799_1-p1489077
Bn-scaff_20294_1-p303417
160,2
106,5
Bn-scaff_17799_1-p1435332
Bn-scaff_20294_1-p236211
160,7
107,9
Bn-scaff_17799_1-p1428594
Bn-scaff_20294_1-p177085
161,1
109,2
Bn-scaff_17799_1-p1367834
Bn-scaff_17799_1-p2467176
161,6
110,5
Bn-scaff_17799_1-p1214222
Bn-scaff_17799_1-p2391248
164,6
112,5
Bn-scaff_15712_10-p423080
Bn-scaff_17799_1-p2273825
168,1
Bn-scaff_17799_1-p300944
172,2
Bn-scaff_17799_1-p2216531
Bn-scaff_24104_1-p13971
Bn-scaff_17799_1-p2111711
179,5
115,0
Bn-scaff_17799_1-p2065343
Bn-scaff_17799_1-p1947398
Bn-scaff_17799_1-p1629892
118,8
Bn-scaff_17799_1-p1658965
Bn-scaff_17799_1-p1495234
120,7
Bn-scaff_17799_1-p1489077
Bn-scaff_17799_1-p1435332
122,0
Bn-scaff_17799_1-p1331277
124,1
Bn-scaff_17799_1-p1082380
125,5
Bn-scaff_17799_1-p1629892
125,0
Bn-scaff_17799_1-p884874
Bn-scaff_17799_1-p1658965
126,9
Bn-scaff_17799_1-p859838
Bn-scaff_17799_1-p1495234
128,3
Bn-scaff_17799_1-p841812
127,5
Bn-scaff_17799_1-p1489077
Bn-scaff_17799_1-p1428594
129,2
Bn-scaff_17799_1-p1399131
129,6
Bn-scaff_17799_1-p1331277
130,5
Bn-scaff_17799_1-p1214222
131,4
Bn-scaff_17799_1-p1180982
132,4
Bn-scaff_17799_1-p1165291
133,8
Bn-scaff_17799_1-p1082380
134,3
Bn-scaff_17799_1-p1070542
135,1
Bn-scaff_17799_1-p905343
137,3
Bn-scaff_17799_1-p884874
137,7
Bn-scaff_17799_1-p859838
Bn-scaff_17799_1-p836919
138,2
Bn-scaff_17799_1-p717821
139,3
Bn-scaff_17799_1-p300944
142,0
Bn-scaff_24104_1-p173692
147,0
Bn-scaff_21018_1-p86419
Ex*1012-98
C06
Ex*V8
C06
Ex*R53
C06
C06162207
0,0
Cnnr61642607
0,8
C062542286
C062590057
2,1
C062612430
C062652319
3,3
C062652395
C062660714
4,5
C062817759
5,6
C062866733
7,9
C062867884
C062875455
9,3
C06r204533
10,3
C06r188410
11,2
C063036254
12,3
C06r291203
12,8
C063674884
16,9
C063756085
18,2
C063756318
C064151995
20,7
C064195735
C064365323
22,7
C018262506
23,1
C064741967
23,5
C064974377
25,2
C065003113
26,8
C065179168
27,2
C072242935het
27,6
C065243259
30,0
C065261843
30,7
C065669141
31,3
C06r515712
C06r575846
33,8
Cnnr67272988
C066090973
36,3
C066188849
37,1
C066200311
C066221578
38,6
C0611594894
44,7
C0612445070
45,2
C0612458518
C0612826412
48,1
C0613125553
48,7
C0613136743
49,3
C0613524561
C09r1187206
C09r1189999
51,8
Cnnr40809009
C0616553561
53,4
C0617543890
56,7
C0617982268
57,1
C0618046506
58,8
C0618257867
59,6
C0618701902
60,0
C0619894887
63,1
C0620218234
63,5
C06r2420531
65,4
C061072671
0,0
C06309133
0,7
C062866733
5,1
C062867884
C062875455
C062983467
7,6
C06r188410
C06r204533
C063160876
C063164449
9,5
C06r291203hetEx
C063496683
12,4
C063594237
13,1
C063674884
13,8
C0115388644PA
C063756085
14,4
C063756318
C064151995
15,7
C064195735
C064228857PA
20,1
C064365323
24,2
C065003113
29,1
C065179168
C065870070
30,5
C065909549
31,3
Cnnr67272988
32,1
C06r515712
35,3
C06r575846
C065956410
37,2
C066188849
C066200311
37,8
C066221578
C066303660
38,5
C067267212
39,9
A0915745353het
42,1
C0611594894
45,0
C0612445070
47,1
C0612458518
C0612826412
48,4
C0613524561
51,7
Cnnr40809009
54,3
C09r1187206
54,9
C09r1189999
55,5
C0621936594
71,6
C0621008977
74,1
C0623278545
C0624587541
76,6
C0624589881
C0625255708
77,5
C0625271464
C0625304796
79,1
C0625446919
C06r2717930
C0625596671
84,2
C0625671215
C0627095038
90,0
C0627032196
90,9
C0628471888
92,5
C06r2952833
C0629172287
93,4
C0629401522
C0629489446
C0629525474
C0629530478
C0630013625
C0630109405
C0630243825
C0630304436
C06843869
0,0
C061072671
0,4
C06r119068
C06309133
2,8
C062542286
4,3
C062590057
5,9
C062651660PA
6,6
C062652395
C062612430
6,8
C062652319
7,1
C062714348
8,9
C063160876
C063164449
21,9
C06r291203
C063555794
25,5
C063674884
32,4
C0612445070
52,8
C0612458518
C0612826412
54,0
C0613524561
56,3
C09r1187206
C09r1189999
63,9
Cnnr30939448
Cnnr40809009
C0615199533
67,9
C0615576918
68,3
C0615656961
68,8
C0615753406
69,4
Cnnr50093002
70,5
A0716334853
91,3
C0621008977
94,6
C0624587541
95,3
C0625271464
96,1
C0624589881
96,8
C0623278545
97,7
C0621936594
99,0
C0622623963
102,3
C0621666747
103,8
C0622614285
105,3
C0622635632
105,8
C0621049946
106,4
Cnnr33147421
106,9
C0624703167
107,4
C0625623248
108,4
C0625424622
109,0
C0626473982
112,7
C0628471888
117,5
C0629489446
C0629525474
121,4
C0629530478
C0632407522
129,6
C0633221083
136,5
C0633209209
137,6
C0633213711
140,8
C0633255327
141,6
C0633566786
C0633672057
C06r2597890
66,8
C0621936594
67,4
C0621008977
C0622614285
C0622623963
69,1
C0623278545
C0624587541
C0624589881
C0625088132
69,9
C0625271464
70,7
C06r2717930
C0625446919
71,8
C0625596671
74,0
C0625671215
Cnnr7086052
76,4
Cnnr16038299
77,6
C0626260307
78,3
C0626473982
C0627032196
79,3
C0627095038
C0627770628
79,8
C0627841889
80,2
C0627985350
81,2
C06r2952833
82,1
C0629401522
85,9
C0629489446
86,3
C0629525474
86,7
C0629530478
C0630013625
89,2
C0630109405
C0630243825
90,2
C0630304436
91,2
C0630781503
93,2
C0630819742
93,6
C0631278885
94,0
C0631348799
95,9
C0631536166
96,6
C0631695303
C0631831436
98,3
C0632026741
98,9
C0632036971
99,4
C0632255286
101,6
C0632296267
102,3
C0632296605
C0632344523
103,3
C0632556401
104,5
C0632873886
105,2
C0632950906
107,4
C0633064876
108,5
C0633582659
111,5
C0633642697
113,0
C06r3244625
C0633749057
115,2
C06r3305591
115,7
C0634110507
117,2
C0634271977
117,7
C0634426218
119,6
C0634498584
C0634617964
121,7
94,3
C0630781503
C0630819742
C0631278885
C0631348799
C0631536166
C0631695303
95,2
C0632297250PA
100,7
C0632296267
C0632296605
103,2
C0632407522
C0632556401
103,9
147,1
C0632807261
C06r3244625
C0632873886
148,4
C06r3262460
C0632950906
C0633642697
149,3
104,5
C0633064876
C06r3261257
149,7
C0633209209
C0634271977
154,6
C0633221083
C0634426218
155,5
C0633520258
105,1
C0635063865
158,9
C0633642697
106,5
C0635061697
159,3
C06r3261257
107,9
C0635069536
160,2
C0633749057
109,2
C0635120848
160,7
C0634039384
110,5
C0635129017
161,1
C0634110507
C0635195606
161,6
112,5
C0634271977
C0635387965
164,6
C0634331757
C055151789
168,1
C0634426218
115,0
C0636342566
172,2
C0634498584
C0637064141
179,5
C0634617964
C0634936075
118,8
Cnnr75691851
C0635063865
120,7
C0635069536
C0635120848
122,0
C0635234587
124,1
C0635520329
125,5
C0635706988
C0634936075
126,9
C0635732420
125,0
Cnnr75691851
C0635750569
127,5
C0635063865
128,3
C0635069536
C0635129017
129,2
C0635159221
129,6
C0635234587
130,5
C0635387965
131,4
C0635423067
132,4
C0635444783
133,8
C0635520329
134,3
C0635526012
135,1
C0635686276
137,3
C0635706988
137,7
C0635732420
C0635755486
138,2
C0635895670
139,3
C0636342566
142,0
C0636896574
147,0
C0637190888

## Slide 17
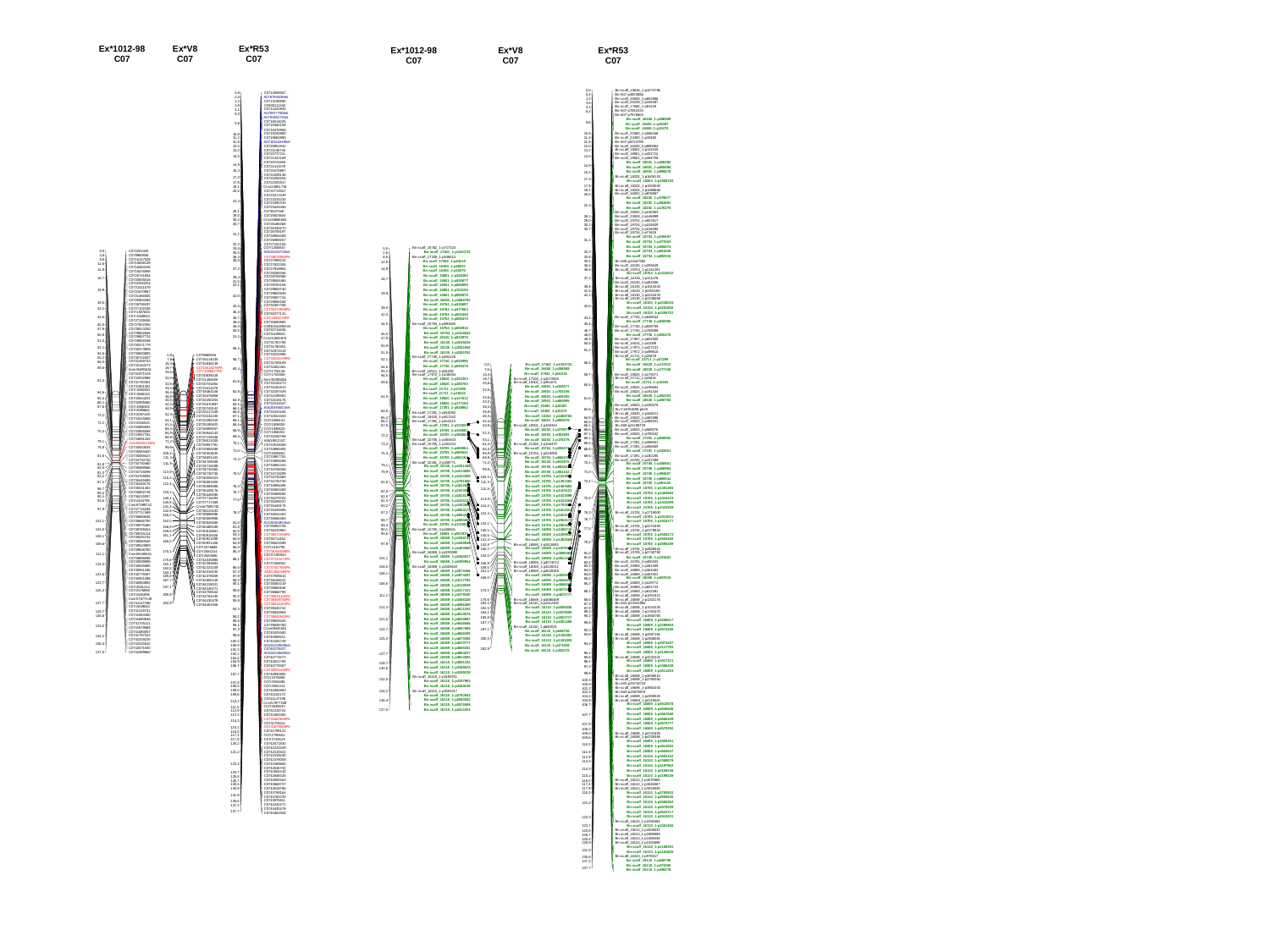

Ex*1012-98
C07
Ex*V8
C07
Ex*R53
C07
C0712808567
0,0
A076794508het
0,4
C0713199890
1,2
C0940111345
3,6
C0714220935
4,1
A078977790het
8,3
A079195273het
C0718214035
9,6
C0718464106
C0718476958
C0719155880
10,8
C0719662980
11,2
A0710152249het
11,6
C0720651942
12,0
C0721145726
13,2
C0720737211
14,5
C0721322169
C0720741654
14,9
C0721411579
C0721670987
15,3
C0722209136
17,3
C0722250254
C0722325917
17,8
Cnnr24951718
19,1
C0722713507
20,5
C0723217549
C0723234230
22,3
C0723395310
C0723424456
C073647048
28,1
C0725823604
29,0
Cnnr40886563
30,2
C0726186368
30,7
C0726335570
C0726708197
31,1
C0726904368
C0726989257
C0727102106
32,2
C07r1368910
33,8
A0623155725het
35,5
C0728076990PA
36,4
C0727589234
36,8
C0727822355
C0727919904
37,2
C0728268346
C0728755088
39,4
C0729504485
41,5
C0729783108
42,1
C0729800740
C0729802848
42,6
C0729807724
C0729858188
C0730357798
43,4
C0730374909PA
45,4
C0730377133
C07r1686276PA
46,7
C0730600885
48,0
C0932542691het
49,3
C0730716636
50,5
C0731109501
51,2
Cnnr31600874
C0731782789
C0731790301
C072201425
0,0
C079960936
2,6
C0715147528
8,8
C0715836139
12,6
C0718464106
14,9
C0718476958
C0720741654
18,7
C0720835018
C0722250254
C0721411579
19,9
C0721670987
C0721456505
C0726904368
39,0
C0726708197
C0727102106
42,3
C07r1367646
C07r1368910
44,0
C0727139436
C0727822355
45,5
C0728513250
47,9
C0729802848
50,9
C0729807724
C0729858188
51,5
C0730371779
52,1
C0730374909
C0730600885
54,6
C0730732407
55,2
C0731459743
56,5
C0732140273
59,6
Annr35495818
C0731971519
C0732032996
61,5
C0731790301
C0731801361
C07r1893260
64,6
C07r1868143
C0732644201
65,4
C0732909460
66,1
C07r1966302
67,6
C07r2085662
C07r2097149
72,2
C0733515068
C07r2055515
73,2
C0733890093
C0733958288
75,3
C0733957781
C0734691443
79,1
C0225920134PA
C0734553635
79,9
C0735083400
C0735058423
81,6
C0734730730
C0734730460
81,8
C0735089586
82,0
C0734719299
82,3
C0734708058
83,2
C0735449495
87,2
C0735449176
C0735531363
88,7
C0735902738
89,4
C0736219357
90,1
C07r2410795
93,6
Cnnr67599742
C0737716294
97,9
C0737717489
56,2
C0731971519
C0732032996
C0732035199PA
58,7
C0731789169
C0731801361
60,4
C07r1756126
C07r1763366
Annr35495818
61,6
C0732140273
C0732181813
C0732187449
62,8
C0732199381
C0732218175
64,9
C0732314547
65,5
A0620494551het
66,1
C0732423168
66,6
C0732551503
67,1
C07r1868143
68,1
C07r1908050
68,5
C07r1946525
68,9
C07r1966302
C0733190768
69,5
A0619512437
70,1
C0733515068
C0733890093
71,0
C07r2085662
C0733957781
73,4
C0733958288
C0734691443
C0734708058
C0734719299
75,5
C0734730460
C0734730730
C0734994495
76,3
C0735083400
76,7
C0735089586
C0735279702
77,5
C0735285037
C0735449176
C0735449495
79,2
C0735531363
C0735566360
A0320381953het
81,0
C0735902738
81,8
C0736232962
82,5
C0736573356PA
83,3
C0736711452
84,0
C0736821599
84,8
C07r2410795
85,0
C0736765598PA
85,3
C0737195853
C0737210474PA
86,1
C0737458061
C0737647555PA
86,5
A0321955449PA
87,3
C0737939610
87,9
C0738188531
89,2
C0738363219
90,1
C0738660636
90,6
C0738666790
C0739015114PA
92,2
C0739369786PA
93,8
C0739416453PA
C0739445742
94,3
C0739543959
C079960936
0,0
C0718214035
7,6
C0715836139
15,4
C0715618276PA
18,7
C07r1086617PA
25,6
C0720835018
32,0
C0721456505
C0720741654
32,6
C0721411579
33,2
C0718464106
36,4
C0718476958
36,9
C0722250254
40,3
C0721670987
42,4
C0720749142
44,9
C0723217549
52,3
C0723234230
C0723395310
54,1
C0725185822
61,4
C0726989257
65,4
C0726944143
65,9
C0727139436
66,8
C0728513250
71,2
C0733957781
99,9
C0733958288
C0734553635
105,4
C0734691443
111,3
C0734708058
111,8
C0734719299
C0734730460
113,9
C0734730730
C0735058423
116,2
C0735083400
122,4
C0735089586
C0735449176
133,1
C0735449495
C0737716294
140,1
C0737717489
140,6
Cnnr67599742
141,4
C0738223183
142,8
C0739898096
150,2
C0740009998
152,0
C0740025580
C0740480189
156,9
C0740616861
159,5
C0740918426
161,1
C0740921098
165,0
C0740931436
C07r2576888
C07r2581314
170,1
C07r2581696
C0741035980
175,6
C0742283684
182,1
C0742223249
182,5
C0742319230
184,1
C0742378059
185,8
C0742605418
187,7
C0738660636
C0738666790
101,1
C0738575465
C0738789453
104,5
C0739015114
106,1
C0739445742
C0739560540
108,6
C0739543959
C0739838780
Cnnr69445501
111,1
C0739898096
C0740009998
114,3
C0740025580
C0740931436
C0740779387
121,5
C0740921098
C0740950892
122,7
C0744239021
C07r2581314
197,1
C0744240271
C07r2576888
125,2
C0743759164
C07r2581696
200,5
C0743764235
Cnnr57877249
C0744430478
C0741147198
202,9
127,7
C0744461558
C07r2649043
C0741318741
128,7
C0741563450
130,6
C0739560540PA
95,1
C0741680846
C0739659425
95,6
C0741705111
C0739838780
96,1
132,0
C0741679569
Cnnr69445501
97,3
C0741680057
C0740025580
99,6
C0741787222
134,2
C0740069211
C0742319230
C0740184749
100,3
C0742315532
136,4
A0324103509het
100,9
C0742071830
C0740279437
101,3
C0742489660
137,0
A0324244585het
102,2
C0740773573
104,2
C0740521785
104,9
C0740779387
106,7
C0740931436PA
C0740950892
107,7
C07r2576888
C07r2581696
107,9
C07r2581314
108,3
C0741084983
109,0
C0741102172
109,6
C0741147198
110,2
Cnnr57877249
C07r2649043
111,5
C0741318741
112,9
C0741563450
113,3
C0741680846PA
114,3
C0741705111
C0741679569PA
115,1
C0741799122
116,5
C07r2780652
117,4
C07r2749119
117,9
C0742071830
120,2
C0742223249
C0742315532
121,2
C0742319230
C0742378059
C0742489660
123,3
C0742536740
C0742605418
123,7
C0742848328
125,8
C0742892563
126,7
C0743568737
130,4
C0743618766
130,9
C0743759164
131,9
C0743764235
C0743976811
135,6
C0744240271
137,2
C0744430478
137,7
C0744461558
Ex*1012-98
C07
Ex*V8
C07
Ex*R53
C07
Bn-scaff_15626_1-p1073796
0,0
Bn-A07-p4923886
0,4
Bn-scaff_15626_1-p651986
1,2
Bn-scaff_26139_1-p106387
3,6
Bn-scaff_17580_1-p83128
4,1
Bn-A07-p7082333
8,3
Bn-A07-p7578604
Bn-scaff_16450_1-p286989
9,6
Bn-scaff_16450_1-p28287
Bn-scaff_16450_1-p15270
Bn-scaff_23400_1-p556346
10,8
Bn-scaff_23400_1-p35185
11,2
Bn-A07-p8724769
11,6
Bn-scaff_16100_1-p890064
12,0
Bn-scaff_18501_1-p114150
13,2
Bn-scaff_18501_1-p421712
14,5
Bn-scaff_18501_1-p264708
Bn-scaff_18501_1-p426392
14,9
Bn-scaff_18501_1-p665995
Bn-scaff_18501_1-p995878
15,3
Bn-scaff_18202_1-p1606133
17,3
Bn-scaff_18202_1-p1568783
Bn-scaff_18202_1-p1503040
17,8
Bn-scaff_18202_1-p1088668
19,1
Bn-scaff_18202_1-p976987
20,5
Bn-scaff_18202_1-p379677
Bn-scaff_18202_1-p363891
22,3
Bn-scaff_18202_1-p176275
Bn-scaff_18202_1-p136363
Bn-scaff_23839_1-p148899
28,1
Bn-scaff_19724_1-p557617
29,0
Bn-scaff_19724_1-p314829
30,2
Bn-scaff_19724_1-p216395
30,7
Bn-scaff_19724_1-p71618
Bn-scaff_15754_1-p245697
31,1
Bn-scaff_15754_1-p477663
Bn-scaff_15754_1-p555074
Bn-scaff_15754_1-p651840
32,2
Bn-scaff_15754_1-p692916
33,8
Bn-A06-p24167592
35,5
Bn-scaff_16130_1-p293649
36,4
Bn-scaff_15754_1-p1141201
36,8
Bn-scaff_15754_1-p1416542
37,2
Bn-scaff_16130_1-p111476
Bn-scaff_16130_1-p461566
Bn-scaff_16130_1-p1013445
39,4
Bn-scaff_16130_1-p1901165
41,5
Bn-scaff_16130_1-p2224476
42,1
Bn-scaff_16130_1-p2246088
Bn-scaff_16130_1-p2248228
42,6
Bn-scaff_16130_1-p2251656
Bn-scaff_16130_1-p2305762
Bn-scaff_17740_1-p848044
43,4
Bn-scaff_17740_1-p830996
45,4
Bn-scaff_17740_1-p828759
Bn-scaff_17740_1-p758099
46,7
Bn-scaff_17740_1-p555478
48,0
Bn-scaff_17367_1-p601905
49,3
Bn-scaff_16514_1-p41089
50,5
Bn-scaff_17972_1-p427131
51,2
Bn-scaff_17972_1-p499610
Bn-scaff_21711_1-p39478
Bn-scaff_21711_1-p27299
Bn-scaff_15762_1-p717223
0,0
Bn-scaff_17461_1-p1193733
2,6
Bn-scaff_17326_1-p536514
8,8
Bn-scaff_17942_1-p64218
12,6
Bn-scaff_16450_1-p28287
14,9
Bn-scaff_16450_1-p15270
Bn-scaff_18501_1-p426392
18,7
Bn-scaff_18501_1-p530677
Bn-scaff_18501_1-p665995
Bn-scaff_18501_1-p703155
19,9
Bn-scaff_18501_1-p995878
Bn-scaff_18202_1-p1568783
Bn-scaff_15754_1-p245697
39,0
Bn-scaff_15754_1-p477663
Bn-scaff_15754_1-p651840
42,3
Bn-scaff_15754_1-p688470
Bn-scaff_15754_1-p691665
44,0
Bn-scaff_15754_1-p692916
Bn-scaff_15754_1-p1416542
45,5
Bn-scaff_16130_1-p810976
47,9
Bn-scaff_16130_1-p2248228
50,9
Bn-scaff_16130_1-p2251656
Bn-scaff_16130_1-p2305762
51,5
Bn-scaff_17740_1-p834125
52,1
Bn-scaff_17740_1-p830996
Bn-scaff_17740_1-p555478
54,6
Bn-scaff_16514_1-p56208
55,2
Bn-scaff_17972_1-p148284
56,5
Bn-scaff_18520_1-p252353
59,6
Bn-scaff_18520_1-p280760
Bn-scaff_21711_1-p27299
Bn-scaff_21711_1-p18343
61,5
Bn-scaff_18520_1-p117612
Bn-scaff_18520_1-p177168
Bn-scaff_17291_1-p838952
64,6
Bn-scaff_17291_1-p815082
Bn-scaff_18520_1-p817342
65,4
Bn-scaff_17291_1-p614212
66,1
Bn-scaff_17291_1-p333551
67,6
Bn-scaff_15705_1-p108561
Bn-scaff_15705_1-p394867
72,2
Bn-scaff_15705_1-p383403
Bn-scaff_15705_1-p432224
73,2
Bn-scaff_15705_1-p480964
Bn-scaff_15705_1-p560641
75,3
Bn-scaff_15705_1-p561162
Bn-scaff_22451_2-p438771
79,1
Bn-scaff_15705_1-p1351365
Bn-scaff_15705_1-p1214955
79,9
Bn-scaff_15705_1-p1433369
Bn-scaff_15705_1-p1791890
81,6
Bn-scaff_15705_1-p1812021
Bn-scaff_15705_1-p1433099
81,8
Bn-scaff_15705_1-p1818177
82,0
Bn-scaff_15705_1-p1415123
82,3
Bn-scaff_15705_1-p1403883
83,2
Bn-scaff_15705_1-p2504173
87,2
Bn-scaff_15705_1-p2504492
Bn-scaff_15705_1-p2590289
88,7
Bn-scaff_15705_3-p131881
89,4
Bn-scaff_15705_3-p436841
90,1
Bn-scaff_16069_1-p557815
93,6
Bn-scaff_16069_1-p1268741
Bn-scaff_16069_1-p1269936
97,9
Bn-scaff_16069_1-p1483568
56,2
Bn-scaff_18520_1-p117612
Bn-scaff_18520_1-p177168
Bn-scaff_18520_1-p179371
58,7
Bn-scaff_21711_1-p28431
Bn-scaff_21711_1-p18343
60,4
Bn-scaff_18520_1-p198684
Bn-scaff_18520_1-p191444
Bn-scaff_18520_1-p252353
61,6
Bn-scaff_18520_1-p280760
Bn-scaff_18520_1-p330575
Bn-C14051598-p519
62,8
Bn-scaff_18520_1-p348211
Bn-scaff_18520_1-p363396
64,9
Bn-scaff_18520_1-p486331
65,5
Bn-A06-p21196739
66,1
Bn-scaff_18520_1-p569076
66,6
Bn-scaff_18520_1-p705102
67,1
Bn-scaff_17291_1-p838952
68,1
Bn-scaff_17291_1-p398501
68,5
Bn-scaff_17291_1-p358565
68,9
Bn-scaff_17291_1-p333551
Bn-scaff_17291_1-p192195
69,5
Bn-scaff_15705_1-p157289
70,1
Bn-scaff_15705_1-p108561
Bn-scaff_15705_1-p480964
71,0
Bn-scaff_15705_1-p394867
Bn-scaff_15705_1-p560641
73,4
Bn-scaff_15705_1-p561162
Bn-scaff_15705_1-p1351365
Bn-scaff_15705_1-p1403883
Bn-scaff_15705_1-p1415123
75,5
Bn-scaff_15705_1-p1433099
Bn-scaff_15705_1-p1433369
Bn-scaff_15705_1-p1718000
76,3
Bn-scaff_15705_1-p1812021
76,7
Bn-scaff_15705_1-p1818177
Bn-scaff_15705_1-p2274493
77,5
Bn-scaff_15705_1-p2279820
Bn-scaff_15705_1-p2504173
Bn-scaff_15705_1-p2504492
79,2
Bn-scaff_15705_1-p2590289
Bn-scaff_15705_1-p2626544
Bn-scaff_15705_1-p2740790
81,0
Bn-scaff_15705_3-p131881
81,8
Bn-scaff_15705_3-p450433
82,5
Bn-scaff_16069_1-p261409
83,3
Bn-scaff_16069_1-p361062
84,0
Bn-scaff_16069_1-p481921
84,8
Bn-scaff_16069_1-p557815
85,0
Bn-scaff_16069_1-p419772
85,3
Bn-scaff_16069_1-p801743
Bn-scaff_16069_1-p812381
86,1
Bn-scaff_16069_1-p1051812
Bn-scaff_16069_1-p1202176
86,5
Bn-A03-p23181984
87,3
Bn-scaff_16069_1-p1531535
87,9
Bn-scaff_16069_1-p1781070
89,2
Bn-scaff_16069_1-p1946765
90,1
Bn-scaff_16069_1-p2288017
90,6
Bn-scaff_16069_1-p2295954
Bn-scaff_16069_1-p2574446
92,2
Bn-scaff_16069_1-p2897181
93,8
Bn-scaff_16069_1-p2946061
Bn-scaff_16069_1-p2974287
94,3
Bn-scaff_16069_1-p3117793
Bn-scaff_17461_1-p1193733
0,0
Bn-scaff_16450_1-p286989
7,6
Bn-scaff_17942_1-p64218
15,4
Bn-scaff_17326_1-p1072830
18,7
Bn-scaff_18501_1-p951071
25,6
Bn-scaff_18501_1-p530677
32,0
Bn-scaff_18501_1-p703155
Bn-scaff_18501_1-p426392
32,6
Bn-scaff_18501_1-p665995
33,2
Bn-scaff_16450_1-p28287
36,4
Bn-scaff_16450_1-p15270
36,9
Bn-scaff_18202_1-p1568783
40,3
Bn-scaff_18501_1-p995878
42,4
Bn-scaff_18501_1-p435344
44,9
Bn-scaff_18202_1-p379677
52,3
Bn-scaff_18202_1-p363891
Bn-scaff_18202_1-p176275
54,1
Bn-scaff_21268_1-p106470
61,4
Bn-scaff_15754_1-p555074
65,4
Bn-scaff_15754_1-p519288
65,9
Bn-scaff_15754_1-p688470
66,8
Bn-scaff_16130_1-p810976
71,2
Bn-scaff_15705_1-p560641
99,9
Bn-scaff_15705_1-p561162
Bn-scaff_15705_1-p1214955
105,4
Bn-scaff_15705_1-p1351365
111,3
Bn-scaff_15705_1-p1403883
111,8
Bn-scaff_15705_1-p1415123
Bn-scaff_15705_1-p1433099
113,9
Bn-scaff_15705_1-p1433369
Bn-scaff_15705_1-p1791890
116,2
Bn-scaff_15705_1-p1812021
122,4
Bn-scaff_15705_1-p1818177
Bn-scaff_15705_1-p2504173
133,1
Bn-scaff_15705_1-p2504492
Bn-scaff_16069_1-p1268741
140,1
Bn-scaff_16069_1-p1269936
140,6
Bn-scaff_16069_1-p1483568
141,4
Bn-scaff_16069_1-p1815891
142,8
Bn-scaff_16069_1-p3479340
150,2
Bn-scaff_16069_1-p3596469
152,0
Bn-scaff_16069_1-p3611263
Bn-scaff_16069_1-p4074074
156,9
Bn-scaff_16069_1-p4228211
159,5
Bn-scaff_16069_1-p4528345
161,1
Bn-scaff_16069_1-p4530987
165,0
Bn-scaff_16069_1-p4546846
Bn-scaff_16069_1-p4666209
Bn-scaff_16069_1-p4670392
170,1
Bn-scaff_16069_1-p4670777
Bn-scaff_16069_1-p4686169
175,6
Bn-scaff_16110_1-p2612205
182,1
Bn-scaff_16110_1-p2655836
182,5
Bn-scaff_16110_1-p2576509
184,1
Bn-scaff_16110_1-p2522717
185,8
Bn-scaff_16110_1-p2261356
187,7
Bn-scaff_16069_1-p2205590
Bn-scaff_16069_1-p2288017
101,1
Bn-scaff_16069_1-p2295954
Bn-scaff_16069_1-p2408540
104,5
Bn-scaff_16069_1-p2574446
106,1
Bn-scaff_16069_1-p2974287
Bn-scaff_16069_1-p3117793
108,6
Bn-scaff_16069_1-p3138539
Bn-scaff_16069_1-p3417121
Bn-scaff_16069_1-p3479340
111,1
Bn-scaff_16069_1-p3456338
Bn-scaff_16069_1-p3596469
114,3
Bn-scaff_16069_1-p3611263
Bn-scaff_16069_1-p4510578
Bn-scaff_16069_1-p4530987
121,5
Bn-scaff_16069_1-p4546846
Bn-scaff_16110_1-p650015
Bn-scaff_16069_1-p4567688
122,7
197,1
Bn-scaff_16110_1-p648756
Bn-scaff_16069_1-p4666209
Bn-scaff_16110_1-p1185491
Bn-scaff_16069_1-p4670392
200,5
125,2
Bn-scaff_16110_1-p1181825
Bn-scaff_16069_1-p4670777
Bn-scaff_16110_1-p471808
Bn-scaff_16069_1-p4805351
202,9
Bn-scaff_16110_1-p436278
Bn-scaff_16069_1-p3138539
Bn-scaff_16069_1-p4854537
95,1
127,7
Bn-scaff_16069_1-p3232221
95,6
Bn-scaff_16069_1-p4814083
Bn-scaff_16069_1-p3417121
96,1
Bn-scaff_16110_1-p3591152
128,7
Bn-scaff_16069_1-p3456338
97,3
Bn-scaff_16110_1-p3368578
130,6
Bn-scaff_16069_1-p3611263
Bn-scaff_16110_1-p3199239
99,6
Bn-scaff_16069_1-p3638510
Bn-scaff_16110_1-p3198751
132,0
Bn-scaff_16069_1-p3780494
100,3
Bn-scaff_16110_1-p3197962
Bn-A03-p25718716
100,9
Bn-scaff_16110_1-p3168236
Bn-scaff_16069_1-p3861034
101,3
Bn-scaff_16110_1-p3092217
134,2
Bn-A03-p25879876
102,2
Bn-scaff_16110_1-p2793943
Bn-scaff_16069_1-p4359025
104,2
Bn-scaff_16110_1-p2580362
136,4
Bn-scaff_16069_1-p4119525
104,9
Bn-scaff_16069_1-p4510578
Bn-scaff_16110_1-p2576509
106,7
Bn-scaff_16069_1-p4546846
Bn-scaff_16110_1-p2412201
137,0
Bn-scaff_16069_1-p4567688
107,7
Bn-scaff_16069_1-p4666209
Bn-scaff_16069_1-p4670777
107,9
Bn-scaff_16069_1-p4670392
108,3
Bn-scaff_16069_1-p4741693
109,0
Bn-scaff_16069_1-p4759395
109,6
Bn-scaff_16069_1-p4805351
110,2
Bn-scaff_16069_1-p4814083
Bn-scaff_16069_1-p4854537
111,5
Bn-scaff_16110_1-p3591152
112,9
Bn-scaff_16110_1-p3368578
113,3
Bn-scaff_16110_1-p3197962
114,3
Bn-scaff_16110_1-p3168236
Bn-scaff_16110_1-p3199239
115,1
Bn-scaff_16110_1-p3075966
116,5
Bn-scaff_16110_1-p3046587
117,4
Bn-scaff_16110_1-p3019038
117,9
Bn-scaff_16110_1-p2793943
120,2
Bn-scaff_16110_1-p2655836
Bn-scaff_16110_1-p2580362
121,2
Bn-scaff_16110_1-p2576509
Bn-scaff_16110_1-p2522717
Bn-scaff_16110_1-p2412201
123,3
Bn-scaff_16110_1-p2336384
Bn-scaff_16110_1-p2261356
123,7
Bn-scaff_16110_1-p2028032
125,8
Bn-scaff_16110_1-p1986889
126,7
Bn-scaff_16110_1-p1388436
130,4
Bn-scaff_16110_1-p1330898
130,9
Bn-scaff_16110_1-p1185491
131,9
Bn-scaff_16110_1-p1181825
Bn-scaff_16110_1-p976517
135,6
Bn-scaff_16110_1-p648756
137,2
Bn-scaff_16110_1-p471808
137,7
Bn-scaff_16110_1-p436278

## Slide 18
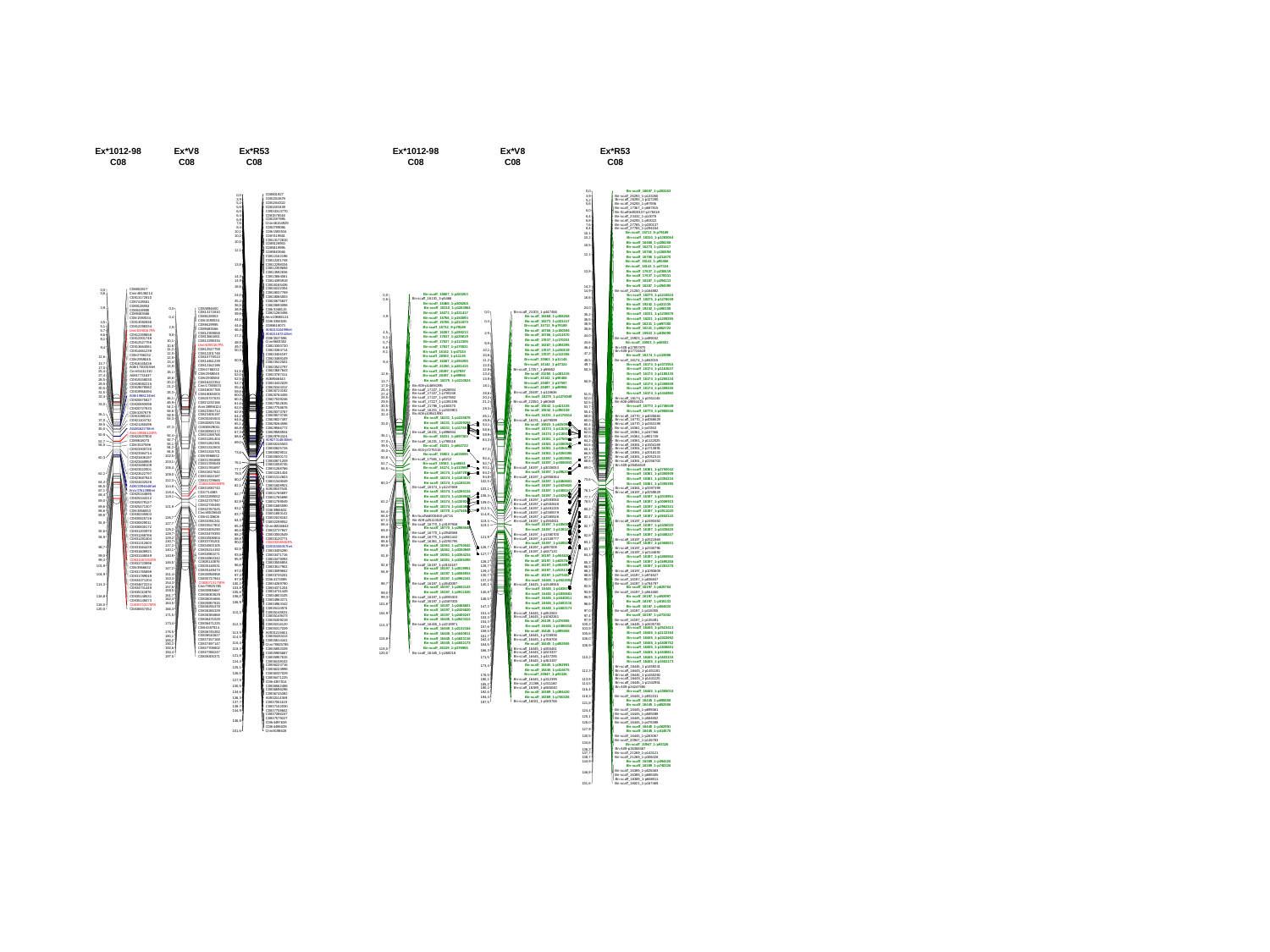

Ex*1012-98
C08
Ex*V8
C08
Ex*R53
C08
C08801927
0,0
C081353878
3,9
C081364322
5,2
C081383439
5,6
C0933313770
6,0
C081576516
6,4
C081387985
6,8
Cnnr46154929
7,6
C082799086
8,4
C08r1593534
10,1
C087419561
10,2
C0813172810
10,5
C089128953
C089419995
12,1
C089483566
C0812162196
C0812201748
C0812298334
13,8
C0812359658
C0813592836
C0813664061
14,3
C0814395919
14,9
C0816165436
16,6
C0816222354
C0818037769
24,0
C0818065003
C0820675827
35,2
C0820693098
36,5
C08r3268115
38,9
C0821260496
39,8
Annr19886124
44,0
C08r3358535
C088618073
44,6
A0815310499het
46,4
A0815167242het
47,2
C08r3537596
Cnnr8603302
48,5
C0823300720
49,7
C0823384714
50,3
C0823406197
C0823488109
50,9
C0823522501
C0823522797
C0823687843
51,5
C0823797411
52,0
A068566443
52,5
C0824432028
53,7
C0825344312
55,4
C0826723192
59,6
C0826764406
60,0
C0827039246
60,4
C0827552635
61,6
C0827758678
62,0
C0828373767
62,8
C0828573746
64,2
C0829027497
64,6
C0829264596
65,1
C0829566772
66,8
C08801927
0,0
Cnnr48138214
0,6
C0813172810
C087419561
C089128953
3,9
C089419995
C089483566
C08r1593534
C0813592836
4,5
C0812298334
5,1
Unnr6390167PA
5,7
C0812359658
6,6
C0812201748
8,1
C0812527759
C0813664061
9,4
C0814461239
C08r2766232
12,6
C08r2959045
C0816165436
13,7
A08r1730053het
17,0
Cnnr51111310
25,4
A0817731637
27,4
C0819158030
28,5
C0819592215
29,5
C0819678582
30,5
C0819966494
31,5
A08r1988134het
32,4
C0820675827
C0820693098
33,0
C0820727633
C08r3267679
36,1
C08r3268115
C0821104732
37,0
C0821260496
39,5
A048562276het
45,0
Annr19886124PA
50,8
C0822637800
C088618073
52,7
C08r3537596
56,5
C0823300720
C0823384714
C0823406197
60,3
C0823469959
C0823488109
C0823522501
C0823522797
62,2
C0823687843
C0824432028
64,4
A0923394648het
66,5
Annr3761398het
67,1
C0825154695
68,4
C0825344312
69,0
C0825379127
C0825471307
69,6
C08r3856553
88,6
C0830245503
89,8
C0830825726
C0830829011
91,8
C0830830172
C0831203970
92,6
C0831268766
94,9
C0831281404
C0831312603
C0831564439
98,7
C0831608921
C084094600
0,0
C0813172810
C089128953
0,4
C08r1593534
C089419995
2,9
C089483566
C0812359658
9,8
C0813664061
C0812298334
10,1
Unnr6390167PA
10,6
C0812527759
11,2
C0812201748
12,0
C0815778513
12,8
C0814461239
13,4
C0812162196
13,9
C08r2766232
18,1
C08r2959045
C08r2930580
18,6
C0816222354
20,2
Cnnr17365674
21,2
C0818037769
28,3
C0818065003
C0820727633
46,1
C0821232166
49,9
Annr19886124
54,1
C0823384714
58,6
C0823406197
59,9
C0830245503
83,3
C0830825726
C0830829011
87,3
C0830830172
C0831268766
92,4
C0831281404
92,7
C0831262381
93,1
C0831312603
94,2
C0831316701
94,8
C08r3966632
102,5
C0831765898
103,1
C0831789049
C0831765697
106,4
C0831617642
109,0
C0831624187
C0831729665
112,3
C0831830690PA
114,8
C0831830742
C02r714383
118,4
C0832299052
119,1
C0832737947
C0832738490
121,9
C0832767625
Cnnr46536643
C08r4133606
126,7
C0833391241
127,7
C0833517902
C0833405290
128,2
C0833479393
128,7
C0833506804
129,2
C0833735201
130,7
C0834803105
C0829598934
67,3
C0829794324
68,6
A0927314845het
69,0
C0830245503
C0830825726
C0830829011
73,6
C0830830172
C0830871209
76,1
C0831008745
C0831268766
77,7
C0831281404
78,5
C0831312603
80,2
C0831348049
C0831608921
82,1
A0928537545
C0831765697
82,7
C0831765898
C0831789049
82,8
C0831685590
83,1
C08r3966632
C0831883141
83,7
C0832028162
84,3
C0832299052
Cnnr46536643
85,3
C0832737947
86,5
C0833000549
88,2
C0833120771
88,8
C0833258545PA
90,0
C0833306357het
92,5
C0833405290
C0833471716
93,8
C0833479393
95,9
C0833506804
96,6
C0833517902
C0833599652
97,0
C0833735201
97,4
C08r4173089
97,9
C0834269780
100,2
C0834371204
103,8
C0834731449
105,6
C0834803105
106,0
C0834963271
106,9
C0834963342
C0835110876
C0835148531
110,2
C0835149473
C0835309219
C0835316120
112,3
C0835317039
A0931215661
113,9
C0835430413
114,5
C0835614441
116,4
Cnnr78925785
C0835853339
118,3
C0835905667
121,8
C0835957815
C0836108102
124,4
C0836223716
125,1
C0836224998
C0836337029
126,0
137,2
C0835214192
140,1
C0834963271
C0831348049
99,0
140,8
C0834963342
C0831367251PA
99,3
C0835110876
146,5
C0831723896
101,8
C0835148531
C08r3966632
147,2
C0835149473
C0831765898
104,9
C0835956958
151,4
C0831789049
C0835717944
153,3
C0834371204
C0835713175PA
154,3
C0834672224
114,3
Cnnr78925785
157,6
C0834731449
C0835905667
159,5
C0835110876
C0836063629
161,7
C0835148531
116,8
C0836034666
162,4
C0835149473
C0835957815
164,5
C0835713175PA
118,0
C0836255378
C0836557452
120,0
166,3
C0836265329
C0836384868
171,5
C0836471029
C0836471225
173,4
C08r4357914
C0836745382
176,5
C0836510627
180,1
C0837157168
185,2
C0837497147
190,2
C0837708602
192,6
C0837386247
194,4
C0838305371
197,5
C0836471225
127,9
C08r4357914
C0836562488
130,5
C0836696296
134,6
C0836745382
A0932514369
136,3
C0837001103
137,7
C0837152030
138,7
C0837708602
144,9
C0837386247
C0837579227
146,8
C08r4497639
C08r4498028
Cnnr8198628
151,6
Ex*1012-98
C08
Ex*V8
C08
Ex*R53
C08
Bn-scaff_18607_1-p283263
0,0
Bn-scaff_26293_1-p133260
3,9
Bn-scaff_26293_1-p117280
5,2
Bn-scaff_26293_1-p97936
5,6
Bn-scaff_17367_1-p687815
6,0
Bn-Scaffold000157-p178418
Bn-scaff_23432_1-p10078
6,4
Bn-scaff_26293_1-p92022
6,8
Bn-scaff_27765_1-p330137
7,6
Bn-scaff_27765_1-p294154
8,4
Bn-scaff_15712_9-p79189
10,1
Bn-scaff_18310_1-p1283064
10,2
Bn-scaff_16468_1-p308268
10,5
Bn-scaff_16273_1-p331417
Bn-scaff_16766_1-p150594
12,1
Bn-scaff_16766_1-p211870
Bn-scaff_16142_1-p90468
Bn-scaff_16142_1-p47324
Bn-scaff_17637_1-p230619
13,8
Bn-scaff_17637_1-p170031
Bn-scaff_16287_1-p294213
Bn-scaff_16287_1-p394395
14,3
Bn-scaff_21250_1-p164882
14,9
Bn-scaff_18275_1-p1210524
16,6
Bn-scaff_18275_1-p1278049
Bn-scaff_19242_1-p421335
24,0
Bn-scaff_19242_1-p390109
Bn-scaff_16231_1-p1235878
35,2
Bn-scaff_16231_1-p1209255
36,5
Bn-scaff_16231_1-p897380
38,9
Bn-scaff_16231_1-p662722
39,8
Bn-scaff_15923_1-p635095
44,0
Bn-scaff_15923_1-p489042
Bn-scaff_15923_1-p46551
44,6
Bn-A08-p17857870
46,4
Bn-A08-p17724619
47,2
Bn-scaff_16174_1-p133998
Bn-scaff_16174_1-p653015
48,5
Bn-scaff_16174_1-p1072915
49,7
Bn-scaff_16174_1-p1163637
50,3
Bn-scaff_16174_1-p1185135
Bn-scaff_16174_1-p1265134
50,9
Bn-scaff_16174_1-p1308949
Bn-scaff_16174_1-p1309245
Bn-scaff_16174_1-p1444980
51,5
Bn-scaff_16174_1-p1551146
52,0
Bn-A06-p9094415
52,5
Bn-scaff_16770_1-p1746549
53,7
Bn-scaff_16770_1-p2985548
55,4
Bn-scaff_16770_1-p4035684
59,6
Bn-scaff_16770_1-p4068629
60,0
Bn-scaff_16770_1-p4353199
60,4
Bn-scaff_16361_1-p43353
61,6
Bn-scaff_16361_1-p247366
62,0
Bn-scaff_16361_1-p901728
62,8
Bn-scaff_16361_1-p1122025
64,2
Bn-scaff_16361_1-p1504169
64,6
Bn-scaff_16361_1-p1713891
65,1
Bn-scaff_18607_1-p283263
0,0
Bn-scaff_16130_3-p5466
0,6
Bn-scaff_16468_1-p308268
Bn-scaff_18310_1-p1283064
Bn-scaff_16273_1-p331417
3,9
Bn-scaff_16766_1-p150594
Bn-scaff_16766_1-p211870
Bn-scaff_15712_9-p79189
Bn-scaff_16287_1-p294213
4,5
Bn-scaff_17637_1-p230619
5,1
Bn-scaff_17637_1-p112306
5,7
Bn-scaff_17637_1-p170031
6,6
Bn-scaff_16142_1-p47324
8,1
Bn-scaff_20563_1-p11145
Bn-scaff_16287_1-p394395
9,4
Bn-scaff_21250_1-p281415
Bn-scaff_20497_1-p37597
12,6
Bn-scaff_20497_1-p89956
Bn-scaff_18275_1-p1210524
13,7
Bn-A08-p14694395
17,0
Bn-scaff_17227_1-p626934
25,4
Bn-scaff_17227_1-p700248
27,4
Bn-scaff_17227_1-p927882
28,5
Bn-scaff_17227_1-p1391195
29,5
Bn-scaff_21786_1-p160470
30,5
Bn-scaff_16231_1-p2303901
31,5
Bn-A08-p19541990
32,4
Bn-scaff_16231_1-p1235878
Bn-scaff_16231_1-p1209255
33,0
Bn-scaff_16231_1-p1176514
Bn-scaff_16231_1-p896944
36,1
Bn-scaff_16231_1-p897380
Bn-scaff_16231_1-p795518
37,0
Bn-scaff_16231_1-p662722
39,5
Bn-A04-p7275318
45,0
Bn-scaff_15923_1-p635095
50,8
Bn-scaff_17565_1-p6212
Bn-scaff_15923_1-p46551
52,7
Bn-scaff_16174_1-p133998
56,5
Bn-scaff_16174_1-p1072915
Bn-scaff_16174_1-p1163637
Bn-scaff_16174_1-p1185135
60,3
Bn-scaff_16174_1-p1247659
Bn-scaff_16174_1-p1265134
Bn-scaff_16174_1-p1308949
Bn-scaff_16174_1-p1309245
62,2
Bn-scaff_16174_1-p1444980
Bn-scaff_16770_1-p1746549
64,4
Bn-Scaffold000460-p5716
66,5
Bn-A09-p25113520
67,1
Bn-scaff_16770_1-p3197948
68,4
Bn-scaff_16770_1-p2985548
69,0
Bn-scaff_16770_1-p2948588
Bn-scaff_16770_1-p2861442
69,6
Bn-scaff_16361_1-p2291795
88,6
Bn-scaff_16361_1-p2750042
89,8
Bn-scaff_16361_1-p3350949
Bn-scaff_16361_1-p3354234
91,8
Bn-scaff_16361_1-p3355395
Bn-scaff_16197_1-p3104187
92,6
Bn-scaff_16197_1-p3019951
94,9
Bn-scaff_16197_1-p3006933
Bn-scaff_16197_1-p2962341
Bn-scaff_16197_1-p2640097
98,7
Bn-scaff_16197_1-p2602143
Bn-scaff_21003_1-p447466
0,0
Bn-scaff_16468_1-p308268
Bn-scaff_16273_1-p331417
0,4
Bn-scaff_15712_9-p79189
Bn-scaff_16766_1-p150594
2,9
Bn-scaff_16766_1-p211870
Bn-scaff_17637_1-p170031
9,8
Bn-scaff_16287_1-p394395
Bn-scaff_17637_1-p230619
10,1
Bn-scaff_17637_1-p112306
10,6
Bn-scaff_20563_1-p11145
11,2
Bn-scaff_16142_1-p47324
12,0
Bn-scaff_17257_1-p98652
12,8
Bn-scaff_21250_1-p281415
13,4
Bn-scaff_16142_1-p90468
13,9
Bn-scaff_20497_1-p37597
18,1
Bn-scaff_20497_1-p89956
Bn-scaff_20497_1-p118636
18,6
Bn-scaff_18275_1-p1278049
20,2
Bn-scaff_22350_1-p80848
21,2
Bn-scaff_19242_1-p421335
28,3
Bn-scaff_19242_1-p390109
Bn-scaff_16231_1-p1176514
46,1
Bn-scaff_16231_1-p678599
49,9
Bn-scaff_15923_1-p635095
54,1
Bn-scaff_16174_1-p1163637
58,6
Bn-scaff_16174_1-p1185135
59,9
Bn-scaff_16361_1-p2750042
83,3
Bn-scaff_16361_1-p3350949
Bn-scaff_16361_1-p3354234
87,3
Bn-scaff_16361_1-p3355395
Bn-scaff_16197_1-p3019951
92,4
Bn-scaff_16197_1-p3006933
92,7
Bn-scaff_16197_1-p3026053
93,1
Bn-scaff_16197_1-p2962341
94,2
Bn-scaff_16197_1-p2958264
94,8
Bn-scaff_16197_1-p2468601
102,5
Bn-scaff_16197_1-p2435820
103,1
Bn-scaff_16197_1-p2406247
Bn-scaff_16197_1-p2436020
106,4
Bn-scaff_16197_1-p2593050
109,0
Bn-scaff_16197_1-p2582618
Bn-scaff_16197_1-p2481235
112,3
Bn-scaff_16197_1-p2365578
114,8
Bn-scaff_16197_1-p2365526
Bn-scaff_16197_1-p1904811
118,4
Bn-scaff_16197_1-p1850852
119,1
Bn-scaff_16197_1-p1381278
Bn-scaff_16197_1-p1380700
121,9
Bn-scaff_16197_1-p1348777
Bn-scaff_16197_1-p1695268
Bn-scaff_16197_1-p697008
126,7
Bn-scaff_16197_1-p637120
127,7
Bn-scaff_16197_1-p504820
Bn-scaff_16197_1-p625764
128,2
Bn-scaff_16197_1-p553997
128,7
Bn-scaff_16197_1-p515133
129,2
Bn-scaff_16197_1-p273402
130,7
Bn-scaff_16445_1-p2022692
Bn-scaff_16361_1-p2016133
66,8
Bn-scaff_16361_1-p2052324
67,3
Bn-scaff_16361_1-p2256702
68,6
Bn-A09-p29454619
69,0
Bn-scaff_16361_1-p2750042
Bn-scaff_16361_1-p3350949
Bn-scaff_16361_1-p3354234
73,6
Bn-scaff_16361_1-p3355395
Bn-scaff_16361_1-p3397399
76,1
Bn-scaff_16197_1-p3259829
Bn-scaff_16197_1-p3019951
77,7
Bn-scaff_16197_1-p3006933
78,5
Bn-scaff_16197_1-p2962341
80,2
Bn-scaff_16197_1-p2911020
Bn-scaff_16197_1-p2602143
82,1
Bn-scaff_16197_1-p2493450
Bn-scaff_16197_1-p2436020
82,7
Bn-scaff_16197_1-p2435820
Bn-scaff_16197_1-p2406247
82,8
Bn-scaff_16197_1-p2521968
83,1
Bn-scaff_16197_1-p2468601
Bn-scaff_16197_1-p2330796
83,7
Bn-scaff_16197_1-p2144692
84,3
Bn-scaff_16197_1-p1850852
Bn-scaff_16197_1-p1695268
85,3
Bn-scaff_16197_1-p1381278
86,5
Bn-scaff_16197_1-p1093609
88,2
Bn-scaff_16197_1-p974627
88,8
Bn-scaff_16197_1-p836847
90,0
Bn-scaff_16197_1-p784797
92,5
Bn-scaff_16197_1-p625764
Bn-scaff_16197_1-p561660
93,8
Bn-scaff_16197_1-p553997
95,9
Bn-scaff_16197_1-p515133
96,6
Bn-scaff_16197_1-p504820
Bn-scaff_16197_1-p418355
97,0
Bn-scaff_16197_1-p273402
97,4
Bn-scaff_16197_1-p135491
97,9
Bn-scaff_16445_1-p2635783
100,2
Bn-scaff_16445_1-p2523413
103,8
Bn-scaff_16445_1-p2132184
105,6
Bn-scaff_16445_1-p2022692
106,0
Bn-scaff_16445_1-p1836752
106,9
Bn-scaff_16445_1-p1836681
Bn-scaff_16445_1-p1640811
Bn-scaff_16445_1-p1603116
110,2
Bn-scaff_16445_1-p1602173
Bn-scaff_16445_1-p1459241
Bn-scaff_16445_1-p1451181
112,3
Bn-scaff_16445_1-p1450260
Bn-scaff_16445_1-p1441125
113,9
Bn-scaff_16445_1-p1342951
114,5
Bn-A09-p34247590
116,4
Bn-scaff_16445_1-p1085018
Bn-scaff_16445_1-p932151
118,3
Bn-scaff_16445_1-p895858
121,8
Bn-scaff_16445_1-p852506
Bn-scaff_16445_1-p699461
124,4
Bn-scaff_16445_1-p585598
125,1
Bn-scaff_16445_1-p584652
Bn-scaff_16445_1-p478398
126,0
137,2
Bn-scaff_16445_1-p1548555
140,1
Bn-scaff_16445_1-p1836752
140,8
Bn-scaff_16197_1-p2911020
99,0
Bn-scaff_16445_1-p1836681
Bn-scaff_16197_1-p2893303
99,3
Bn-scaff_16445_1-p1640811
146,5
Bn-scaff_16197_1-p2487005
Bn-scaff_16445_1-p1603116
101,8
Bn-scaff_16197_1-p2468601
147,2
Bn-scaff_16445_1-p1602173
Bn-scaff_16197_1-p2435820
104,9
Bn-scaff_16445_1-p853363
151,4
Bn-scaff_16197_1-p2406247
Bn-scaff_16445_1-p1062261
153,3
Bn-scaff_16445_1-p2523413
Bn-scaff_26139_1-p376956
154,3
Bn-scaff_16445_1-p2218971
114,3
Bn-scaff_16445_1-p1085018
157,6
Bn-scaff_16445_1-p2132184
Bn-scaff_16445_1-p895858
159,5
Bn-scaff_16445_1-p1640811
Bn-scaff_16445_1-p728936
161,7
Bn-scaff_16445_1-p1603116
116,8
Bn-scaff_16445_1-p766708
162,4
Bn-scaff_16445_1-p1602173
Bn-scaff_16445_1-p852506
164,5
Bn-scaff_26139_1-p376956
118,0
Bn-scaff_16445_1-p555461
166,3
Bn-scaff_16445_1-p523337
Bn-scaff_16445_1-p268218
120,0
Bn-scaff_16445_1-p437285
171,5
Bn-scaff_16445_1-p363187
Bn-scaff_16445_1-p362991
173,4
Bn-scaff_16445_1-p414675
Bn-scaff_20947_1-p93326
176,5
Bn-scaff_16445_1-p312395
180,1
Bn-scaff_21269_1-p311160
185,2
Bn-scaff_16389_1-p585040
190,2
Bn-scaff_16389_1-p394420
192,6
Bn-scaff_16389_1-p740326
194,4
Bn-scaff_16021_1-p585766
197,5
Bn-scaff_16445_1-p362991
127,9
Bn-scaff_16445_1-p414675
Bn-scaff_16445_1-p263067
130,5
Bn-scaff_20947_1-p146783
134,6
Bn-scaff_20947_1-p93326
Bn-A09-p35368467
136,3
Bn-scaff_21269_1-p143121
137,7
Bn-scaff_21269_1-p306028
138,7
Bn-scaff_16389_1-p394420
144,9
Bn-scaff_16389_1-p740326
Bn-scaff_16389_1-p526483
146,8
Bn-scaff_16389_1-p688405
Bn-scaff_16389_1-p688011
Bn-scaff_16021_1-p167469
151,6

## Slide 19
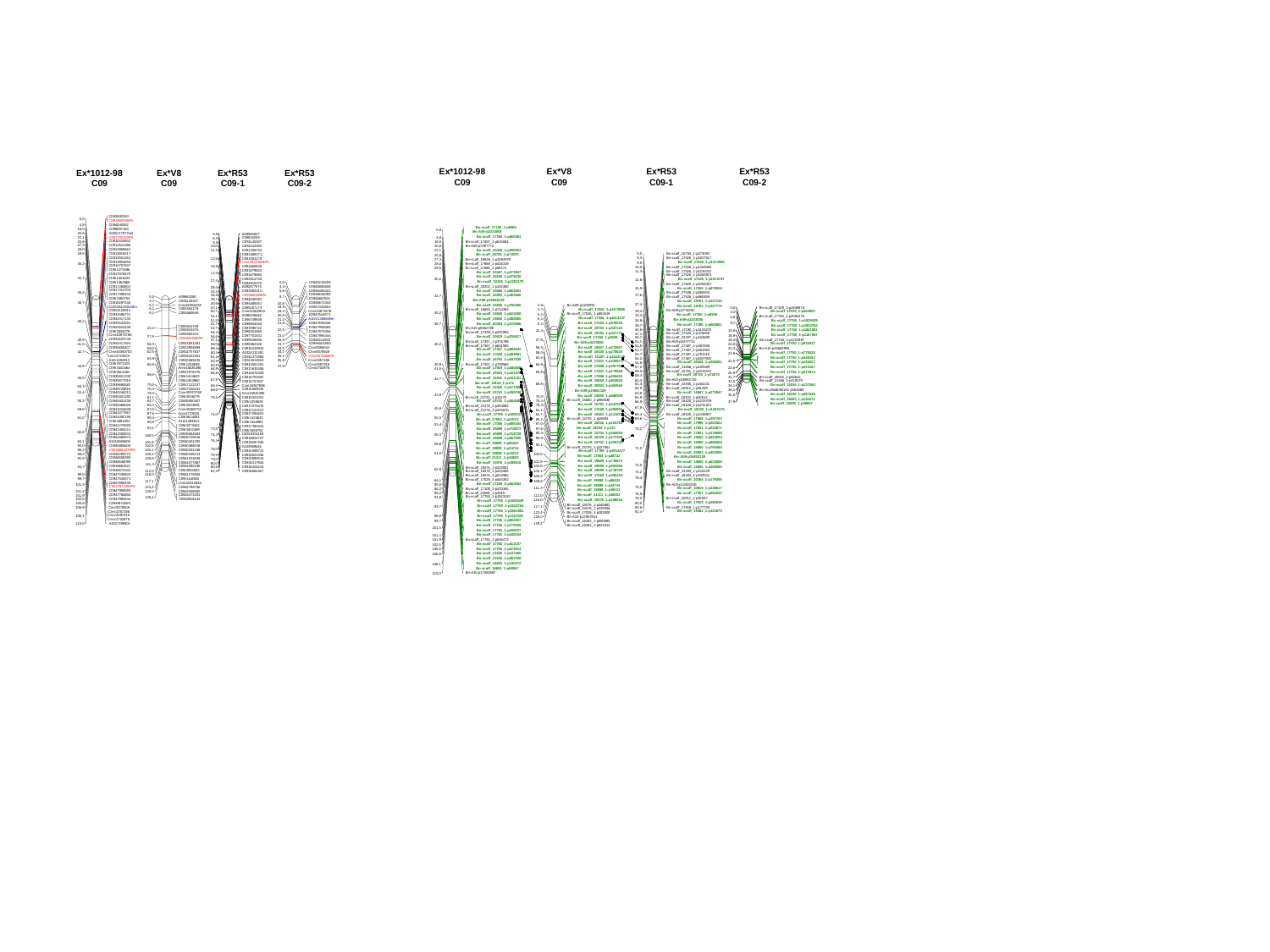

Ex*1012-98
C09
Ex*V8
C09
Ex*R53
C09-1
Ex*R53
C09-2
Bn-scaff_17190_1-p9206
0,0
Bn-A09-p3233005
Bn-scaff_17190_1-p660881
4,8
Bn-scaff_17487_1-p615064
18,0
Bn-A09-p7087772
20,8
Bn-scaff_20428_1-p256051
22,1
Bn-scaff_28725_1-p72570
25,8
Bn-scaff_18839_1-p1092979
27,3
Bn-scaff_17869_1-p416319
28,0
Bn-scaff_17888_1-p65372
29,5
Bn-scaff_15847_1-p273947
Bn-scaff_18100_1-p275630
30,2
Bn-scaff_18100_1-p1283175
Bn-scaff_20202_1-p182480
Bn-scaff_15650_1-p924603
Bn-scaff_20903_1-p453556
32,7
Bn-A09-p15852129
Bn-scaff_15650_1-p750450
Bn-scaff_15650_1-p714363
35,2
Bn-scaff_15650_1-p624000
Bn-scaff_15650_1-p368565
Bn-scaff_20354_1-p179456
36,7
Bn-A10-p5662796
Bn-scaff_17339_1-p292992
Bn-scaff_20619_1-p238617
Bn-scaff_17367_1-p376798
40,2
Bn-scaff_17367_1-p601905
Bn-scaff_17367_1-p684032
Bn-scaff_17048_1-p294991
Bn-scaff_16793_1-p547539
Bn-scaff_17367_1-p709565
40,9
Bn-scaff_17919_1-p280830
41,5
Bn-scaff_19483_1-p121870
Bn-scaff_16042_1-p101781
42,7
Bn-scaff_19154_1-p172
Bn-scaff_16325_1-p177408
Bn-scaff_15743_1-p255740
43,9
Bn-scaff_23720_1-p13275
Bn-scaff_15743_1-p284685
Bn-scaff_21276_1-p354893
45,8
Bn-scaff_21276_1-p303670
Bn-scaff_17799_1-p2932477
50,3
Bn-scaff_17554_1-p28732
Bn-scaff_17088_1-p455184
53,4
Bn-scaff_19899_1-p719873
Bn-scaff_19899_1-p719729
55,3
Bn-scaff_19899_1-p657806
Bn-scaff_19899_1-p86432
59,6
Bn-scaff_19899_1-p34734
Bn-scaff_19899_1-p16013
61,0
Bn-scaff_21311_1-p38653
Bn-scaff_15576_1-p196618
Bn-scaff_15576_1-p323551
62,8
Bn-scaff_15576_1-p425585
Bn-scaff_15576_1-p814965
Bn-scaff_17028_1-p444352
64,1
Bn-scaff_17109_2-p364002
85,0
Bn-scaff_17109_2-p370345
86,2
Bn-scaff_23066_1-p3540
89,2
Bn-scaff_17750_1-p1923167
91,9
Bn-scaff_17750_1-p1839429
Bn-scaff_17750_1-p1810764
93,7
Bn-scaff_17750_1-p1801981
Bn-scaff_17750_1-p1167557
98,0
Bn-scaff_17750_1-p932637
99,7
Bn-scaff_17750_1-p779325
101,3
Bn-scaff_17750_1-p436527
Bn-scaff_17750_1-p448154
101,4
Bn-scaff_17750_1-p636473
101,9
Bn-scaff_17750_1-p413107
102,5
Bn-scaff_17750_1-p274914
105,6
Bn-scaff_15783_1-p273092
0,0
Bn-scaff_17526_1-p1827517
8,3
Bn-scaff_17526_1-p1474906
9,5
Bn-scaff_17526_1-p1469380
10,0
Bn-scaff_17526_1-p1235752
11,3
Bn-scaff_17526_1-p1063974
Bn-scaff_17526_1-p1014747
12,6
Bn-scaff_17526_1-p1091087
Bn-scaff_17526_1-p879036
15,9
Bn-scaff_17526_1-p860504
17,6
Bn-scaff_17526_1-p860459
Bn-scaff_19783_1-p327125
27,4
Bn-scaff_19783_1-p327775
Bn-A09-p2733282
29,4
Bn-scaff_17190_1-p9206
33,3
Bn-A09-p3233005
34,8
Bn-scaff_17190_1-p660881
39,7
Bn-scaff_17190_1-p1134270
40,6
Bn-scaff_17423_1-p376558
47,1
Bn-scaff_23207_1-p153689
50,7
Bn-A09-p5327731
51,1
Bn-scaff_17487_1-p487836
51,5
Bn-scaff_17487_1-p561594
51,7
Bn-scaff_17487_1-p781216
53,7
Bn-scaff_17487_1-p1927920
54,2
Bn-scaff_20428_1-p256051
54,6
Bn-scaff_21656_1-p109389
57,2
Bn-scaff_15701_1-p1015232
59,0
Bn-scaff_28725_1-p72570
59,4
Bn-A03-p25952725
60,3
Bn-scaff_22835_1-p164231
61,4
Bn-scaff_16912_1-p94309
62,9
Bn-scaff_15847_1-p273947
63,9
Bn-scaff_18100_1-p50512
64,9
Bn-scaff_18100_1-p1215709
66,8
Bn-scaff_18100_1-p1241201
67,8
Bn-scaff_18100_1-p1283175
Bn-scaff_18100_1-p1336807
68,4
Bn-scaff_17888_1-p397760
69,6
Bn-scaff_17996_1-p204422
Bn-scaff_17801_1-p220871
70,2
Bn-scaff_17801_1-p178058
Bn-scaff_15650_1-p924603
Bn-scaff_15650_1-p886508
Bn-scaff_15650_1-p750450
71,0
Bn-scaff_20903_1-p453556
Bn-A09-p15852129
Bn-scaff_15650_1-p624000
73,5
Bn-scaff_15650_1-p368565
Bn-scaff_23293_1-p215229
74,2
Bn-scaff_18424_1-p534531
76,4
Bn-scaff_20354_1-p179456
Bn-A04-p12452535
78,0
Bn-scaff_20619_1-p238617
Bn-scaff_17367_1-p684032
78,5
Bn-scaff_20972_1-p92557
79,0
Bn-scaff_17919_1-p280830
80,0
Bn-scaff_17919_1-p177730
80,6
Bn-A09-p1188091
0,0
Bn-scaff_17109_1-p1948974
0,0
Bn-scaff_17526_1-p1474906
3,7
Bn-scaff_17109_2-p364002
4,3
Bn-scaff_17526_1-p952618
5,1
Bn-scaff_17750_1-p2082478
5,6
Bn-scaff_17526_1-p1014747
6,1
Bn-scaff_17750_1-p1839429
Bn-scaff_17526_1-p879036
9,7
8,1
Bn-scaff_17750_1-p1810764
Bn-scaff_19783_1-p327125
Bn-scaff_17750_1-p1801981
10,6
22,4
Bn-scaff_19783_1-p327775
Bn-scaff_17750_1-p1167557
18,9
Bn-scaff_17190_1-p9206
Bn-scaff_17750_1-p1107897
19,4
27,9
Bn-A09-p3233005
Bn-scaff_17750_1-p932637
20,8
Bn-scaff_15847_1-p273947
56,3
Bn-A10-p15606935
21,3
Bn-scaff_18100_1-p275630
58,0
Bn-scaff_17750_1-p779325
21,9
Bn-scaff_18100_1-p1283175
62,5
Bn-scaff_17750_1-p448154
22,5
Bn-scaff_17801_1-p220871
Bn-scaff_17750_1-p436527
64,9
Bn-scaff_17888_1-p397760
Bn-scaff_17750_1-p413107
23,0
Bn-scaff_17801_1-p178058
65,8
Bn-scaff_17750_1-p274914
26,8
Bn-scaff_17996_1-p204422
Bn-scaff_28053_1-p60542
31,7
Bn-scaff_15650_1-p924603
Bn-scaff_21636_1-p119179
33,5
68,6
Bn-scaff_21636_1-p137260
Bn-scaff_20903_1-p453556
34,1
Bn-Scaffold000191-p154088
35,1
Bn-A09-p15852129
Bn-scaff_21636_1-p497038
35,8
Bn-scaff_15650_1-p886508
70,0
Bn-scaff_16693_1-p145372
Bn-scaff_15650_1-p908638
70,4
37,9
Bn-scaff_16693_1-p58067
Bn-scaff_16793_1-p547539
79,3
Bn-scaff_17048_1-p294991
81,1
Bn-scaff_19483_1-p121870
84,7
Bn-scaff_15743_1-p26884
85,2
Bn-scaff_16042_1-p101781
87,0
Bn-scaff_19154_1-p172
87,6
Bn-scaff_15743_1-p284685
90,4
Bn-scaff_16325_1-p177408
90,9
Bn-scaff_15743_1-p255740
93,1
Bn-scaff_15743_1-p227851
Bn-scaff_17799_1-p2932477
100,5
Bn-scaff_17554_1-p28732
Bn-scaff_19899_1-p719873
101,8
Bn-scaff_19899_1-p657806
102,6
Bn-scaff_19899_1-p719729
103,1
Bn-scaff_17088_1-p455184
105,2
Bn-scaff_19899_1-p86432
109,8
Bn-scaff_19899_1-p34734
111,3
Bn-scaff_19899_1-p16013
Bn-scaff_21311_1-p38653
112,0
Bn-scaff_15576_1-p196618
116,0
Bn-scaff_15576_1-p240863
117,1
Bn-scaff_15576_1-p225938
Bn-scaff_19483_1-p121870
81,4
Bn-scaff_17028_1-p283000
123,4
Bn-A10-p12957851
126,0
Bn-scaff_16362_1-p650685
129,4
Bn-scaff_16362_1-p621813
Bn-scaff_21636_1-p137260
106,9
Bn-scaff_21636_1-p497038
Bn-scaff_16693_1-p145372
108,1
Bn-scaff_16693_1-p58067
Bn-A10-p17500897
110,0
Ex*1012-98
C09
Ex*V8
C09
Ex*R53
C09-1
Ex*R53
C09-2
C093592310
0,0
C093600506PA
C094040382
4,8
C096897184
18,0
A095747977het
20,8
C097701632PA
22,1
C0910318942
25,8
C0912521386
27,3
C0912959661
28,0
C0915916217
29,5
C0913551261
C0913904859
30,2
C0914757837
C09r1274596
C0917078475
C09r1424633
32,7
C09r1452960
C0917284601
C0917312703
35,2
C0917390433
C09r1580754
C0919297345
36,7
A105304433hetEx
C0921120814
C0931596731
C0932317130
40,2
C0932542691
C0932622436
C09r2036279
Cnnr40973788
C0932633748
40,9
C0934317924
41,5
C0935650407
Cnnr30360753
42,7
Unnr2723535
A10r1494912
C09r3373423
43,9
C09r3440463
C09r3614452
C0935821709
45,8
C0935877016
C0939684583
50,3
C0939729816
C0940246213
53,4
C0940401292
C0940401436
55,3
C0940468028
C0941024639
59,6
C0941077867
C0941092195
61,0
C09r4091003
C0941275920
C0941404221
62,8
C0941500833
C0941890974
A1012865606
64,1
C0945865608
85,0
C0945861679PA
86,2
C0946289774
89,2
C0946558390
91,9
C0946646099
C0946663511
93,7
C0946672343
C0947330603
98,0
C0947544571
99,7
C0947694436
101,3
C0947972456PA
C0947958585
101,4
C0947784850
101,9
C0947994444
102,5
C0948114920
105,6
A09565847
0,0
C09816258
8,3
C091145307
9,5
C091152093
10,0
C091338743
11,3
C091489171
C091554179
12,6
Cnnr29370694PA
C091660648
15,9
C091679820
17,6
C091679865
C092814749
27,4
C092815376
A092677575
29,4
C093592310
33,3
C093600506PA
34,8
C094040382
39,7
C094485061
40,6
C095187173
47,1
Cnnr51603954
50,7
A095008448
51,1
C096749838
51,5
C096843040
51,7
C097096722
53,7
C098201860
54,2
C097701632
54,6
C099026506
57,2
C099382326
59,0
C0910318942
59,4
A0324311391
60,3
C0911373585
61,4
C0912618115
62,9
C0913551261
63,9
C0913635358
64,9
C0914675428
66,8
C0914701691
67,8
C0914757837
Cnnr34947896
68,4
C0915569035
69,6
Annr45535390
C0915322451
70,2
C09r1255639
C0917078475
C0917122237
C0917284601
71,0
C09r1424633
C09r1452960
C0917390433
73,5
C09r1580754
C0918151150
74,2
C0918924707
76,4
C0919297345
A103969446
78,0
C0931596731
C0932622436
78,5
C0932889314
79,0
C0934317924
80,0
C0934424224
80,6
C0945218259
0,0
C0945865608
4,3
C0946406422
5,6
C0946646099
A09963280
0,0
9,7
C0946663511
C091145307
3,7
C0946672343
10,6
Cnnr62556258
5,1
C0947330603
18,9
C091554179
6,1
Cnnr34873678
19,4
C091660648
8,1
C0947544571
20,8
A10r2136866het
21,3
C0947694436
21,9
C092814749
C0947958585
22,4
22,5
C092815376
C0947972456
C093592310
C0947994444
23,0
27,9
C093600506PA
C0948114920
26,8
C0913551261
C0948401850
56,3
31,7
C0913904859
Cnnr9356042
58,0
33,5
C0914757837
Cnnr9378509
62,5
34,1
C0915322451
Cnnr9478864PA
35,1
64,9
C0915569035
Cnnr2357198
35,8
C09r1255639
Cnnr2597318
65,8
37,9
Annr45535390
Cnnr2702978
C0917078475
68,6
C09r1424633
C09r1452960
C0917122237
70,0
C0917100181
70,4
Cnnr40973788
79,3
C09r2036279
81,1
C0935650407
84,7
C09r3293536
85,2
Cnnr30360753
87,0
Unnr2723535
87,6
C09r3614452
90,4
A10r1494912
90,9
C09r3373423
93,1
C09r3401369
C0939684583
100,5
C0939729816
C0940401292
101,8
C0940468028
102,6
C0940401436
103,1
C0940246213
105,2
C0941024639
109,8
C0941077867
111,3
C0941092195
C09r4091003
112,0
C0935650407
81,4
C0941275920
116,0
C09r4116925
117,1
Cnnr41612645
C0942799756
123,4
C0943206892
126,0
C0943473291
129,4
C0943501132
Cnnr9378509
106,9
Cnnr2357198
Cnnr2597318
108,1
Cnnr2702978
A1017299401
110,0
